# Secretome analysis of breast cancer-associated adipose tissue to identify paracrine regulators of breast cancer growth

**DOI:** 10.18632/oncotarget.17592

**Published:** 2017-05-03

**Authors:** Lapeire Lore, Hendrix An, Lecoutere Evelyne, Van Bockstal Mieke, Vandesompele Jo, Maynard Dawn, Braems Geert, Van Den Broecke Rudy, Müller Cathérine, Bracke Marc, Cocquyt Véronique, Denys Hannelore, De Wever Olivier

**Affiliations:** ^1^ Department of Medical Oncology, Ghent University Hospital, Ghent, Belgium; ^2^ Cancer Research Institute Ghent (CRIG), Ghent University Hospital, Ghent, Belgium; ^3^ Laboratory of Experimental Cancer Research, Department of Radiation Oncology and Experimental Cancer Research, Ghent University Hospital, Ghent, Belgium; ^4^ Department of Pathology, Ghent University Hospital, Ghent, Belgium; ^5^ Center for Medical Genetics, Ghent University Hospital, Ghent, Belgium; ^6^ Medical Genetics Branch, National Human Genome Research Institute, Bethesda, Maryland, USA; ^7^ Department of Gynecology, Ghent University Hospital, Ghent, Belgium; ^8^ Institut de Pharmacologie et de Biologie Structurale, Université de Toulouse, UPS, Toulouse, France

**Keywords:** adipose tissue, breast cancer, proliferation, secretome, palbociclib

## Abstract

Adipose tissue secretes a plethora of adipokines as evidenced by characterization of subcutaneous and visceral adipose tissue secretomes. However, adipose tissue composition and secretion pattern is depot and disease dependent, influencing the adipose tissue secretome. We investigated the secretome of cancer-associated adipose tissue (CAAT) explants from breast cancer patients and explored its role in breast cancer proliferation. CAAT proteins were identified by LC-MS/MS and human protein antibody arrays and stimulated proliferation of three breast cancer cell lines. Kinomics and transcriptomics of MCF-7 breast cancer cells treated with the secretome of CAAT revealed activation of Akt-, ERK- and JNK-pathways and differential expression of activator protein 1 (AP-1) and cAMP responsive element-binding protein (CREB) target genes. The cyclin-dependent kinase (CDK)4/6-inhibitor palbociclib significantly abrogated CAAT-enhanced breast cancer cell proliferation. Our work characterizes the specific breast CAAT protein secretome and reveals its pro-proliferative potency in breast cancer.

## INTRODUCTION

In breast cancer, adipose tissue is the main component of the tumor environment and the heterogeneous cell types of the adipose tissue stimulate breast cancer cells towards further progression. Adipocytes can drive breast cancer progression by lipid transfer, androgen-to-estrogen production and secretion of several adipokines [1–3]. Human adipose tissue-derived stem cells isolated from breast tumors promote breast cancer growth through secretion of several growth factors and interleukin(IL)-8 [4]. Endothelial progenitor cells derived from human adipose tissue enhance tumor growth and metastasis through increased tumor vasculature when co-injected with human breast cancer cells in orthotopic mouse models [5]. Adipose tissue macrophages linked with obesity and chronic inflammation are described as tumor growth promoters [6]. Secretome analysis of separate adipose tissue-derived cell types has been performed and can be useful for designing potential therapeutic strategies [7, 8]. However, they do not reflect the secretome of adipose tissue as a whole and neglect important bilateral communication between different adipose tissue cell types influencing the adipose tissue secretome.

Adipose tissue can be found throughout the body but its composition and secretion pattern differs and is influenced by its localisation in the body and disease states such as obesity and cancer [9–11]. This implicates that available adipose tissue secretome data cannot be simply extrapolated when studying the paracrine effects of breast CAAT.

We performed the first proteomic analysis of the human breast CAAT secretome and explored its influence on biological processes, pathways and transcription factors involved in breast cancer growth.

## RESULTS

### Secretome analysis of CAAT secreted soluble factors

**Figure 1:**
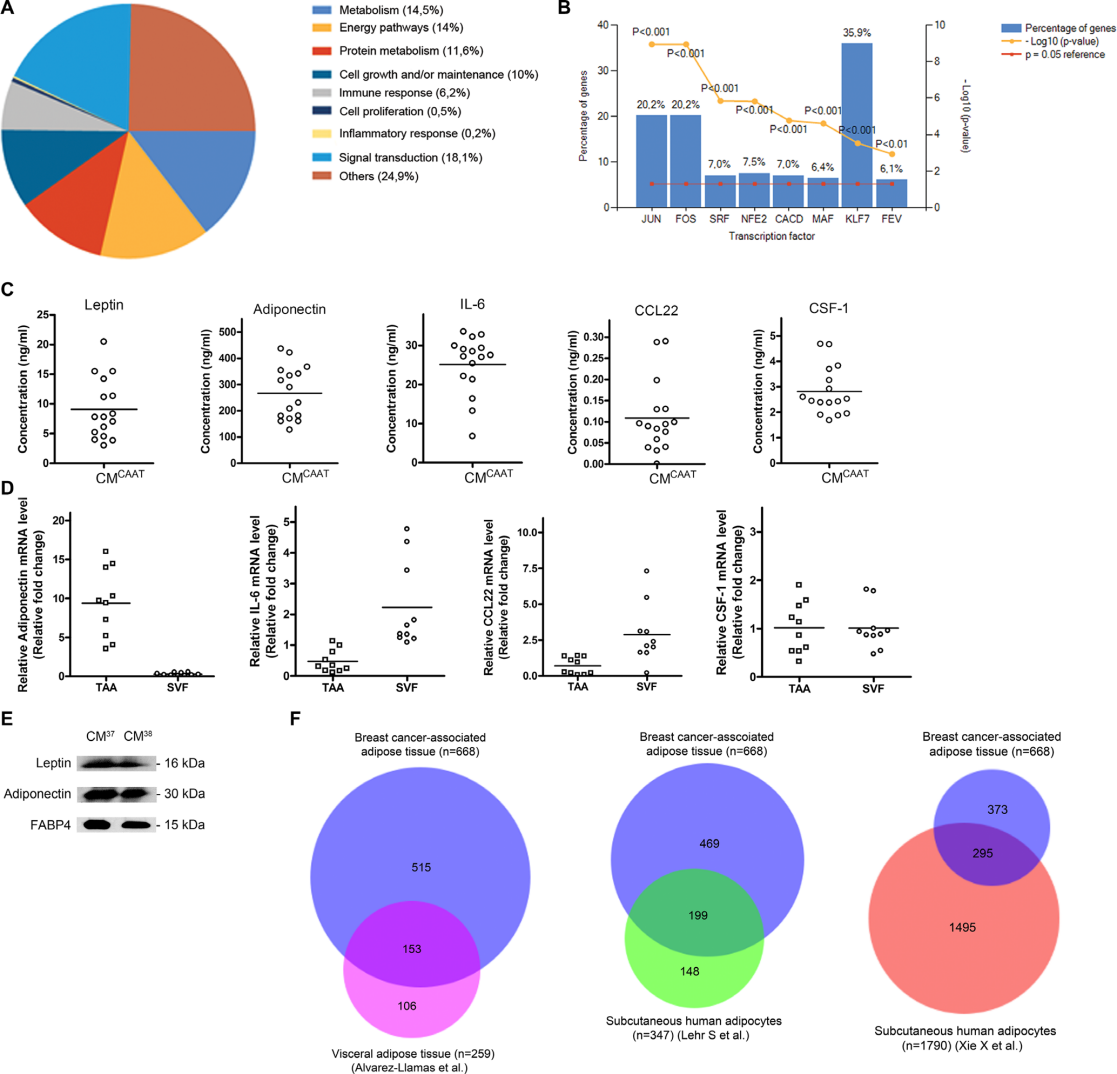
Secretome analysis of CAAT secreted soluble factors (**A**) pie chart, biological processes annotated to CAAT secreted factors. (**B**) bar chart, transcription factors annotated to CAAT secreted factors. (**C**) scatter plots of leptin, adiponectin, IL-6, CCL22 and CSF-1 concentrations in CM^CAAT^ from 16 breast cancer patients measured by ELISA. (**D**) scatter plots of relative mRNA levels of adiponectin, IL-6, CCL22 and CSF-1 in TAA and SVF of CAAT from 10 breast cancer patients. (**E**) Western blot analysis identifies leptin, adiponectin and FABP4 in CM^CAAT^ from 2 breast cancer patients. (**F**) Area-proportional Venn diagrams visualizing unique and common proteins between CAAT and three available data sets.

To identify adipose tissue secreted proteins, we analysed a discovery set of CM^CAAT^ from 6 patients from which 2 CM^CAAT^ were used for LC-MS/MS analysis and 4 CM^CAAT^ for antibody array as low levels of highly bioactive proteins are often not detected by gel-electrophoresis-based techniques [12]. A total of 668 proteins were identified (Supplementary Table 1) and proteins were assigned to several biological processes like signal transduction (18,1%), metabolism (14,5%), energy pathways (14%), protein metabolism (11, 6%), cell growth and/or maintenance (10%) and immune response (6,2%) (Figure 1A). CAAT proteins activate transcription factors like Krüppel-like factor 7 (KLF7) (36, 9%), JUN (20, 2%), FOS (20, 2%) and NFE2 (7, 5%) (Figure 1B). A detailed overview of the biological pathways and functions each of the 668 proteins belong to can be found in Supplementary Table 2. To validate these finding we made use of CM^CAAT^ from an additional 18 patients. In 16 patients, the concentration of 5 identified proteins was measured by ELISA, i.e. leptin (9.1 ± 5.1 ng/ml), adiponectin (267.2 ± 101.5 ng/ml), IL-6 (25.1 ± 7.5 ng/ml), CSF-1 (2.8 ± 1.0 ng/ml) and CCL22 (0.1 ± 0.1 ng/ml) (Figure 1C). Western blot identified leptin, adiponectin and fatty acid binding protein 4 (FABP4) in CM^CAAT^ of the two remaining patients (Figure 1E). As adipose tissue is composed of several cell types, we separated breast cancer-associated adipose tissue from 10 breast cancer patients into TAA and SVF. RT-qPCR revealed expression of adiponectin in TAA (35-fold higher expression in TAA vs SVF, *p* = 0.005). In contrast, a 5-fold higher IL-6 (*p* = 0.005) and a 4-fold higher CCL22 expression (*p* = 0.005) in SVF compared to TAA were observed. CSF-1 did not significantly differ in both fractions (*p* = 0.959) (Figure 1D). Comparison of our detected proteins in CAAT with the visceral adipose tissue secretome from Alvarez-Llamas et al. [13] containing 259 proteins revealed 153 common proteins (24,6%). CAAT proteins were also compared with the secretome of isolated human adipocytes from non-obese subcutaneous adipose tissue from Lehr et al. [14] and Xie et al. [15], and showed a 32,3% (199 proteins) and 15,8% (295 proteins) overlap respectively (Figure 1F). A detailed description of the common proteins between the different data sets is provided in Supplementary Table 3.

### CAAT stimulates proliferation of breast cancer cells

**Figure 2:**
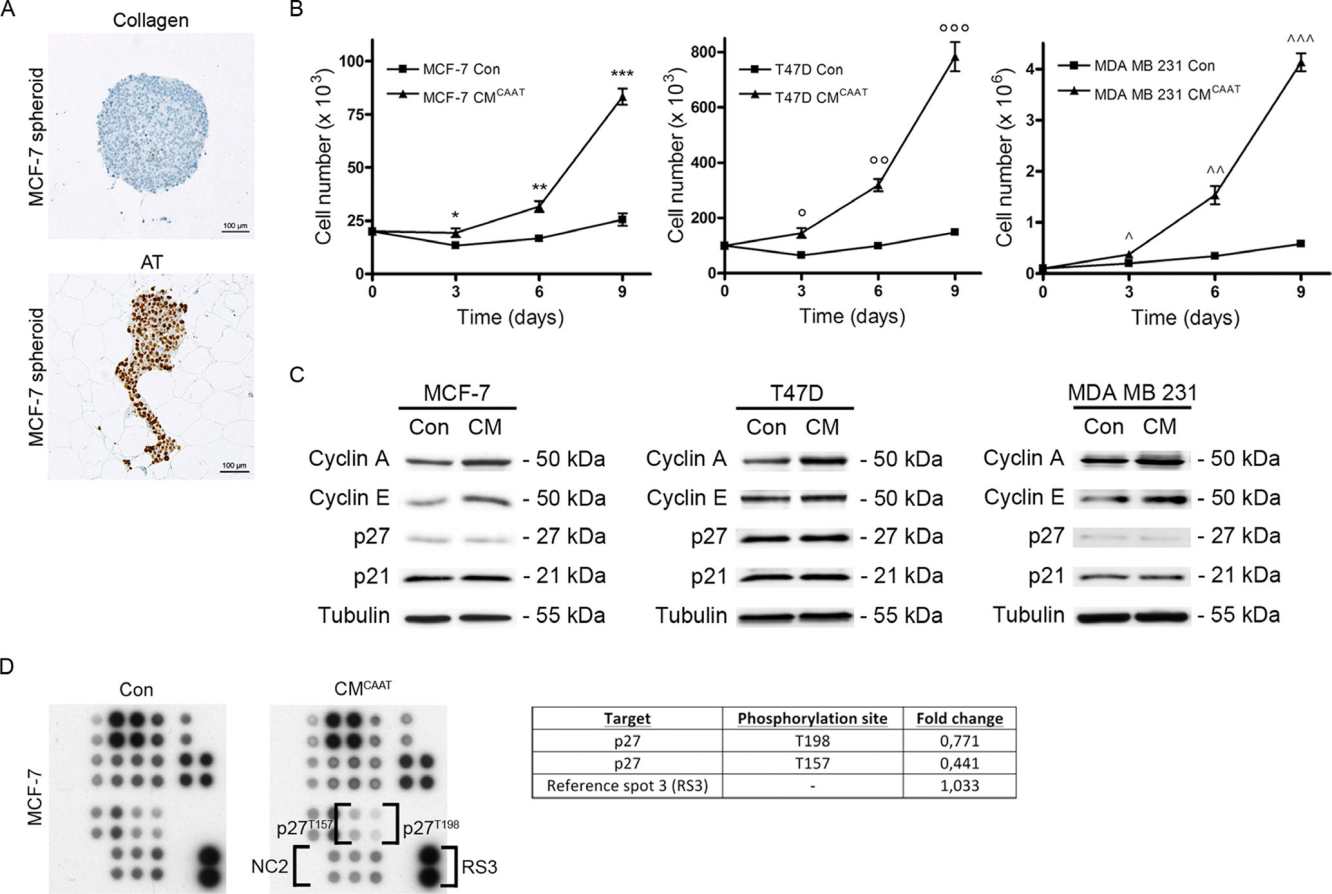
CAAT stimulates proliferation of breast cancer cells (**A**) Ki67 staining of MCF-7 spheroids cultured in CAAT or collagen type I (SC is 100μm). (**B**) graphs representing proliferation tests of MCF-7, T47D or MDA MB 231 cells treated with control medium (Con) or CM^CAAT^; *p = 0.0989, **p = 0.0093, ***p = 0.0003, *°p* = 0.0187, °*°p* = 0.0007, °°*°p* = 0.0003, ^*p* = 0.0327, ^^*p* = 0.0093, ^^*p* = 0.0001. (**C**) Western blot analysis of cyclin A, cyclin E, p27 and P21 in MCF-7, T47D or MDA MB 231 cells treated for 48 h with Con or CM^CAAT^, tubulin serves as internal control. The image shows the results of 1 of 3 repeats (*n* = 3) with each time 3 biological repeats. (**D**) phospho kinase array (part B) indicating phosphorylation levels of p27 in MCF-7 cells treated for 48 h with Con or CM^CAAT^, NC = negative control (Of note, NC1, RS1 and RS2 are shown in part A of the phospho kinase array displayed in Figure 2A of our previous publication [12]).

As JUN and FOS are both proto-oncogenes involved in cell proliferation, we investigated the effect of CAAT on breast cancer cell proliferation. MCF-7 aggregates were confronted with CAAT in native type I collagen, the main structural component of the mammary gland. Next to clear reorganisation of the aggregate, CAAT induces a strong proliferation rate of MCF-7 breast cancer cells as evidenced by Ki67-staining, with 88,1% of MCF-7 cells showing a positive nuclear signal. In contrast, MCF-7 aggregates not confronted with CAAT lost their proliferative ability after a few days of culture (difference CAAT to no CAAT = 86,9%, 95% CI = 83,1% to 90,7%, *p* < 0,0001) (Figure 2A). We next questioned if soluble factors secreted by CAAT could be responsible for the effects on proliferation as seen by direct co-culture. Treatment of three breast cancer cell lines with CM^CAAT^ led to a significant higher number of cells in time (Con vs CM^CAAT^; MCF-7 at day 9: 26 × 10^3^ ± 5 × 10^3^ vs 83 × 10^3^ ± 7 × 10^3^, *p* = 0.0003; T47D at day 9: 135 × 10^3^ ± 13 × 10^3^ vs 783 × 10^3^ ± 91 × 10^3^, *p* = 0.0003; MDA MB 231 at day 9: 580×10^3^ ± 60 × 10^3^ vs 4133 × 10^3^ ± 301 × 10^3^, *p* = 0.0001) (Figure 2B). Positive cell cycle regulators Cyclin A and Cyclin E were increased in CM^CAAT^ treated breast cancer cells compared to control, while negative cell cycle regulators p27 and p21 remained unchanged (Figure 2C). A phospho kinase array revealed less activation of p27 in MCF-7 breast cancer cells upon CM^CAAT^ treatment (Figure 2D).

### Regulation of AP-1 and CREB transcription factors in CAAT mediated breast cancer growth

**Figure 3:**
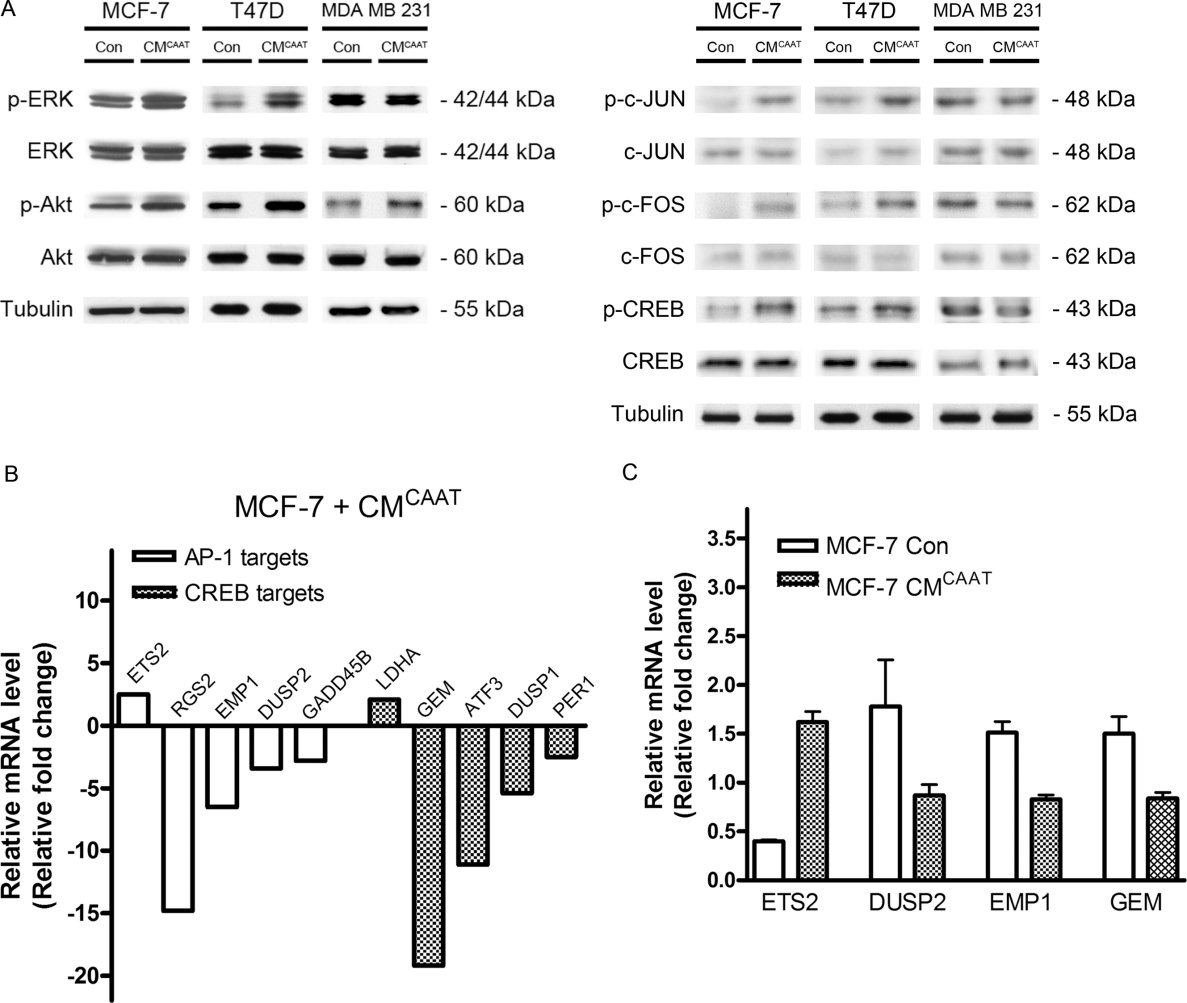
CAAT enhanced breast cancer proliferation correlates with differential activation of AP-1 and CREB dependent genes (**A**) Western blot analysis of (p-)Erk, (p-)Akt, (p-)c-JUN, (p-)c-FOS and (p-)CREB in MCF-7, T47D and MDA MB 231 cells treated for 48 h with Con or CM^CAAT^. (**B**) relative fold change of indicated AP-1 and CREB genes in MCF-7 cells treated for 48 h with CM^CAAT^ compared to control. (**C**) relative mRNA levels of indicated genes in MCF-7 cells treated for 48 h with Con or CM^CAAT^.

As CAAT soluble factors were able to stimulate breast cancer cell proliferation, we further explored the influence of CAAT on pro-proliferative pathways in breast cancer cells. As shown in our previous publication, phospho kinase screening revealed a clear activation of ERK1/2- (1.75-fold), Akt- (1.75-fold), JNK- (1.94-fold) and CREB- (2.02-fold) pathways in CM^CAAT^ treated MCF-7 cells when compared to control [16]. Western blot analysis confirmed these results in three breast cancer cell lines except for CREB activation in MDA MB 231 cells (Figure 3A). CAAT secretome analysis identified 113 proteins (20,2%) playing a role in JUN/FOS expression. Activation of c-JUN and c-FOS was confirmed by Western blot in both MCF-7 and T47D cells but not in MDA MB 231 cells. Activated c-Jun and c-Fos proteins form homo- or heterodimers binding the activator protein 1 (AP-1) binding site in DNA followed by transcriptional activation [17]. Micro-array analysis of MCF-7 cells treated with CM^CAAT^ revealed several AP-1 transcriptional targets of which the upregulated gene ETS2 (ETS proto-oncogene 2; 2.5-fold) and the downregulated genes RGS2 (regulator of G-protein signaling 2; 14.8-fold), EMP1 (epithelial membrane protein 1; 6.5-fold), DUSP2 (dual specificity phosphatase 2; 3.4-fold) and GADD45B (growth arrest and DNA damage inducible beta; 2.8-fold) are linked with a pro-proliferative signature in breast cancer [18–22] (Figure 3B). As CREB was clearly activated on phospho kinase screening and Western blot analysis, we also looked at CREB transcriptional genes in CM^CAAT^ treated MCF-7 cells and found an upregulation of LDHA (lactate dehydrogenase A; 2.1-fold) and downregulation of GEM (GTP binding protein overexpressed in skeletal muscle; 19.2-fold), ATF3 (activating transcription factor 3; 11.1-fold), DUSP1 (dual specificity phosphatase 1; 5.4-fold) and PER1 (period circadian clock 1; 2.5-fold), known to affect breast cancer proliferation and growth [23–29] (Figure 3B). Interestingly, protein analysis of CM^CAAT^ revealed 30 proteins (5,4%) involved in CREB-mediated transcription. RT-qPCR validated the differential expression of 4 of the above mentioned AP-1 and CREB transcriptional targets (Con vs. CM^CAAT^, *p* = 0.004 for all genes except DUSP2 *p* = 0.197) (Figure 3C).

### Impact of palbociclib on CAAT enhanced breast cancer cell proliferation

**Figure 4:**
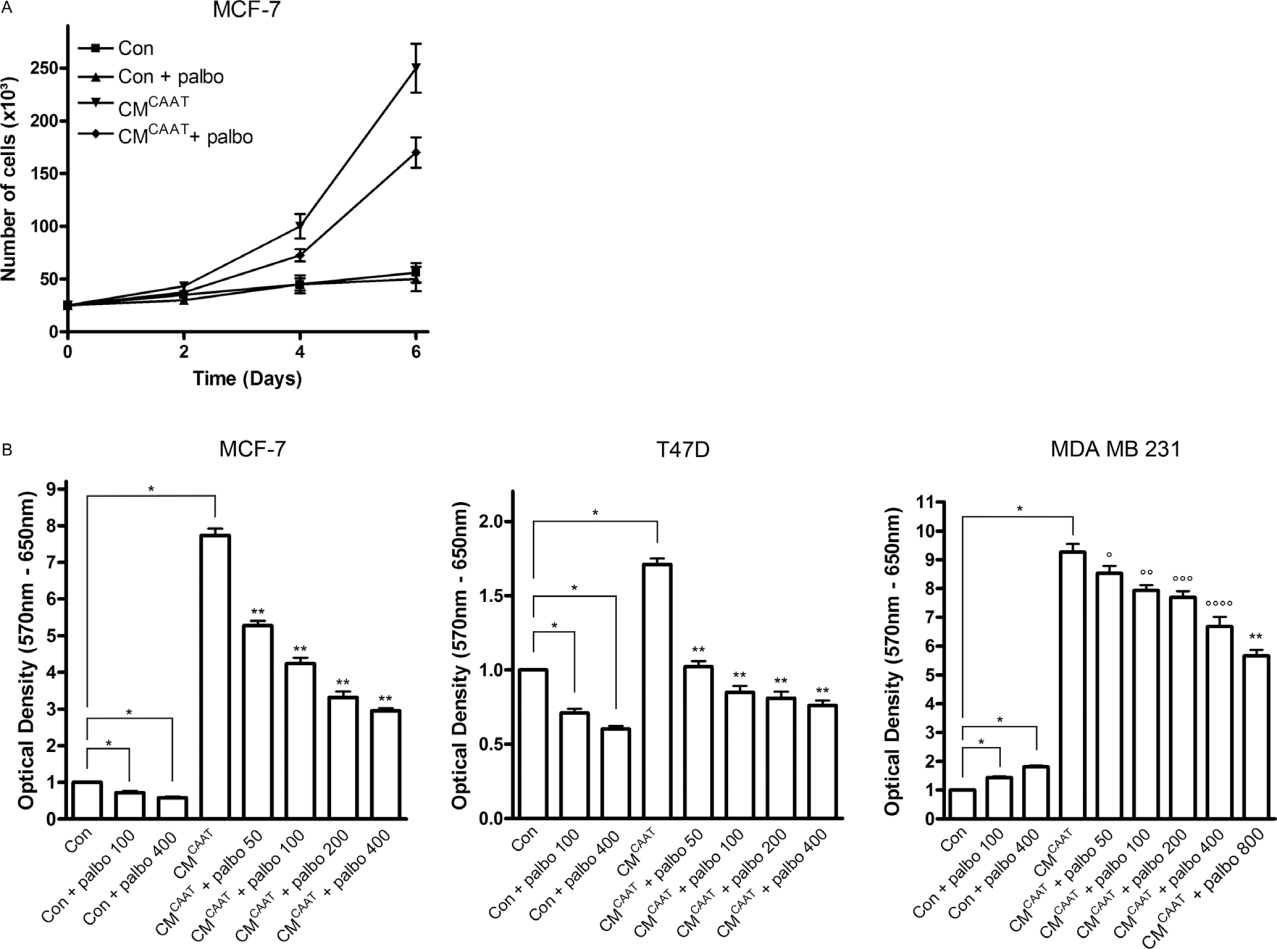
Palbociclib effectively inhibits CAAT enhanced breast cancer cell proliferation (**A**) graph representing proliferation test of MCF-7 cells treated with Con, Con + 100 nM palbociclib, CM^CAAT^ or CM^CAAT^ + 100 nM palbociclib. The graph represents the results of 1 biologic experiment with 3 replicates. (**B**) graphs representing 3-(4,5-dimethylthiazol-2-yl)-2,5-diphenyltetrazolium bromide (MTT)-tests of MCF-7, T47D or MDA MB 231 cells treated with Con, CM^CAAT^ and palbociclib at indicated concentrations; *p < 0.001, **p = 0.001, *°p* = 0.035, °*°p* = 0.009, °°*°p* = 0.005, °°°*°p* = 0.002.

Proliferation of cancer cells requires entry of the cell into the cell cycle comprising different sequential stages tightly controlled by cyclins and CDKs, amongst others. As CAAT proteins are able to significantly stimulate breast cancer cell proliferation (Figures 2A, 2B, 4A and 4B), we evaluated the effect of palbociclib, a selective CDK4/6-inhibitor, on CM^CAAT^ enhanced proliferation of MCF-7, T47D and MDA MB 231 breast cancer cells. Palbociclib significantly reduced the stimulatory effect of CAAT in all cell lines (MCF-7 and T47D: CM vs. CM + palbo, *p* = 0.001 for all palbociclib concentrations; MDA MB 231: CM vs. CM + palbo, *p* = 0.035 at 50 nM, *p* = 0.009 at 100 nM, *p* = 0.005 at 200 nM, *p* = 0.002 at 400 nM and *p* = 0.001 at 800 nM palbociclib) (Figure 4B).

## DISCUSSION

Several studies have described the secretome of isolated and *in vitro* cultured adipogenic differentiated mesenchymal stem cells, adipose tissue-derived adipocytes and stromal vascular fraction, and *ex vivo* cultured visceral adipose tissue [13, 14, 30, 31]. To our knowledge, we characterized for the first time the secretome of *ex vivo* cultured human breast cancer-associated adipose tissue and identified the capacity of the CAAT secretome to activate several pro-proliferative pathways in different breast cancer cell lines.

Our study has some limitations that need to be pointed out. First, we used CAAT from a limited number of breast cancer patients with diverse clinical and pathological characteristics (Supplementary Table 4). Second, we did not compare our results with normal breast adipose tissue. The main reason for this is that adipose tissue is a complicated and adaptive organ influenced by different factors form the environment making it difficult to determine ‘normal’ (breast) adipose tissue (see next paragraph). Third, our functional experiments are mainly performed with breast cancer cells cultured as adherent cells to plastic. We did not use 3D-tests with breast cancer cell organoids in matrigel or collagen, resembling more closely the *in vivo* situation.

The secretome of adipose tissue is subject to many determinants. First, adipose tissue is dispersed all over the body but not all adipose tissue is equal [32]. For example, subcutaneous and visceral abdominal adipose tissue is biologically and genetically distinct [9, 11, 33]. Second, adipose tissue is composed of many different cell types like adipocytes, macrophages, endothelial cells, mesenchymal stem cells, etc. and each cell type contributes to the adipose tissue secretome [34, 35]. Third, certain disease states such as obesity and cancer alter the composition of adipose tissue and influence its secretion pattern [10, 11, 36]. In light of this, we used patient-derived explant culture of breast cancer-associated adipose tissue instead of plastic-adherent adipocyte precursors stimulated to adipocyte differentiation. Of note, this also explains the variability in adipokine levels between different patients as seen in Figure 1C. As we did not find a significant correlation between BMI and adipokine levels (results not shown), other micro or macro environmental factors must play a role in the adipose tissue secretion pattern.

Proteome analysis of CAAT-secreted factors revealed a large number of proteins involved in energy pathways and metabolism, which reflects the normal adipose tissue function and has been described by others [31, 37]. When investigating the affected biological pathways, more than 30% of adipose tissue proteins are linked with regulation of KLF7 transcription factor. Krüppel-like factors are drivers of cell proliferation and differentiation in different organ systems and many of them play a role in fat biology [38]. In human adipocytes, KLF7 expression is involved in adipogenesis, obesity and type 2 diabetes [39–41]. Both the biological process and transcription factor analysis confirmed the adipose nature of our CAAT proteome and this was further validated by ELISA (leptin and adiponectin), qPCR (adiponectin) and Western blot analysis (leptin, adiponectin and FABP4) on CM^CAAT^ derived from multiple patients. Physical separation of adipocytes from SVF revealed unique expression patterns with leptin and adiponectin dominantly expressed in adipocytes, and IL-6 and CCL22 dominantly expressed in SVF. Ultimately, we compared our 668 CAAT proteins with available data sets from human visceral abdominal adipose tissue [13] and isolated adipocytes from human subcutaneous adipose tissue [14, 15]. A substantial proportion of proteins was retraced in the three data sets (Supplementary Table 3). However, as expected, CAAT secretome has a unique secretion profile with multiple proteins not previously identified. This indicates that at least a part of our proteome will represent breast CAAT specific proteins, useful when studying paracrine effects of adipose tissue in breast cancer. Whether the identified proteins are breast adipose tissue specific or cancer specific will need more research using comparison of adipose tissue from non-cancer patients or from other sites in a breast cancer patient (e.g. breast adipose tissue versus visceral adipose tissue).

c-Jun and c-Fos are both proto-oncogenes and their homo- or heterodimers form the transcription factor AP-1, linked with cell proliferation, differentiation and survival [17, 42, 43]. A variety of growth factors, chemokines and cytokines stimulate the expression of c-Jun and c-Fos. Several of them like CCL22, four and a half LIM domains protein 1 (FHL1) and leukemia inhibiting factor (LIF) were detected in the breast CAAT proteome and have been linked with breast cancer proliferation and growth [44–47]. The transcriptional activity of c-Jun and c-Fos was stimulated by ERK, JNK and CREB signaling and their activation was detected by phospho kinase screening and Western blot in hormone-dependent MCF-7 and T47D breast cancer cells. However, we were not able to validate this pathway activation in hormone-independent MDA MB 231 cells where levels of p-ERK, p-c-Jun, p-c-Fos and p-CREB remained unchanged upon CM^CAAT^ treatment. This indicates that CAAT proteins are able to enhance MDA MB 231 cell proliferation via other pathways than c-Jun and c-Fos. Moreover, Hardy et al. describes the influence of free fatty acids (FFAs) like oleate and palmitate on Akt-activation and subsequent cell proliferation in MDA MB 231 cells demonstrating that not only proteins but also FFAs can exert a pro-proliferative effect [48]. This correlates with Western blot analysis showing activation of the Akt-pathway in CM^CAAT^ treated MDA MB 231 cells. As our research focused on breast cancer-associated adipose tissue proteins, further research will be necessary to elucidate the role of FFAs in breast cancer.

Palbociclib has recently been FDA approved for use in combination with letrozole for the treatment of postmenopausal women with estrogen receptor-positive, human epidermal growth factor (HER)2-negative advanced breast cancer as initial endocrine-based therapy for their metastatic disease. Palbociclib inhibits CDK4 and 6 to bind cyclin D1, keeping the tumor suppressor retinoblastoma protein (pRb) in its activated form and halting cell cycle progression [49, 50]. *In vitro* experimentation showed that palbociclib is most effective in estrogen receptor positive breast cancer cell lines [51]. This correlates with our observation that proliferation of hormone-dependent MCF-7 and T47D breast cancer cells was significantly inhibited upon palbociclib treatment, while this effect was not observed in hormone-independent MDA MB 231 breast cancer cells. On the contrary, we see a significant, dose dependent increase in palbociclib treated MDA MB 231 cells (non treated with CM^CAAT^). A possible explanation of these findings may be that CDK4/6 inhibition induces a compensatory mitogenic response as has been described in ER+/HER2- models [52]. In these models, the increased mitogenic signals due to palbociclib treatment were completely suppressed when the ER signaling pathway was simultaneously blocked by administration of an ER antagonist. This explains why the most positive clinical results are seen when palbociclib is combined with an endocrine therapy like the aromatase inhibitor letrozole in ER+/HER2- breast cancer patients. It is possible that in MDA MB 231 cells, which lack concurrent ER signaling, there is no mechanism present that could overrule the compensatory mitogenic signaling elicited by CDK4/6 inhibition. Further research will be necessary to test this hypothesis.

In contrast with the results in MDA MB 231 cells in control conditions, palbociclib was able to significantly inhibit CAAT-enhanced proliferation of all three breast cancer cell lines. This might signify that CAAT shifts the cell cycle control in MDA MB 231 cells from a non-pRb-regulated to a pRb-regulated way, sensitizing the breast cancer cells to CDK4/6 inhibitory treatment. This is supported by the fact that MDA MB 231 is a pRb-positive cell line, which is an absolute necessity for sensitivity to palbociclib treatment [53, 54]. Moreover, a MDA MB 231 orthotopic xenograft model showed a significant inhibition of the proliferation marker Ki67 by treatment with palbociclib [52]. These *in vivo* results correlate with our results of palbociclib-induced inhibition of CM^CAAT^-stimulated proliferation of MDA MB 231 cells given the fact that CM^CAAT^-treated MDA MB 231 cells resemble more closely the *in vivo* situation (MDA MB 231 cells injected into the mammary fat pad) than MDA MB 231 cells grown in absence of adipose tissue-derived factors (*in vitro* situation with control medium).

This the first report identifying patient-derived breast cancer-associated adipose tissue proteins representing potential therapeutic targets of paracrine signalling implicated in breast cancer growth.

## MATERIALS AND METHODS

### Cell lines

MCF-7, T47D and MDA MB 231 cell lines were obtained from American Type Culture Collection (ATCC, Manassas, VA). Cells were maintained in Dulbecco's minimal essential medium (DMEM) supplemented with 10% fetal calf serum, 100 U/mL penicillin, 100 μg/mL streptomycin (Invitrogen, Carlsbad, CA) and 2.5 μg/mL fungizone (Bristol-Meyers Squibb, Brussels, Belgium). Every month cell cultures were tested for Mycoplasma contamination using MycoAlert Plus kit (Lonza, Verviers, Belgium).

### Antibodies and reagents

Primary antibodies used for Western blot analysis and immunohistochemistry: mouse monoclonal anti-Ki67 (clone MIB-1) (Dako, Glostrup, Denmark), mouse monoclonal anti-cyclin A and anti-cyclin E (Invitrogen, Carlsbad, CA), mouse monoclonal anti-p27 and anti-p21 (Santa Cruz Biotechnology, Heidelberg, Germany), mouse monoclonal anti-tubulin (Sigma-Aldrich, St Louis, MO), rabbit monoclonal anti-phospho-p44/42 MAPK (Erk1/2), anti-p44/42 MAPK (Erk1/2), anti-phospho-Akt, anti-Akt, anti-phospho-c-Jun, anti-c-Jun, anti-phospho-c-Fos, anti-c-Fos, anti-phospho-CREB, anti-CREB (Cell Signaling Technology, Danvers, MA). Secondary antibodies coupled to horseradish peroxidase were obtained from Amersham Pharmacia Biotech (Diegem, Belgium). Palbociclib (PD-0332991) was purchased at Selleck Chemicals (Munich, Germany).

### Conditioned medium of CAAT (CM^CAAT^), isolation of tumor-associated adipocytes (TAA) and stromal vascular fraction (SVF)

CAAT was obtained from breast cancer patients undergoing mastectomy at Ghent University Hospital in accordance with local ethics committee and written informed consent was obtained from all subjects (Supplementary Table 4). CM^CAAT^ preparation and separation of adipose tissue in TAA and SVF were previously described [16]. In short, CAAT was washed, cut into pieces and placed in 6-well plates with DMEM/F12 + 0.5% BSA (= control medium) on a nutating mixer at 37°C and 5% CO_2_. After 24 h, CM^CAAT^ is harvested, centrifuged, filtered, aliquoted and stored at –80°C until experimentation. For separation of CAAT into TAA and SVF, CAAT is washed, thoroughly minced and digested with collagenase. After centrifugation, the upper layer containing TAA is separated from SVF and RNA of both fractions was extracted using RNeasy Plus Universal Kit (Qiagen, Venlo, The Netherlands). Supplementary Table 5 demonstrates the use of CM^CAAT^ throughout the article.

### Mass spectrometry

CM^CAAT^ samples were run on NuPAGE 4%-20% Bis-Tris gradient gels (Invitrogen) in SDS-containing denaturation buffer, stained with 0.5% Coomassie Brilliant Blue (Bio-Rad) in 40% methanol and 10% acetic acid for 20 minutes, and destained. Gel bands were processed and analyzed by LC-MS/MS as previously described [16, 55].

### Protein analysis

Samples for western blot were prepared, runned and immunostained as described in [55]. Human L507 array (Raybiotech, Norcross, GA) was used to detect relative levels of 507 proteins in CM^CAAT^. Human phospho-kinase antibody array (R&D systems) was used to detect relative phosphorylation levels of 44 kinases. Concentrations of human leptin, adiponectin, IL-6, C-C motif chemokine 22 (CCL22) and colony stimulating factor 1 (CSF-1) in CM^CAAT^ were measured with a Duoset or Quantikine ELISA Kit (R&D systems). Scanning densitometry was carried out with the Quantity One Program (Bio-Rad, Hercules, CA). Functional analysis of detected proteins was performed using FunRich version 2.1 [56]. Venn diagrams were created with BioVenn software (www.cmbi.ru.nl/cdd/biovenn/).

### Microarray analysis

Total RNA was isolated using the Nucleospin RNA II kit (Macherey-Nagel, Düren, Germany) including DNAse I treatment. Quality control was performed using Agilent 2100 Bioanalyser (Agilent Technologies). Total RNA (0.5 μg) was processed and analysed on Human GE Agilent 4 × 44K microarrays. Four biological samples were studied. Data can be found on GEO (GEO accession number GSE58574).

### Quantitative real-time PCR (RT-qPCR)

RNA was isolated using RNeasy Plus Universal Kit (Qiagen) including DNAse I treatment. cDNA synthesis and SYBR Green I RT-qPCR were carried out as described in [57]. Prime PCR assays for ADIPOQ (Adiponectin), IL-6, CCL22, CSF-1, ETS2, DUSP2, EMP1 and GEM were purchased at Bio-Rad. RNA quality index (RQI > 8) was assessed using Experion software (version 3.2, Bio-Rad).

### Proliferation test and MTT-assay

For proliferation tests, 20.000 cells were seeded in culture flasks with normal growth medium for 24 h. Every three days, cells were washed with serum-free medium and control medium or CM^CAAT^ with control medium (ratio 1:1) was added. On day 3, 6 and 9, cells were trypsinized and counted with Countess Automated Cell Counter (Life Technologies-Invitrogen, Gent, Belgium).

For MTT-assays, 2000 cells were seeded in a 96-well plate with normal growth medium for 24 h. Cells were washed with serum-free medium and control medium, CM^CAAT^ with control medium (ratio 1:1) and/or palbociclib were added and refreshed after 2 days. On day 5, 40 μl/well of 1 mg/ml MTT-solution was added and incubated for 2 h protected from light. Supernatant was removed and the formazan crystals were dissolved in 100 μl/well DMSO. Optical density was measured with a Spectramax Paradigm Multi-Mode Microplate Reader (Molecular Devices, Sunnyvale, CA).

### Statistics

Statistical analyses were performed using IBM SPSS Statistics 21.0 software (Chicago, IL). Continuous data were analysed with Mann-Whitney *U* test (mean ± standard deviation) or Student's *t-test* (difference of means and 95% confidence interval) where appropriate. Statistical tests were two-sided, *p*-values less than 0.05 were considered statistically significant.

## SUPPLEMENTARY MATERIALS FIGURES AND TABLES









## Abbreviations

AP-1: activator protein 1
ATCC: American Type Culture Collection
CAAT: cancer-associated adipose tissue
CCL22: C-C motif chemokine 22
CDK: cyclin-dependent kinase
CM: conditioned medium
CREB: cAMP responsive element-binding protein (CREB)
CSF-1: colony stimulating factor 1
FABP4: fatty acid binding protein 4
FHL1: four and a half LIM domains protein 1
HER2: human epidermal growth factor receptor 2
IL: interleukin
KLF: Krüppel-like factor
LIF: leukemia inhibiting factor
pRb: Retinoblastoma protein
SVF: stromal vascular fraction
TAA: tumor-associated adipocytes.
