# Supplementary material for: Secretome analysis of breast cancer-associated adipose tissue to identify paracrine regulators of breast cancer growth
Source: Oncotarget. 2017 May 3;8(29):47239–49. doi: 10.18632/oncotarget.17592 (PMC5564561; doi:10.18632/oncotarget.17592)
Supplement: Supplementary file 1 [file oncotarget-08-47239-s001.pdf]

Secretome analysis of breast cancer-associated adipose tissue to identify paracrine regulators of breast cancer growth

Supplementary Materials

Supplementary Table 1: Identification of CAAT secreted factors detected by LC-MS/MS from 2 breast cancer patients (CM37 and CM38) and antibody array from 4 breast cancer patients (CM13 and CM14, CM29 and CM32). See Supplementary_Table_1

Supplementary Table 2: Funrich analysis of the 668 detected proteins for biological processes and biological pathways. See Supplementary_Table_2

Supplementary Table 3: Table representing the common proteins between breast cancer-associated adipose tissue (BCAAT) and the proteomics data of Alvarez-Llamas G et al, Lehr S et al and Xie X et al. See Supplementary_Table_3

Supplementary Table 4: Clinical and pathological parameters of breast cancer patients undergoing mastectomy and isolation of adipose tissue for collecting the corresponding CM^{CAAT}

Patient Sample	Type	Grade	Max diam	ER	PR	Her2/Neu	Pos LN	BMI
			(mm)				(#)	
P13	Muc	II	7	+	+	-		24,6
	IDCA	I	6	+	+	-		
P14	IDCA	II	10	-	-	-	6	29,7
	IDCA	II	27	-	-	-		
P15	DCIS	III	45				0	19,6
P16	IDCA	III	2	+	-	+	7	31,2
P20	DCIS	III	65	+	-		0	26,6
P21	LOB	III	80	+	+	-	3	24,1
	LOB	II	11	+	+	-		
P23	LOB	II	60	+	+	-	9	19,1
P24	IDCA	III	40	-	-	-	0	23,5
P25	IDCA	III	17	+	+	-	4	23,0
	IDCA	II	7	+	+	-	0	
P26	DCIS	III						29,7
P27	IDCA	II	35	+	+	+	0	27,6
	LOB		5	+	+	+	0	
P29	IDCA	II	12	+	+	-	0	26,2
	IDCA	II	10	+	+	-		
P30	IDCA	II		+	+	+	5	41,0
P31	LOB	II	30	+	+	-	0	34,9
P32	IDCA	III	35	-	-	-	0	23,9
P33	IDCA	II	35	+	+	-	0	24,2
P35	LOB	III	50	+	+	-	5	25,1
P36	IDCA	I	28	+	+	-	1	25,8
P37	IDCA	I	19	+	+	-	0	27,7
	IDCA	I	10	+	+	-		
P38	IDCA	II	20	+	+	-	0	27,9
P40	LOB	III	64	+	+	+	13	35,7
P43	IDCA	III	72	+	+	+	29	23,9
P44	IDCA	III	34	+	+	-	6	24,8

MUC: mucoid carcinoma, IDCA: invasive ductal carcinoma, DCIS: ductal carcinoma in situ, LOB: lobular carcinoma, Max diam: maximal diameter, ER: estrogen receptor, PR: progesterone receptor, Pos LN: positive lymph nodes, BMI: body mass index.

Supplementary Table 5: Flow chart demonstrating the use of CM^{CAAT} throughout the manuscript

CM^{CAAT} of 18 patients

**Proteome/kinome**	**Transcriptomics**	**Functional tests**	
P13	P13	P15	P30
P14	P14	P16	P31
P29	P29	P20	P32
P32	P32	P21	P33
P37		P23	P35
P38		P24	P36
		P25	P38
		P26	P40
		P27	P43
		P29	P44
