# Supplementary material for: Secretome analysis of breast cancer-associated adipose tissue to identify paracrine regulators of breast cancer growth
Source: Oncotarget. 2017 May 3;8(29):47239–49. doi: 10.18632/oncotarget.17592 (PMC5564561; doi:10.18632/oncotarget.17592)
Supplement: Supplementary file 2 [file oncotarget-08-47239-s002.docx]

**Supplementary Table 1: Identification of CAAT secreted factors detected by LC-MS/MS from 2 breast cancer patients (CM37 and CM38) and antibody array from 4 breast cancer patients (CM13 and CM14, CM29 and CM32).**

|  |  |  |  | LC-MS/MS | | | | |  | Antibody Array | | |
| --- | --- | --- | --- | --- | --- | --- | --- | --- | --- | --- | --- | --- |
|  |  |  |  | CM37 | |  | CM38 | |  | CM13/14 |  | CM29/32 |
| **Accession Number** | **UniProt Number** | **Identified Proteins** | **MW (kDa)** | **Peptide Hits** | **Coverage** |  | **Peptide Hits** | **Coverage** |  |  |  |  |
| 1433B_HUMAN | P31946 | 14-3-3 protein beta/alpha | 28 | 7 | 54.1% |  | 4 | 39% |  |  |  |  |
| 1433E_HUMAN | P62258 | 14-3-3 protein epsilon | 29 | 16 | 54.9% |  | 12 | 63.9% |  |  |  |  |
| 1433G_HUMAN | P61981 | 14-3-3 protein gamma | 28 | 14 | 68.4% |  | 16 | 74.5% |  |  |  |  |
| 1433T_HUMAN | P27348 | 14-3-3 protein theta | 28 | 3 | 32.2% |  | 3 | 34.3% |  |  |  |  |
| 1433Z_HUMAN | P63104 | 14-3-3 protein zeta/delta | 28 | 18 | 65.7% |  | 18 | 71% |  |  |  |  |
| 6PGD_HUMAN | P52209 | 6-phosphogluconate dehydrogenase, decarboxylating | 53 | 2 | 6% |  | 11 | 31.1% |  |  |  |  |
| 6PGL_HUMAN | O95336 | 6-phosphogluconolactonase | 28 | 4 | 21.3% |  | 4 | 24.8% |  |  |  |  |
| A1AG1_HUMAN | P02763 | Alpha-1-acid glycoprotein 1 | 24 | 2 | 14.4% |  | 4 | 23.9% |  |  |  |  |
| A1AG2_HUMAN | P19652 | Alpha-1-acid glycoprotein 2 | 24 | 0 | 0% |  | 3 | 14.4% |  |  |  |  |
| A1AT_HUMAN | P01009 | Alpha-1-antitrypsin | 47 | 13 | 38.3% |  | 6 | 21.8% |  |  |  |  |
| A1BG_HUMAN | P04217 | Alpha-1B-glycoprotein | 54 | 10 | 37.6% |  | 5 | 17% |  |  |  |  |
| A2GL_HUMAN | P02750 | Leucine-rich alpha-2-glycoprotein | 38 | 0 | 0% |  | 2 | 10.4% |  |  |  |  |
| A2MG_HUMAN | P01023 | Alpha-2-macroglobulin | 163 | 48 | 43.1% |  | 59 | 50.8% |  |  |  |  |
| A6PVI2_HUMAN | A6PVI2 | Angiostatin | 19 |  |  |  |  |  |  | + |  | + |
| AATC_HUMAN | P17174 | Aspartate aminotransferase, cytoplasmic | 46 | 0 | 0% |  | 6 | 23.2% |  |  |  |  |
| ABHEB_HUMAN | Q96IU4 | Alpha/beta hydrolase domain-containing protein 14B | 22 | 5 | 27.1% |  | 7 | 46.2% |  |  |  |  |
| ACACB_HUMAN | O00763 | Acetyl-CoA carboxylase 2 | 277 | 5 | 4% |  | 5 | 4% |  |  |  |  |
| ACBP_HUMAN | P07108 | Acyl-CoA-binding protein | 10 | 2 | 34.5% |  | 3 | 52.9% |  |  |  |  |
| ACOC_HUMAN | P21399 | Cytoplasmic aconitate hydratase | 98 | 9 | 17.9% |  | 17 | 28.6% |  |  |  |  |
| ACOT1_HUMAN | Q86TX2 | Acyl-coenzyme A thioesterase 1 | 46 | 0 | 0% |  | 7 | 27.3% |  |  |  |  |
| ACTB_HUMAN | P60709 | Actin, cytoplasmic 1 | 42 | 16 | 62.1% |  | 23 | 67.2% |  |  |  |  |
| ACTN1_HUMAN | P12814 | Alpha-actinin-1 | 103 | 20 | 28.0% |  | 26 | 35.1% |  |  |  |  |
| ACTN4_HUMAN | O43707 | Alpha-actinin-4 | 105 | 7 | 26.1% |  | 9 | 29.9% |  |  |  |  |
| ACTS_HUMAN | P68133 | Actin, alpha skeletal muscle | 42 | 3 | 36.9% |  | 6 | 44.6% |  |  |  |  |
| ACTZ_HUMAN | P61163 | Alpha-centractin | 43 | 0 | 0% |  | 2 | 9.3% |  |  |  |  |
| ACY1_HUMAN | Q03154 | Aminoacylase-1 | 46 | 0 | 0% |  | 5 | 21.3% |  |  |  |  |
| ACYP2_HUMAN | P14621 | Acylphosphatase-2 | 11 | 2 | 22.2% |  | 1 | 9.1% |  |  |  |  |
| ADH1A_HUMAN | P07327 | Alcohol dehydrogenase 1A | 40 | 0 | 14.1% |  | 2 | 40.3% |  |  |  |  |
| ADH1B_HUMAN | P00325 | Alcohol dehydrogenase 1B | 40 | 9 | 24.5% |  | 29 | 67.5% |  |  |  |  |
| ADHX_HUMAN | P11766 | Alcohol dehydrogenase class-3 | 40 | 1 | 7.8% |  | 3 | 14.2% |  |  |  |  |
| ADIPO_HUMAN | Q15848 | Adiponectin | 26 | 4 | 30.7% |  | 3 | 21.7% |  | + |  | + |
| ADIRF_HUMAN | Q15847 | Adipogenesis regulatory factor | 8 | 0 | 0% |  | 2 | 48.7% |  |  |  |  |
| ADK_HUMAN | P55263 | Adenosine kinase | 41 | 0 | 0% |  | 2 | 13% |  |  |  |  |
| AFAM_HUMAN | P43652 | Afamin | 69 | 4 | 11.7% |  | 2 | 5.5% |  |  |  |  |
| AHNK_HUMAN | Q09666 | Neuroblast differentiation-associated protein AHNAK | 629 | 3 | 0.9% |  | 8 | 2.1% |  |  |  |  |
| AIFM2_HUMAN | Q9BRQ8 | Apoptosis-inducing factor 2 | 41 | 0 | 0% |  | 2 | 5.1% |  |  |  |  |
| AK1A1_HUMAN | P14550 | Alcohol dehydrogenase [NADP(+)] | 37 | 1 | 3.1% |  | 6 | 20.6% |  |  |  |  |
| AK1C1_HUMAN | Q04828 | Aldo-keto reductase family 1 member C1 | 37 | 4 | 17.3% |  | 13 | 52.6% |  |  |  |  |
| AK1C3_HUMAN | P42330 | Aldo-keto reductase family 1 member C3 | 37 | 3 | 22.3% |  | 7 | 43.3% |  |  |  |  |
| AKA12_HUMAN | Q02952 | A-kinase anchor protein 12 | 191 | 4 | 3.7% |  | 8 | 6.4% |  |  |  |  |
| AL1A1_HUMAN | P00352 | Retinal dehydrogenase 1 | 55 | 2 | 8.2% |  | 4 | 12.8% |  |  |  |  |
| AL1L1_HUMAN | O75891 | Cytosolic 10-formyltetrahydrofolate dehydrogenase | 99 | 6 | 9.8% |  | 11 | 22.2% |  |  |  |  |
| ALBU_HUMAN | P02768 | Serum albumin | 69 | 62 | 85.1% |  | 50 | 80.5% |  |  |  |  |
| ALDH2_HUMAN | P05091 | Aldehyde dehydrogenase, mitochondrial | 56 | 1 | 3.3% |  | 5 | 15.5% |  |  |  |  |
| ALDOA_HUMAN | P04075 | Fructose-bisphosphate aldolase A | 39 | 2 | 6.9% |  | 24 | 78.3% |  |  |  |  |
| ALDOC_HUMAN | P09972 | Fructose-bisphosphate aldolase C | 39 | 1 | 6.3% |  | 14 | 53% |  |  |  |  |
| ALDR_HUMAN | P15121 | Aldose reductase | 36 | 1 | 6.7% |  | 3 | 11.7% |  |  |  |  |
| AMBP_HUMAN | P02760 | Protein AMBP | 39 | 10 | 29.8% |  | 5 | 23% |  |  |  |  |
| AMPL_HUMAN | P28838 | Cytosol aminopeptidase | 56 | 2 | 5% |  | 0 | 0% |  |  |  |  |
| AN32B_HUMAN | Q92688 | Acidic leucine-rich nuclear phosphoprotein 32 family member B | 29 |  |  |  |  |  |  | + |  | + |
| ANGL1_HUMAN | O95841 | Angiopoietin-related protein 1 | 57 |  |  |  |  |  |  | + |  | + |
| ANGP1_HUMAN | Q15389 | Angiopoietin-1 | 58 |  |  |  |  |  |  |  |  | + |
| ANGP2_HUMAN | O15123 | Angiopoietin-2 | 57 |  |  |  |  |  |  | + |  | + |
| ANXA1_HUMAN | P04083 | Annexin A1 | 39 | 17 | 53.5% |  | 9 | 35.8% |  |  |  |  |
| ANXA2_HUMAN | P07355 | Annexin A2 | 39 | 28 | 67.3% |  | 11 | 41.6% |  |  |  |  |
| ANXA4_HUMAN | P09525 | Annexin A4 | 36 | 5 | 20.7% |  | 0 | 0% |  |  |  |  |
| ANXA5_HUMAN | P08758 | Annexin A5 | 36 | 10 | 43.1% |  | 7 | 27.5% |  |  |  |  |
| AOC3_HUMAN | Q16853 | Membrane primary amine oxidase | 85 | 4 | 8.1% |  | 9 | 19.5% |  |  |  |  |
| AP1B1_HUMAN | Q10567 | AP-1 complex subunit beta-1 | 105 | 2 | 3.5% |  | 2 | 4% |  |  |  |  |
| APOA1_HUMAN | P02647 | Apolipoprotein A-I | 31 | 23 | 70.8% |  | 22 | 60.7% |  |  |  |  |
| APOA2_HUMAN | P02652 | Apolipoprotein A-II | 11 | 5 | 59% |  | 6 | 69% |  |  |  |  |
| APOA4_HUMAN | P06727 | Apolipoprotein A-IV | 45 | 0 | 0% |  | 15 | 36.4% |  |  |  |  |
| APOB_HUMAN | P04114 | Apolipoprotein B-100 | 516 | 1 | 0.4% |  | 2 | 0.8% |  |  |  |  |
| APOC3_HUMAN | P02656 | Apolipoprotein C-III | 11 | 2 | 19.2% |  | 1 | 16.2% |  |  |  |  |
| APOD_HUMAN | P05090 | Apolipoprotein D | 21 | 12 | 42.3% |  | 9 | 33.3% |  |  |  |  |
| APOE_HUMAN | P02649 | Apolipoprotein E | 36 | 16 | 54.9% |  | 13 | 47.6% |  |  |  |  |
| APOH_HUMAN | P02749 | Beta-2-glycoprotein 1 | 38 | 2 | 11.6% |  | 1 | 4.4% |  |  |  |  |
| APT_HUMAN | P07741 | Adenine phosphoribosyltransferase | 20 | 2 | 8.3% |  | 2 | 8.3% |  |  |  |  |
| ARF3_HUMAN | P61204 | ADP-ribosylation factor 3 | 21 | 2 | 15.5% |  | 2 | 16.6% |  |  |  |  |
| ARK72_HUMAN | O43488 | Aflatoxin B1 aldehyde reductase member 2 | 40 | 0 | 0% |  | 3 | 12.5% |  |  |  |  |
| ARP3_HUMAN | P61158 | Actin-related protein 3 | 47 | 0 | 0% |  | 3 | 10.8% |  |  |  |  |
| ARPC2_HUMAN | O15144 | Actin-related protein 2/3 complex subunit 2 | 34 | 0 | 0% |  | 2 | 14% |  |  |  |  |
| ARPC3_HUMAN | O15145 | Actin-related protein 2/3 complex subunit 3 | 21 | 4 | 22.5% |  | 1 | 6.2% |  |  |  |  |
| ARPC4_HUMAN | P59998 | Actin-related protein 2/3 complex subunit 4 | 20 | 5 | 24.4% |  | 4 | 19% |  |  |  |  |
| ASAH1_HUMAN | Q13510 | Acid ceramidase | 45 | 3 | 12.2% |  | 4 | 14.2% |  |  |  |  |
| ASC_HUMAN | Q9ULZ3 | Apoptosis-associated speck-like protein containing a CARD | 22 | 2 | 12.8% |  | 2 | 12.8% |  |  |  |  |
| AT2L2_HUMAN | Q8IUZ5 | 5-phosphohydroxy-L-lysine phospho-lyase | 50 | 0 | 0% |  | 2 | 9.8% |  |  |  |  |
| ATPB_HUMAN | P06576 | ATP synthase subunit beta, mitochondrial | 57 | 0 | 0% |  | 2 | 5.1% |  |  |  |  |
| ATPD_HUMAN | P30049 | ATP synthase subunit delta, mitochondrial | 17 | 0 | 0% |  | 2 | 13.7% |  |  |  |  |
| ATRN_HUMAN | O75882 | Attractin | 159 | 5 | 4.4% |  | 0 | 0% |  |  |  |  |
| AVR2A_HUMAN | P27037 | Activin receptor type-2A | 58 |  |  |  |  |  |  | + |  | + |
| B2MG_HUMAN | P61769 | Beta-2-microglobulin | 14 | 3 | 35.3% |  | 2 | 26.9% |  |  |  |  |
| BAX_HUMAN | Q07812 | Apoptosis regulator BAX | 21 | 2 | 13.5% |  | 2 | 13% |  |  |  |  |
| BDNF_HUMAN | P23560 | Brain-derived neurotrophic factor | 28 |  |  |  |  |  |  | + |  | + |
| BLVRB_HUMAN | P30043 | Flavin reductase (NADPH) | 22 | 3 | 20.9% |  | 5 | 37.4% |  |  |  |  |
| BMP4_HUMAN | P12644 | Bone morphogenetic protein 4 | 47 |  |  |  |  |  |  |  |  | + |
| BMP8B_HUMAN | P34820 | Bone morphogenetic protein 8B | 45 |  |  |  |  |  |  |  |  | + |
| BMR1B_HUMAN | O00238 | Bone morphogenetic protein receptor type-1B | 57 |  |  |  |  |  |  | + |  | + |
| BPNT1_HUMAN | O95861 | 3'(2'),5'-bisphosphate nucleotidase 1 | 33 | 0 | 0% |  | 2 | 6.5% |  |  |  |  |
| C163A_HUMAN | Q86VB7 | Scavenger receptor cysteine-rich type 1 protein M130 | 125 | 0 | 0% |  | 11 | 12.2% |  |  |  |  |
| C1QA_HUMAN | P02745 | Complement C1q subcomponent subunit A | 26 | 2 | 9.4% |  | 2 | 9.4% |  |  |  |  |
| C1QB_HUMAN | P02746 | Complement C1q subcomponent subunit B | 27 | 4 | 22.5% |  | 4 | 23.3% |  |  |  |  |
| C1QC_HUMAN | P02747 | Complement C1q subcomponent subunit C | 26 | 4 | 18% |  | 3 | 15.9% |  |  |  |  |
| C1R_HUMAN | P00736 | Complement C1r subcomponent | 80 | 3 | 8.5% |  | 0 | 0% |  |  |  |  |
| C1S_HUMAN | P09871 | Complement C1s subcomponent | 77 | 4 | 6.7% |  | 3 | 4.4% |  |  |  |  |
| C1TC_HUMAN | P11586 | C-1-tetrahydrofolate synthase, cytoplasmic | 102 | 1 | 2.6% |  | 2 | 2.9% |  |  |  |  |
| CAH1_HUMAN | P00915 | Carbonic anhydrase 1 | 29 | 13 | 62.5% |  | 10 | 55.9% |  |  |  |  |
| CAH2_HUMAN | P00918 | Carbonic anhydrase 2 | 29 | 7 | 34.2% |  | 6 | 29.2% |  |  |  |  |
| CALB2_HUMAN | P22676 | Calretinin | 32 | 3 | 14.8% |  | 11 | 40.2% |  |  |  |  |
| CALM_HUMAN | P62158 | Calmodulin | 17 | 0 | 0% |  | 2 | 18.1% |  |  |  |  |
| CAND1_HUMAN | Q86VP6 | Cullin-associated NEDD8-dissociated protein 1 | 136 | 1 | 1.1% |  | 3 | 4.6% |  |  |  |  |
| CAP1_HUMAN | Q01518 | Adenylyl cyclase-associated protein 1 | 52 | 7 | 26.3% |  | 6 | 22.9% |  |  |  |  |
| CAPG_HUMAN | P40121 | Macrophage-capping protein | 38 | 0 | 0% |  | 3 | 10.6% |  |  |  |  |
| CAPZB_HUMAN | P47756 | F-actin-capping protein subunit beta | 31 | 5 | 32.1% |  | 9 | 42.2% |  |  |  |  |
| CATC_HUMAN | P53634 | Dipeptidyl peptidase 1 | 52 | 1 | 2.6% |  | 2 | 6.9% |  |  |  |  |
| CATD_HUMAN | P07339 | Cathepsin D | 45 | 1 | 3.2% |  | 4 | 14.3% |  |  |  |  |
| CATZ_HUMAN | Q9UBR2 | Cathepsin Z | 34 | 2 | 7.3% |  | 3 | 10.6% |  |  |  |  |
| CAV1_HUMAN | Q03135 | Caveolin-1 | 20 | 3 | 21.3% |  | 3 | 21.3% |  |  |  |  |
| CAZA1_HUMAN | P52907 | F-actin-capping protein subunit alpha-1 | 33 | 2 | 14.3% |  | 2 | 14% |  |  |  |  |
| CAZA2_HUMAN | P47755 | F-actin-capping protein subunit alpha-2 | 33 | 3 | 13.3% |  | 4 | 19.6% |  |  |  |  |
| CBR1_HUMAN | P16152 | Carbonyl reductase [NADPH] 1 | 30 | 1 | 10.8% |  | 7 | 40.1% |  |  |  |  |
| CCL2_HUMAN | P13500 | C-C motif chemokine 2 | 11 |  |  |  |  |  |  | + |  |  |
| CCL22_HUMAN | O00626 | C-C motif chemokine 22 | 11 |  |  |  |  |  |  | + |  | + |
| CCL27_HUMAN | Q9Y4X3 | C-C motif chemokine 27 | 13 |  |  |  |  |  |  | + |  | + |
| CCL3_HUMAN | P10147 | C-C motif chemokine 3 | 10 |  |  |  |  |  |  | + |  | + |
| CCL4_HUMAN | P13236 | C-C motif chemokine 4 | 10 |  |  |  |  |  |  | + |  | + |
| CCR2_HUMAN | P41597 | C-C chemokine receptor type 2 | 42 |  |  |  |  |  |  |  |  | + |
| CCR4_HUMAN | P51679 | C-C chemokine receptor type 4 | 41 |  |  |  |  |  |  |  |  | + |
| CCR7_HUMAN | P32248 | C-C chemokine receptor type 7 | 43 |  |  |  |  |  |  | + |  | + |
| CCR8_HUMAN | P51685 | C-C chemokine receptor type 8 | 41 |  |  |  |  |  |  | + |  | + |
| CCR9_HUMAN | P51686 | C-C chemokine receptor type 9 | 42 |  |  |  |  |  |  | + |  | + |
| CD248_HUMAN | Q9HCU0 | Endosialin | 81 | 0 | 0% |  | 2 | 3.4% |  |  |  |  |
| CD5L_HUMAN | O43866 | CD5 antigen-like | 38 | 0 | 0% |  | 5 | 24.5% |  |  |  |  |
| CD81_HUMAN | P60033 | CD81 antigen | 26 | 2 | 18.2% |  | 0 | 0% |  |  |  |  |
| CDN2C_HUMAN | P42773 | Cyclin-dependent kinase 4 inhibitor C | 18 | 1 | 4.8% |  | 2 | 11.3% |  |  |  |  |
| CERU_HUMAN | P00450 | Ceruloplasmin | 122 | 30 | 38.7% |  | 21 | 29.9% |  |  |  |  |
| CFAB_HUMAN | P00751 | Complement factor B | 86 | 12 | 19.0% |  | 14 | 25.4% |  |  |  |  |
| CFAD_HUMAN | P00746 | Complement factor D | 27 | 2 | 16.6% |  | 4 | 26.9% |  |  |  |  |
| CFAH_HUMAN | P08603 | Complement factor H | 139 | 0 | 0% |  | 32 | 34.5% |  |  |  |  |
| CFAI_HUMAN | P05156 | Complement factor I | 66 | 2 | 4% |  | 1 | 1.7% |  |  |  |  |
| CH10_HUMAN | P61604 | 10 kDa heat shock protein, mitochondrial | 11 | 3 | 37.3% |  | 5 | 62.7% |  |  |  |  |
| CISY_HUMAN | O75390 | Citrate synthase, mitochondrial | 52 | 0 | 0% |  | 4 | 10.5% |  |  |  |  |
| CK054_HUMAN | Q9H0W9 | Ester hydrolase C11orf54 | 35 | 2 | 9.2% |  | 2 | 9.2% |  |  |  |  |
| CLH1_HUMAN | Q00610 | Clathrin heavy chain 1 | 192 | 0 | 0% |  | 23 | 19.6% |  |  |  |  |
| CLIC1_HUMAN | O00299 | Chloride intracellular channel protein 1 | 27 | 3 | 24.1% |  | 5 | 32.4% |  |  |  |  |
| CLIC4_HUMAN | Q9Y696 | Chloride intracellular channel protein 4 | 29 | 2 | 7.91% |  | 1 | 4.7% |  |  |  |  |
| CLUS_HUMAN | P10909 | Clusterin | 52 | 5 | 17.1% |  | 5 | 16.7% |  |  |  |  |
| CNDP2_HUMAN | Q96KP4 | Cytosolic non-specific dipeptidase | 53 | 0 | 0% |  | 3 | 9.5% |  |  |  |  |
| CNTF_HUMAN | P26441 | Ciliary neurotrophic factor | 23 |  |  |  |  |  |  | + |  | + |
| CO1A1_HUMAN | P02452 | Collagen alpha-1(I) chain | 139 | 0 | 0% |  | 2 | 1.3% |  |  |  |  |
| CO3_HUMAN | P01024 | Complement C3 | 187 | 46 | 39.9% |  | 36 | 26.7% |  |  |  |  |
| CO4A_HUMAN | P0C0L4 | Complement C4-A | 193 | 34 | 28.9% |  | 20 | 18.5% |  |  |  |  |
| CO5_HUMAN | P01031 | Complement C5 | 188 | 3 | 3.8% |  | 2 | 1.1% |  |  |  |  |
| CO6A1_HUMAN | P12109 | Collagen alpha-1(VI) chain | 109 | 0 | 0% |  | 7 | 8.8% |  |  |  |  |
| CO6A3_HUMAN | P12111 | Collagen alpha-3(VI) chain | 344 | 38 | 17.5% |  | 12 | 5.7% |  |  |  |  |
| CO7_HUMAN | P10643 | Complement component C7 | 94 | 6 | 10.6% |  | 3 | 4.5% |  |  |  |  |
| CO8G_HUMAN | P07360 | Complement component C8 gamma chain | 22 | 9 | 50.0% |  | 2 | 12.9% |  |  |  |  |
| CO9_HUMAN | P02748 | Complement component C9 | 63 | 2 | 6.8% |  | 0 | 0% |  |  |  |  |
| COEA1_HUMAN | Q05707 | Collagen alpha-1(XIV) chain | 194 | 5 | 4.2% |  | 3 | 2.2% |  |  |  |  |
| COF1_HUMAN | P23528 | Cofilin-1 | 19 | 2 | 31.9% |  | 2 | 31.9% |  |  |  |  |
| COF2_HUMAN | Q9Y281 | Cofilin-2 | 19 | 3 | 27.1% |  | 3 | 27.1% |  |  |  |  |
| COFA1_HUMAN | P39059 | Collagen alpha-1(XV) chain | 142 | 3 | 3% |  | 1 | 1.2% |  |  |  |  |
| COIA1_HUMAN | P39060 | Collagen alpha-1(XVIII) chain | 178 | 8 | 6.9% |  | 7 | 6.9% |  | + |  |  |
| COR1C_HUMAN | Q9ULV4 | Coronin-1C | 53 | 0 | 0% |  | 2 | 6.1% |  |  |  |  |
| COTL1_HUMAN | Q14019 | Coactosin-like protein | 16 | 4 | 28.2% |  | 3 | 26.1% |  |  |  |  |
| CPN2_HUMAN | P22792 | Carboxypeptidase N subunit 2 | 61 | 3 | 11.7% |  | 1 | 5% |  |  |  |  |
| CPPED_HUMAN | Q9BRF8 | Serine/threonine-protein phosphatase CPPED1 | 36 | 3 | 15% |  | 3 | 8.9% |  |  |  |  |
| CRDL2_HUMAN | Q6WN34 | Chordin-like protein 2 | 47 |  |  |  |  |  |  | + |  | + |
| CRYAB_HUMAN | P02511 | Alpha-crystallin B chain | 20 | 7 | 50.9% |  | 12 | 66.3% |  |  |  |  |
| CS010_HUMAN | Q969H8 | UPF0556 protein C19orf10 | 19 | 0 | 0% |  | 4 | 27.2% |  |  |  |  |
| CSF1_HUMAN | P09603 | Macrophage colony-stimulating factor 1 | 60 |  |  |  |  |  |  | + |  | + |
| CSN4_HUMAN | Q9BT78 | COP9 signalosome complex subunit 4 | 46 | 0 | 0% |  | 2 | 7.9% |  |  |  |  |
| CSN7A_HUMAN | Q9UBW8 | COP9 signalosome complex subunit 7a | 30 | 1 | 5.8% |  | 3 | 16% |  |  |  |  |
| CSN8_HUMAN | Q99627 | COP9 signalosome complex subunit 8 | 23 | 2 | 12.9% |  | 1 | 6.7% |  |  |  |  |
| CSPG4_HUMAN | Q6UVK1 | Chondroitin sulfate proteoglycan 4 | 251 | 7 | 4.7% |  | 18 | 11.3% |  |  |  |  |
| CUTA_HUMAN | O60888 | Protein CutA | 19 | 1 | 7.8% |  | 2 | 17.9% |  |  |  |  |
| CX6B1_HUMAN | P14854 | Cytochrome c oxidase subunit 6B1 | 10 | 1 | 14% |  | 2 | 44.2% |  |  |  |  |
| CXCL2_HUMAN | P19875 | C-X-C motif chemokine 2 | 11 |  |  |  |  |  |  | + |  | + |
| CXCL9_HUMAN | Q07325 | C-X-C motif chemokine 9 | 14 |  |  |  |  |  |  | + |  |  |
| CXCR1_HUMAN | P25024 | C-X-C chemokine receptor type 1 | 40 |  |  |  |  |  |  | + |  | + |
| CXCR2_HUMAN | P25025 | C-X-C chemokine receptor type 2 | 41 |  |  |  |  |  |  | + |  | + |
| CXCR4_HUMAN | P61073 | C-X-C chemokine receptor type 4 | 40 |  |  |  |  |  |  |  |  | + |
| CXCR5_HUMAN | P32302 | C-X-C chemokine receptor type 5 | 42 |  |  |  |  |  |  |  |  | + |
| CXCR6_HUMAN | O00574 | C-X-C chemokine receptor type 6 | 39 |  |  |  |  |  |  |  |  | + |
| CXL11_HUMAN | O14625 | C-X-C motif chemokine 11 | 10 |  |  |  |  |  |  | + |  | + |
| CXL14_HUMAN | O95715 | C-X-C motif chemokine 14 | 13 |  |  |  |  |  |  | + |  | + |
| CYB5_HUMAN | P00167 | Cytochrome b5 | 15 | 4 | 47% |  | 4 | 40.3% |  |  |  |  |
| CYC_HUMAN | P99999 | Cytochrome c | 12 | 1 | 13.3% |  | 3 | 24.8% |  |  |  |  |
| CYTB_HUMAN | P04080 | Cystatin-B | 11 | 2 | 24.5% |  | 1 | 12.2% |  |  |  |  |
| DBLOH_HUMAN | Q9NR28 | Diablo homolog, mitochondrial | 27 | 2 | 10% |  | 0 | 0% |  |  |  |  |
| DCXR_HUMAN | Q7Z4W1 | L-xylulose reductase | 26 | 5 | 32% |  | 6 | 38.9% |  |  |  |  |
| DDAH2_HUMAN | O95865 | N(G),N(G)-dimethylarginine dimethylaminohydrolase 2 | 30 | 7 | 39.3% |  | 10 | 54.4% |  |  |  |  |
| DECR_HUMAN | Q16698 | 2,4-dienoyl-CoA reductase, mitochondrial | 36 | 0 | 0% |  | 2 | 7.8% |  |  |  |  |
| DERM_HUMAN | Q07507 | Dermatopontin | 24 | 3 | 21.9% |  | 1 | 8.5% |  |  |  |  |
| DHPR_HUMAN | P09417 | Dihydropteridine reductase | 26 | 0 | 0% |  | 2 | 10.2% |  |  |  |  |
| DKK4_HUMAN | Q9UBT3 | Dickkopf-related protein 4 | 25 |  |  |  |  |  |  |  |  | + |
| DLRB1_HUMAN | Q9NP97 | Dynein light chain roadblock-type 1 | 11 | 1 | 16.7% |  | 2 | 29.2% |  |  |  |  |
| DOPD_HUMAN | P30046 | D-dopachrome decarboxylase | 13 | 4 | 39% |  | 3 | 33.1% |  |  |  |  |
| DUS3_HUMAN | P51452 | Dual specificity protein phosphatase 3 | 20 | 3 | 22.7% |  | 2 | 15.1% |  |  |  |  |
| DYHC1_HUMAN | Q14204 | Cytoplasmic dynein 1 heavy chain 1 | 532 | 0 | 0% |  | 2 | 0.8% |  |  |  |  |
| DYL2_HUMAN | Q96FJ2 | Dynein light chain 2, cytoplasmic | 10 | 2 | 22.5% |  | 2 | 25.8% |  |  |  |  |
| ECH1_HUMAN | Q13011 | Delta(3,5)-Delta(2,4)-dienoyl-CoA isomerase, mitochondrial | 36 | 6 | 25.6% |  | 8 | 34.8% |  |  |  |  |
| ECHD1_HUMAN | Q9NTX5 | Ethylmalonyl-CoA decarboxylase | 34 | 8 | 45.0% |  | 14 | 51.8% |  |  |  |  |
| ECHM_HUMAN | P30084 | Enoyl-CoA hydratase, mitochondrial | 31 | 0 | 0% |  | 2 | 12.1% |  |  |  |  |
| ECI1_HUMAN | P42126 | Enoyl-CoA delta isomerase 1, mitochondrial | 33 | 0 | 0% |  | 2 | 10.6% |  |  |  |  |
| EEA1_HUMAN | Q15075 | Early endosome antigen 1 | 162 | 0 | 0% |  | 6 | 6.4% |  |  |  |  |
| EF1A3_HUMAN | Q5VTE0 | Putative elongation factor 1-alpha-like 3 | 50 | 1 | 2.4% |  | 6 | 20.3% |  |  |  |  |
| EF1D_HUMAN | P29692 | Elongation factor 1-delta | 31 | 3 | 17.1% |  | 3 | 18.5% |  |  |  |  |
| EF1G_HUMAN | P26641 | Elongation factor 1-gamma | 50 | 2 | 5.5% |  | 3 | 8.5% |  |  |  |  |
| EF2_HUMAN | P13639 | Elongation factor 2 | 95 | 0 | 0% |  | 8 | 15.4% |  |  |  |  |
| EHD2_HUMAN | Q9NZN4 | EH domain-containing protein 2 | 61 | 12 | 33.9% |  | 12 | 30.9% |  |  |  |  |
| EIF3B_HUMAN | P55884 | Eukaryotic translation initiation factor 3 subunit B | 92 | 1 | 1.7% |  | 2 | 3.8% |  |  |  |  |
| EIF3H_HUMAN | O15372 | Eukaryotic translation initiation factor 3 subunit H | 40 | 1 | 5.4% |  | 2 | 5.7% |  |  |  |  |
| ELTD1_HUMAN | Q9HBW9 | EGF, latrophilin and seven transmembrane domain-containing protein 1 | 78 |  |  |  |  |  |  | + |  | + |
| ENOA_HUMAN | P06733 | Alpha-enolase | 47 | 8 | 32.9% |  | 20 | 63.8% |  |  |  |  |
| ENOB_HUMAN | P13929 | Beta-enolase | 47 | 1 | 9.5% |  | 2 | 22.8% |  |  |  |  |
| ENPL_HUMAN | P14625 | Endoplasmin | 92 | 0 | 0% |  | 5 | 9.7% |  |  |  |  |
| EPO_HUMAN | P01588 | Erythropoietin | 21 |  |  |  |  |  |  | + |  | + |
| EPS15_HUMAN | P42566 | Epidermal growth factor receptor substrate 15 | 99 | 0 | 0% |  | 2 | 4.8% |  |  |  |  |
| ERAP1_HUMAN | Q9NZ08 | Endoplasmic reticulum aminopeptidase 1 | 107 | 1 | 1.4% |  | 3 | 4.4% |  |  |  |  |
| ERBB4_HUMAN | Q15303 | Receptor tyrosine-protein kinase erbB-4 | 147 |  |  |  |  |  |  |  |  | + |
| ERP29_HUMAN | P30040 | Endoplasmic reticulum resident protein 29 | 29 | 0 | 0% |  | 2 | 9.6% |  |  |  |  |
| ES1_HUMAN | P30042 | ES1 protein homolog, mitochondrial | 28 | 1 | 3.7% |  | 3 | 16% |  |  |  |  |
| EST1_HUMAN | P23141 | Liver carboxylesterase 1 | 63 | 0 | 0% |  | 6 | 16.2% |  |  |  |  |
| ESYT1_HUMAN | Q9BSJ8 | Extended synaptotagmin-1 | 123 | 5 | 7.8% |  | 3 | 3.4% |  |  |  |  |
| F13A_HUMAN | P00488 | Coagulation factor XIII A chain | 83 | 15 | 30.6% |  | 20 | 34.6% |  |  |  |  |
| FA49B_HUMAN | Q9NUQ9 | Protein FAM49B | 37 | 6 | 24.7% |  | 8 | 32.7% |  |  |  |  |
| FAAA_HUMAN | P16930 | Fumarylacetoacetase | 46 | 0 | 0% |  | 16 | 54.7% |  |  |  |  |
| FABP4_HUMAN | P15090 | Fatty acid-binding protein, adipocyte | 15 | 14 | 78.0% |  | 11 | 75.0% |  |  |  |  |
| FABP5_HUMAN | Q01469 | Fatty acid-binding protein, epidermal | 15 | 7 | 65.2% |  | 10 | 72.6% |  |  |  |  |
| FAS_HUMAN | P49327 | Fatty acid synthase | 273 | 79 | 45.8% |  | 98 | 53.3% |  |  |  |  |
| FBLN1_HUMAN | P23142 | Fibulin-1 | 77 | 9 | 17.5% |  | 2 | 3% |  |  |  |  |
| FBLN2_HUMAN | P98095 | Fibulin-2 | 127 | 3 | 4.6% |  | 7 | 8.5% |  |  |  |  |
| FERM2_HUMAN | Q96AC1 | Fermitin family homolog 2 | 78 | 6 | 12.6% |  | 10 | 20.3% |  |  |  |  |
| FETA_HUMAN | P02771 | Alpha-fetoprotein | 69 | 2 | 6.4% |  | 2 | 3.9% |  |  |  |  |
| FETUA_HUMAN | P02765 | Alpha-2-HS-glycoprotein | 39 | 5 | 17.2% |  | 4 | 18% |  |  |  |  |
| FGF11_HUMAN | Q92914 | Fibroblast growth factor 11 | 25 |  |  |  |  |  |  |  |  | + |
| FGF16_HUMAN | O43320 | Fibroblast growth factor 16 | 24 |  |  |  |  |  |  |  |  | + |
| FGF2_HUMAN | P09038 | Fibroblast growth factor 2 | 31 |  |  |  |  |  |  |  |  | + |
| FGF20_HUMAN | Q9NP95 | Fibroblast growth factor 20 | 23 |  |  |  |  |  |  | + |  | + |
| FGF23_HUMAN | Q9GZV9 | Fibroblast growth factor 23 | 28 |  |  |  |  |  |  | + |  | + |
| FGF9_HUMAN | P31371 | Fibroblast growth factor 9 | 23 |  |  |  |  |  |  | + |  | + |
| FGFR4_HUMAN | P22455 | Fibroblast growth factor receptor 4 | 88 |  |  |  |  |  |  | + |  | + |
| FHL1_HUMAN | Q13642 | Four and a half LIM domains protein 1 | 36 | 6 | 25.1% |  | 7 | 22% |  |  |  |  |
| FIBA_HUMAN | P02671 | Fibrinogen alpha chain | 95 | 18 | 20.1% |  | 8 | 9.9% |  |  |  |  |
| FIBB_HUMAN | P02675 | Fibrinogen beta chain | 56 | 5 | 19.6% |  | 17 | 42% |  |  |  |  |
| FIBG_HUMAN | P02679 | Fibrinogen gamma chain | 52 | 15 | 48.1% |  | 14 | 41.7% |  |  |  |  |
| FINC_HUMAN | P02751 | Fibronectin | 263 | 28 | 19.5% |  | 17 | 11.2% |  |  |  |  |
| FIS1_HUMAN | Q9Y3D6 | Mitochondrial fission 1 protein | 17 | 2 | 15.8% |  | 2 | 15.8% |  |  |  |  |
| FKB1A_HUMAN | P62942 | Peptidyl-prolyl cis-trans isomerase FKBP1A | 12 | 2 | 28.7% |  | 1 | 13% |  |  |  |  |
| FLNA_HUMAN | P21333 | Filamin-A | 281 | 23 | 15.3% |  | 40 | 24.2% |  |  |  |  |
| FLNB_HUMAN | O75369 | Filamin-B | 278 | 4 | 2.5% |  | 3 | 1.7% |  |  |  |  |
| FRIH_HUMAN | P02794 | Ferritin heavy chain | 21 | 4 | 33.3% |  | 6 | 59% |  |  |  |  |
| FRIL_HUMAN | P02792 | Ferritin light chain | 20 | 7 | 43.4% |  | 9 | 48.6% |  |  |  |  |
| FST_HUMAN | P19883 | Follistatin | 38 |  |  |  |  |  |  | + |  | + |
| FSTL1_HUMAN | Q12841 | Follistatin-related protein 1 | 35 |  |  |  |  |  |  | + |  | + |
| FUMH_HUMAN | P07954 | Fumarate hydratase, mitochondrial | 55 | 0 | 0% |  | 5 | 17.8% |  |  |  |  |
| G3P_HUMAN | P04406 | Glyceraldehyde-3-phosphate dehydrogenase | 36 | 8 | 37.0% |  | 9 | 40.9% |  |  |  |  |
| G6PI_HUMAN | P06744 | Glucose-6-phosphate isomerase | 63 | 4 | 10.2% |  | 5 | 12.4% |  |  |  |  |
| GAPR1_HUMAN | Q9H4G4 | Golgi-associated plant pathogenesis-related protein 1 | 17 | 2 | 16.9% |  | 2 | 16.9% |  |  |  |  |
| GBB1_HUMAN | P62873 | Guanine nucleotide-binding protein G(I)/G(S)/G(T) subunit beta-1 | 37 | 8 | 24.7% |  | 6 | 19.7% |  |  |  |  |
| GBB2_HUMAN | P62879 | Guanine nucleotide-binding protein G(I)/G(S)/G(T) subunit beta-2 | 37 | 3 | 24.4% |  | 2 | 15.6% |  |  |  |  |
| GBLP_HUMAN | P63244 | Guanine nucleotide-binding protein subunit beta-2-like 1 | 35 | 1 | 5.1% |  | 3 | 12.6% |  |  |  |  |
| GDF15_HUMAN | Q99988 | Growth/differentiation factor 15 | 34 |  |  |  |  |  |  | + |  | + |
| GDIB_HUMAN | P50395 | Rab GDP dissociation inhibitor beta | 51 | 1 | 4.3% |  | 6 | 19.8% |  |  |  |  |
| GDIR1_HUMAN | P52565 | Rho GDP-dissociation inhibitor 1 | 23 | 5 | 30.4% |  | 7 | 39.2% |  |  |  |  |
| GDIR2_HUMAN | P52566 | Rho GDP-dissociation inhibitor 2 | 23 | 3 | 22.9% |  | 2 | 16.9% |  |  |  |  |
| GELS_HUMAN | P06396 | Gelsolin | 86 | 15 | 28.3% |  | 16 | 29.8% |  |  |  |  |
| GFRA2_HUMAN | O00451 | GDNF family receptor alpha-2 | 52 |  |  |  |  |  |  |  |  | + |
| GFRP_HUMAN | P30047 | GTP cyclohydrolase 1 feedback regulatory protein | 10 | 3 | 40.5% |  | 1 | 9.5% |  |  |  |  |
| GGCT_HUMAN | O75223 | Gamma-glutamylcyclotransferase | 21 | 2 | 13.8% |  | 1 | 6.4% |  |  |  |  |
| GLNA_HUMAN | P15104 | Glutamine synthetase | 42 | 0 | 0% |  | 5 | 19% |  |  |  |  |
| GLOD4_HUMAN | Q9HC38 | Glyoxalase domain-containing protein 4 | 35 | 3 | 11.5% |  | 4 | 16.6% |  |  |  |  |
| GLRX1_HUMAN | P35754 | Glutaredoxin-1 | 12 | 3 | 23.6% |  | 5 | 55.7% |  |  |  |  |
| GLU2B_HUMAN | P14314 | Glucosidase 2 subunit beta | 59 | 0 | 0% |  | 5 | 9.7% |  |  |  |  |
| GLUC_HUMAN | P01275 | Glucagon | 21 |  |  |  |  |  |  | + |  | + |
| GNAI1_HUMAN | P63096 | Guanine nucleotide-binding protein G(i) subunit alpha-1 | 40 | 0 | 0% |  | 2 | 10.5% |  |  |  |  |
| GNAI2_HUMAN | P04899 | Guanine nucleotide-binding protein G(i) subunit alpha-2 | 40 | 0 | 0% |  | 2 | 7.3% |  |  |  |  |
| GNPI1_HUMAN | P46926 | Glucosamine-6-phosphate isomerase 1 | 33 | 3 | 24.6% |  | 1 | 5.9% |  |  |  |  |
| GPC5_HUMAN | P78333 | Glypican-5 | 64 |  |  |  |  |  |  | + |  | + |
| GPD1L_HUMAN | Q8N335 | Glycerol-3-phosphate dehydrogenase 1-like protein | 38 | 4 | 14.8% |  | 3 | 8.8% |  |  |  |  |
| GPDA_HUMAN | P21695 | Glycerol-3-phosphate dehydrogenase [NAD(+)], cytoplasmic | 38 | 29 | 95.1% |  | 27 | 90.3% |  |  |  |  |
| GPNMB_HUMAN | Q14956 | Transmembrane glycoprotein NMB | 64 |  |  |  |  |  |  | + |  | + |
| GPX3_HUMAN | P22352 | Glutathione peroxidase 3 | 26 | 4 | 25.7% |  | 2 | 12.8% |  |  |  |  |
| GRB2_HUMAN | P62993 | Growth factor receptor-bound protein 2 | 25 | 2 | 10.1% |  | 1 | 4.6% |  |  |  |  |
| GRHPR_HUMAN | Q9UBQ7 | Glyoxylate reductase/hydroxypyruvate reductase | 36 | 0 | 0% |  | 3 | 21.6% |  |  |  |  |
| GROA_HUMAN | P09341 | Growth-regulated alpha protein | 11 |  |  |  |  |  |  | + |  |  |
| GSTM3_HUMAN | P21266 | Glutathione S-transferase Mu 3 | 27 | 4 | 24% |  | 3 | 16.4% |  |  |  |  |
| GSTO1_HUMAN | P78417 | Glutathione S-transferase omega-1 | 28 | 3 | 12% |  | 5 | 21.2% |  |  |  |  |
| GSTP1_HUMAN | P09211 | Glutathione S-transferase P | 23 | 6 | 51.9% |  | 5 | 43.8% |  |  |  |  |
| GSTT1_HUMAN | P30711 | Glutathione S-transferase theta-1 | 27 | 0 | 0% |  | 2 | 12.5% |  |  |  |  |
| GTR2_HUMAN | P11168 | Solute carrier family 2, facilitated glucose transporter member 2 | 57 |  |  |  |  |  |  | + |  | + |
| GTR5_HUMAN | P22732 | Solute carrier family 2, facilitated glucose transporter member 5 | 55 |  |  |  |  |  |  | + |  | + |
| H2A2C_HUMAN | Q16777 | Histone H2A type 2-C | 14 | 4 | 47.3% |  | 3 | 30.2% |  |  |  |  |
| H2B1D_HUMAN | P58876 | Histone H2B type 1-D | 14 | 7 | 53.2% |  | 4 | 36.5% |  |  |  |  |
| H33_HUMAN | P84243 | Histone H3.3 | 15 | 2 | 31.6% |  | 2 | 31.6% |  |  |  |  |
| H4_HUMAN | P62805 | Histone H4 | 11 | 10 | 57.3% |  | 8 | 56.3% |  |  |  |  |
| HBA_HUMAN | P69905 | Hemoglobin subunit alpha | 15 | 7 | 65.5% |  | 6 | 64.8% |  |  |  |  |
| HBB_HUMAN | P68871 | Hemoglobin subunit beta | 16 | 16 | 95.2% |  | 16 | 95.2% |  |  |  |  |
| HBD_HUMAN | P02042 | Hemoglobin subunit delta | 16 | 6 | 72.1% |  | 6 | 80.3% |  |  |  |  |
| HBEGF_HUMAN | Q99075 | Proheparin-binding EGF-like growth factor | 23 |  |  |  |  |  |  | + |  | + |
| HBG1_HUMAN | P69891 | Hemoglobin subunit gamma-1 | 16 | 3 | 40.1% |  | 1 | 15.6% |  |  |  |  |
| HCDH_HUMAN | Q16836 | Hydroxyacyl-coenzyme A dehydrogenase, mitochondrial | 34 | 4 | 22.3% |  | 10 | 53.2% |  |  |  |  |
| HDHD2_HUMAN | Q9H0R4 | Haloacid dehalogenase-like hydrolase domain-containing protein 2 | 29 | 1 | 8.1% |  | 2 | 11.2% |  |  |  |  |
| HEBP1_HUMAN | Q9NRV9 | Heme-binding protein 1 | 21 | 3 | 22.2% |  | 4 | 27.5% |  |  |  |  |
| HEBP2_HUMAN | Q9Y5Z4 | Heme-binding protein 2 | 23 | 6 | 32.7% |  | 6 | 32.7% |  |  |  |  |
| HEM2_HUMAN | P13716 | Delta-aminolevulinic acid dehydratase | 36 | 5 | 12.4% |  | 13 | 37% |  |  |  |  |
| HEMO_HUMAN | P02790 | Hemopexin | 52 | 6 | 23.4% |  | 8 | 22.5% |  |  |  |  |
| HIBCH_HUMAN | Q6NVY1 | 3-hydroxyisobutyryl-CoA hydrolase, mitochondrial | 43 | 0 | 0% |  | 2 | 5.2% |  |  |  |  |
| HINT2_HUMAN | Q9BX68 | Histidine triad nucleotide-binding protein 2, mitochondrial | 17 | 1 | 9.8% |  | 2 | 20.2% |  |  |  |  |
| HNMT_HUMAN | P50135 | Histamine N-methyltransferase | 33 | 2 | 11.0% |  | 8 | 33.9% |  |  |  |  |
| HNRPC_HUMAN | P07910 | Heterogeneous nuclear ribonucleoproteins C1/C2 | 34 | 5 | 19.9% |  | 5 | 15.4% |  |  |  |  |
| HPT_HUMAN | P00738 | Haptoglobin | 45 | 10 | 19.5% |  | 19 | 55.2% |  |  |  |  |
| HPTR_HUMAN | P00739 | Haptoglobin-related protein | 39 | 2 | 15.5% |  | 2 | 31% |  |  |  |  |
| HRG_HUMAN | P04196 | Histidine-rich glycoprotein | 60 | 3 | 8% |  | 2 | 5.7% |  |  |  |  |
| HS90A_HUMAN | P07900 | Heat shock protein HSP 90-alpha | 85 | 8 | 13.9% |  | 17 | 29.8% |  |  |  |  |
| HS90B_HUMAN | P08238 | Heat shock protein HSP 90-beta | 83 | 0 | 7% |  | 5 | 19.3% |  |  |  |  |
| HSP71_HUMAN | P08107 | Heat shock 70 kDa protein 1A/1B | 70 | 3 | 10% |  | 3 | 10% |  |  |  |  |
| HSP7C_HUMAN | P11142 | Heat shock cognate 71 kDa protein | 71 | 2 | 2.6% |  | 2 | 2.6% |  |  |  |  |
| HSPB1_HUMAN | P04792 | Heat shock protein beta-1 | 23 | 4 | 36.1% |  | 8 | 55.6% |  |  |  |  |
| HSPB6_HUMAN | O14558 | Heat shock protein beta-6 | 17 | 5 | 47.5% |  | 7 | 78.8% |  |  |  |  |
| HV305_HUMAN | P01766 | Ig heavy chain V-III region BRO | 13 | 2 | 25.0% |  | 2 | 25% |  |  |  |  |
| HV310_HUMAN | P01771 | Ig heavy chain V-III region HIL | 14 | 1 | 13.2% |  | 2 | 22.3% |  |  |  |  |
| HYEP_HUMAN | P07099 | Epoxide hydrolase 1 | 53 | 0 | 0% |  | 6 | 15.6% |  |  |  |  |
| HYI_HUMAN | Q5T013 | Putative hydroxypyruvate isomerase | 30 | 1 | 4% |  | 3 | 19.9% |  |  |  |  |
| I15RA_HUMAN | Q13261 | Interleukin-15 receptor subunit alpha | 28 |  |  |  |  |  |  |  |  | + |
| I17RD_HUMAN | Q8NFM7 | Interleukin-17 receptor D | 82 |  |  |  |  |  |  | + |  |  |
| I20RB_HUMAN | Q6UXL0 | Interleukin-20 receptor subunit beta | 35 |  |  |  |  |  |  |  |  | + |
| I36RA_HUMAN | Q9UBH0 | Interleukin-36 receptor antagonist protein | 17 |  |  |  |  |  |  |  |  | + |
| IBP3_HUMAN | P17936 | Insulin-like growth factor-binding protein 3 | 32 |  |  |  |  |  |  |  |  | + |
| IBP6_HUMAN | P24592 | Insulin-like growth factor-binding protein 6 | 25 |  |  |  |  |  |  |  |  | + |
| IBP7_HUMAN | Q16270 | Insulin-like growth factor-binding protein 7 | 29 | 6 | 35.1% |  | 4 | 23.4% |  | + |  | + |
| IC1_HUMAN | P05155 | Plasma protease C1 inhibitor | 55 | 17 | 35.2% |  | 12 | 24.8% |  |  |  |  |
| ICAM5_HUMAN | Q9UMF0 | Intercellular adhesion molecule 5 | 97 |  |  |  |  |  |  | + |  | + |
| IDHC_HUMAN | O75874 | Isocitrate dehydrogenase [NADP] cytoplasmic | 47 | 3 | 9.2% |  | 15 | 41.1% |  |  |  |  |
| IF4B_HUMAN | P23588 | Eukaryotic translation initiation factor 4B | 69 | 1 | 2.8% |  | 2 | 5.6% |  |  |  |  |
| IFNL1_HUMAN | Q8IU54 | Interferon lambda-1 | 22 |  |  |  |  |  |  | + |  | + |
| IGF2_HUMAN | P01344 | Insulin-like growth factor II | 20 |  |  |  |  |  |  | + |  | + |
| IGHA1_HUMAN | P01876 | Ig alpha-1 chain C region | 38 | 13 | 47.3% |  | 7 | 29.5% |  |  |  |  |
| IGHG1_HUMAN | P01857 | Ig gamma-1 chain C region | 36 | 15 | 60.3% |  | 17 | 61.8% |  |  |  |  |
| IGHG2_HUMAN | P01859 | Ig gamma-2 chain C region | 36 | 7 | 55.8% |  | 8 | 54.9% |  |  |  |  |
| IGHG3_HUMAN | P01860 | Ig gamma-3 chain C region | 41 | 7 | 40.8% |  | 7 | 40.8% |  |  |  |  |
| IGHG4_HUMAN | P01861 | Ig gamma-4 chain C region | 36 | 7 | 63.6% |  | 4 | 54.1% |  |  |  |  |
| IGHM_HUMAN | P01871 | Ig mu chain C region | 49 | 11 | 34.3% |  | 15 | 40% |  |  |  |  |
| IGJ_HUMAN | P01591 | Immunoglobulin J chain | 18 | 4 | 24.5% |  | 4 | 24.5% |  |  |  |  |
| IGKC_HUMAN | P01834 | Ig kappa chain C region | 12 | 10 | 89.6% |  | 9 | 85.8% |  |  |  |  |
| IGLL5_HUMAN | B9A064 | Immunoglobulin lambda-like polypeptide 5 | 23 | 11 | 50.0% |  | 11 | 50.0% |  |  |  |  |
| IL17B_HUMAN | Q9UHF5 | Interleukin-17B | 20 |  |  |  |  |  |  |  |  | + |
| IL17C_HUMAN | Q9P0M4 | Interleukin-17C | 22 |  |  |  |  |  |  | + |  | + |
| IL17D_HUMAN | Q8TAD2 | Interleukin-17D | 22 |  |  |  |  |  |  |  |  | + |
| IL17F_HUMAN | Q96PD4 | Interleukin-17F | 18 |  |  |  |  |  |  | + |  | + |
| IL19_HUMAN | Q9UHD0 | Interleukin-19 | 20 |  |  |  |  |  |  |  |  | + |
| IL1R1_HUMAN | P14778 | Interleukin-1 receptor type 1 | 65 |  |  |  |  |  |  | + |  | + |
| IL22_HUMAN | Q9GZX6 | Interleukin-22 | 20 |  |  |  |  |  |  | + |  | + |
| IL26_HUMAN | Q9NPH9 | Interleukin-26 | 20 |  |  |  |  |  |  |  |  | + |
| IL36A_HUMAN | Q9UHA7 | Interleukin-36 alpha | 18 |  |  |  |  |  |  |  |  | + |
| IL37_HUMAN | Q9NZH6 | Interleukin-37 | 24 |  |  |  |  |  |  |  |  | + |
| IL4RA_HUMAN | P24394 | Interleukin-4 receptor subunit alpha | 90 |  |  |  |  |  |  | + |  | + |
| IL6_HUMAN | P05231 | Interleukin-6 | 24 |  |  |  |  |  |  | + |  | + |
| IL8_HUMAN | P10145 | Interleukin-8 | 11 |  |  |  |  |  |  | + |  | + |
| IMB1_HUMAN | Q14974 | Importin subunit beta-1 | 97 | 1 | 1.4% |  | 3 | 4.9% |  |  |  |  |
| INHBB_HUMAN | P09529 | Inhibin beta B chain | 45 |  |  |  |  |  |  | + |  | + |
| IPYR_HUMAN | Q15181 | Inorganic pyrophosphatase | 33 | 1 | 7.6% |  | 3 | 16.3% |  |  |  |  |
| IQGA1_HUMAN | P46940 | Ras GTPase-activating-like protein IQGAP1 | 189 | 3 | 3.1% |  | 5 | 6.2% |  |  |  |  |
| ISOC1_HUMAN | Q96CN7 | Isochorismatase domain-containing protein 1 | 32 | 5 | 24.5% |  | 4 | 22.5% |  |  |  |  |
| ITIH1_HUMAN | P19827 | Inter-alpha-trypsin inhibitor heavy chain H1 | 101 | 11 | 14.7% |  | 6 | 7.7% |  |  |  |  |
| ITIH2_HUMAN | P19823 | Inter-alpha-trypsin inhibitor heavy chain H2 | 106 | 14 | 20.5% |  | 12 | 15.5% |  |  |  |  |
| ITIH4_HUMAN | Q14624 | Inter-alpha-trypsin inhibitor heavy chain H4 | 103 | 5 | 7.1% |  | 6 | 9.4% |  |  |  |  |
| K1C10_HUMAN | P13645 | Keratin, type I cytoskeletal 10 | 59 | 11 | 33.0% |  | 25 | 54.8% |  |  |  |  |
| K1C9_HUMAN | P35527 | Keratin, type I cytoskeletal 9 | 62 | 11 | 29.9% |  | 5 | 10.3% |  |  |  |  |
| K22E_HUMAN | P35908 | Keratin, type II cytoskeletal 2 epidermal | 65 | 5 | 13.0% |  | 19 | 40.1% |  |  |  |  |
| K2C1_HUMAN | P04264 | Keratin, type II cytoskeletal 1 | 66 | 16 | 39.4% |  | 14 | 20% |  |  |  |  |
| KAD1_HUMAN | P00568 | Adenylate kinase isoenzyme 1 | 22 | 0 | 0% |  | 2 | 17% |  |  |  |  |
| KAD2_HUMAN | P54819 | Adenylate kinase 2, mitochondrial | 26 | 1 | 10.5% |  | 3 | 22.6% |  |  |  |  |
| KAD3_HUMAN | Q9UIJ7 | GTP:AMP phosphotransferase AK3, mitochondrial | 26 | 0 | 0% |  | 3 | 15.4% |  |  |  |  |
| KAP3_HUMAN | P31323 | cAMP-dependent protein kinase type II-beta regulatory subunit | 46 | 1 | 4.1% |  | 2 | 7.7% |  |  |  |  |
| KCD12_HUMAN | Q96CX2 | BTB/POZ domain-containing protein KCTD12 | 36 | 5 | 22.8% |  | 4 | 16.0% |  |  |  |  |
| KCRB_HUMAN | P12277 | Creatine kinase B-type | 43 | 3 | 12.1% |  | 6 | 23.1% |  |  |  |  |
| KLKB1_HUMAN | P03952 | Plasma kallikrein | 71 | 0 | 0% |  | 4 | 7.2% |  |  |  |  |
| KREM2_HUMAN | Q8NCW0 | Kremen protein 2 | 49 |  |  |  |  |  |  | + |  | + |
| KV106_HUMAN | P01598 | Ig kappa chain V-I region EU | 12 | 2 | 26.9% |  | 1 | 16.7% |  |  |  |  |
| KV114_HUMAN | P01606 | Ig kappa chain V-I region OU | 12 | 2 | 22.2% |  | 2 | 22.2% |  |  |  |  |
| KV121_HUMAN | P01613 | Ig kappa chain V-I region Ni | 12 | 2 | 30.4% |  | 1 | 14.3% |  |  |  |  |
| KV202_HUMAN | P01615 | Ig kappa chain V-II region FR | 13 | 1 | 7.1% |  | 2 | 18.6% |  |  |  |  |
| KV204_HUMAN | P01617 | Ig kappa chain V-II region TEW | 12 | 2 | 38.9% |  | 2 | 38.9% |  |  |  |  |
| KV206_HUMAN | P06310 | Ig kappa chain V-II region RPMI 6410 | 15 | 3 | 34.6% |  | 1 | 9.8% |  |  |  |  |
| KV302_HUMAN | P01620 | Ig kappa chain V-III region SIE | 12 | 4 | 45.9% |  | 5 | 66.1% |  |  |  |  |
| KV307_HUMAN | P04206 | Ig kappa chain V-III region GOL | 12 | 0 | 0% |  | 2 | 63.3% |  |  |  |  |
| KV308_HUMAN | P04207 | Ig kappa chain V-III region CLL | 14 | 2 | 19.4% |  | 1 | 7% |  |  |  |  |
| KV402_HUMAN | P01625 | Ig kappa chain V-IV region Len | 13 | 3 | 36.8% |  | 2 | 23.7% |  |  |  |  |
| LAC2_HUMAN | P0CG05 | Ig lambda-2 chain C regions | 11 | 5 | 93.4% |  | 5 | 93.4% |  |  |  |  |
| LAC3_HUMAN | P0CG06 | Ig lambda-3 chain C regions | 11 | 2 | 96.2% |  | 1 | 93.4% |  |  |  |  |
| LAC7_HUMAN | A0M8Q6 | Ig lambda-7 chain C region | 11 | 1 | 50.9% |  | 2 | 60.4% |  |  |  |  |
| LAMA2_HUMAN | P24043 | Laminin subunit alpha-2 | 344 | 0 | 0% |  | 10 | 4.1% |  |  |  |  |
| LAMA4_HUMAN | Q16363 | Laminin subunit alpha-4 | 203 | 42 | 28.7% |  | 24 | 17.8% |  |  |  |  |
| LAMB1_HUMAN | P07942 | Laminin subunit beta-1 | 198 | 27 | 21.2% |  | 26 | 21.6% |  |  |  |  |
| LAMB2_HUMAN | P55268 | Laminin subunit beta-2 | 196 | 24 | 19.4% |  | 28 | 20% |  |  |  |  |
| LAMC1_HUMAN | P11047 | Laminin subunit gamma-1 | 178 | 43 | 36.7% |  | 31 | 25.7% |  |  |  |  |
| LBP_HUMAN | P18428 | Lipopolysaccharide-binding protein | 53 |  |  |  |  |  |  | + |  |  |
| LCK_HUMAN | P06239 | Tyrosine-protein kinase Lck | 58 |  |  |  |  |  |  |  |  | + |
| LCN1_HUMAN | P31025 | Lipocalin-1 | 19 |  |  |  |  |  |  | + |  | + |
| LDHA_HUMAN | P00338 | L-lactate dehydrogenase A chain | 37 | 23 | 69.3% |  | 23 | 64.2% |  |  |  |  |
| LDHB_HUMAN | P07195 | L-lactate dehydrogenase B chain | 37 | 17 | 53.3% |  | 22 | 60.2% |  |  |  |  |
| LEG1_HUMAN | P09382 | Galectin-1 | 15 | 10 | 80.7% |  | 10 | 80.7% |  |  |  |  |
| LEG3_HUMAN | P17931 | Galectin-3 | 26 | 3 | 16.0% |  | 3 | 15.6% |  | + |  | + |
| LEPR_HUMAN | P48357 | Leptin receptor | 132 |  |  |  |  |  |  | + |  | + |
| LEP_HUMAN | P41159 | Leptin (Ob) | 16 |  |  |  |  |  |  | + |  |  |
| LFTY2_HUMAN | O00292 | Left-right determination factor 2 | 41 |  |  |  |  |  |  | + |  | + |
| LG3BP_HUMAN | Q08380 | Galectin-3-binding protein | 65 | 2 | 4.6% |  | 1 | 1.9% |  |  |  |  |
| LGUL_HUMAN | Q04760 | Lactoylglutathione lyase | 21 | 3 | 19.6% |  | 3 | 19.6% |  |  |  |  |
| LHPP_HUMAN | Q9H008 | Phospholysine phosphohistidine inorganic pyrophosphate phosphatase | 29 | 0 | 0% |  | 6 | 35.6% |  |  |  |  |
| LIF_HUMAN | P15018 | Leukemia inhibitory factor | 22 |  |  |  |  |  |  |  |  | + |
| LIFR_HUMAN | P42702 | Leukemia inhibitory factor receptor | 124 |  |  |  |  |  |  | + |  | + |
| LIPS_HUMAN | Q05469 | Hormone-sensitive lipase | 117 | 9 | 12.4% |  | 6 | 10.6% |  |  |  |  |
| LIS1_HUMAN | P43034 | Platelet-activating factor acetylhydrolase IB subunit alpha | 47 | 0 | 0% |  | 2 | 6.8% |  |  |  |  |
| LMNA_HUMAN | P02545 | Prelamin-A/C | 74 | 17 | 27.7% |  | 23 | 39.2% |  |  |  |  |
| LMNB2_HUMAN | Q03252 | Lamin-B2 | 68 | 2 | 5.3% |  | 3 | 7.3% |  |  |  |  |
| LRP6_HUMAN | O75581 | Low-density lipoprotein receptor-related protein 6 | 180 |  |  |  |  |  |  | + |  |  |
| LUM_HUMAN | P51884 | Lumican | 38 | 14 | 39.9% |  | 13 | 39.9% |  |  |  |  |
| LV102_HUMAN | P01700 | Ig lambda chain V-I region HA | 12 | 2 | 23.2% |  | 0 | 0% |  |  |  |  |
| LV301_HUMAN | P01714 | Ig lambda chain V-III region SH | 11 | 2 | 25% |  | 2 | 25% |  |  |  |  |
| LV302_HUMAN | P80748 | Ig lambda chain V-III region LOI | 12 | 3 | 37.8% |  | 3 | 37.8% |  |  |  |  |
| LYSC_HUMAN | P61626 | Lysozyme C | 17 | 2 | 29.1% |  | 0 | 0% |  |  |  |  |
| MAP4_HUMAN | P27816 | Microtubule-associated protein 4 | 121 | 2 | 3.7% |  | 5 | 7.3% |  |  |  |  |
| MAT2B_HUMAN | Q9NZL9 | Methionine adenosyltransferase 2 subunit beta | 38 | 0 | 0% |  | 2 | 11.1% |  |  |  |  |
| MATR3_HUMAN | P43243 | Matrin-3 | 95 | 2 | 4.6% |  | 0 | 0% |  |  |  |  |
| MDHC_HUMAN | P40925 | Malate dehydrogenase, cytoplasmic | 36 | 14 | 54.8% |  | 14 | 53.9% |  |  |  |  |
| MDHM_HUMAN | P40926 | Malate dehydrogenase, mitochondrial | 36 | 9 | 34.3% |  | 17 | 57.1% |  |  |  |  |
| MFGM_HUMAN | Q08431 | Lactadherin | 43 |  |  |  |  |  |  | + |  | + |
| MGLL_HUMAN | Q99685 | Monoglyceride lipase | 33 | 7 | 30.4% |  | 9 | 37.0% |  |  |  |  |
| MIF_HUMAN | P14174 | Macrophage migration inhibitory factor | 12 | 1 | 9.6% |  | 2 | 17.4% |  |  |  |  |
| MIME_HUMAN | P20774 | Mimecan | 34 | 7 | 29.2% |  | 11 | 31.9% |  |  |  |  |
| MMP1_HUMAN | P03956 | Interstitial collagenase (Matrix metalloproteinase-1) | 54 |  |  |  |  |  |  | + |  |  |
| MMP10_HUMAN | P09238 | Stromelysin-2 (Matrix metalloproteinase-10) | 54 |  |  |  |  |  |  | + |  | + |
| MMP2_HUMAN | P08253 | 72 kDa type IV collagenase (Matrix metalloproteinase-2) | 74 |  |  |  |  |  |  |  |  | + |
| MMP3_HUMAN | P08254 | Stromelysin-1 (Matrix metalloproteinase-3) | 54 |  |  |  |  |  |  | + |  | + |
| MMP7_HUMAN | P09237 | Matrilysin (Matrix metalloproteinase-7) | 30 |  |  |  |  |  |  | + |  | + |
| MMP8_HUMAN | P22894 | Neutrophil collagenase (Matrix metalloproteinase-8) | 53 |  |  |  |  |  |  |  |  | + |
| MMP9_HUMAN | P14780 | Matrix metalloproteinase-9 | 78 |  |  |  |  |  |  | + |  |  |
| MOES_HUMAN | P26038 | Moesin | 68 | 0 | 0% |  | 4 | 8.5% |  |  |  |  |
| MRC1_HUMAN | P22897 | Macrophage mannose receptor 1 | 166 | 0 | 0% |  | 5 | 4.3% |  |  |  |  |
| MTAP_HUMAN | Q13126 | S-methyl-5'-thioadenosine phosphorylase | 31 | 1 | 6.4% |  | 2 | 14.5% |  |  |  |  |
| MUC18_HUMAN | P43121 | Cell surface glycoprotein MUC18 | 72 | 11 | 23.2% |  | 17 | 35.6% |  |  |  |  |
| MUCB_HUMAN | P04220 | Ig mu heavy chain disease protein | 43 | 3 | 41.4% |  | 4 | 51.4% |  |  |  |  |
| MYH9_HUMAN | P35579 | Myosin-9 | 227 | 7 | 5.6% |  | 14 | 11.3% |  |  |  |  |
| MYL6_HUMAN | P60660 | Myosin light polypeptide 6 | 17 | 2 | 19.2% |  | 3 | 26.5% |  |  |  |  |
| MYO1C_HUMAN | O00159 | Unconventional myosin-Ic | 122 | 2 | 3.3% |  | 0 | 0% |  |  |  |  |
| NACAM_HUMAN | E9PAV3 | Nascent polypeptide-associated complex subunit alpha, muscle-specific form | 205 | 2 | 1.4% |  | 1 | 0.7% |  |  |  |  |
| NAGK_HUMAN | Q9UJ70 | N-acetyl-D-glucosamine kinase | 37 | 1 | 3.8% |  | 3 | 10.5% |  |  |  |  |
| NB5R3_HUMAN | P00387 | NADH-cytochrome b5 reductase 3 | 34 | 1 | 3.7% |  | 3 | 14% |  |  |  |  |
| NBL1_HUMAN | P41271 | Neuroblastoma suppressor of tumorigenicity 1 | 19 |  |  |  |  |  |  |  |  | + |
| NDKB_HUMAN | P22392 | Nucleoside diphosphate kinase B | 17 | 7 | 52% |  | 7 | 60.5% |  |  |  |  |
| NID1_HUMAN | P14543 | Nidogen-1 | 136 | 5 | 6.4% |  | 10 | 13.1% |  |  |  |  |
| NID2_HUMAN | Q14112 | Nidogen-2 | 151 | 3 | 4.6% |  | 6 | 8.2% |  |  |  |  |
| NIT2_HUMAN | Q9NQR4 | Omega-amidase NIT2 | 31 | 1 | 5.4% |  | 4 | 23.6% |  |  |  |  |
| NNRE_HUMAN | Q8NCW5 | NAD(P)H-hydrate epimerase | 32 | 1 | 6.6% |  | 2 | 12.8% |  |  |  |  |
| NQO1_HUMAN | P15559 | NAD(P)H dehydrogenase [quinone] 1 | 31 | 0 | 0% |  | 5 | 18.6% |  |  |  |  |
| NRG1_HUMAN | Q02297 | Pro-neuregulin-1 | 70 |  |  |  |  |  |  | + |  | + |
| NRG2_HUMAN | O14511 | Pro-neuregulin-2 | 92 |  |  |  |  |  |  | + |  |  |
| NRN1_HUMAN | Q9NPD7 | Neuritin | 15 |  |  |  |  |  |  |  |  | + |
| NRTN_HUMAN | Q99748 | Neurturin | 22 |  |  |  |  |  |  | + |  | + |
| NUDT5_HUMAN | Q9UKK9 | ADP-sugar pyrophosphatase | 24 | 0 | 0% |  | 3 | 15.1% |  |  |  |  |
| NUMA1_HUMAN | Q14980 | Nuclear mitotic apparatus protein 1 | 238 | 2 | 1.3% |  | 0 | 0% |  |  |  |  |
| ONCM_HUMAN | P13725 | Oncostatin-M | 28 | 0 | 0% |  | 14 | 52% |  | + |  | + |
| OPLA_HUMAN | O14841 | 5-oxoprolinase | 137 | 0 | 0% |  | 6 | 7.1% |  |  |  |  |
| OREX_HUMAN | O43612 | Orexin | 13 |  |  |  |  |  |  | + |  | + |
| OTUB1_HUMAN | Q96FW1 | Ubiquitin thioesterase OTUB1 | 31 | 0 | 0% |  | 2 | 8.9% |  |  |  |  |
| PAI1_HUMAN | P05121 | Plasminogen activator inhibitor 1 | 45 | 0 | 0% |  | 6 | 12.7% |  |  |  |  |
| PARK7_HUMAN | Q99497 | Protein DJ-1 | 20 | 11 | 75.7% |  | 11 | 75.1% |  |  |  |  |
| PARVA_HUMAN | Q9NVD7 | Alpha-parvin | 42 | 5 | 16.7% |  | 10 | 25.3% |  |  |  |  |
| PCBP1_HUMAN | Q15365 | Poly(rC)-binding protein 1 | 37 | 3 | 8.7% |  | 5 | 20.5% |  |  |  |  |
| PD2R2_HUMAN | Q9Y5Y4 | Prostaglandin D2 receptor 2 | 43 |  |  |  |  |  |  | + |  | + |
| PDCD6_HUMAN | O75340 | Programmed cell death protein 6 | 22 | 2 | 11% |  | 0 | 0% |  |  |  |  |
| PDIA3_HUMAN | P30101 | Protein disulfide-isomerase A3 | 57 | 1 | 2.2% |  | 2 | 4.6% |  |  |  |  |
| PDIA6_HUMAN | Q15084 | Protein disulfide-isomerase A6 | 48 | 2 | 9.1% |  | 2 | 9.1% |  |  |  |  |
| PDXK_HUMAN | O00764 | Pyridoxal kinase | 35 | 7 | 27.9% |  | 9 | 42.6% |  |  |  |  |
| PEA15_HUMAN | Q15121 | Astrocytic phosphoprotein PEA-15 | 15 | 0 | 0% |  | 2 | 18.5% |  |  |  |  |
| PEBP1_HUMAN | P30086 | Phosphatidylethanolamine-binding protein 1 | 21 | 7 | 64.2% |  | 10 | 75.9% |  |  |  |  |
| PEDF_HUMAN | P36955 | Pigment epithelium-derived factor | 46 | 1 | 3.4% |  | 13 | 38.8% |  |  |  |  |
| PGAM1_HUMAN | P18669 | Phosphoglycerate mutase 1 | 29 | 9 | 51.6% |  | 11 | 61% |  |  |  |  |
| PGBM_HUMAN | P98160 | Basement membrane-specific heparan sulfate proteoglycan core protein | 469 | 17 | 6% |  | 23 | 6.8% |  |  |  |  |
| PGK1_HUMAN | P00558 | Phosphoglycerate kinase 1 | 45 | 4 | 16.1% |  | 16 | 52.8% |  |  |  |  |
| PGM1_HUMAN | P36871 | Phosphoglucomutase-1 | 61 | 4 | 12.5% |  | 4 | 12.8% |  |  |  |  |
| PGRC2_HUMAN | O15173 | Membrane-associated progesterone receptor component 2 | 24 | 3 | 16.1% |  | 0 | 0% |  |  |  |  |
| PHS_HUMAN | P61457 | Pterin-4-alpha-carbinolamine dehydratase | 12 | 5 | 57.7% |  | 3 | 38.5% |  |  |  |  |
| PIGR_HUMAN | P01833 | Polymeric immunoglobulin receptor | 83 | 10 | 18.2% |  | 0 | 0% |  |  |  |  |
| PIP_HUMAN | P12273 | Prolactin-inducible protein | 17 | 7 | 69.9% |  | 0 | 0% |  |  |  |  |
| PIPNB_HUMAN | P48739 | Phosphatidylinositol transfer protein beta isoform | 32 | 0 | 0% |  | 2 | 11.8% |  |  |  |  |
| PLEC_HUMAN | Q15149 | Plectin | 532 | 0 | 0% |  | 9 | 2.3% |  |  |  |  |
| PLIN1_HUMAN | O60240 | Perilipin-1 | 56 | 14 | 38.7% |  | 15 | 41.8% |  |  |  |  |
| PLIN3_HUMAN | O60664 | Perilipin-3 | 47 | 0 | 0% |  | 2 | 10.1% |  |  |  |  |
| PLIN4_HUMAN | Q96Q06 | Perilipin-4 | 134 | 41 | 44.6% |  | 65 | 61.1% |  |  |  |  |
| PLMN_HUMAN | P00747 | Plasminogen | 91 | 2 | 2.4% |  | 1 | 1% |  |  |  |  |
| PLTP_HUMAN | P55058 | Phospholipid transfer protein | 55 | 2 | 8.1% |  | 2 | 4.5% |  |  |  |  |
| PMGE_HUMAN | P07738 | Bisphosphoglycerate mutase | 30 | 2 | 8.1% |  | 1 | 3.9% |  |  |  |  |
| POTEF_HUMAN | A5A3E0 | POTE ankyrin domain family member F | 121 | 0 | 0% |  | 2 | 8.1% |  |  |  |  |
| POTEI_HUMAN | P0CG38 | POTE ankyrin domain family member I | 121 | 0 | 4.2% |  | 2 | 6.6% |  |  |  |  |
| PP1A_HUMAN | P62136 | Serine/threonine-protein phosphatase PP1-alpha catalytic subunit | 38 | 3 | 11.2% |  | 3 | 11.2% |  |  |  |  |
| PPIA_HUMAN | P62937 | Peptidyl-prolyl cis-trans isomerase A | 18 | 7 | 64.8% |  | 10 | 76.4% |  |  |  |  |
| PPIB_HUMAN | P23284 | Peptidyl-prolyl cis-trans isomerase B | 24 | 7 | 36.1% |  | 8 | 34.7% |  |  |  |  |
| PRDBP_HUMAN | Q969G5 | Protein kinase C delta-binding protein | 28 | 2 | 8.1% |  | 1 | 3.8% |  |  |  |  |
| PRDX1_HUMAN | Q06830 | Peroxiredoxin-1 | 22 | 10 | 55.3% |  | 6 | 36.2% |  |  |  |  |
| PRDX2_HUMAN | P32119 | Peroxiredoxin-2 | 22 | 12 | 53.0% |  | 14 | 51.5% |  |  |  |  |
| PRDX3_HUMAN | P30048 | Thioredoxin-dependent peroxide reductase, mitochondrial | 28 | 0 | 0% |  | 2 | 10.2% |  |  |  |  |
| PRDX5_HUMAN | P30044 | Peroxiredoxin-5, mitochondrial | 22 | 1 | 7.5% |  | 6 | 36% |  |  |  |  |
| PRDX6_HUMAN | P30041 | Peroxiredoxin-6 | 25 | 9 | 62.9% |  | 15 | 78.6% |  |  |  |  |
| PROF1_HUMAN | P07737 | Profilin-1 | 15 | 5 | 40.7% |  | 7 | 59.3% |  |  |  |  |
| PROK1_HUMAN | P58294 | Prokineticin-1 | 12 |  |  |  |  |  |  |  |  | + |
| PRS8_HUMAN | P62195 | 26S protease regulatory subunit 8 | 46 | 1 | 3.2% |  | 2 | 5.4% |  |  |  |  |
| PSA_HUMAN | P55786 | Puromycin-sensitive aminopeptidase | 103 | 2 | 4.4% |  | 3 | 4.1% |  |  |  |  |
| PSA1_HUMAN | P25786 | Proteasome subunit alpha type-1 | 30 | 2 | 10.3% |  | 4 | 13.7% |  |  |  |  |
| PSA2_HUMAN | P25787 | Proteasome subunit alpha type-2 | 26 | 0 | 0% |  | 2 | 18.4% |  |  |  |  |
| PSA3_HUMAN | P25788 | Proteasome subunit alpha type-3 | 28 | 2 | 10.2% |  | 2 | 9.4% |  |  |  |  |
| PSA4_HUMAN | P25789 | Proteasome subunit alpha type-4 | 29 | 3 | 20.3% |  | 5 | 24.9% |  |  |  |  |
| PSA5_HUMAN | P28066 | Proteasome subunit alpha type-5 | 26 | 3 | 18.3% |  | 3 | 14.5% |  |  |  |  |
| PSA6_HUMAN | P60900 | Proteasome subunit alpha type-6 | 27 | 1 | 4.1% |  | 5 | 24.8% |  |  |  |  |
| PSA7_HUMAN | O14818 | Proteasome subunit alpha type-7 | 28 | 3 | 19% |  | 7 | 46.8% |  |  |  |  |
| PSB1_HUMAN | P20618 | Proteasome subunit beta type-1 | 26 | 0 | 0% |  | 3 | 24.5% |  |  |  |  |
| PSB2_HUMAN | P49721 | Proteasome subunit beta type-2 | 23 | 3 | 17.9% |  | 2 | 13.9% |  |  |  |  |
| PSB3_HUMAN | P49720 | Proteasome subunit beta type-3 | 23 | 3 | 19% |  | 3 | 29.3% |  |  |  |  |
| PSB4_HUMAN | P28070 | Proteasome subunit beta type-4 | 29 | 2 | 12.9% |  | 2 | 12.9% |  |  |  |  |
| PSB5_HUMAN | P28074 | Proteasome subunit beta type-5 | 28 | 8 | 28.1% |  | 9 | 31.9% |  |  |  |  |
| PSB6_HUMAN | P28072 | Proteasome subunit beta type-6 | 25 | 2 | 8.4% |  | 3 | 13% |  |  |  |  |
| PSD13_HUMAN | Q9UNM6 | 26S proteasome non-ATPase regulatory subunit 13 | 43 | 0 | 0% |  | 2 | 6.4% |  |  |  |  |
| PSDE_HUMAN | O00487 | 26S proteasome non-ATPase regulatory subunit 14 | 35 | 0 | 0% |  | 2 | 18.1% |  |  |  |  |
| PSMD2_HUMAN | Q13200 | 26S proteasome non-ATPase regulatory subunit 2 | 100 | 0 | 0% |  | 2 | 4.1% |  |  |  |  |
| PSMD6_HUMAN | Q15008 | 26S proteasome non-ATPase regulatory subunit 6 | 46 | 0 | 0% |  | 2 | 6.2% |  |  |  |  |
| PSMD7_HUMAN | P51665 | 26S proteasome non-ATPase regulatory subunit 7 | 37 | 2 | 6.8% |  | 5 | 21.9% |  |  |  |  |
| PSME1_HUMAN | Q06323 | Proteasome activator complex subunit 1 | 29 | 2 | 14.9% |  | 2 | 10.4% |  |  |  |  |
| PSME2_HUMAN | Q9UL46 | Proteasome activator complex subunit 2 | 27 | 2 | 14.6% |  | 4 | 27.6% |  |  |  |  |
| PTBP1_HUMAN | P26599 | Polypyrimidine tract-binding protein 1 | 57 | 3 | 9.2% |  | 3 | 11.1% |  |  |  |  |
| PTER_HUMAN | Q96BW5 | Phosphotriesterase-related protein | 39 | 2 | 10.3% |  | 1 | 3.2% |  |  |  |  |
| PTGR1_HUMAN | Q14914 | Prostaglandin reductase 1 | 36 | 7 | 29.2% |  | 10 | 38% |  |  |  |  |
| PTMA_HUMAN | P06454 | Prothymosin alpha | 12 | 2 | 12.6% |  | 2 | 12.6% |  |  |  |  |
| PTN11_HUMAN | Q06124 | Tyrosine-protein phosphatase non-receptor type 11 | 68 | 1 | 1.8% |  | 4 | 7.4% |  |  |  |  |
| PTPA_HUMAN | Q15257 | Serine/threonine-protein phosphatase 2A activator | 41 | 0 | 0% |  | 4 | 24.3% |  |  |  |  |
| PTPRS_HUMAN | Q13332 | Receptor-type tyrosine-protein phosphatase S | 217 | 0 | 0% |  | 2 | 1.5% |  |  |  |  |
| PTRF_HUMAN | Q6NZI2 | Polymerase I and transcript release factor | 43 | 5 | 21.0% |  | 9 | 25.1% |  |  |  |  |
| PUR6_HUMAN | P22234 | Multifunctional protein ADE2 | 47 | 0 | 0% |  | 2 | 6.8% |  |  |  |  |
| PXDC2_HUMAN | Q6UX71 | Plexin domain-containing protein 2 | 60 | 2 | 4% |  | 1 | 2.1% |  |  |  |  |
| PYGB_HUMAN | P11216 | Glycogen phosphorylase, brain form | 97 | 1 | 6.9% |  | 10 | 23.3% |  |  |  |  |
| PYGL_HUMAN | P06737 | Glycogen phosphorylase, liver form | 97 | 9 | 16.1% |  | 40 | 52.3% |  |  |  |  |
| PZP_HUMAN | P20742 | Pregnancy zone protein | 164 | 1 | 6% |  | 3 | 9.3% |  |  |  |  |
| Q9NS53_HUMAN | Q9NS53 | Preadipocyte factor | 2 |  |  |  |  |  |  |  |  | + |
| QOR_HUMAN | Q08257 | Quinone oxidoreductase | 35 | 4 | 21% |  | 4 | 20.4% |  |  |  |  |
| RAB1A_HUMAN | P62820 | Ras-related protein Rab-1A | 23 | 2 | 13.2% |  | 3 | 18.5% |  |  |  |  |
| RAB2A_HUMAN | P61019 | Ras-related protein Rab-2A | 24 | 0 | 0% |  | 3 | 19.3% |  |  |  |  |
| RAB5C_HUMAN | P51148 | Ras-related protein Rab-5C | 23 | 2 | 11.1% |  | 2 | 11.1% |  |  |  |  |
| RAP1A_HUMAN | P62834 | Ras-related protein Rap-1A | 21 | 4 | 20.1% |  | 3 | 13.6% |  |  |  |  |
| REEP6_HUMAN | Q96HR9 | Receptor expression-enhancing protein 6 | 21 | 1 | 7.1% |  | 2 | 11.4% |  |  |  |  |
| RET4_HUMAN | P02753 | Retinol-binding protein 4 | 23 | 12 | 75.1% |  | 10 | 59.2% |  |  |  |  |
| RHOA_HUMAN | P61586 | Transforming protein RhoA | 22 | 3 | 14% |  | 0 | 0% |  |  |  |  |
| RINI_HUMAN | P13489 | Ribonuclease inhibitor | 50 | 0 | 0% |  | 12 | 47.9% |  |  |  |  |
| RLA0_HUMAN | P05388 | 60S acidic ribosomal protein P0 | 34 | 4 | 19.6% |  | 5 | 25.6% |  |  |  |  |
| RLA1_HUMAN | P05386 | 60S acidic ribosomal protein P1 | 12 | 1 | 37.7% |  | 2 | 51.8% |  |  |  |  |
| RNAS4_HUMAN | P34096 | Ribonuclease 4 | 17 | 2 | 19% |  | 1 | 7.5% |  |  |  |  |
| ROBO4_HUMAN | Q8WZ75 | Roundabout homolog 4 | 107 |  |  |  |  |  |  | + |  | + |
| RRAS2_HUMAN | P62070 | Ras-related protein R-Ras2 | 23 | 0 | 0% |  | 2 | 11.3% |  |  |  |  |
| RRBP1_HUMAN | Q9P2E9 | Ribosome-binding protein 1 | 152 | 1 | 0.9% |  | 3 | 1.9% |  |  |  |  |
| RS25_HUMAN | P62851 | 40S ribosomal protein S25 | 14 | 2 | 19.2% |  | 0 | 0% |  |  |  |  |
| RS27A_HUMAN | P62979 | Ubiquitin-40S ribosomal protein S27a | 18 | 3 | 21.8% |  | 6 | 35.3% |  |  |  |  |
| RS3_HUMAN | P23396 | 40S ribosomal protein S3 | 27 | 1 | 5.8% |  | 3 | 17.7% |  |  |  |  |
| RSSA_HUMAN | P08865 | 40S ribosomal protein SA | 33 | 0 | 0% |  | 3 | 19.3% |  |  |  |  |
| RSU1_HUMAN | Q15404 | Ras suppressor protein 1 | 32 | 5 | 33.6% |  | 9 | 52.7% |  |  |  |  |
| RTN1_HUMAN | Q16799 | Reticulon-1 | 84 | 0 | 0% |  | 2 | 3.7% |  |  |  |  |
| RTN4_HUMAN | Q9NQC3 | Reticulon-4 | 130 | 3 | 3.2% |  | 3 | 3.1% |  |  |  |  |
| S10A1_HUMAN | P23297 | Protein S100-A1 | 11 | 2 | 23.4% |  | 1 | 10.6% |  |  |  |  |
| S10A4_HUMAN | P26447 | Protein S100-A4 | 12 | 4 | 36.6% |  | 5 | 36.6% |  |  |  |  |
| S10A6_HUMAN | P06703 | Protein S100-A6 | 10 | 1 | 8.9% |  | 3 | 22.2% |  |  |  |  |
| S10A8_HUMAN | P05109 | Protein S100-A8 | 11 | 4 | 33.3% |  | 2 | 23.7% |  |  |  |  |
| S10A9_HUMAN | P06702 | Protein S100-A9 | 13 | 2 | 24.6% |  | 0 | 0% |  |  |  |  |
| S10AA_HUMAN | P60903 | Protein S100-A10 | 11 | 2 | 27.8% |  | 2 | 27.8% |  | + |  |  |
| S10AB_HUMAN | P31949 | Protein S100-A11 | 12 | 5 | 67.6% |  | 4 | 46.7% |  |  |  |  |
| S10AD_HUMAN | Q99584 | Protein S100-A13 | 11 | 3 | 48% |  | 0 | 0% |  |  |  |  |
| S10AG_HUMAN | Q96FQ6 | Protein S100-A16 | 12 | 2 | 21.4% |  | 1 | 10.7% |  |  |  |  |
| SAA4_HUMAN | P35542 | Serum amyloid A-4 protein | 15 | 4 | 32.3% |  | 3 | 23.1% |  |  |  |  |
| SAHH_HUMAN | P23526 | Adenosylhomocysteinase | 48 | 0 | 0% |  | 3 | 9.3% |  |  |  |  |
| SAMP_HUMAN | P02743 | Serum amyloid P-component | 25 | 6 | 26% |  | 0 | 0% |  |  |  |  |
| SAP_HUMAN | P07602 | Prosaposin | 58 | 4 | 8.6% |  | 5 | 10.7% |  |  |  |  |
| SBP1_HUMAN | Q13228 | Selenium-binding protein 1 | 52 | 7 | 21.0% |  | 11 | 30.3% |  |  |  |  |
| SC22B_HUMAN | O75396 | Vesicle-trafficking protein SEC22b | 25 | 3 | 10.7% |  | 4 | 15.3% |  |  |  |  |
| SCRN2_HUMAN | Q96FV2 | Secernin-2 | 47 | 0 | 0% |  | 2 | 9.2% |  |  |  |  |
| SDPR_HUMAN | O95810 | Serum deprivation-response protein | 47 | 2 | 6.6% |  | 4 | 10.6% |  |  |  |  |
| SEPT2_HUMAN | Q15019 | Septin-2 | 41 | 0 | 0% |  | 5 | 18.8% |  |  |  |  |
| SGTA_HUMAN | O43765 | Small glutamine-rich tetratricopeptide repeat-containing protein alpha | 34 | 0 | 0% |  | 2 | 8.3% |  |  |  |  |
| SH3L1_HUMAN | O75368 | SH3 domain-binding glutamic acid-rich-like protein | 13 | 3 | 24.6% |  | 0 | 0% |  |  |  |  |
| SH3L3_HUMAN | Q9H299 | SH3 domain-binding glutamic acid-rich-like protein 3 | 10 | 3 | 43% |  | 3 | 43% |  |  |  |  |
| SIGIR_HUMAN | Q6IA17 | Single Ig IL-1-related receptor | 46 |  |  |  |  |  |  | + |  | + |
| SIGL9_HUMAN | Q9Y336 | Sialic acid-binding Ig-like lectin 9 | 50 |  |  |  |  |  |  | + |  | + |
| SMD1_HUMAN | P62314 | Small nuclear ribonucleoprotein Sm D1 | 13 | 2 | 27.7% |  | 2 | 27.7% |  |  |  |  |
| SNAA_HUMAN | P54920 | Alpha-soluble NSF attachment protein | 33 | 2 | 8.5% |  | 6 | 28.1% |  |  |  |  |
| SODC_HUMAN | P00441 | Superoxide dismutase [Cu-Zn] | 16 | 7 | 70.8% |  | 6 | 42.9% |  |  |  |  |
| SODE_HUMAN | P08294 | Extracellular superoxide dismutase [Cu-Zn] | 26 | 10 | 44.2% |  | 11 | 44.2% |  |  |  |  |
| SODM_HUMAN | P04179 | Superoxide dismutase [Mn], mitochondrial | 25 | 2 | 12.6% |  | 3 | 19.4% |  |  |  |  |
| SORCN_HUMAN | P30626 | Sorcin | 22 | 2 | 17.2% |  | 1 | 5.6% |  |  |  |  |
| SPB6_HUMAN | P35237 | Serpin B6 | 43 | 0 | 0% |  | 11 | 39.4% |  |  |  |  |
| SPRC_HUMAN | P09486 | SPARC | 35 | 0 | 0% |  | 3 | 15.8% |  | + |  | + |
| SPRE_HUMAN | P35270 | Sepiapterin reductase | 28 | 1 | 7.3% |  | 2 | 15.3% |  |  |  |  |
| SPRL1_HUMAN | Q14515 | SPARC-like protein 1 | 75 | 0 | 0% |  | 4 | 9.2% |  |  |  |  |
| SPTB2_HUMAN | Q01082 | Spectrin beta chain, non-erythrocytic 1 | 275 | 57 | 31.6% |  | 77 | 41.6% |  |  |  |  |
| SPTN1_HUMAN | Q13813 | Spectrin alpha chain, non-erythrocytic 1 | 285 | 37 | 24.1% |  | 86 | 46.7% |  |  |  |  |
| SRBS1_HUMAN | Q9BX66 | Sorbin and SH3 domain-containing protein 1 | 143 | 2 | 2% |  | 7 | 5.5% |  |  |  |  |
| SRSF7_HUMAN | Q16629 | Serine/arginine-rich splicing factor 7 | 27 | 2 | 8.8% |  | 0 | 0% |  |  |  |  |
| STOM_HUMAN | P27105 | Erythrocyte band 7 integral membrane protein | 32 | 2 | 12.5% |  | 0 | 0% |  |  |  |  |
| STX7_HUMAN | O15400 | Syntaxin-7 | 30 | 2 | 9.2% |  | 1 | 3.8% |  |  |  |  |
| SYUG_HUMAN | O76070 | Gamma-synuclein | 13 | 1 | 11% |  | 12 | 81.9% |  |  |  |  |
| TAGL_HUMAN | Q01995 | Transgelin | 23 | 11 | 50.7% |  | 8 | 45.3% |  |  |  |  |
| TAGL2_HUMAN | P37802 | Transgelin-2 | 22 | 2 | 13.1% |  | 4 | 23.6% |  |  |  |  |
| TALDO_HUMAN | P37837 | Transaldolase | 38 | 4 | 15.4% |  | 12 | 35.9% |  |  |  |  |
| TBA1B_HUMAN | P68363 | Tubulin alpha-1B chain | 50 | 11 | 41.7% |  | 10 | 36.1% |  |  |  |  |
| TBB5_HUMAN | P07437 | Tubulin beta chain | 50 | 10 | 35.4% |  | 12 | 41.9% |  |  |  |  |
| TBC9B_HUMAN | Q66K14 | TBC1 domain family member 9B | 141 | 0 | 0% |  | 2 | 3% |  |  |  |  |
| TBCA_HUMAN | O75347 | Tubulin-specific chaperone A | 13 | 1 | 9.3% |  | 3 | 27.8% |  |  |  |  |
| TCPZ_HUMAN | P40227 | T-complex protein 1 subunit zeta | 58 | 0 | 0% |  | 2 | 7.5% |  |  |  |  |
| TEFF1_HUMAN | Q8IYR6 | Tomoregulin-1 | 41 |  |  |  |  |  |  | + |  | + |
| TENS1_HUMAN | Q9HBL0 | Tensin-1 | 186 | 0 | 0% |  | 2 | 1.6% |  |  |  |  |
| TENX_HUMAN | P22105 | Tenascin-X | 464 | 10 | 3.2% |  | 11 | 3.4% |  |  |  |  |
| TERA_HUMAN | P55072 | Transitional endoplasmic reticulum ATPase | 89 | 11 | 17.1% |  | 21 | 38.3% |  |  |  |  |
| TETN_HUMAN | P05452 | Tetranectin | 23 | 6 | 35.6% |  | 4 | 29.2% |  |  |  |  |
| TF_HUMAN | P13726 | Tissue factor | 33 |  |  |  |  |  |  | + |  | + |
| TGFB2_HUMAN | P61812 | Transforming growth factor beta-2 | 48 |  |  |  |  |  |  |  |  | + |
| TGFR1_HUMAN | P36897 | TGF-beta receptor type-1 | 56 |  |  |  |  |  |  | + |  |  |
| THIC_HUMAN | Q9BWD1 | Acetyl-CoA acetyltransferase, cytosolic | 41 | 0 | 0% |  | 2 | 9.3% |  |  |  |  |
| THIL_HUMAN | P24752 | Acetyl-CoA acetyltransferase, mitochondrial | 45 | 0 | 0% |  | 3 | 13.3% |  |  |  |  |
| THIM_HUMAN | P42765 | 3-ketoacyl-CoA thiolase, mitochondrial | 42 | 0 | 0% |  | 12 | 48.6% |  |  |  |  |
| THIO_HUMAN | P10599 | Thioredoxin | 12 | 3 | 19% |  | 0 | 0% |  |  |  |  |
| THRB_HUMAN | P00734 | Prothrombin | 70 | 3 | 6.6% |  | 2 | 3.4% |  |  |  |  |
| TIMP1_HUMAN | P01033 | Metalloproteinase inhibitor 1 | 23 |  |  |  |  |  |  | + |  | + |
| TKT_HUMAN | P29401 | Transketolase | 68 | 2 | 7.2% |  | 4 | 12.4% |  |  |  |  |
| TLN1_HUMAN | Q9Y490 | Talin-1 | 270 | 46 | 32.8% |  | 57 | 36.2% |  |  |  |  |
| TLN2_HUMAN | Q9Y4G6 | Talin-2 | 272 | 6 | 6.3% |  | 12 | 10.3% |  |  |  |  |
| TNFC_HUMAN | Q06643 | Lymphotoxin-beta | 25 |  |  |  |  |  |  | + |  | + |
| TNFL8_HUMAN | P32971 | Tumor necrosis factor ligand superfamily member 8 | 26 |  |  |  |  |  |  | + |  | + |
| TNR11_HUMAN | Q9Y6Q6 | Tumor necrosis factor receptor superfamily member 11A | 66 |  |  |  |  |  |  |  |  | + |
| TNR18_HUMAN | Q9Y5U5 | Tumor necrosis factor receptor superfamily member 18 | 26 |  |  |  |  |  |  |  |  | + |
| TPIS_HUMAN | P60174 | Triosephosphate isomerase | 31 | 10 | 46.2% |  | 15 | 70.6% |  |  |  |  |
| TPM3_HUMAN | P06753 | Tropomyosin alpha-3 chain | 33 | 0 | 0% |  | 2 | 19.3% |  |  |  |  |
| TPM4_HUMAN | P67936 | Tropomyosin alpha-4 chain | 29 | 5 | 31.5% |  | 9 | 41.5% |  |  |  |  |
| TPO_HUMAN | P40225 | Thrombopoietin | 38 |  |  |  |  |  |  |  |  | + |
| TR10B_HUMAN | O14763 | Tumor necrosis factor receptor superfamily member 10B | 48 |  |  |  |  |  |  |  |  | + |
| TR11B_HUMAN | O00300 | Tumor necrosis factor receptor superfamily member 11B | 46 |  |  |  |  |  |  | + |  | + |
| TR19L_HUMAN | Q969Z4 | Tumor necrosis factor receptor superfamily member 19L | 46 |  |  |  |  |  |  | + |  | + |
| TRADD_HUMAN | Q15628 | Tumor necrosis factor receptor type 1-associated DEATH domain protein | 34 |  |  |  |  |  |  | + |  | + |
| TRFE_HUMAN | P02787 | Serotransferrin | 77 | 50 | 64.3% |  | 44 | 64.2% |  |  |  |  |
| TRFL_HUMAN | P02788 | Lactotransferrin | 78 | 10 | 21.0% |  | 3 | 7.3% |  |  |  |  |
| TRYB1_HUMAN | Q15661 | Tryptase alpha/beta-1 | 31 | 5 | 21.5% |  | 3 | 14.5% |  |  |  |  |
| TSG6_HUMAN | P98066 | Tumor necrosis factor-inducible gene 6 protein | 31 |  |  |  |  |  |  | + |  | + |
| TSNAX_HUMAN | Q99598 | Translin-associated protein X | 33 | 2 | 7.2% |  | 1 | 6.6% |  |  |  |  |
| TSP1_HUMAN | P07996 | Thrombospondin-1 | 129 | 0 | 0% |  | 4 | 5.56% |  | + |  |  |
| TSP2_HUMAN | P35442 | Thrombospondin-2 | 130 |  |  |  |  |  |  | + |  | + |
| TSP4_HUMAN | P35443 | Thrombospondin-4 | 106 | 4 | 7.9% |  | 10 | 18.8% |  |  |  |  |
| TTHY_HUMAN | P02766 | Transthyretin | 16 | 11 | 73.5% |  | 10 | 69.4% |  |  |  |  |
| TXND5_HUMAN | Q8NBS9 | Thioredoxin domain-containing protein 5 | 48 | 0 | 0% |  | 3 | 6.7% |  |  |  |  |
| TXNL1_HUMAN | O43396 | Thioredoxin-like protein 1 | 32 | 3 | 16.6% |  | 4 | 24.6% |  |  |  |  |
| UB2V1_HUMAN | Q13404 | Ubiquitin-conjugating enzyme E2 variant 1 | 16 | 1 | 6.8% |  | 4 | 38.1% |  |  |  |  |
| UBA1_HUMAN | P22314 | Ubiquitin-like modifier-activating enzyme 1 | 118 | 4 | 6.9% |  | 5 | 7.4% |  |  |  |  |
| UBE2N_HUMAN | P61088 | Ubiquitin-conjugating enzyme E2 N | 17 | 3 | 19.7% |  | 4 | 27% |  |  |  |  |
| UBP14_HUMAN | P54578 | Ubiquitin carboxyl-terminal hydrolase 14 | 56 | 2 | 4.3% |  | 2 | 5.3% |  |  |  |  |
| UCHL1_HUMAN | P09936 | Ubiquitin carboxyl-terminal hydrolase isozyme L1 | 25 | 0 | 0% |  | 3 | 13% |  |  |  |  |
| UFM1_HUMAN | P61960 | Ubiquitin-fold modifier 1 | 9 | 1 | 17.6% |  | 2 | 58.8% |  |  |  |  |
| UGGG1_HUMAN | Q9NYU2 | UDP-glucose:glycoprotein glucosyltransferase 1 | 177 | 0 | 0% |  | 4 | 3.5% |  |  |  |  |
| UGPA_HUMAN | Q16851 | UTP--glucose-1-phosphate uridylyltransferase | 57 | 4 | 13.2% |  | 9 | 24.8% |  |  |  |  |
| UK114_HUMAN | P52758 | Ribonuclease UK114 | 14 | 3 | 27% |  | 1 | 7.3% |  |  |  |  |
| UROK_HUMAN | P00749 | Urokinase-type plasminogen activator | 49 |  |  |  |  |  |  | + |  | + |
| VASN_HUMAN | Q6EMK4 | Vasorin | 72 | 1 | 2.4% |  | 2 | 6.5% |  |  |  |  |
| VAT1_HUMAN | Q99536 | Synaptic vesicle membrane protein VAT-1 homolog | 42 | 0 | 0% |  | 4 | 13.5% |  |  |  |  |
| VEGFA_HUMAN | P15692 | Vascular endothelial growth factor A | 27 |  |  |  |  |  |  | + |  | + |
| VEGFD_HUMAN | O43915 | Vascular endothelial growth factor D | 40 |  |  |  |  |  |  | + |  | + |
| VIME_HUMAN | P08670 | Vimentin | 54 | 27 | 57.3% |  | 43 | 78.1% |  |  |  |  |
| VINC_HUMAN | P18206 | Vinculin | 124 | 13 | 17.8% |  | 50 | 53.4% |  |  |  |  |
| VP26A_HUMAN | O75436 | Vacuolar protein sorting-associated protein 26A | 38 | 2 | 9.2% |  | 2 | 9.2% |  |  |  |  |
| VPS29_HUMAN | Q9UBQ0 | Vacuolar protein sorting-associated protein 29 | 21 | 2 | 12.6% |  | 1 | 7.1% |  |  |  |  |
| VPS35_HUMAN | Q96QK1 | Vacuolar protein sorting-associated protein 35 | 92 | 1 | 2.9% |  | 2 | 3.9% |  |  |  |  |
| VTDB_HUMAN | P02774 | Vitamin D-binding protein | 53 | 12 | 34.4% |  | 7 | 21.1% |  |  |  |  |
| VTNC_HUMAN | P04004 | Vitronectin | 54 | 2 | 6.3% |  | 2 | 6.3% |  |  |  |  |
| VWF_HUMAN | P04275 | von Willebrand factor | 309 | 21 | 10.4% |  | 23 | 12.3% |  |  |  |  |
| WFKN2_HUMAN | Q8TEU8 | WAP, Kazal, immunoglobulin, Kunitz and NTR domain-containing protein 2 | 64 |  |  |  |  |  |  | + |  | + |
| ZA2G_HUMAN | P25311 | Zinc-alpha-2-glycoprotein | 34 | 0 | 0% |  | 10 | 40.3% |  |  |  |  |
