# Supplementary material for: Secretome analysis of breast cancer-associated adipose tissue to identify paracrine regulators of breast cancer growth
Source: Oncotarget. 2017 May 3;8(29):47239–49. doi: 10.18632/oncotarget.17592 (PMC5564561; doi:10.18632/oncotarget.17592)
Supplement: Supplementary file 3 [file oncotarget-08-47239-s003.docx]

**Supplementary Table 2: Funrich analysis of the 668 detected proteins for biological processes and biological pathways.**

| **Funrich analysis of 668 proteins detected in CMCAAT by LC-MS/MS and antibody protein array** | | |
| --- | --- | --- |
|  |  |  |
|  |  |  |
| **Biological process** | **No. of genes  in the data set** | **Genes mapped from  input data set** |
| Metabolism | 119 | PGD,PGLS,GOT1,ACACB,DBI,ACY1,ACYP2,ADH1A,ADH1B,ADH5,ADIPOQ,AKR1A1,AKR1C1,AKR1C3,ALDH1L1,ALDH2,ALDOA,ALDOC,AKR1B1,AKR7A2,ASAH1,ATP5B,BLVRB,MTHFD1,CA1,CA2,CBR1,CD81,CP,CS,COX6B1,CYB5A,CYCS,DCXR,DDAH2,DECR1,QDPR,DYNC1H1,ECH1,ECHS1,ECI1,ENO1,ENO3,CES1,F13A1,FAH,FASN,FH,GAPDH,GPI,GCHFR,GLUL,GLRX,GNPDA1,GPD1,GPX3,GRHPR,GSTM3,GSTO1,GSTP1,GSTT1,HDHD2,ALAD,HIBCH,HINT2,EPHX1,IDH1,PPA1,AK1,AK2,AK3,CKB,LDHA,LDHB,GLO1,LIPE,PAFAH1B1,LYZ,MAT2B,MDH1,MDH2,MTAP,NAGK,CYB5R3,NIT2,NQO1,NUDT5,OPLAH,PDXK,PGAM1,PGK1,PGM1,PCBD1,BPGM,PRDX1,PRDX2,PRDX3,PRDX5,PRDX6,PTGR1,PAICS,PYGB,PYGL,CRYZ,AHCY,SOD1,SOD3,SPR,TALDO1,VCP,ACAT2,ACAT1,ACAA2,TXN,TKT,TPI1,TXNL1,UGGT1,UGP2 |
| Energy pathways | 115 | PGD,PGLS,GOT1,ACACB,DBI,ACY1,ACYP2,ADH1A,ADH1B,ADH5,ADIPOQ,AKR1C1,AKR1C3,ALDH1L1,ALDH2,ALDOA,ALDOC,AKR1B1,AKR7A2,ASAH1,ATP5B,BLVRB,MTHFD1,CA1,CA2,CBR1,CD81,CP,CS,COX6B1,CYB5A,CYCS,DCXR,DDAH2,DECR1,QDPR,DYNC1H1,ECH1,ECHS1,ECI1,ENO1,ENO3,F13A1,FAH,FASN,FH,GAPDH,GPI,GCHFR,GLUL,GLRX,GNPDA1,GPD1,GPX3,GRHPR,GSTM3,GSTO1,GSTP1,GSTT1,HDHD2,ALAD,HIBCH,HINT2,EPHX1,IDH1,PPA1,AK1,AK2,AK3,CKB,LDHA,LDHB,GLO1,LIPE,PAFAH1B1,LYZ,MDH1,MDH2,MTAP,NAGK,CYB5R3,NIT2,NQO1,NUDT5,OPLAH,PDXK,PGAM1,PGK1,PGM1,BPGM,PRDX1,PRDX2,PRDX3,PRDX5,PRDX6,PTGR1,PAICS,PYGB,PYGL,CRYZ,AHCY,SOD1,SOD3,SPR,TALDO1,VCP,ACAT2,ACAT1,ACAA2,TXN,TKT,TPI1,TXNL1,UGGT1,UGP2 |
| Protein metabolism | 95 | SERPINA1,A2M,LAP3,CTSC,CTSD,CTSZ,HSPE1,CNDP2,CPN2,CRYAB,COPS4,CSTB,EEF1G,EEF2,EIF3B,EIF3H,HSP90B1,ERAP1,FGA,FGB,FGG,FSTL1,HSP90AA1,HSPA1A,HSPA8,HSPB1,HSPB6,SERPING1,EIF4B,ITIH1,ITIH2,ITIH4,KLKB1,MMP1,MMP10,MMP2,MMP3,MMP7,MMP8,MMP9,NACA,OTUB1,SERPINE1,PDIA3,PDIA6,PLG,PPIB,PSMC5,NPEPPS,PSMA1,PSMA2,PSMA3,PSMA4,PSMA5,PSMA6,PSMA7,PSMB1,PSMB2,PSMB3,PSMB4,PSMB5,PSMB6,PSMD13,PSMD14,PSMD2,PSMD6,PSMD7,PSME1,PSME2,RPLP0,RPLP1,RPS25,RPS27A,RPS3,APCS,SELENBP1,SCRN2,SGTA,SERPINB6,SNCG,TBCA,CCT6A,F3,F2,TPSAB1,TXNDC5,-,UBE2N,USP14,UCHL1,HRSP12,PLAU,VPS29,VWF,WFIKKN2 |
| Cell growth and/or maintenance | 82 | ACTB,ACTN1,ACTN4,ACTA1,ACTR1A,AFM,AKAP12,ACTR3,ARPC2,ARPC3,CAP1,CAPG,CAPZB,CAV1,CAPZA1,CAPZA2,CLTC,COL1A1,COL6A1,COL6A3,COL14A1,CFL1,COL15A1,COL18A1,CORO1C,COTL1,CHRDL2,DPT,DYNLL2,FBLN1,FBLN2,FERMT2,FHL1,FN1,FLNA,FLNB,ARHGDIA,GSN,KRT10,KRT9,KRT2,KRT1,LAMA2,LAMA4,LAMB1,LAMB2,LAMC1,LMNA,LMNB2,LRP6,LUM,MAP4,MSN,MYH9,MYL6,MYO1C,NID1,NID2,NUMA1,PARVA,HSPG2,PFN1,S100A4,SDPR,SPARC,SPTBN1,SPTAN1,TUBA1B,TUBB,TNXB,TIMP1,TLN1,TLN2,TPM3,TPM4,TNFAIP6,THBS1,THBS2,THBS4,VIM,VCL,VTN |
| Immune response | 51 | ORM1,ORM2,AMBP,AOC3,ATRN,B2M,C1QA,C1QB,C1QC,C1R,C1S,CCL2,CCL27,CCL3,CCL4L1,CD5L,CFB,CFD,CFH,CFI,CLU,C3,-,C5,C7,C8G,C9,CSF1,CXCL2,CXCL9,DDT,CXCL1,HP,HPR,IL36RN,IGJ,IL17D,IL17F,IL19,IL22,IL26,IL36A,IL37,IL6,CXCL8,LBP,LGALS1,LGALS3BP,PIGR,TNFSF8,AZGP1 |
| Cell proliferation | 4 | CSF1,PPP1CA,PTMA,SOD2 |
| Inflammatory response | 2 | IL36RN,LTB |
| Signal transduction | 149 | YWHAE,YWHAG,YWHAQ,ANGPTL1,ANGPT1,ANGPT2,ANXA1,ANXA2,ANXA4,ANXA5,ARF3,ACVR2A,BDNF,BMP4,BMP8B,BMPR1B,BPNT1,CD163,CALB2,CALM1,CCL22,CCR4,CCR7,CCR8,CDKN2C,CNTF,C19orf10,CSF1,CSPG4,CXCR1,CXCR2,CXCR4,CXCR5,CXCR6,CXCL11,CXCL14,DIABLO,DKK4,DUSP3,EEF1D,EHD2,ELTD1,EPO,EPS15,ERBB4,ESYT1,FABP4,AHSG,FGF11,FGF16,FGF2,FGF20,FGF23,FGF9,FGFR4,FKBP1A,FST,GNB1,GNB2,GNB2L1,GDF15,ARHGDIB,GFRA2,GCG,GNAI1,GNAI2,GPC5,GPNMB,HBEGF,HSP90AB1,IL17RD,IGFBP3,IGFBP6,IGFBP7,ICAM5,IFNL1,IGF2,IL17B,IL17C,IL1R1,IL37,INHBB,IQGAP1,PRKAR2B,LCK,LEPR,LEFTY2,LIFR,MFGE8,MIF,OGN,MRC1,MCAM,NRG1,NRN1,NRTN,OSM,HCRT,PTGDR2,PDCD6,PEA15,PEBP1,SERPINF1,PGRMC2,PRKCDBP,PTPN11,PPP2R4,PTPRS,PLXDC2,RAB1A,RAB2A,RAB5C,RAP1A,RHOA,ROBO4,RRAS2,RPSA,RSU1,RTN1,S100A1,S100A6,S100A8,S100A9,S100A10,S100A11,S100A13,S100A16,PSAP,SEC22B,SEPT2,SH3BGRL,SIGIRR,SIGLEC9,SRI,SPARCL1,STOM,TNS1,TGFB2,TGFBR1,LTB,TNFRSF11A,TNFRSF18,THPO,TNFRSF10B,TNFRSF11B,TRADD,VASN,VEGFA,FIGF |
| Peptide metabolism | 1 | PPIA |
| Aldehyde metabolism | 1 | ALDH1A1 |
| Purine salvage | 1 | APRT |
| Cell communication | 146 | YWHAE,YWHAG,YWHAQ,ANGPTL1,ANGPT1,ANGPT2,ANXA1,ANXA2,ANXA4,ANXA5,ARF3,ACVR2A,BDNF,BMP4,BMP8B,BMPR1B,BPNT1,CD163,CALB2,CALM1,CCL22,CCR4,CCR7,CCR8,CDKN2C,CNTF,C19orf10,CSPG4,CXCR1,CXCR2,CXCR4,CXCR5,CXCR6,CXCL11,CXCL14,DIABLO,DKK4,DUSP3,EEF1D,EHD2,ELTD1,EPO,EPS15,ERBB4,ESYT1,FABP4,AHSG,FGF11,FGF16,FGF2,FGF20,FGF23,FGF9,FGFR4,FKBP1A,FST,GNB1,GNB2,GNB2L1,GDF15,ARHGDIB,GFRA2,GCG,GNAI1,GNAI2,GPC5,GPNMB,HBEGF,HSP90AB1,IL17RD,IGFBP3,IGFBP6,IGFBP7,ICAM5,IFNL1,IGF2,IL17B,IL17C,IL1R1,IL37,INHBB,IQGAP1,PRKAR2B,LCK,LEPR,LEFTY2,LIFR,MFGE8,MIF,MRC1,MCAM,NRG1,NRN1,NRTN,OSM,HCRT,PTGDR2,PDCD6,PEA15,PEBP1,SERPINF1,PGRMC2,PRKCDBP,PTPN11,PPP2R4,PTPRS,PLXDC2,RAB1A,RAB2A,RAB5C,RAP1A,RHOA,ROBO4,RRAS2,RPSA,RTN1,S100A1,S100A6,S100A8,S100A9,S100A10,S100A11,S100A13,S100A16,PSAP,SEC22B,SEPT2,SH3BGRL,SIGIRR,SIGLEC9,SRI,SPARCL1,STOM,TNS1,TGFB2,TGFBR1,LTB,TNFRSF11A,TNFRSF18,THPO,TNFRSF10B,TNFRSF11B,TRADD,VASN,VEGFA,FIGF |
| Muscle development | 1 | TAGLN |
| G-protein coupled receptor protein signaling pathway | 1 | CCR9 |
| Lipid storage | 1 | PLIN1 |
| Anti-apoptosis | 2 | SOD2,TNFRSF18 |
| Cell growth and/or maintenance | 2 | NRG2,SOD2 |
| Cytoskeleton organization and biogenesis | 2 | ARPC2,CFL2 |
| Amino acid and derivative metabolism | 1 | HNMT |
| Xenobiotic metabolism | 1 | NQO1 |
| Protein folding | 2 | ERP29,PPIA |
| Regulation of signal transduction | 2 | COPS8,KREMEN2 |
| Cell proliferation | 2 | IL15RA,IL4R |
| Chromosome organization and biogenesis | 1 | HIST1H2BD |
| Hemopoiesis | 1 | CSF1 |
| Synaptic transmission | 1 | NQO1 |
| Cellular defense response | 1 | CCR9 |
| Cytoskeletal anchoring | 1 | PLEC |
| Protein targeting | 1 | VPS29 |
| Muscle contraction | 1 | AHNAK |
| Regulation of cell cycle | 3 | YWHAZ,CDKN2C,PPP2R4 |
| Organogenesis | 1 | OGN |
| Cytoskeleton organization and biogenesis | 1 | SORBS1 |
| Cell adhesion | 1 | RPSA |
| Cell communication | 8 | ANXA2,CD163,CALB2,EEF1D,IL4R,LEPR,OGN,STOM |
| Apoptosis | 8 | AIFM2,PYCARD,BAX,DIABLO,FIS1,GRB2,HRG,TNFRSF10B |
| Regulation of nucleobase, nucleoside, nucleotide and nucleic acid metabolism | 1 | NME2 |
| Regulation of cell cycle | 1 | GRB2 |
| Regulation of cell growth | 1 | RTN4 |
| Metabolism | 1 | PRKCSH |
| Immune response | 1 | IL4R |
| Cell differentiation | 1 | CSF1 |
| Proteolysis and peptidolysis | 1 | UBA1 |
| Transport | 42 | ALB,AP1B1,APOA1,APOA2,APOB,APOC3,APOD,APOE,APOH,CLIC1,CLIC4,EEA1,FABP5,AFP,FTH1,FTL,GDI2,SLC2A2,SLC2A5,-HBB,HBD,HBG1,HPX,KPNB1,KCTD12,LCN1,PITPNB,PLIN3,PLTP,RBP4,RRBP1,SAA4,NAPA,STX7,TF,LTF,TTR,VAT1,VPS26A,VPS35,GC |
| Cell cycle | 1 | SEPT2 |
| Lipid metabolism | 1 | MGLL |
| Energy pathways | 1 | PRKCSH |
| Signal transduction | 13 | YWHAB,ANXA2,CD163,CALB2,EEF1D,GRB2,IL15RA,IL4R,LEPR,NME2,NRG2,SORBS1,STOM |
| Apoptosis | 1 | IGFBP3 |
| Ion transport | 1 | ATP5D |
| Cell adhesion | 1 | RSU1 |
| Regulation of nucleobase, nucleoside, nucleotide and nucleic acid metabolism | 20 | ACO1,ADK,CAND1,COPS7A,HIST2H2AC,-,-,HNRNPC,HYI,LGALS3,MATR3,PARK7,PCBP1,PTBP1,PTRF,RNH1,RNASE4,SNRPD1,SRSF7,TSNAX |
| Biological_process unknown | 38 | A1BG,LRG1,ABHD14B,ACOT1,ADIRF,ANP32B,PHYKPL,CD248,C11orf54,CFL2,CPPED1,CUTA,DYNLRB1,ECHDC1,FAM49B,GLIPR2,GGCT,GLOD4,GPD1L,GPNMB,HADH,HEBP1,HEBP2,ISOC1,LHPP,LIF,APOA1BP,PIP,PLIN4,PROK1,PTER,REEP6,SH3BGRL3,TAGLN2,TBC1D9B,CLEC3B,RELT,UFM1 |

| **Funrich analysis of 668 proteins detected in CMCAAT by LC-MS/MS and antibody protein array** | | |
| --- | --- | --- |
|  |  |  |
|  |  |  |
| **Biological pathway** | **No. of genes  in the data set** | **Genes mapped from  input data set** |
| Destabilization of mRNA by AUF1 (hnRNP D0) | 25 | HSPA1A,HSPA8,HSPB1,PSMC5,PSMA1,PSMA2,PSMA3,PSMA4,PSMA5,PSMA6,PSMA7,PSMB1,PSMB2,PSMB3,PSMB4,PSMB5,PSMB6,PSMD13,PSMD14,PSMD2,PSMD6,PSMD7,PSME1,PSME2,RPS27A |
| Autodegradation of Cdh1 by Cdh1:APC/C | 22 | PSMC5,PSMA1,PSMA2,PSMA3,PSMA4,PSMA5,PSMA6,PSMA7,PSMB1,PSMB2,PSMB3,PSMB4,PSMB5,PSMB6,PSMD13,PSMD14,PSMD2,PSMD6,PSMD7,PSME1,PSME2,RPS27A |
| Antigen processing: Ubiquitination & Proteasome degradation | 23 | PSMC5,NPEPPS,PSMA1,PSMA2,PSMA3,PSMA4,PSMA5,PSMA6,PSMA7,PSMB1,PSMB2,PSMB3,PSMB4,PSMB5,PSMB6,PSMD13,PSMD14,PSMD2,PSMD6,PSMD7,PSME1,PSME2,RPS27A |
| Complement cascade | 13 | C1QA,C1QB,C1QC,C1R,C1S,CFB,CFD,C3,-,C5,C7,C8G,C9 |
| Regulation of ornithine decarboxylase (ODC) | 23 | NQO1,PSMC5,PSMA1,PSMA2,PSMA3,PSMA4,PSMA5,PSMA6,PSMA7,PSMB1,PSMB2,PSMB3,PSMB4,PSMB5,PSMB6,PSMD13,PSMD14,PSMD2,PSMD6,PSMD7,PSME1,PSME2,RPS27A |
| Ubiquitin-dependent degradation of Cyclin D1 | 22 | PSMC5,PSMA1,PSMA2,PSMA3,PSMA4,PSMA5,PSMA6,PSMA7,PSMB1,PSMB2,PSMB3,PSMB4,PSMB5,PSMB6,PSMD13,PSMD14,PSMD2,PSMD6,PSMD7,PSME1,PSME2,RPS27A |
| APC/C:Cdc20 mediated degradation of Securin | 22 | PSMC5,PSMA1,PSMA2,PSMA3,PSMA4,PSMA5,PSMA6,PSMA7,PSMB1,PSMB2,PSMB3,PSMB4,PSMB5,PSMB6,PSMD13,PSMD14,PSMD2,PSMD6,PSMD7,PSME1,PSME2,RPS27A |
| Ubiquitin-dependent degradation of Cyclin D | 22 | PSMC5,PSMA1,PSMA2,PSMA3,PSMA4,PSMA5,PSMA6,PSMA7,PSMB1,PSMB2,PSMB3,PSMB4,PSMB5,PSMB6,PSMD13,PSMD14,PSMD2,PSMD6,PSMD7,PSME1,PSME2,RPS27A |
| CDK-mediated phosphorylation and removal of Cdc6 | 22 | PSMC5,PSMA1,PSMA2,PSMA3,PSMA4,PSMA5,PSMA6,PSMA7,PSMB1,PSMB2,PSMB3,PSMB4,PSMB5,PSMB6,PSMD13,PSMD14,PSMD2,PSMD6,PSMD7,PSME1,PSME2,RPS27A |
| Regulation of activated PAK-2p34 by proteasome mediated degradation | 22 | PSMC5,PSMA1,PSMA2,PSMA3,PSMA4,PSMA5,PSMA6,PSMA7,PSMB1,PSMB2,PSMB3,PSMB4,PSMB5,PSMB6,PSMD13,PSMD14,PSMD2,PSMD6,PSMD7,PSME1,PSME2,RPS27A |
| Cross-presentation of soluble exogenous antigens (endosomes) | 22 | PSMC5,PSMA1,PSMA2,PSMA3,PSMA4,PSMA5,PSMA6,PSMA7,PSMB1,PSMB2,PSMB3,PSMB4,PSMB5,PSMB6,PSMD13,PSMD14,PSMD2,PSMD6,PSMD7,PSME1,PSME2,RPS27A |
| SCF(Skp2)-mediated degradation of p27/p21 | 22 | PSMC5,PSMA1,PSMA2,PSMA3,PSMA4,PSMA5,PSMA6,PSMA7,PSMB1,PSMB2,PSMB3,PSMB4,PSMB5,PSMB6,PSMD13,PSMD14,PSMD2,PSMD6,PSMD7,PSME1,PSME2,RPS27A |
| Vpu mediated degradation of CD4 | 22 | PSMC5,PSMA1,PSMA2,PSMA3,PSMA4,PSMA5,PSMA6,PSMA7,PSMB1,PSMB2,PSMB3,PSMB4,PSMB5,PSMB6,PSMD13,PSMD14,PSMD2,PSMD6,PSMD7,PSME1,PSME2,RPS27A |
| Apoptosis | 35 | YWHAB,BAX,CYCS,DIABLO,DYNLL2,GSN,KPNB1,LMNA,PLEC,PSMC5,PSMA1,PSMA2,PSMA3,PSMA4,PSMA5,PSMA6,PSMA7,PSMB1,PSMB2,PSMB3,PSMB4,PSMB5,PSMB6,PSMD13,PSMD14,PSMD2,PSMD6,PSMD7,PSME1,PSME2,RPS27A,SPTAN1,TNFRSF10B,TRADD,VIM |
| ER-Phagosome pathway | 24 | B2M,PDIA3,PSMC5,PSMA1,PSMA2,PSMA3,PSMA4,PSMA5,PSMA6,PSMA7,PSMB1,PSMB2,PSMB3,PSMB4,PSMB5,PSMB6,PSMD13,PSMD14,PSMD2,PSMD6,PSMD7,PSME1,PSME2,RPS27A |
| Autodegradation of the E3 ubiquitin ligase COP1 | 22 | PSMC5,PSMA1,PSMA2,PSMA3,PSMA4,PSMA5,PSMA6,PSMA7,PSMB1,PSMB2,PSMB3,PSMB4,PSMB5,PSMB6,PSMD13,PSMD14,PSMD2,PSMD6,PSMD7,PSME1,PSME2,RPS27A |
| Cdc20:Phospho-APC/C mediated degradation of Cyclin A | 22 | PSMC5,PSMA1,PSMA2,PSMA3,PSMA4,PSMA5,PSMA6,PSMA7,PSMB1,PSMB2,PSMB3,PSMB4,PSMB5,PSMB6,PSMD13,PSMD14,PSMD2,PSMD6,PSMD7,PSME1,PSME2,RPS27A |
| Stabilization of p53 | 22 | PSMC5,PSMA1,PSMA2,PSMA3,PSMA4,PSMA5,PSMA6,PSMA7,PSMB1,PSMB2,PSMB3,PSMB4,PSMB5,PSMB6,PSMD13,PSMD14,PSMD2,PSMD6,PSMD7,PSME1,PSME2,RPS27A |
| Vif-mediated degradation of APOBEC3G | 22 | PSMC5,PSMA1,PSMA2,PSMA3,PSMA4,PSMA5,PSMA6,PSMA7,PSMB1,PSMB2,PSMB3,PSMB4,PSMB5,PSMB6,PSMD13,PSMD14,PSMD2,PSMD6,PSMD7,PSME1,PSME2,RPS27A |
| Ubiquitin Mediated Degradation of Phosphorylated Cdc25A | 22 | PSMC5,PSMA1,PSMA2,PSMA3,PSMA4,PSMA5,PSMA6,PSMA7,PSMB1,PSMB2,PSMB3,PSMB4,PSMB5,PSMB6,PSMD13,PSMD14,PSMD2,PSMD6,PSMD7,PSME1,PSME2,RPS27A |
| APC/C:Cdh1 mediated degradation of Cdc20 and other APC/C:Cdh1 targeted proteins in late mitosis/early G1 | 22 | PSMC5,PSMA1,PSMA2,PSMA3,PSMA4,PSMA5,PSMA6,PSMA7,PSMB1,PSMB2,PSMB3,PSMB4,PSMB5,PSMB6,PSMD13,PSMD14,PSMD2,PSMD6,PSMD7,PSME1,PSME2,RPS27A |
| p53-Independent G1/S DNA damage checkpoint | 22 | PSMC5,PSMA1,PSMA2,PSMA3,PSMA4,PSMA5,PSMA6,PSMA7,PSMB1,PSMB2,PSMB3,PSMB4,PSMB5,PSMB6,PSMD13,PSMD14,PSMD2,PSMD6,PSMD7,PSME1,PSME2,RPS27A |
| p53-Independent DNA Damage Response | 22 | PSMC5,PSMA1,PSMA2,PSMA3,PSMA4,PSMA5,PSMA6,PSMA7,PSMB1,PSMB2,PSMB3,PSMB4,PSMB5,PSMB6,PSMD13,PSMD14,PSMD2,PSMD6,PSMD7,PSME1,PSME2,RPS27A |
| SCF-beta-TrCP mediated degradation of Emi1 | 22 | PSMC5,PSMA1,PSMA2,PSMA3,PSMA4,PSMA5,PSMA6,PSMA7,PSMB1,PSMB2,PSMB3,PSMB4,PSMB5,PSMB6,PSMD13,PSMD14,PSMD2,PSMD6,PSMD7,PSME1,PSME2,RPS27A |
| Regulation of mRNA Stability by Proteins that Bind AU-rich Elements | 27 | YWHAB,YWHAZ,HSPA1A,HSPA8,HSPB1,PSMC5,PSMA1,PSMA2,PSMA3,PSMA4,PSMA5,PSMA6,PSMA7,PSMB1,PSMB2,PSMB3,PSMB4,PSMB5,PSMB6,PSMD13,PSMD14,PSMD2,PSMD6,PSMD7,PSME1,PSME2,RPS27A |
| APC/C:Cdc20 mediated degradation of mitotic proteins | 22 | PSMC5,PSMA1,PSMA2,PSMA3,PSMA4,PSMA5,PSMA6,PSMA7,PSMB1,PSMB2,PSMB3,PSMB4,PSMB5,PSMB6,PSMD13,PSMD14,PSMD2,PSMD6,PSMD7,PSME1,PSME2,RPS27A |
| p53-Dependent G1 DNA Damage Response | 22 | PSMC5,PSMA1,PSMA2,PSMA3,PSMA4,PSMA5,PSMA6,PSMA7,PSMB1,PSMB2,PSMB3,PSMB4,PSMB5,PSMB6,PSMD13,PSMD14,PSMD2,PSMD6,PSMD7,PSME1,PSME2,RPS27A |
| Activation of APC/C and APC/C:Cdc20 mediated degradation of mitotic proteins | 22 | PSMC5,PSMA1,PSMA2,PSMA3,PSMA4,PSMA5,PSMA6,PSMA7,PSMB1,PSMB2,PSMB3,PSMB4,PSMB5,PSMB6,PSMD13,PSMD14,PSMD2,PSMD6,PSMD7,PSME1,PSME2,RPS27A |
| p53-Dependent G1/S DNA damage checkpoint | 22 | PSMC5,PSMA1,PSMA2,PSMA3,PSMA4,PSMA5,PSMA6,PSMA7,PSMB1,PSMB2,PSMB3,PSMB4,PSMB5,PSMB6,PSMD13,PSMD14,PSMD2,PSMD6,PSMD7,PSME1,PSME2,RPS27A |
| Antigen processing-Cross presentation | 24 | B2M,PDIA3,PSMC5,PSMA1,PSMA2,PSMA3,PSMA4,PSMA5,PSMA6,PSMA7,PSMB1,PSMB2,PSMB3,PSMB4,PSMB5,PSMB6,PSMD13,PSMD14,PSMD2,PSMD6,PSMD7,PSME1,PSME2,RPS27A |
| CDT1 association with the CDC6:ORC:origin complex | 22 | PSMC5,PSMA1,PSMA2,PSMA3,PSMA4,PSMA5,PSMA6,PSMA7,PSMB1,PSMB2,PSMB3,PSMB4,PSMB5,PSMB6,PSMD13,PSMD14,PSMD2,PSMD6,PSMD7,PSME1,PSME2,RPS27A |
| Class I MHC mediated antigen processing & presentation | 26 | B2M,ERAP1,PDIA3,PSMC5,NPEPPS,PSMA1,PSMA2,PSMA3,PSMA4,PSMA5,PSMA6,PSMA7,PSMB1,PSMB2,PSMB3,PSMB4,PSMB5,PSMB6,PSMD13,PSMD14,PSMD2,PSMD6,PSMD7,PSME1,PSME2,RPS27A |
| Degradation of beta-catenin by the destruction complex | 22 | PSMC5,PSMA1,PSMA2,PSMA3,PSMA4,PSMA5,PSMA6,PSMA7,PSMB1,PSMB2,PSMB3,PSMB4,PSMB5,PSMB6,PSMD13,PSMD14,PSMD2,PSMD6,PSMD7,PSME1,PSME2,RPS27A |
| Signaling by Wnt | 22 | PSMC5,PSMA1,PSMA2,PSMA3,PSMA4,PSMA5,PSMA6,PSMA7,PSMB1,PSMB2,PSMB3,PSMB4,PSMB5,PSMB6,PSMD13,PSMD14,PSMD2,PSMD6,PSMD7,PSME1,PSME2,RPS27A |
| Regulation of Apoptosis | 22 | PSMC5,PSMA1,PSMA2,PSMA3,PSMA4,PSMA5,PSMA6,PSMA7,PSMB1,PSMB2,PSMB3,PSMB4,PSMB5,PSMB6,PSMD13,PSMD14,PSMD2,PSMD6,PSMD7,PSME1,PSME2,RPS27A |
| G1/S DNA Damage Checkpoints | 22 | PSMC5,PSMA1,PSMA2,PSMA3,PSMA4,PSMA5,PSMA6,PSMA7,PSMB1,PSMB2,PSMB3,PSMB4,PSMB5,PSMB6,PSMD13,PSMD14,PSMD2,PSMD6,PSMD7,PSME1,PSME2,RPS27A |
| Cyclin E associated events during G1/S transition | 22 | PSMC5,PSMA1,PSMA2,PSMA3,PSMA4,PSMA5,PSMA6,PSMA7,PSMB1,PSMB2,PSMB3,PSMB4,PSMB5,PSMB6,PSMD13,PSMD14,PSMD2,PSMD6,PSMD7,PSME1,PSME2,RPS27A |
| Regulation of APC/C activators between G1/S and early anaphase | 22 | PSMC5,PSMA1,PSMA2,PSMA3,PSMA4,PSMA5,PSMA6,PSMA7,PSMB1,PSMB2,PSMB3,PSMB4,PSMB5,PSMB6,PSMD13,PSMD14,PSMD2,PSMD6,PSMD7,PSME1,PSME2,RPS27A |
| Hemostasis | 55 | YWHAZ,A2M,ANGPT1,ANGPT2,APOA1,APOB,CALM1,CAP1,CAPZB,CAV1,CAPZA1,CAPZA2,COL1A1,CFL1,EHD2,F13A1,FGA,FGB,FGG,FN1,FLNA,GNB1,GNAI1,GNAI2,GRB2,-,HBB,HBD,HBG1,HRG,IGFBP3,SERPING1,AK3,PRKAR2B,KLKB1,LCK,MGLL,MMP1,SERPINE1,PLG,PPIA,PFN1,PTPN11,RAP1A,RHOA,PSAP,SOD1,F3,F2,TLN1,THPO,TF,PLAU,VCL,VWF |
| Regulation of mitotic cell cycle | 22 | PSMC5,PSMA1,PSMA2,PSMA3,PSMA4,PSMA5,PSMA6,PSMA7,PSMB1,PSMB2,PSMB3,PSMB4,PSMB5,PSMB6,PSMD13,PSMD14,PSMD2,PSMD6,PSMD7,PSME1,PSME2,RPS27A |
| APC/C-mediated degradation of cell cycle proteins | 22 | PSMC5,PSMA1,PSMA2,PSMA3,PSMA4,PSMA5,PSMA6,PSMA7,PSMB1,PSMB2,PSMB3,PSMB4,PSMB5,PSMB6,PSMD13,PSMD14,PSMD2,PSMD6,PSMD7,PSME1,PSME2,RPS27A |
| Assembly of the pre-replicative complex | 22 | PSMC5,PSMA1,PSMA2,PSMA3,PSMA4,PSMA5,PSMA6,PSMA7,PSMB1,PSMB2,PSMB3,PSMB4,PSMB5,PSMB6,PSMD13,PSMD14,PSMD2,PSMD6,PSMD7,PSME1,PSME2,RPS27A |
| Switching of origins to a post-replicative state | 22 | PSMC5,PSMA1,PSMA2,PSMA3,PSMA4,PSMA5,PSMA6,PSMA7,PSMB1,PSMB2,PSMB3,PSMB4,PSMB5,PSMB6,PSMD13,PSMD14,PSMD2,PSMD6,PSMD7,PSME1,PSME2,RPS27A |
| Orc1 removal from chromatin | 22 | PSMC5,PSMA1,PSMA2,PSMA3,PSMA4,PSMA5,PSMA6,PSMA7,PSMB1,PSMB2,PSMB3,PSMB4,PSMB5,PSMB6,PSMD13,PSMD14,PSMD2,PSMD6,PSMD7,PSME1,PSME2,RPS27A |
| Removal of licensing factors from origins | 22 | PSMC5,PSMA1,PSMA2,PSMA3,PSMA4,PSMA5,PSMA6,PSMA7,PSMB1,PSMB2,PSMB3,PSMB4,PSMB5,PSMB6,PSMD13,PSMD14,PSMD2,PSMD6,PSMD7,PSME1,PSME2,RPS27A |
| Initial triggering of complement | 9 | C1QA,C1QB,C1QC,C1R,C1S,CFB,CFD,C3,- |
| Regulation of DNA replication | 22 | PSMC5,PSMA1,PSMA2,PSMA3,PSMA4,PSMA5,PSMA6,PSMA7,PSMB1,PSMB2,PSMB3,PSMB4,PSMB5,PSMB6,PSMD13,PSMD14,PSMD2,PSMD6,PSMD7,PSME1,PSME2,RPS27A |
| Platelet degranulation | 12 | APOA1,CALM1,CAP1,CFL1,FLNA,PPIA,PFN1,PSAP,SOD1,TLN1,TF,VCL |
| Cyclin A:Cdk2-associated events at S phase entry | 22 | PSMC5,PSMA1,PSMA2,PSMA3,PSMA4,PSMA5,PSMA6,PSMA7,PSMB1,PSMB2,PSMB3,PSMB4,PSMB5,PSMB6,PSMD13,PSMD14,PSMD2,PSMD6,PSMD7,PSME1,PSME2,RPS27A |
| "Lipid digestion, mobilization, and transport" | 15 | A2M,ALB,APOA1,APOA2,APOB,APOC3,APOE,CAV1,FABP4,LIPE,MGLL,HSPG2,PLIN1,PLTP,PPP1CA |
| Metabolism of amino acids and derivatives | 33 | GOT1,QDPR,FAH,GLUL,GRHPR,HIBCH,INHBB,MTAP,NQO1,PCBD1,PSMC5,PSMA1,PSMA2,PSMA3,PSMA4,PSMA5,PSMA6,PSMA7,PSMB1,PSMB2,PSMB3,PSMB4,PSMB5,PSMB6,PSMD13,PSMD14,PSMD2,PSMD6,PSMD7,PSME1,PSME2,RPS27A,ACAT1 |
| Adaptive Immune System | 38 | YWHAB,YWHAZ,B2M,CD81,C3,ERAP1,GRB2,LCK,PDIA3,PSMC5,NPEPPS,PSMA1,PSMA2,PSMA3,PSMA4,PSMA5,PSMA6,PSMA7,PSMB1,PSMB2,PSMB3,PSMB4,PSMB5,PSMB6,PSMD13,PSMD14,PSMD2,PSMD6,PSMD7,PSME1,PSME2,PTPN11,RAP1A,RPS27A,SPTBN1,SPTAN1,-,UBE2N |
| DNA Replication Pre-Initiation | 22 | PSMC5,PSMA1,PSMA2,PSMA3,PSMA4,PSMA5,PSMA6,PSMA7,PSMB1,PSMB2,PSMB3,PSMB4,PSMB5,PSMB6,PSMD13,PSMD14,PSMD2,PSMD6,PSMD7,PSME1,PSME2,RPS27A |
| M/G1 Transition | 22 | PSMC5,PSMA1,PSMA2,PSMA3,PSMA4,PSMA5,PSMA6,PSMA7,PSMB1,PSMB2,PSMB3,PSMB4,PSMB5,PSMB6,PSMD13,PSMD14,PSMD2,PSMD6,PSMD7,PSME1,PSME2,RPS27A |
| Host Interactions of HIV factors | 27 | AP1B1,B2M,KPNB1,LCK,PPIA,PSMC5,PSMA1,PSMA2,PSMA3,PSMA4,PSMA5,PSMA6,PSMA7,PSMB1,PSMB2,PSMB3,PSMB4,PSMB5,PSMB6,PSMD13,PSMD14,PSMD2,PSMD6,PSMD7,PSME1,PSME2,RPS27A |
| Chemokine receptors bind chemokines | 13 | CCL2,CCL22,CCL27,CCL3,CCR4,CCR7,CCR9,CXCL9,CXCR1,CXCR2,CXCR5,CXCR6,CXCL11 |
| Immune System | 63 | YWHAB,YWHAZ,PYCARD,B2M,C1QA,C1QB,C1QC,C1R,C1S,CAPZA1,CAPZA2,CD81,CFB,CFD,C3,-,C5,C7,C8G,C9,EEA1,ERAP1,PRKCSH,GRB2,HSP90AB1,IL1R1,IL6,LBP,LCK,LGALS3,PDIA3,PSMC5,NPEPPS,PSMA1,PSMA2,PSMA3,PSMA4,PSMA5,PSMA6,PSMA7,PSMB1,PSMB2,PSMB3,PSMB4,PSMB5,PSMB6,PSMD13,PSMD14,PSMD2,PSMD6,PSMD7,PSME1,PSME2,PTPN11,RAP1A,RPS27A,SIGIRR,SPTBN1,SPTAN1,TXN,TLN1,-,UBE2N |
| Metabolism | 88 | YWHAB,YWHAZ,GOT1,ADK,ALDH1A1,ALDH2,APRT,ATP5B,ATP5D,BPNT1,MTHFD1,CALM1,CAV1,CS,CNDP2,COX6B1,CYB5A,CYCS,QDPR,FAH,FASN,FH,GNB1,GNB2,GCHFR,GGCT,GLUL,GLRX,GCG,GRHPR,GSTO1,GSTP1,ALAD,HIBCH,HSP90AA1,HSPA1A,HSPA8,HSPB1,EIF4B,INHBB,IQGAP1,AK1,AK2,PRKAR2B,MDH2,MTAP,CYB5R3,NQO1,OPLAH,PDXK,PCBD1,PSMC5,PSMA1,PSMA2,PSMA3,PSMA4,PSMA5,PSMA6,PSMA7,PSMB1,PSMB2,PSMB3,PSMB4,PSMB5,PSMB6,PSMD13,PSMD14,PSMD2,PSMD6,PSMD7,PSME1,PSME2,PAICS,RAP1A,RPLP0,RPLP1,RPS25,RPS27A,RPS3,RPSA,AHCY,SNRPD1,SPR,TALDO1,ACAT1,TXN,TKT,UGP2 |
| Synthesis of DNA | 22 | PSMC5,PSMA1,PSMA2,PSMA3,PSMA4,PSMA5,PSMA6,PSMA7,PSMB1,PSMB2,PSMB3,PSMB4,PSMB5,PSMB6,PSMD13,PSMD14,PSMD2,PSMD6,PSMD7,PSME1,PSME2,RPS27A |
| Response to elevated platelet cytosolic Ca2+ | 12 | APOA1,CALM1,CAP1,CFL1,FLNA,PPIA,PFN1,PSAP,SOD1,TLN1,TF,VCL |
| "Platelet activation, signaling and aggregation" | 25 | YWHAZ,APOA1,CALM1,CAP1,CFL1,FLNA,GNB1,GNAI1,GNAI2,GRB2,IGFBP3,LCK,MGLL,PPIA,PFN1,RAP1A,RHOA,PSAP,SOD1,F2,TLN1,THPO,TF,VCL,VWF |
| Beta1 integrin cell surface interactions | 129 | YWHAB,YWHAE,YWHAG,YWHAQ,YWHAZ,A2M,ACTN1,ACTN4,ACTA1,AIFM2,ALDOA,ACTR3,ARPC2,ARPC3,PYCARD,B2M,BAX,BDNF,BMP4,BMPR1B,CALM1,CTSD,CAV1,CCL2,CD81,CP,CLTC,CLU,COL1A1,COL6A1,COL6A3,COL18A1,CSF1,CSPG4,CXCR4,CYCS,DKK4,DYNLRB1,EEF2,ENO1,HSP90B1,EPO,EPS15,F13A1,FABP4,AFP,AHSG,FGF23,FGFR4,FGA,FGB,FGG,FN1,FKBP1A,FTH1,FST,GAPDH,GDF15,ARHGDIA,GSN,GNAI1,GNAI2,GRB2,HSP90AA1,HSPA1A,HSPA8,HSPB1,IL17RD,IGFBP3,EIF4B,IGF2,IL4R,IL6,CXCL8,KPNB1,IQGAP1,KRT1,LAMA2,LAMA4,LAMB1,LAMB2,LAMC1,LBP,LCK,LDHA,LGALS1,LIF,LRP6,MMP1,MMP2,MMP3,MMP7,MMP9,NME2,NID1,OSM,SERPINE1,PEBP1,PGK1,PGM1,PIGR,PLG,PPP1CA,PRDX1,PRDX3,PTMA,PTPN11,PPP2R4,RAP1A,RHOA,SOD2,SPTBN1,SORBS1,TAGLN,TGFB2,TGFBR1,TXN,TIMP1,TLN1,TNFRSF18,TNFRSF10B,TF,THBS1,THBS2,PLAU,VEGFA,FIGF,VCL,VTN |
| Formation of Fibrin Clot (Clotting Cascade) | 11 | A2M,F13A1,FGA,FGB,FGG,IGFBP3,SERPING1,KLKB1,F3,F2,VWF |
| Integrin family cell surface interactions | 130 | YWHAB,YWHAE,YWHAG,YWHAQ,YWHAZ,A2M,ACTN1,ACTN4,ACTA1,AIFM2,ALDOA,ACTR3,ARPC2,ARPC3,PYCARD,B2M,BAX,BDNF,BMP4,BMPR1B,CALM1,CTSD,CAV1,CCL2,CD81,CP,CLTC,CLU,COL1A1,C3,COL6A1,COL6A3,COL18A1,CSF1,CSPG4,CXCR4,CYCS,DKK4,DYNLRB1,EEF2,ENO1,HSP90B1,EPO,EPS15,F13A1,FABP4,AFP,AHSG,FGF23,FGFR4,FGA,FGB,FGG,FN1,FKBP1A,FTH1,FST,GAPDH,GDF15,ARHGDIA,GSN,GNAI1,GNAI2,GRB2,HSP90AA1,HSPA1A,HSPA8,HSPB1,IL17RD,IGFBP3,EIF4B,IGF2,IL4R,IL6,CXCL8,KPNB1,IQGAP1,KRT1,LAMA2,LAMA4,LAMB1,LAMB2,LAMC1,LBP,LCK,LDHA,LGALS1,LIF,LRP6,MMP1,MMP2,MMP3,MMP7,MMP9,NME2,NID1,OSM,SERPINE1,PEBP1,PGK1,PGM1,PIGR,PLG,PPP1CA,PRDX1,PRDX3,PTMA,PTPN11,PPP2R4,RAP1A,RHOA,SOD2,SPTBN1,SORBS1,TAGLN,TGFB2,TGFBR1,TXN,TIMP1,TLN1,TNFRSF18,TNFRSF10B,TF,THBS1,THBS2,PLAU,VEGFA,FIGF,VCL,VTN |
| Classical antibody-mediated complement activation | 5 | C1QA,C1QB,C1QC,C1R,C1S |
| G1/S Transition | 22 | PSMC5,PSMA1,PSMA2,PSMA3,PSMA4,PSMA5,PSMA6,PSMA7,PSMB1,PSMB2,PSMB3,PSMB4,PSMB5,PSMB6,PSMD13,PSMD14,PSMD2,PSMD6,PSMD7,PSME1,PSME2,RPS27A |
| Cell Cycle Checkpoints | 22 | PSMC5,PSMA1,PSMA2,PSMA3,PSMA4,PSMA5,PSMA6,PSMA7,PSMB1,PSMB2,PSMB3,PSMB4,PSMB5,PSMB6,PSMD13,PSMD14,PSMD2,PSMD6,PSMD7,PSME1,PSME2,RPS27A |
| TRAIL signaling pathway | 125 | YWHAB,YWHAE,YWHAG,YWHAQ,YWHAZ,A2M,ACTN1,ACTN4,ACTA1,AIFM2,ALDOA,ACTR3,ARPC2,ARPC3,ASAH1,PYCARD,B2M,BAX,BDNF,BMP4,BMPR1B,CALM1,CTSD,CAV1,CCL2,CP,CLTC,CLU,COL1A1,CFL2,COL18A1,CSF1,CXCR4,CYCS,DIABLO,DKK4,DYNLRB1,EEF2,ENO1,HSP90B1,EPO,EPS15,FABP4,AFP,AHSG,FGF23,FGFR4,FGA,FGB,FGG,FN1,FKBP1A,FTH1,FST,GAPDH,GDF15,ARHGDIA,ARHGDIB,GSN,GNAI1,GNAI2,GRB2,HSP90AA1,HSPA1A,HSPA8,HSPB1,IL17RD,IGFBP3,EIF4B,IGF2,IL4R,IL6,CXCL8,KPNB1,IQGAP1,KRT1,LAMC1,LBP,LCK,LDHA,LGALS1,LIF,LMNA,LMNB2,LRP6,MMP1,MMP2,MMP3,MMP7,MMP9,NME2,NUMA1,SERPINE1,PEBP1,PGK1,PGM1,PIGR,PLG,PPP1CA,PRDX1,PRDX3,PTMA,PTPN11,PPP2R4,RAP1A,RHOA,SOD2,SPTBN1,SPTAN1,SORBS1,TAGLN,TGFB2,TGFBR1,TXN,TIMP1,TLN1,TNFRSF18,TNFRSF10B,TRADD,TF,PLAU,VEGFA,VIM,VCL,VTN |
| S Phase | 22 | PSMC5,PSMA1,PSMA2,PSMA3,PSMA4,PSMA5,PSMA6,PSMA7,PSMB1,PSMB2,PSMB3,PSMB4,PSMB5,PSMB6,PSMD13,PSMD14,PSMD2,PSMD6,PSMD7,PSME1,PSME2,RPS27A |
| Integrins in angiogenesis | 15 | CSF1,FGF2,FN1,GSN,HSP90AA1,MFGE8,MMP2,MMP9,PTPN11,RHOA,TLN1,PLAU,VEGFA,VCL,VTN |
| Beta3 integrin cell surface interactions | 12 | COL1A1,FGA,FGB,FGG,FN1,LAMA4,LAMB1,LAMC1,THBS1,PLAU,VEGFA,VTN |
| Caspase cascade in apoptosis | 13 | ACTA1,BAX,CFL2,CYCS,DIABLO,ARHGDIB,GSN,LMNA,LMNB2,NUMA1,SPTAN1,TRADD,VIM |
| Metabolism of mRNA | 33 | YWHAB,YWHAZ,HSPA1A,HSPA8,HSPB1,EIF4B,PSMC5,PSMA1,PSMA2,PSMA3,PSMA4,PSMA5,PSMA6,PSMA7,PSMB1,PSMB2,PSMB3,PSMB4,PSMB5,PSMB6,PSMD13,PSMD14,PSMD2,PSMD6,PSMD7,PSME1,PSME2,RPLP0,RPLP1,RPS25,RPS27A,RPS3,RPSA |
| Lipoprotein metabolism | 9 | A2M,ALB,APOA1,APOA2,APOB,APOC3,APOE,HSPG2,PLTP |
| Mitotic G1-G1/S phases | 23 | CDKN2C,PSMC5,PSMA1,PSMA2,PSMA3,PSMA4,PSMA5,PSMA6,PSMA7,PSMB1,PSMB2,PSMB3,PSMB4,PSMB5,PSMB6,PSMD13,PSMD14,PSMD2,PSMD6,PSMD7,PSME1,PSME2,RPS27A |
| Hormone-sensitive lipase (HSL)-mediated triacylglycerol hydrolysis | 6 | CAV1,FABP4,LIPE,MGLL,PLIN1,PPP1CA |
| Innate Immune System | 27 | PYCARD,C1QA,C1QB,C1QC,C1R,C1S,CAPZA1,CAPZA2,CFB,CFD,C3,-,C5,C7,C8G,C9,EEA1,PRKCSH,HSP90AB1,LBP,LGALS3,PTPN11,RPS27A,SIGIRR,TXN,-,UBE2N |
| Glucose metabolism | 10 | GOT1,CALM1,GPI,MDH1,MDH2,PGK1,PYGB,PYGL,TPI1,UGP2 |
| Plasma membrane estrogen receptor signaling | 119 | YWHAB,YWHAE,YWHAG,YWHAQ,YWHAZ,A2M,ACTN1,ACTN4,ACTA1,AIFM2,ALDOA,ACTR3,ARPC2,ARPC3,PYCARD,B2M,BAX,BDNF,BMP4,BMPR1B,CALM1,CTSD,CAV1,CCL2,CP,CLTC,CLU,COL1A1,COL18A1,CSF1,CXCR4,CYCS,DKK4,DYNLRB1,EEF2,ENO1,HSP90B1,EPO,EPS15,FABP4,AFP,AHSG,FGF23,FGFR4,FGA,FGB,FGG,FN1,FKBP1A,FTH1,FST,GAPDH,GNB1,GNB2L1,GDF15,ARHGDIA,GSN,GNAI1,GNAI2,GRB2,HBEGF,HSP90AA1,HSPA1A,HSPA8,HSPB1,IL17RD,IGFBP3,EIF4B,IGF2,IL4R,IL6,CXCL8,KPNB1,IQGAP1,KRT1,LAMC1,LBP,LCK,LDHA,LGALS1,LIF,LRP6,MMP1,MMP2,MMP3,MMP7,MMP9,MSN,NME2,SERPINE1,PEBP1,PGK1,PGM1,PIGR,PLG,PPP1CA,PRDX1,PRDX3,PTMA,PTPN11,PPP2R4,RAP1A,RHOA,SOD2,SPTBN1,SORBS1,TAGLN,TGFB2,TGFBR1,TXN,TIMP1,TLN1,TNFRSF18,TNFRSF10B,TF,PLAU,VEGFA,VCL,VTN |
| ErbB receptor signaling network | 119 | YWHAB,YWHAE,YWHAG,YWHAQ,YWHAZ,A2M,ACTN1,ACTN4,ACTA1,AIFM2,ALDOA,ACTR3,ARPC2,ARPC3,PYCARD,B2M,BAX,BDNF,BMP4,BMPR1B,CALM1,CTSD,CAV1,CCL2,CP,CLTC,CLU,COL1A1,COL18A1,CSF1,CXCR4,CYCS,DKK4,DYNLRB1,EEF2,ENO1,HSP90B1,EPO,EPS15,ERBB4,FABP4,AFP,AHSG,FGF23,FGFR4,FGA,FGB,FGG,FN1,FKBP1A,FTH1,FST,GAPDH,GDF15,ARHGDIA,GSN,GNAI1,GNAI2,GRB2,HBEGF,HSP90AA1,HSPA1A,HSPA8,HSPB1,IL17RD,IGFBP3,EIF4B,IGF2,IL4R,IL6,CXCL8,KPNB1,IQGAP1,KRT1,LAMC1,LBP,LCK,LDHA,LGALS1,LIF,LRP6,MMP1,MMP2,MMP3,MMP7,MMP9,NME2,NRG1,NRG2,SERPINE1,PEBP1,PGK1,PGM1,PIGR,PLG,PPP1CA,PRDX1,PRDX3,PTMA,PTPN11,PPP2R4,RAP1A,RHOA,SOD2,SPTBN1,SORBS1,TAGLN,TGFB2,TGFBR1,TXN,TIMP1,TLN1,TNFRSF18,TNFRSF10B,TF,PLAU,VEGFA,VCL,VTN |
| Creation of C4 and C2 activators | 5 | C1QA,C1QB,C1QC,C1R,C1S |
| Alpha9 beta1 integrin signaling events | 118 | YWHAB,YWHAE,YWHAG,YWHAQ,YWHAZ,A2M,ACTN1,ACTN4,ACTA1,AIFM2,ALDOA,ACTR3,ARPC2,ARPC3,PYCARD,B2M,BAX,BDNF,BMP4,BMPR1B,CALM1,CTSD,CAV1,CCL2,CP,CLTC,CLU,COL1A1,COL18A1,CSF1,CXCR4,CYCS,DKK4,DYNLRB1,EEF2,ENO1,HSP90B1,EPO,EPS15,F13A1,FABP4,AFP,AHSG,FGF23,FGFR4,FGA,FGB,FGG,FN1,FKBP1A,FTH1,FST,GAPDH,GDF15,ARHGDIA,GSN,GNAI1,GNAI2,GRB2,HSP90AA1,HSPA1A,HSPA8,HSPB1,IL17RD,IGFBP3,EIF4B,IGF2,IL4R,IL6,CXCL8,KPNB1,IQGAP1,KRT1,LAMC1,LBP,LCK,LDHA,LGALS1,LIF,LRP6,MMP1,MMP2,MMP3,MMP7,MMP9,NME2,OSM,SERPINE1,PEBP1,PGK1,PGM1,PIGR,PLG,PPP1CA,PRDX1,PRDX3,PTMA,PTPN11,PPP2R4,RAP1A,RHOA,SOD2,SPTBN1,SORBS1,TAGLN,TGFB2,TGFBR1,TXN,TIMP1,TLN1,TNFRSF18,TNFRSF10B,TF,PLAU,VEGFA,FIGF,VCL,VTN |
| Proteoglycan syndecan-mediated signaling events | 121 | YWHAB,YWHAE,YWHAG,YWHAQ,YWHAZ,A2M,ACTN1,ACTN4,ACTA1,AIFM2,ALDOA,ACTR3,ARPC2,ARPC3,PYCARD,B2M,BAX,BDNF,BMP4,BMPR1B,CALM1,CTSD,CAV1,CCL2,CP,CLTC,CLU,COL1A1,COL18A1,CSF1,CXCR4,CYCS,DKK4,DYNLRB1,EEF2,ENO1,HSP90B1,EPO,EPS15,FABP4,AFP,AHSG,FGF2,FGF23,FGFR4,FGA,FGB,FGG,FN1,FKBP1A,FLNA,FTH1,FST,GAPDH,GNB2L1,GDF15,ARHGDIA,GSN,GNAI1,GNAI2,GRB2,HSP90AA1,HSPA1A,HSPA8,HSPB1,IL17RD,IGFBP3,EIF4B,IGF2,IL4R,IL6,CXCL8,KPNB1,IQGAP1,KRT1,LAMC1,LBP,LCK,LDHA,LGALS1,LIF,LRP6,MMP1,MMP2,MMP3,MMP7,MMP9,NME2,SERPINE1,PEBP1,PGK1,PGM1,PIGR,PLG,PPP1CA,PPIB,PRDX1,PRDX3,PTMA,PTPN11,PPP2R4,RAP1A,RHOA,SOD2,SPTBN1,SORBS1,TAGLN,TGFB2,TGFBR1,TXN,F2,TIMP1,TLN1,TNFRSF18,TNFRSF10B,TF,THBS1,PLAU,VEGFA,VCL,VTN |
| Regulation of Insulin-like Growth Factor (IGF) Activity by Insulin-like Growth Factor Binding Proteins (IGFBPs) | 6 | IGFBP3,IGFBP6,MMP1,MMP2,PLG,F2 |
| Glypican 1 network | 117 | YWHAB,YWHAE,YWHAG,YWHAQ,YWHAZ,A2M,ACTN1,ACTN4,ACTA1,AIFM2,ALDOA,ACTR3,ARPC2,ARPC3,PYCARD,B2M,BAX,BDNF,BMP4,BMPR1B,CALM1,CTSD,CAV1,CCL2,CP,CLTC,CLU,COL1A1,COL18A1,CSF1,CXCR4,CYCS,DKK4,DYNLRB1,EEF2,ENO1,HSP90B1,EPO,EPS15,FABP4,AFP,AHSG,FGF2,FGF23,FGFR4,FGA,FGB,FGG,FN1,FKBP1A,FTH1,FST,GAPDH,GDF15,ARHGDIA,GSN,GNAI1,GNAI2,GRB2,HSP90AA1,HSPA1A,HSPA8,HSPB1,IL17RD,IGFBP3,EIF4B,IGF2,IL4R,IL6,CXCL8,KPNB1,IQGAP1,KRT1,LAMC1,LBP,LCK,LDHA,LGALS1,LIF,LRP6,MMP1,MMP2,MMP3,MMP7,MMP9,NME2,NRG1,SERPINE1,PEBP1,PGK1,PGM1,PIGR,PLG,PPP1CA,PRDX1,PRDX3,PTMA,PTPN11,PPP2R4,RAP1A,RHOA,SOD2,SPTBN1,SORBS1,TAGLN,TGFB2,TGFBR1,TXN,TIMP1,TLN1,TNFRSF18,TNFRSF10B,TF,PLAU,VEGFA,VCL,VTN |
| PAR1-mediated thrombin signaling events | 117 | YWHAB,YWHAE,YWHAG,YWHAQ,YWHAZ,A2M,ACTN1,ACTN4,ACTA1,AIFM2,ALDOA,ACTR3,ARPC2,ARPC3,PYCARD,B2M,BAX,BDNF,BMP4,BMPR1B,CALM1,CTSD,CAV1,CCL2,CP,CLTC,CLU,COL1A1,COL18A1,CSF1,CXCR4,CYCS,DKK4,DYNLRB1,EEF2,ENO1,HSP90B1,EPO,EPS15,FABP4,AFP,AHSG,FGF23,FGFR4,FGA,FGB,FGG,FN1,FKBP1A,FTH1,FST,GAPDH,GNB1,GDF15,ARHGDIA,GSN,GNAI1,GNAI2,GRB2,HSP90AA1,HSPA1A,HSPA8,HSPB1,IL17RD,IGFBP3,EIF4B,IGF2,IL4R,IL6,CXCL8,KPNB1,IQGAP1,KRT1,LAMC1,LBP,LCK,LDHA,LGALS1,LIF,LRP6,MMP1,MMP2,MMP3,MMP7,MMP9,NME2,SERPINE1,PEBP1,PGK1,PGM1,PIGR,PLG,PPP1CA,PRDX1,PRDX3,PTMA,PTPN11,PPP2R4,RAP1A,RHOA,SOD2,SPTBN1,SORBS1,TAGLN,TGFB2,TGFBR1,TXN,F2,TIMP1,TLN1,TNFRSF18,TNFRSF10B,TF,PLAU,VEGFA,VCL,VTN |
| Thrombin/protease-activated receptor (PAR) pathway | 117 | YWHAB,YWHAE,YWHAG,YWHAQ,YWHAZ,A2M,ACTN1,ACTN4,ACTA1,AIFM2,ALDOA,ACTR3,ARPC2,ARPC3,PYCARD,B2M,BAX,BDNF,BMP4,BMPR1B,CALM1,CTSD,CAV1,CCL2,CP,CLTC,CLU,COL1A1,COL18A1,CSF1,CXCR4,CYCS,DKK4,DYNLRB1,EEF2,ENO1,HSP90B1,EPO,EPS15,FABP4,AFP,AHSG,FGF23,FGFR4,FGA,FGB,FGG,FN1,FKBP1A,FTH1,FST,GAPDH,GNB1,GDF15,ARHGDIA,GSN,GNAI1,GNAI2,GRB2,HSP90AA1,HSPA1A,HSPA8,HSPB1,IL17RD,IGFBP3,EIF4B,IGF2,IL4R,IL6,CXCL8,KPNB1,IQGAP1,KRT1,LAMC1,LBP,LCK,LDHA,LGALS1,LIF,LRP6,MMP1,MMP2,MMP3,MMP7,MMP9,NME2,SERPINE1,PEBP1,PGK1,PGM1,PIGR,PLG,PPP1CA,PRDX1,PRDX3,PTMA,PTPN11,PPP2R4,RAP1A,RHOA,SOD2,SPTBN1,SORBS1,TAGLN,TGFB2,TGFBR1,TXN,F2,TIMP1,TLN1,TNFRSF18,TNFRSF10B,TF,PLAU,VEGFA,VCL,VTN |
| VEGF and VEGFR signaling network | 117 | YWHAB,YWHAE,YWHAG,YWHAQ,YWHAZ,A2M,ACTN1,ACTN4,ACTA1,AIFM2,ALDOA,ACTR3,ARPC2,ARPC3,PYCARD,B2M,BAX,BDNF,BMP4,BMPR1B,CALM1,CTSD,CAV1,CCL2,CP,CLTC,CLU,COL1A1,COL18A1,CSF1,CXCR4,CYCS,DKK4,DYNLRB1,EEF2,ENO1,HSP90B1,EPO,EPS15,FABP4,AFP,AHSG,FGF23,FGFR4,FGA,FGB,FGG,FN1,FKBP1A,FTH1,FST,GAPDH,GDF15,ARHGDIA,GSN,GNAI1,GNAI2,GRB2,HSP90AA1,HSP90AB1,HSPA1A,HSPA8,HSPB1,IL17RD,IGFBP3,EIF4B,IGF2,IL4R,IL6,CXCL8,KPNB1,IQGAP1,KRT1,LAMC1,LBP,LCK,LDHA,LGALS1,LIF,LRP6,MMP1,MMP2,MMP3,MMP7,MMP9,NME2,SERPINE1,PEBP1,PGK1,PGM1,PIGR,PLG,PPP1CA,PRDX1,PRDX3,PTMA,PTPN11,PPP2R4,RAP1A,RHOA,SOD2,SPTBN1,SORBS1,TAGLN,TGFB2,TGFBR1,TXN,TIMP1,TLN1,TNFRSF18,TNFRSF10B,TF,PLAU,VEGFA,FIGF,VCL,VTN |
| IGF1 pathway | 116 | YWHAB,YWHAE,YWHAG,YWHAQ,YWHAZ,A2M,ACTN1,ACTN4,ACTA1,AIFM2,ALDOA,ACTR3,ARPC2,ARPC3,PYCARD,B2M,BAX,BDNF,BMP4,BMPR1B,CALM1,CTSD,CAV1,CCL2,CP,CLTC,CLU,COL1A1,COL18A1,CSF1,CXCR4,CYCS,DKK4,DYNLRB1,EEF2,ENO1,HSP90B1,EPO,EPS15,FABP4,AFP,AHSG,FGF23,FGFR4,FGA,FGB,FGG,FN1,FKBP1A,FTH1,FST,GAPDH,GNB2L1,GDF15,ARHGDIA,GSN,GNAI1,GNAI2,GRB2,HSP90AA1,HSPA1A,HSPA8,HSPB1,IL17RD,IGFBP3,EIF4B,IGF2,IL4R,IL6,CXCL8,KPNB1,IQGAP1,KRT1,LAMC1,LBP,LCK,LDHA,LGALS1,LIF,LRP6,MMP1,MMP2,MMP3,MMP7,MMP9,NME2,SERPINE1,PEBP1,PGK1,PGM1,PIGR,PLG,PPP1CA,PRDX1,PRDX3,PTMA,PTPN11,PPP2R4,RAP1A,RHOA,SOD2,SPTBN1,SORBS1,TAGLN,TGFB2,TGFBR1,TXN,TIMP1,TLN1,TNFRSF18,TNFRSF10B,TF,PLAU,VEGFA,VCL,VTN |
| GMCSF-mediated signaling events | 116 | YWHAB,YWHAE,YWHAG,YWHAQ,YWHAZ,A2M,ACTN1,ACTN4,ACTA1,AIFM2,ALDOA,ACTR3,ARPC2,ARPC3,PYCARD,B2M,BAX,BDNF,BMP4,BMPR1B,CALM1,CTSD,CAV1,CCL2,CP,CLTC,CLU,COL1A1,COL18A1,CSF1,CXCR4,CYCS,DKK4,DYNLRB1,EEF2,ENO1,HSP90B1,EPO,EPS15,FABP4,AFP,AHSG,FGF23,FGFR4,FGA,FGB,FGG,FN1,FKBP1A,FTH1,FST,GAPDH,GDF15,ARHGDIA,GSN,GNAI1,GNAI2,GRB2,HSP90AA1,HSPA1A,HSPA8,HSPB1,IL17RD,IGFBP3,EIF4B,IGF2,IL4R,IL6,CXCL8,KPNB1,IQGAP1,KRT1,LAMC1,LBP,LCK,LDHA,LGALS1,LIF,LRP6,MMP1,MMP2,MMP3,MMP7,MMP9,NME2,OSM,SERPINE1,PEBP1,PGK1,PGM1,PIGR,PLG,PPP1CA,PRDX1,PRDX3,PTMA,PTPN11,PPP2R4,RAP1A,RHOA,SOD2,SPTBN1,SORBS1,TAGLN,TGFB2,TGFBR1,TXN,TIMP1,TLN1,TNFRSF18,TNFRSF10B,TF,PLAU,VEGFA,VCL,VTN |
| amb2 Integrin signaling | 10 | APOB,IL6,LCK,MMP2,MMP9,PLG,RAP1A,RHOA,TLN1,PLAU |
| Dissolution of Fibrin Clot | 5 | HRG,IGFBP3,SERPINE1,PLG,PLAU |
| IL3-mediated signaling events | 116 | YWHAB,YWHAE,YWHAG,YWHAQ,YWHAZ,A2M,ACTN1,ACTN4,ACTA1,AIFM2,ALDOA,ACTR3,ARPC2,ARPC3,PYCARD,B2M,BAX,BDNF,BMP4,BMPR1B,CALM1,CTSD,CAV1,CCL2,CP,CLTC,CLU,COL1A1,COL18A1,CSF1,CXCR4,CYCS,DKK4,DYNLRB1,EEF2,ENO1,HSP90B1,EPO,EPS15,FABP4,AFP,AHSG,FGF23,FGFR4,FGA,FGB,FGG,FN1,FKBP1A,FTH1,FST,GAPDH,GDF15,ARHGDIA,GSN,GNAI1,GNAI2,GRB2,HSP90AA1,HSPA1A,HSPA8,HSPB1,IL17RD,IGFBP3,EIF4B,IGF2,IL4R,IL6,CXCL8,KPNB1,IQGAP1,KRT1,LAMC1,LBP,LCK,LDHA,LGALS1,LIF,LRP6,MMP1,MMP2,MMP3,MMP7,MMP9,NME2,OSM,SERPINE1,PEBP1,PGK1,PGM1,PIGR,PLG,PPP1CA,PRDX1,PRDX3,PTMA,PTPN11,PPP2R4,RAP1A,RHOA,SOD2,SPTBN1,SORBS1,TAGLN,TGFB2,TGFBR1,TXN,TIMP1,TLN1,TNFRSF18,TNFRSF10B,TF,PLAU,VEGFA,VCL,VTN |
| Integrin cell surface interactions | 14 | COL1A1,FGA,FGB,FGG,FN1,GRB2,LAMA2,LAMB1,LAMB2,LAMC1,RAP1A,TLN1,THBS1,VTN |
| HDL-mediated lipid transport | 6 | A2M,ALB,APOA1,APOC3,APOE,PLTP |
| Pentose phosphate pathway (hexose monophosphate shunt) | 4 | PGD,PGLS,TALDO1,TKT |
| Signaling events mediated by VEGFR1 and VEGFR2 | 116 | YWHAB,YWHAE,YWHAG,YWHAQ,YWHAZ,A2M,ACTN1,ACTN4,ACTA1,AIFM2,ALDOA,ACTR3,ARPC2,ARPC3,PYCARD,B2M,BAX,BDNF,BMP4,BMPR1B,CALM1,CTSD,CAV1,CCL2,CP,CLTC,CLU,COL1A1,COL18A1,CSF1,CXCR4,CYCS,DKK4,DYNLRB1,EEF2,ENO1,HSP90B1,EPO,EPS15,FABP4,AFP,AHSG,FGF23,FGFR4,FGA,FGB,FGG,FN1,FKBP1A,FTH1,FST,GAPDH,GDF15,ARHGDIA,GSN,GNAI1,GNAI2,GRB2,HSP90AA1,HSP90AB1,HSPA1A,HSPA8,HSPB1,IL17RD,IGFBP3,EIF4B,IGF2,IL4R,IL6,CXCL8,KPNB1,IQGAP1,KRT1,LAMC1,LBP,LCK,LDHA,LGALS1,LIF,LRP6,MMP1,MMP2,MMP3,MMP7,MMP9,NME2,SERPINE1,PEBP1,PGK1,PGM1,PIGR,PLG,PPP1CA,PRDX1,PRDX3,PTMA,PTPN11,PPP2R4,RAP1A,RHOA,SOD2,SPTBN1,SORBS1,TAGLN,TGFB2,TGFBR1,TXN,TIMP1,TLN1,TNFRSF18,TNFRSF10B,TF,PLAU,VEGFA,VCL,VTN |
| Sphingosine 1-phosphate (S1P) pathway | 117 | YWHAB,YWHAE,YWHAG,YWHAQ,YWHAZ,A2M,ACTN1,ACTN4,ACTA1,AIFM2,ALDOA,ACTR3,ARPC2,ARPC3,ASAH1,PYCARD,B2M,BAX,BDNF,BMP4,BMPR1B,CALM1,CTSD,CAV1,CCL2,CP,CLTC,CLU,COL1A1,COL18A1,CSF1,CXCR4,CYCS,DKK4,DYNLRB1,EEF2,ENO1,HSP90B1,EPO,EPS15,FABP4,AFP,AHSG,FGF23,FGFR4,FGA,FGB,FGG,FN1,FKBP1A,FTH1,FST,GAPDH,GDF15,ARHGDIA,GSN,GNAI1,GNAI2,GRB2,HSP90AA1,HSPA1A,HSPA8,HSPB1,IL17RD,IGFBP3,EIF4B,IGF2,IL4R,IL6,CXCL8,KPNB1,IQGAP1,KRT1,LAMC1,LBP,LCK,LDHA,LGALS1,LIF,LRP6,MMP1,MMP2,MMP3,MMP7,MMP9,NME2,SERPINE1,PEBP1,PGK1,PGM1,PIGR,PLG,PPP1CA,PRDX1,PRDX3,PTMA,PTPN11,PPP2R4,RAP1A,RHOA,SOD2,SPTBN1,SORBS1,TAGLN,TGFB2,TGFBR1,TXN,TIMP1,TLN1,TNFRSF18,TNFRSF10B,TRADD,TF,PLAU,VEGFA,VCL,VTN |
| Glypican pathway | 119 | YWHAB,YWHAE,YWHAG,YWHAQ,YWHAZ,A2M,ACTN1,ACTN4,ACTA1,AIFM2,ALDOA,ACTR3,ARPC2,ARPC3,PYCARD,B2M,BAX,BDNF,BMP4,BMPR1B,CALM1,CTSD,CAV1,CCL2,CP,CLTC,CLU,COL1A1,COL18A1,CSF1,CXCR4,CYCS,DKK4,DYNLRB1,EEF2,ENO1,HSP90B1,EPO,EPS15,FABP4,AFP,AHSG,FGF2,FGF23,FGFR4,FGA,FGB,FGG,FN1,FKBP1A,FLNA,FTH1,FST,GAPDH,GDF15,ARHGDIA,GSN,GNAI1,GNAI2,GRB2,HSP90AA1,HSPA1A,HSPA8,HSPB1,IL17RD,IGFBP3,EIF4B,IGF2,IL4R,IL6,CXCL8,KPNB1,IQGAP1,KRT1,KREMEN2,LAMC1,LBP,LCK,LDHA,LGALS1,LIF,LRP6,MMP1,MMP2,MMP3,MMP7,MMP9,NME2,NRG1,SERPINE1,PEBP1,PGK1,PGM1,PIGR,PLG,PPP1CA,PRDX1,PRDX3,PTMA,PTPN11,PPP2R4,RAP1A,RHOA,SOD2,SPTBN1,SORBS1,TAGLN,TGFB2,TGFBR1,TXN,TIMP1,TLN1,TNFRSF18,TNFRSF10B,TF,PLAU,VEGFA,VCL,VTN |
| Alternative complement activation | 3 | CFB,CFD,C3 |
| Syndecan-1-mediated signaling events | 116 | YWHAB,YWHAE,YWHAG,YWHAQ,YWHAZ,A2M,ACTN1,ACTN4,ACTA1,AIFM2,ALDOA,ACTR3,ARPC2,ARPC3,PYCARD,B2M,BAX,BDNF,BMP4,BMPR1B,CALM1,CTSD,CAV1,CCL2,CP,CLTC,CLU,COL1A1,COL18A1,CSF1,CXCR4,CYCS,DKK4,DYNLRB1,EEF2,ENO1,HSP90B1,EPO,EPS15,FABP4,AFP,AHSG,FGF23,FGFR4,FGA,FGB,FGG,FN1,FKBP1A,FTH1,FST,GAPDH,GDF15,ARHGDIA,GSN,GNAI1,GNAI2,GRB2,HSP90AA1,HSPA1A,HSPA8,HSPB1,IL17RD,IGFBP3,EIF4B,IGF2,IL4R,IL6,CXCL8,KPNB1,IQGAP1,KRT1,LAMC1,LBP,LCK,LDHA,LGALS1,LIF,LRP6,MMP1,MMP2,MMP3,MMP7,MMP9,NME2,SERPINE1,PEBP1,PGK1,PGM1,PIGR,PLG,PPP1CA,PPIB,PRDX1,PRDX3,PTMA,PTPN11,PPP2R4,RAP1A,RHOA,SOD2,SPTBN1,SORBS1,TAGLN,TGFB2,TGFBR1,TXN,TIMP1,TLN1,TNFRSF18,TNFRSF10B,TF,PLAU,VEGFA,VCL,VTN |
| Arf6 signaling events | 115 | YWHAB,YWHAE,YWHAG,YWHAQ,YWHAZ,A2M,ACTN1,ACTN4,ACTA1,AIFM2,ALDOA,ACTR3,ARPC2,ARPC3,PYCARD,B2M,BAX,BDNF,BMP4,BMPR1B,CALM1,CTSD,CAV1,CCL2,CP,CLTC,CLU,COL1A1,COL18A1,CSF1,CXCR4,CYCS,DKK4,DYNLRB1,EEF2,ENO1,HSP90B1,EPO,EPS15,FABP4,AFP,AHSG,FGF23,FGFR4,FGA,FGB,FGG,FN1,FKBP1A,FTH1,FST,GAPDH,GDF15,ARHGDIA,GSN,GNAI1,GNAI2,GRB2,HSP90AA1,HSPA1A,HSPA8,HSPB1,IL17RD,IGFBP3,EIF4B,IGF2,IL4R,IL6,CXCL8,KPNB1,IQGAP1,KRT1,LAMC1,LBP,LCK,LDHA,LGALS1,LIF,LRP6,MMP1,MMP2,MMP3,MMP7,MMP9,NME2,SERPINE1,PEBP1,PGK1,PGM1,PIGR,PLG,PPP1CA,PRDX1,PRDX3,PTMA,PTPN11,PPP2R4,RAP1A,RHOA,SOD2,SPTBN1,SORBS1,TAGLN,TGFB2,TGFBR1,TXN,TIMP1,TLN1,TNFRSF18,TNFRSF10B,TF,PLAU,VEGFA,VCL,VTN |
| Internalization of ErbB1 | 115 | YWHAB,YWHAE,YWHAG,YWHAQ,YWHAZ,A2M,ACTN1,ACTN4,ACTA1,AIFM2,ALDOA,ACTR3,ARPC2,ARPC3,PYCARD,B2M,BAX,BDNF,BMP4,BMPR1B,CALM1,CTSD,CAV1,CCL2,CP,CLTC,CLU,COL1A1,COL18A1,CSF1,CXCR4,CYCS,DKK4,DYNLRB1,EEF2,ENO1,HSP90B1,EPO,EPS15,FABP4,AFP,AHSG,FGF23,FGFR4,FGA,FGB,FGG,FN1,FKBP1A,FTH1,FST,GAPDH,GDF15,ARHGDIA,GSN,GNAI1,GNAI2,GRB2,HSP90AA1,HSPA1A,HSPA8,HSPB1,IL17RD,IGFBP3,EIF4B,IGF2,IL4R,IL6,CXCL8,KPNB1,IQGAP1,KRT1,LAMC1,LBP,LCK,LDHA,LGALS1,LIF,LRP6,MMP1,MMP2,MMP3,MMP7,MMP9,NME2,SERPINE1,PEBP1,PGK1,PGM1,PIGR,PLG,PPP1CA,PRDX1,PRDX3,PTMA,PTPN11,PPP2R4,RAP1A,RHOA,SOD2,SPTBN1,SORBS1,TAGLN,TGFB2,TGFBR1,TXN,TIMP1,TLN1,TNFRSF18,TNFRSF10B,TF,PLAU,VEGFA,VCL,VTN |
| Class I PI3K signaling events mediated by Akt | 115 | YWHAB,YWHAE,YWHAG,YWHAQ,YWHAZ,A2M,ACTN1,ACTN4,ACTA1,AIFM2,ALDOA,ACTR3,ARPC2,ARPC3,PYCARD,B2M,BAX,BDNF,BMP4,BMPR1B,CALM1,CTSD,CAV1,CCL2,CP,CLTC,CLU,COL1A1,COL18A1,CSF1,CXCR4,CYCS,DKK4,DYNLRB1,EEF2,ENO1,HSP90B1,EPO,EPS15,FABP4,AFP,AHSG,FGF23,FGFR4,FGA,FGB,FGG,FN1,FKBP1A,FTH1,FST,GAPDH,GDF15,ARHGDIA,GSN,GNAI1,GNAI2,GRB2,HSP90AA1,HSPA1A,HSPA8,HSPB1,IL17RD,IGFBP3,EIF4B,IGF2,IL4R,IL6,CXCL8,KPNB1,IQGAP1,KRT1,LAMC1,LBP,LCK,LDHA,LGALS1,LIF,LRP6,MMP1,MMP2,MMP3,MMP7,MMP9,NME2,SERPINE1,PEBP1,PGK1,PGM1,PIGR,PLG,PPP1CA,PRDX1,PRDX3,PTMA,PTPN11,PPP2R4,RAP1A,RHOA,SOD2,SPTBN1,SORBS1,TAGLN,TGFB2,TGFBR1,TXN,TIMP1,TLN1,TNFRSF18,TNFRSF10B,TF,PLAU,VEGFA,VCL,VTN |
| Urokinase-type plasminogen activator (uPA) and uPAR-mediated signaling | 115 | YWHAB,YWHAE,YWHAG,YWHAQ,YWHAZ,A2M,ACTN1,ACTN4,ACTA1,AIFM2,ALDOA,ACTR3,ARPC2,ARPC3,PYCARD,B2M,BAX,BDNF,BMP4,BMPR1B,CALM1,CTSD,CAV1,CCL2,CP,CLTC,CLU,COL1A1,COL18A1,CSF1,CXCR4,CYCS,DKK4,DYNLRB1,EEF2,ENO1,HSP90B1,EPO,EPS15,FABP4,AFP,AHSG,FGF23,FGFR4,FGA,FGB,FGG,FN1,FKBP1A,FTH1,FST,GAPDH,GDF15,ARHGDIA,GSN,GNAI1,GNAI2,GRB2,HSP90AA1,HSPA1A,HSPA8,HSPB1,IL17RD,IGFBP3,EIF4B,IGF2,IL4R,IL6,CXCL8,KPNB1,IQGAP1,KRT1,LAMC1,LBP,LCK,LDHA,LGALS1,LIF,LRP6,MMP1,MMP2,MMP3,MMP7,MMP9,NME2,SERPINE1,PEBP1,PGK1,PGM1,PIGR,PLG,PPP1CA,PRDX1,PRDX3,PTMA,PTPN11,PPP2R4,RAP1A,RHOA,SOD2,SPTBN1,SORBS1,TAGLN,TGFB2,TGFBR1,TXN,TIMP1,TLN1,TNFRSF18,TNFRSF10B,TF,PLAU,VEGFA,VCL,VTN |
| Signaling events mediated by focal adhesion kinase | 115 | YWHAB,YWHAE,YWHAG,YWHAQ,YWHAZ,A2M,ACTN1,ACTN4,ACTA1,AIFM2,ALDOA,ACTR3,ARPC2,ARPC3,PYCARD,B2M,BAX,BDNF,BMP4,BMPR1B,CALM1,CTSD,CAV1,CCL2,CP,CLTC,CLU,COL1A1,COL18A1,CSF1,CXCR4,CYCS,DKK4,DYNLRB1,EEF2,ENO1,HSP90B1,EPO,EPS15,FABP4,AFP,AHSG,FGF23,FGFR4,FGA,FGB,FGG,FN1,FKBP1A,FTH1,FST,GAPDH,GDF15,ARHGDIA,GSN,GNAI1,GNAI2,GRB2,HSP90AA1,HSPA1A,HSPA8,HSPB1,IL17RD,IGFBP3,EIF4B,IGF2,IL4R,IL6,CXCL8,KPNB1,IQGAP1,KRT1,LAMC1,LBP,LCK,LDHA,LGALS1,LIF,LRP6,MMP1,MMP2,MMP3,MMP7,MMP9,NME2,SERPINE1,PEBP1,PGK1,PGM1,PIGR,PLG,PPP1CA,PRDX1,PRDX3,PTMA,PTPN11,PPP2R4,RAP1A,RHOA,SOD2,SPTBN1,SORBS1,TAGLN,TGFB2,TGFBR1,TXN,TIMP1,TLN1,TNFRSF18,TNFRSF10B,TF,PLAU,VEGFA,VCL,VTN |
| Insulin Pathway | 115 | YWHAB,YWHAE,YWHAG,YWHAQ,YWHAZ,A2M,ACTN1,ACTN4,ACTA1,AIFM2,ALDOA,ACTR3,ARPC2,ARPC3,PYCARD,B2M,BAX,BDNF,BMP4,BMPR1B,CALM1,CTSD,CAV1,CCL2,CP,CLTC,CLU,COL1A1,COL18A1,CSF1,CXCR4,CYCS,DKK4,DYNLRB1,EEF2,ENO1,HSP90B1,EPO,EPS15,FABP4,AFP,AHSG,FGF23,FGFR4,FGA,FGB,FGG,FN1,FKBP1A,FTH1,FST,GAPDH,GDF15,ARHGDIA,GSN,GNAI1,GNAI2,GRB2,HSP90AA1,HSPA1A,HSPA8,HSPB1,IL17RD,IGFBP3,EIF4B,IGF2,IL4R,IL6,CXCL8,KPNB1,IQGAP1,KRT1,LAMC1,LBP,LCK,LDHA,LGALS1,LIF,LRP6,MMP1,MMP2,MMP3,MMP7,MMP9,NME2,SERPINE1,PEBP1,PGK1,PGM1,PIGR,PLG,PPP1CA,PRDX1,PRDX3,PTMA,PTPN11,PPP2R4,RAP1A,RHOA,SOD2,SPTBN1,SORBS1,TAGLN,TGFB2,TGFBR1,TXN,TIMP1,TLN1,TNFRSF18,TNFRSF10B,TF,PLAU,VEGFA,VCL,VTN |
| Arf6 trafficking events | 115 | YWHAB,YWHAE,YWHAG,YWHAQ,YWHAZ,A2M,ACTN1,ACTN4,ACTA1,AIFM2,ALDOA,ACTR3,ARPC2,ARPC3,PYCARD,B2M,BAX,BDNF,BMP4,BMPR1B,CALM1,CTSD,CAV1,CCL2,CP,CLTC,CLU,COL1A1,COL18A1,CSF1,CXCR4,CYCS,DKK4,DYNLRB1,EEF2,ENO1,HSP90B1,EPO,EPS15,FABP4,AFP,AHSG,FGF23,FGFR4,FGA,FGB,FGG,FN1,FKBP1A,FTH1,FST,GAPDH,GDF15,ARHGDIA,GSN,GNAI1,GNAI2,GRB2,HSP90AA1,HSPA1A,HSPA8,HSPB1,IL17RD,IGFBP3,EIF4B,IGF2,IL4R,IL6,CXCL8,KPNB1,IQGAP1,KRT1,LAMC1,LBP,LCK,LDHA,LGALS1,LIF,LRP6,MMP1,MMP2,MMP3,MMP7,MMP9,NME2,SERPINE1,PEBP1,PGK1,PGM1,PIGR,PLG,PPP1CA,PRDX1,PRDX3,PTMA,PTPN11,PPP2R4,RAP1A,RHOA,SOD2,SPTBN1,SORBS1,TAGLN,TGFB2,TGFBR1,TXN,TIMP1,TLN1,TNFRSF18,TNFRSF10B,TF,PLAU,VEGFA,VCL,VTN |
| Class I PI3K signaling events | 115 | YWHAB,YWHAE,YWHAG,YWHAQ,YWHAZ,A2M,ACTN1,ACTN4,ACTA1,AIFM2,ALDOA,ACTR3,ARPC2,ARPC3,PYCARD,B2M,BAX,BDNF,BMP4,BMPR1B,CALM1,CTSD,CAV1,CCL2,CP,CLTC,CLU,COL1A1,COL18A1,CSF1,CXCR4,CYCS,DKK4,DYNLRB1,EEF2,ENO1,HSP90B1,EPO,EPS15,FABP4,AFP,AHSG,FGF23,FGFR4,FGA,FGB,FGG,FN1,FKBP1A,FTH1,FST,GAPDH,GDF15,ARHGDIA,GSN,GNAI1,GNAI2,GRB2,HSP90AA1,HSPA1A,HSPA8,HSPB1,IL17RD,IGFBP3,EIF4B,IGF2,IL4R,IL6,CXCL8,KPNB1,IQGAP1,KRT1,LAMC1,LBP,LCK,LDHA,LGALS1,LIF,LRP6,MMP1,MMP2,MMP3,MMP7,MMP9,NME2,SERPINE1,PEBP1,PGK1,PGM1,PIGR,PLG,PPP1CA,PRDX1,PRDX3,PTMA,PTPN11,PPP2R4,RAP1A,RHOA,SOD2,SPTBN1,SORBS1,TAGLN,TGFB2,TGFBR1,TXN,TIMP1,TLN1,TNFRSF18,TNFRSF10B,TF,PLAU,VEGFA,VCL,VTN |
| PDGFR-beta signaling pathway | 115 | YWHAB,YWHAE,YWHAG,YWHAQ,YWHAZ,A2M,ACTN1,ACTN4,ACTA1,AIFM2,ALDOA,ACTR3,ARPC2,ARPC3,PYCARD,B2M,BAX,BDNF,BMP4,BMPR1B,CALM1,CTSD,CAV1,CCL2,CP,CLTC,CLU,COL1A1,COL18A1,CSF1,CXCR4,CYCS,DKK4,DYNLRB1,EEF2,ENO1,HSP90B1,EPO,EPS15,FABP4,AFP,AHSG,FGF23,FGFR4,FGA,FGB,FGG,FN1,FKBP1A,FTH1,FST,GAPDH,GDF15,ARHGDIA,GSN,GNAI1,GNAI2,GRB2,HSP90AA1,HSPA1A,HSPA8,HSPB1,IL17RD,IGFBP3,EIF4B,IGF2,IL4R,IL6,CXCL8,KPNB1,IQGAP1,KRT1,LAMC1,LBP,LCK,LDHA,LGALS1,LIF,LRP6,MMP1,MMP2,MMP3,MMP7,MMP9,NME2,SERPINE1,PEBP1,PGK1,PGM1,PIGR,PLG,PPP1CA,PRDX1,PRDX3,PTMA,PTPN11,PPP2R4,RAP1A,RHOA,SOD2,SPTBN1,SORBS1,TAGLN,TGFB2,TGFBR1,TXN,TIMP1,TLN1,TNFRSF18,TNFRSF10B,TF,PLAU,VEGFA,VCL,VTN |
| S1P1 pathway | 115 | YWHAB,YWHAE,YWHAG,YWHAQ,YWHAZ,A2M,ACTN1,ACTN4,ACTA1,AIFM2,ALDOA,ACTR3,ARPC2,ARPC3,PYCARD,B2M,BAX,BDNF,BMP4,BMPR1B,CALM1,CTSD,CAV1,CCL2,CP,CLTC,CLU,COL1A1,COL18A1,CSF1,CXCR4,CYCS,DKK4,DYNLRB1,EEF2,ENO1,HSP90B1,EPO,EPS15,FABP4,AFP,AHSG,FGF23,FGFR4,FGA,FGB,FGG,FN1,FKBP1A,FTH1,FST,GAPDH,GDF15,ARHGDIA,GSN,GNAI1,GNAI2,GRB2,HSP90AA1,HSPA1A,HSPA8,HSPB1,IL17RD,IGFBP3,EIF4B,IGF2,IL4R,IL6,CXCL8,KPNB1,IQGAP1,KRT1,LAMC1,LBP,LCK,LDHA,LGALS1,LIF,LRP6,MMP1,MMP2,MMP3,MMP7,MMP9,NME2,SERPINE1,PEBP1,PGK1,PGM1,PIGR,PLG,PPP1CA,PRDX1,PRDX3,PTMA,PTPN11,PPP2R4,RAP1A,RHOA,SOD2,SPTBN1,SORBS1,TAGLN,TGFB2,TGFBR1,TXN,TIMP1,TLN1,TNFRSF18,TNFRSF10B,TF,PLAU,VEGFA,VCL,VTN |
| EGF receptor (ErbB1) signaling pathway | 115 | YWHAB,YWHAE,YWHAG,YWHAQ,YWHAZ,A2M,ACTN1,ACTN4,ACTA1,AIFM2,ALDOA,ACTR3,ARPC2,ARPC3,PYCARD,B2M,BAX,BDNF,BMP4,BMPR1B,CALM1,CTSD,CAV1,CCL2,CP,CLTC,CLU,COL1A1,COL18A1,CSF1,CXCR4,CYCS,DKK4,DYNLRB1,EEF2,ENO1,HSP90B1,EPO,EPS15,FABP4,AFP,AHSG,FGF23,FGFR4,FGA,FGB,FGG,FN1,FKBP1A,FTH1,FST,GAPDH,GDF15,ARHGDIA,GSN,GNAI1,GNAI2,GRB2,HSP90AA1,HSPA1A,HSPA8,HSPB1,IL17RD,IGFBP3,EIF4B,IGF2,IL4R,IL6,CXCL8,KPNB1,IQGAP1,KRT1,LAMC1,LBP,LCK,LDHA,LGALS1,LIF,LRP6,MMP1,MMP2,MMP3,MMP7,MMP9,NME2,SERPINE1,PEBP1,PGK1,PGM1,PIGR,PLG,PPP1CA,PRDX1,PRDX3,PTMA,PTPN11,PPP2R4,RAP1A,RHOA,SOD2,SPTBN1,SORBS1,TAGLN,TGFB2,TGFBR1,TXN,TIMP1,TLN1,TNFRSF18,TNFRSF10B,TF,PLAU,VEGFA,VCL,VTN |
| mTOR signaling pathway | 115 | YWHAB,YWHAE,YWHAG,YWHAQ,YWHAZ,A2M,ACTN1,ACTN4,ACTA1,AIFM2,ALDOA,ACTR3,ARPC2,ARPC3,PYCARD,B2M,BAX,BDNF,BMP4,BMPR1B,CALM1,CTSD,CAV1,CCL2,CP,CLTC,CLU,COL1A1,COL18A1,CSF1,CXCR4,CYCS,DKK4,DYNLRB1,EEF2,ENO1,HSP90B1,EPO,EPS15,FABP4,AFP,AHSG,FGF23,FGFR4,FGA,FGB,FGG,FN1,FKBP1A,FTH1,FST,GAPDH,GDF15,ARHGDIA,GSN,GNAI1,GNAI2,GRB2,HSP90AA1,HSPA1A,HSPA8,HSPB1,IL17RD,IGFBP3,EIF4B,IGF2,IL4R,IL6,CXCL8,KPNB1,IQGAP1,KRT1,LAMC1,LBP,LCK,LDHA,LGALS1,LIF,LRP6,MMP1,MMP2,MMP3,MMP7,MMP9,NME2,SERPINE1,PEBP1,PGK1,PGM1,PIGR,PLG,PPP1CA,PRDX1,PRDX3,PTMA,PTPN11,PPP2R4,RAP1A,RHOA,SOD2,SPTBN1,SORBS1,TAGLN,TGFB2,TGFBR1,TXN,TIMP1,TLN1,TNFRSF18,TNFRSF10B,TF,PLAU,VEGFA,VCL,VTN |
| Arf6 downstream pathway | 115 | YWHAB,YWHAE,YWHAG,YWHAQ,YWHAZ,A2M,ACTN1,ACTN4,ACTA1,AIFM2,ALDOA,ACTR3,ARPC2,ARPC3,PYCARD,B2M,BAX,BDNF,BMP4,BMPR1B,CALM1,CTSD,CAV1,CCL2,CP,CLTC,CLU,COL1A1,COL18A1,CSF1,CXCR4,CYCS,DKK4,DYNLRB1,EEF2,ENO1,HSP90B1,EPO,EPS15,FABP4,AFP,AHSG,FGF23,FGFR4,FGA,FGB,FGG,FN1,FKBP1A,FTH1,FST,GAPDH,GDF15,ARHGDIA,GSN,GNAI1,GNAI2,GRB2,HSP90AA1,HSPA1A,HSPA8,HSPB1,IL17RD,IGFBP3,EIF4B,IGF2,IL4R,IL6,CXCL8,KPNB1,IQGAP1,KRT1,LAMC1,LBP,LCK,LDHA,LGALS1,LIF,LRP6,MMP1,MMP2,MMP3,MMP7,MMP9,NME2,SERPINE1,PEBP1,PGK1,PGM1,PIGR,PLG,PPP1CA,PRDX1,PRDX3,PTMA,PTPN11,PPP2R4,RAP1A,RHOA,SOD2,SPTBN1,SORBS1,TAGLN,TGFB2,TGFBR1,TXN,TIMP1,TLN1,TNFRSF18,TNFRSF10B,TF,PLAU,VEGFA,VCL,VTN |
| ErbB1 downstream signaling | 115 | YWHAB,YWHAE,YWHAG,YWHAQ,YWHAZ,A2M,ACTN1,ACTN4,ACTA1,AIFM2,ALDOA,ACTR3,ARPC2,ARPC3,PYCARD,B2M,BAX,BDNF,BMP4,BMPR1B,CALM1,CTSD,CAV1,CCL2,CP,CLTC,CLU,COL1A1,COL18A1,CSF1,CXCR4,CYCS,DKK4,DYNLRB1,EEF2,ENO1,HSP90B1,EPO,EPS15,FABP4,AFP,AHSG,FGF23,FGFR4,FGA,FGB,FGG,FN1,FKBP1A,FTH1,FST,GAPDH,GDF15,ARHGDIA,GSN,GNAI1,GNAI2,GRB2,HSP90AA1,HSPA1A,HSPA8,HSPB1,IL17RD,IGFBP3,EIF4B,IGF2,IL4R,IL6,CXCL8,KPNB1,IQGAP1,KRT1,LAMC1,LBP,LCK,LDHA,LGALS1,LIF,LRP6,MMP1,MMP2,MMP3,MMP7,MMP9,NME2,SERPINE1,PEBP1,PGK1,PGM1,PIGR,PLG,PPP1CA,PRDX1,PRDX3,PTMA,PTPN11,PPP2R4,RAP1A,RHOA,SOD2,SPTBN1,SORBS1,TAGLN,TGFB2,TGFBR1,TXN,TIMP1,TLN1,TNFRSF18,TNFRSF10B,TF,PLAU,VEGFA,VCL,VTN |
| EGFR-dependent Endothelin signaling events | 115 | YWHAB,YWHAE,YWHAG,YWHAQ,YWHAZ,A2M,ACTN1,ACTN4,ACTA1,AIFM2,ALDOA,ACTR3,ARPC2,ARPC3,PYCARD,B2M,BAX,BDNF,BMP4,BMPR1B,CALM1,CTSD,CAV1,CCL2,CP,CLTC,CLU,COL1A1,COL18A1,CSF1,CXCR4,CYCS,DKK4,DYNLRB1,EEF2,ENO1,HSP90B1,EPO,EPS15,FABP4,AFP,AHSG,FGF23,FGFR4,FGA,FGB,FGG,FN1,FKBP1A,FTH1,FST,GAPDH,GDF15,ARHGDIA,GSN,GNAI1,GNAI2,GRB2,HSP90AA1,HSPA1A,HSPA8,HSPB1,IL17RD,IGFBP3,EIF4B,IGF2,IL4R,IL6,CXCL8,KPNB1,IQGAP1,KRT1,LAMC1,LBP,LCK,LDHA,LGALS1,LIF,LRP6,MMP1,MMP2,MMP3,MMP7,MMP9,NME2,SERPINE1,PEBP1,PGK1,PGM1,PIGR,PLG,PPP1CA,PRDX1,PRDX3,PTMA,PTPN11,PPP2R4,RAP1A,RHOA,SOD2,SPTBN1,SORBS1,TAGLN,TGFB2,TGFBR1,TXN,TIMP1,TLN1,TNFRSF18,TNFRSF10B,TF,PLAU,VEGFA,VCL,VTN |
| Metabolism of carbohydrates | 16 | PGD,PGLS,GOT1,CALM1,GPI,SLC2A2,SLC2A5,MDH1,MDH2,PGK1,PYGB,PYGL,TALDO1,TKT,TPI1,UGP2 |
| IL5-mediated signaling events | 115 | YWHAB,YWHAE,YWHAG,YWHAQ,YWHAZ,A2M,ACTN1,ACTN4,ACTA1,AIFM2,ALDOA,ACTR3,ARPC2,ARPC3,PYCARD,B2M,BAX,BDNF,BMP4,BMPR1B,CALM1,CTSD,CAV1,CCL2,CP,CLTC,CLU,COL1A1,COL18A1,CSF1,CXCR4,CYCS,DKK4,DYNLRB1,EEF2,ENO1,HSP90B1,EPO,EPS15,FABP4,AFP,AHSG,FGF23,FGFR4,FGA,FGB,FGG,FN1,FKBP1A,FTH1,FST,GAPDH,GDF15,ARHGDIA,GSN,GNAI1,GNAI2,GRB2,HSP90AA1,HSPA1A,HSPA8,HSPB1,IL17RD,IGFBP3,EIF4B,IGF2,IL4R,IL6,CXCL8,KPNB1,IQGAP1,KRT1,LAMC1,LBP,LCK,LDHA,LGALS1,LIF,LRP6,MMP1,MMP2,MMP3,MMP7,MMP9,NME2,SERPINE1,PEBP1,PGK1,PGM1,PIGR,PLG,PPP1CA,PRDX1,PRDX3,PTMA,PTPN11,PPP2R4,RAP1A,RHOA,SOD2,SPTBN1,SORBS1,TAGLN,TGFB2,TGFBR1,TXN,TIMP1,TLN1,TNFRSF18,TNFRSF10B,TF,PLAU,VEGFA,VCL,VTN |
| PDGF receptor signaling network | 115 | YWHAB,YWHAE,YWHAG,YWHAQ,YWHAZ,A2M,ACTN1,ACTN4,ACTA1,AIFM2,ALDOA,ACTR3,ARPC2,ARPC3,PYCARD,B2M,BAX,BDNF,BMP4,BMPR1B,CALM1,CTSD,CAV1,CCL2,CP,CLTC,CLU,COL1A1,COL18A1,CSF1,CXCR4,CYCS,DKK4,DYNLRB1,EEF2,ENO1,HSP90B1,EPO,EPS15,FABP4,AFP,AHSG,FGF23,FGFR4,FGA,FGB,FGG,FN1,FKBP1A,FTH1,FST,GAPDH,GDF15,ARHGDIA,GSN,GNAI1,GNAI2,GRB2,HSP90AA1,HSPA1A,HSPA8,HSPB1,IL17RD,IGFBP3,EIF4B,IGF2,IL4R,IL6,CXCL8,KPNB1,IQGAP1,KRT1,LAMC1,LBP,LCK,LDHA,LGALS1,LIF,LRP6,MMP1,MMP2,MMP3,MMP7,MMP9,NME2,SERPINE1,PEBP1,PGK1,PGM1,PIGR,PLG,PPP1CA,PRDX1,PRDX3,PTMA,PTPN11,PPP2R4,RAP1A,RHOA,SOD2,SPTBN1,SORBS1,TAGLN,TGFB2,TGFBR1,TXN,TIMP1,TLN1,TNFRSF18,TNFRSF10B,TF,PLAU,VEGFA,VCL,VTN |
| Signaling events mediated by Hepatocyte Growth Factor Receptor (c-Met) | 115 | YWHAB,YWHAE,YWHAG,YWHAQ,YWHAZ,A2M,ACTN1,ACTN4,ACTA1,AIFM2,ALDOA,ACTR3,ARPC2,ARPC3,PYCARD,B2M,BAX,BDNF,BMP4,BMPR1B,CALM1,CTSD,CAV1,CCL2,CP,CLTC,CLU,COL1A1,COL18A1,CSF1,CXCR4,CYCS,DKK4,DYNLRB1,EEF2,ENO1,HSP90B1,EPO,EPS15,FABP4,AFP,AHSG,FGF23,FGFR4,FGA,FGB,FGG,FN1,FKBP1A,FTH1,FST,GAPDH,GDF15,ARHGDIA,GSN,GNAI1,GNAI2,GRB2,HSP90AA1,HSPA1A,HSPA8,HSPB1,IL17RD,IGFBP3,EIF4B,IGF2,IL4R,IL6,CXCL8,KPNB1,IQGAP1,KRT1,LAMC1,LBP,LCK,LDHA,LGALS1,LIF,LRP6,MMP1,MMP2,MMP3,MMP7,MMP9,NME2,SERPINE1,PEBP1,PGK1,PGM1,PIGR,PLG,PPP1CA,PRDX1,PRDX3,PTMA,PTPN11,PPP2R4,RAP1A,RHOA,SOD2,SPTBN1,SORBS1,TAGLN,TGFB2,TGFBR1,TXN,TIMP1,TLN1,TNFRSF18,TNFRSF10B,TF,PLAU,VEGFA,VCL,VTN |
| Nectin adhesion pathway | 115 | YWHAB,YWHAE,YWHAG,YWHAQ,YWHAZ,A2M,ACTN1,ACTN4,ACTA1,AIFM2,ALDOA,ACTR3,ARPC2,ARPC3,PYCARD,B2M,BAX,BDNF,BMP4,BMPR1B,CALM1,CTSD,CAV1,CCL2,CP,CLTC,CLU,COL1A1,COL18A1,CSF1,CXCR4,CYCS,DKK4,DYNLRB1,EEF2,ENO1,HSP90B1,EPO,EPS15,FABP4,AFP,AHSG,FGF23,FGFR4,FGA,FGB,FGG,FN1,FKBP1A,FTH1,FST,GAPDH,GDF15,ARHGDIA,GSN,GNAI1,GNAI2,GRB2,HSP90AA1,HSPA1A,HSPA8,HSPB1,IL17RD,IGFBP3,EIF4B,IGF2,IL4R,IL6,CXCL8,KPNB1,IQGAP1,KRT1,LAMC1,LBP,LCK,LDHA,LGALS1,LIF,LRP6,MMP1,MMP2,MMP3,MMP7,MMP9,NME2,SERPINE1,PEBP1,PGK1,PGM1,PIGR,PLG,PPP1CA,PRDX1,PRDX3,PTMA,PTPN11,PPP2R4,RAP1A,RHOA,SOD2,SPTBN1,SORBS1,TAGLN,TGFB2,TGFBR1,TXN,TIMP1,TLN1,TNFRSF18,TNFRSF10B,TF,PLAU,VEGFA,VCL,VTN |
| Common Pathway | 6 | F13A1,FGA,FGB,FGG,IGFBP3,F2 |
| Chylomicron-mediated lipid transport | 6 | APOA1,APOA2,APOB,APOC3,APOE,HSPG2 |
| IFN-gamma pathway | 115 | YWHAB,YWHAE,YWHAG,YWHAQ,YWHAZ,A2M,ACTN1,ACTN4,ACTA1,AIFM2,ALDOA,ACTR3,ARPC2,ARPC3,PYCARD,B2M,BAX,BDNF,BMP4,BMPR1B,CALM1,CTSD,CAV1,CCL2,CP,CLTC,CLU,COL1A1,COL18A1,CSF1,CXCR4,CYCS,DKK4,DYNLRB1,EEF2,ENO1,HSP90B1,EPO,EPS15,FABP4,AFP,AHSG,FGF23,FGFR4,FGA,FGB,FGG,FN1,FKBP1A,FTH1,FST,GAPDH,GDF15,ARHGDIA,GSN,GNAI1,GNAI2,GRB2,HSP90AA1,HSPA1A,HSPA8,HSPB1,IL17RD,IGFBP3,EIF4B,IGF2,IL4R,IL6,CXCL8,KPNB1,IQGAP1,KRT1,LAMC1,LBP,LCK,LDHA,LGALS1,LIF,LRP6,MMP1,MMP2,MMP3,MMP7,MMP9,NME2,SERPINE1,PEBP1,PGK1,PGM1,PIGR,PLG,PPP1CA,PRDX1,PRDX3,PTMA,PTPN11,PPP2R4,RAP1A,RHOA,SOD2,SPTBN1,SORBS1,TAGLN,TGFB2,TGFBR1,TXN,TIMP1,TLN1,TNFRSF18,TNFRSF10B,TF,PLAU,VEGFA,VCL,VTN |
| Metabolism of RNA | 34 | YWHAB,YWHAZ,HSPA1A,HSPA8,HSPB1,EIF4B,PSMC5,PSMA1,PSMA2,PSMA3,PSMA4,PSMA5,PSMA6,PSMA7,PSMB1,PSMB2,PSMB3,PSMB4,PSMB5,PSMB6,PSMD13,PSMD14,PSMD2,PSMD6,PSMD7,PSME1,PSME2,RPLP0,RPLP1,RPS25,RPS27A,RPS3,RPSA,SNRPD1 |
| HIV Infection | 27 | AP1B1,B2M,KPNB1,LCK,PPIA,PSMC5,PSMA1,PSMA2,PSMA3,PSMA4,PSMA5,PSMA6,PSMA7,PSMB1,PSMB2,PSMB3,PSMB4,PSMB5,PSMB6,PSMD13,PSMD14,PSMD2,PSMD6,PSMD7,PSME1,PSME2,RPS27A |
| Smooth Muscle Contraction | 7 | CALM1,MYL6,SORBS1,TLN1,TPM3,TPM4,VCL |
| Endothelins | 115 | YWHAB,YWHAE,YWHAG,YWHAQ,YWHAZ,A2M,ACTN1,ACTN4,ACTA1,AIFM2,ALDOA,ACTR3,ARPC2,ARPC3,PYCARD,B2M,BAX,BDNF,BMP4,BMPR1B,CALM1,CTSD,CAV1,CCL2,CP,CLTC,CLU,COL1A1,COL18A1,CSF1,CXCR4,CYCS,DKK4,DYNLRB1,EEF2,ENO1,HSP90B1,EPO,EPS15,FABP4,AFP,AHSG,FGF23,FGFR4,FGA,FGB,FGG,FN1,FKBP1A,FTH1,FST,GAPDH,GDF15,ARHGDIA,GSN,GNAI1,GNAI2,GRB2,HSP90AA1,HSPA1A,HSPA8,HSPB1,IL17RD,IGFBP3,EIF4B,IGF2,IL4R,IL6,CXCL8,KPNB1,IQGAP1,KRT1,LAMC1,LBP,LCK,LDHA,LGALS1,LIF,LRP6,MMP1,MMP2,MMP3,MMP7,MMP9,NME2,SERPINE1,PEBP1,PGK1,PGM1,PIGR,PLG,PPP1CA,PRDX1,PRDX3,PTMA,PTPN11,PPP2R4,RAP1A,RHOA,SOD2,SPTBN1,SORBS1,TAGLN,TGFB2,TGFBR1,TXN,TIMP1,TLN1,TNFRSF18,TNFRSF10B,TF,PLAU,VEGFA,VCL,VTN |
| LKB1 signaling events | 115 | YWHAB,YWHAE,YWHAG,YWHAQ,YWHAZ,A2M,ACTN1,ACTN4,ACTA1,AIFM2,ALDOA,ACTR3,ARPC2,ARPC3,PYCARD,B2M,BAX,BDNF,BMP4,BMPR1B,CALM1,CTSD,CAV1,CCL2,CP,CLTC,CLU,COL1A1,COL18A1,CSF1,CXCR4,CYCS,DKK4,DYNLRB1,EEF2,ENO1,HSP90B1,EPO,EPS15,FABP4,AFP,AHSG,FGF23,FGFR4,FGA,FGB,FGG,FN1,FKBP1A,FTH1,FST,GAPDH,GDF15,ARHGDIA,GSN,GNAI1,GNAI2,GRB2,HSP90AA1,HSPA1A,HSPA8,HSPB1,IL17RD,IGFBP3,EIF4B,IGF2,IL4R,IL6,CXCL8,KPNB1,IQGAP1,KRT1,LAMC1,LBP,LCK,LDHA,LGALS1,LIF,LRP6,MMP1,MMP2,MMP3,MMP7,MMP9,NME2,SERPINE1,PEBP1,PGK1,PGM1,PIGR,PLG,PPP1CA,PRDX1,PRDX3,PTMA,PTPN11,PPP2R4,RAP1A,RHOA,SOD2,SPTBN1,SORBS1,TAGLN,TGFB2,TGFBR1,TXN,TIMP1,TLN1,TNFRSF18,TNFRSF10B,TF,PLAU,VEGFA,VCL,VTN |
| Terminal pathway of complement | 4 | C5,C7,C8G,C9 |
| Mitotic M-M/G1 phases | 30 | YWHAE,YWHAG,ACTR1A,DYNC1H1,HSP90AA1,PRKAR2B,PAFAH1B1,PSMC5,PSMA1,PSMA2,PSMA3,PSMA4,PSMA5,PSMA6,PSMA7,PSMB1,PSMB2,PSMB3,PSMB4,PSMB5,PSMB6,PSMD13,PSMD14,PSMD2,PSMD6,PSMD7,PSME1,PSME2,RPS27A,TUBB |
| Intrinsic Pathway | 6 | A2M,IGFBP3,SERPING1,KLKB1,F2,VWF |
| FOXA transcription factor networks | 14 | SERPINA1,ALB,AP1B1,APOA1,APOB,COL18A1,AFP,GCG,SLC2A2,HADH,SOD1,F2,TTR,VTN |
| TCA cycle variation III (eukaryotic) | 5 | ACO1,CS,FH,MDH1,MDH2 |
| Alpha6Beta4Integrin | 10 | YWHAB,YWHAE,YWHAQ,YWHAZ,GRB2,MMP7,PLEC,RHOA,RPSA,VIM |
| Signaling by Rho GTPases | 4 | GDI2,ARHGDIA,ARHGDIB,RHOA |
| Rho GTPase cycle | 4 | GDI2,ARHGDIA,ARHGDIB,RHOA |
| Gluconeogenesis | 6 | GOT1,GPI,MDH1,MDH2,PGK1,TPI1 |
| Golgi Associated Vesicle Biogenesis | 7 | AP1B1,CLTC,FTH1,FTL,HSPA8,NAPA,TXNDC5 |
| Syndecan-2-mediated signaling events | 13 | BAX,FGF23,FGFR4,FN1,GNB2L1,GRB2,IL17RD,CXCL8,MMP2,MMP9,PTPN11,RHOA,PLAU |
| Advanced glycosylation endproduct receptor signaling | 4 | CAPZA1,CAPZA2,PRKCSH,LGALS3 |
| p38 signaling mediated by MAPKAP kinases | 6 | YWHAB,YWHAE,YWHAG,YWHAQ,YWHAZ,HSPB1 |
| DNA Replication | 30 | YWHAE,YWHAG,ACTR1A,DYNC1H1,HSP90AA1,PRKAR2B,PAFAH1B1,PSMC5,PSMA1,PSMA2,PSMA3,PSMA4,PSMA5,PSMA6,PSMA7,PSMB1,PSMB2,PSMB3,PSMB4,PSMB5,PSMB6,PSMD13,PSMD14,PSMD2,PSMD6,PSMD7,PSME1,PSME2,RPS27A,TUBB |
| Glutathione conjugation | 5 | CNDP2,GGCT,GSTO1,GSTP1,OPLAH |
| Hypoxic and oxygen homeostasis regulation of HIF-1-alpha | 13 | ALDOA,CP,CXCR4,ENO1,EPO,GNB2L1,HSP90AA1,LDHA,SERPINE1,PGK1,PGM1,TF,VEGFA |
| superoxide radicals degradation | 3 | SOD1,SOD3,SOD2 |
| Glycolysis | 3 | GPI,PGK1,TPI1 |
| aspartate degradation II | 3 | GOT1,MDH1,MDH2 |
| Phase II conjugation | 8 | BPNT1,CNDP2,GGCT,GSTO1,GSTP1,OPLAH,AHCY,UGP2 |
| Integrin-linked kinase signaling | 62 | YWHAB,YWHAE,YWHAG,YWHAQ,YWHAZ,A2M,ACTN1,ACTA1,ALDOA,B2M,BAX,CALM1,CAV1,CCL2,CP,CXCR4,DKK4,DYNLRB1,ENO1,HSP90B1,EPO,FABP4,AFP,FGG,FKBP1A,GSN,GRB2,HSP90AA1,HSPA8,HSPB1,IL6,CXCL8,KPNB1,IQGAP1,KRT1,LAMC1,LBP,LCK,LDHA,LIF,MMP1,MMP2,MMP9,NACA,SERPINE1,PARVA,PGK1,PGM1,PPP1CA,PRDX1,PTPN11,RHOA,SPTBN1,TNS1,TGFB2,TGFBR1,TXN,TIMP1,TNFRSF18,TF,PLAU,VEGFA |
| pentose phosphate pathway | 4 | PGD,PGLS,TALDO1,TKT |
| Cell-extracellular matrix interactions | 5 | ACTN1,FERMT2,FLNA,PARVA,RSU1 |
| Peptide ligand-binding receptors | 21 | ANXA1,CCL2,CCL22,CCL27,CCL3,CCR4,CCR7,CCR9,C3,C5,CXCL9,CXCR1,CXCR2,CXCR5,CXCR6,CXCL11,HEBP1,CXCL8,HCRT,PROK1,F2 |
| Metabolism of nitric oxide | 5 | CALM1,CAV1,GCHFR,HSP90AA1,SPR |
| eNOS activation and regulation | 5 | CALM1,CAV1,GCHFR,HSP90AA1,SPR |
| HIF-1-alpha transcription factor network | 11 | ALDOA,CP,CXCR4,ENO1,EPO,LDHA,SERPINE1,PGK1,PGM1,TF,VEGFA |
| Amyloids | 2 | HSPG2,APCS |
| Release of apoptotic factors from the mitochondria | 2 | CYCS,DIABLO |
| Insulin effects increased synthesis of Xylulose-5-Phosphate | 2 | TALDO1,TKT |
| Alpha6 beta4 integrin-ligand interactions | 4 | LAMA2,LAMB1,LAMB2,LAMC1 |
| Caspase-mediated cleavage of cytoskeletal proteins | 4 | GSN,PLEC,SPTAN1,VIM |
| "Tetrahydrobiopterin (BH4) synthesis, recycling, salvage and regulation" | 4 | CALM1,GCHFR,HSP90AA1,SPR |
| Glutathione synthesis and recycling | 3 | CNDP2,GGCT,OPLAH |
| ADP signalling through P2Y purinoceptor 12 | 3 | GNB1,GNAI1,GNAI2 |
| trans-Golgi Network Vesicle Budding | 7 | AP1B1,CLTC,FTH1,FTL,HSPA8,NAPA,TXNDC5 |
| Clathrin derived vesicle budding | 7 | AP1B1,CLTC,FTH1,FTL,HSPA8,NAPA,TXNDC5 |
| Angiopoietin receptor Tie2-mediated signaling | 9 | ANGPT1,ANGPT2,FGF2,FN1,GRB2,MMP2,PLG,PTPN11,F2 |
| CDC42 signaling events | 68 | YWHAB,YWHAE,YWHAG,YWHAQ,YWHAZ,A2M,ACTA1,ALDOA,ACTR3,ARPC2,ARPC3,B2M,BAX,CALM1,CAV1,CCL2,CP,CFL1,CXCR4,DKK4,DYNLRB1,ENO1,HSP90B1,EPO,FABP4,AFP,FGG,FKBP1A,ARHGDIA,ARHGDIB,GSN,GRB2,HSP90AA1,HSPA8,HSPB1,IL6,CXCL8,KPNB1,IQGAP1,KRT1,LAMC1,LBP,LCK,LDHA,LIF,MMP1,MMP2,MMP9,MSN,SERPINE1,PGK1,PGM1,PPP1CA,PRDX1,PTPN11,RHOA,SEPT2,SPTBN1,TGFB2,TGFBR1,TXN,TIMP1,TLN1,TNFRSF18,TF,PLAU,VEGFA,VCL |
| FAS (CD95) signaling pathway | 17 | ACTA1,ASAH1,BAX,CTSD,CLTC,CFL2,CYCS,DIABLO,ARHGDIB,GSN,LCK,LMNA,LMNB2,NUMA1,SPTAN1,TRADD,VIM |
| AP-1 transcription factor network | 57 | YWHAB,YWHAE,YWHAG,YWHAQ,YWHAZ,A2M,ACTA1,ALDOA,B2M,BAX,CALM1,CAV1,CCL2,CP,CXCR4,DKK4,DYNLRB1,ENO1,HSP90B1,EPO,FABP4,AFP,FGG,FKBP1A,GSN,GRB2,HSP90AA1,HSPA8,HSPB1,IL6,CXCL8,KPNB1,KRT1,LAMC1,LBP,LCK,LDHA,LIF,MMP1,MMP2,MMP9,SERPINE1,PGK1,PGM1,PPP1CA,PRDX1,PTPN11,RHOA,SPTBN1,TGFB2,TGFBR1,TXN,TIMP1,TNFRSF18,TF,PLAU,VEGFA |
| sucrose degradation | 3 | ALDOA,ALDOC,TPI1 |
| Axonal growth inhibition (RHOA activation) | 3 | ARHGDIA,RHOA,RTN4 |
| Regulation of CDC42 activity | 68 | YWHAB,YWHAE,YWHAG,YWHAQ,YWHAZ,A2M,ACTA1,ALDOA,ACTR3,ARPC2,ARPC3,B2M,BAX,CALM1,CAV1,CCL2,CP,CFL1,CXCR4,DKK4,DYNLRB1,ENO1,HSP90B1,EPO,FABP4,AFP,FGG,FKBP1A,ARHGDIA,ARHGDIB,GSN,GRB2,HSP90AA1,HSPA8,HSPB1,IL6,CXCL8,KPNB1,IQGAP1,KRT1,LAMC1,LBP,LCK,LDHA,LIF,MMP1,MMP2,MMP9,MSN,SERPINE1,PGK1,PGM1,PPP1CA,PRDX1,PTPN11,RHOA,SEPT2,SPTBN1,TGFB2,TGFBR1,TXN,TIMP1,TLN1,TNFRSF18,TF,PLAU,VEGFA,VCL |
| Insulin-mediated glucose transport | 6 | YWHAB,YWHAE,YWHAG,YWHAQ,YWHAZ,CALM1 |
| "Cell Cycle, Mitotic" | 32 | YWHAE,YWHAG,ACTR1A,CDKN2C,DYNC1H1,HSP90AA1,PRKAR2B,PAFAH1B1,NUMA1,PSMC5,PSMA1,PSMA2,PSMA3,PSMA4,PSMA5,PSMA6,PSMA7,PSMB1,PSMB2,PSMB3,PSMB4,PSMB5,PSMB6,PSMD13,PSMD14,PSMD2,PSMD6,PSMD7,PSME1,PSME2,RPS27A,TUBB |
| Mitochondrial Fatty Acid Beta-Oxidation | 4 | DECR1,ECHS1,ECI1,HADH |
| IL6-mediated signaling events | 8 | A2M,HSP90B1,FGG,GRB2,IL6,LBP,PTPN11,TIMP1 |
| Beta oxidation of butanoyl-CoA to acetyl-CoA | 2 | ECHS1,HADH |
| Activation of C3 and C5 | 2 | C3,C5 |
| glutaryl-CoA degradation | 2 | ACAT2,ACAT1 |
| Syndecan-4-mediated signaling events | 23 | YWHAB,YWHAE,YWHAG,YWHAQ,YWHAZ,ACTN1,CAV1,CXCR4,DKK4,FGF2,FN1,FLNA,GSN,CXCL8,KRT1,LRP6,MMP2,MMP9,PLG,PRDX1,RHOA,F2,THBS1 |
| Basigin interactions | 3 | CAV1,MMP1,PPIA |
| S1P5 pathway | 3 | GNAI1,GNAI2,RHOA |
| Regulation of cytoskeletal remodeling and cell spreading by IPP complex components | 3 | ACTN1,PARVA,RSU1 |
| eNOS activation | 3 | CALM1,CAV1,HSP90AA1 |
| p75NTR regulates axonogenesis | 3 | ARHGDIA,RHOA,RTN4 |
| Lissencephaly gene (LIS1) in neuronal migration and development | 6 | YWHAE,CALM1,DYNC1H1,IQGAP1,PAFAH1B1,RHOA |
| Metabolism of lipids and lipoproteins | 27 | A2M,ACACB,ALB,AKR1B1,APOA1,APOA2,APOB,APOC3,APOE,CAV1,DECR1,ECHS1,ECI1,FABP4,FASN,GPD1,SLC2A2,HADH,IDH1,LIPE,MGLL,HSPG2,PLIN1,PLTP,PPP1CA,ACAT1,GC |
| Muscle contraction | 8 | CALM1,MYL6,SORBS1,TLN1,TPM3,TPM4,VIM,VCL |
| Intrinsic Pathway for Apoptosis | 6 | YWHAB,BAX,CYCS,DIABLO,DYNLL2,VIM |
| N-cadherin signaling events | 26 | YWHAB,YWHAE,YWHAG,YWHAQ,YWHAZ,BDNF,CALM1,DKK4,FGF23,FGFR4,GSN,GRB2,IL17RD,IGF2,CXCL8,IQGAP1,KRT1,LRP6,MMP2,MMP3,MMP7,MMP9,PRDX1,PTPN11,RHOA,PLAU |
| Stabilization and expansion of the E-cadherin adherens junction | 28 | YWHAB,YWHAE,YWHAG,YWHAQ,YWHAZ,ACTN1,BDNF,DKK4,FGF23,FGFR4,GSN,GRB2,IL17RD,IGF2,CXCL8,IQGAP1,KRT1,LRP6,MMP2,MMP3,MMP7,MMP9,PRDX1,PTPN11,RAP1A,RHOA,PLAU,VCL |
| E-cadherin signaling in the nascent adherens junction | 28 | YWHAB,YWHAE,YWHAG,YWHAQ,YWHAZ,ACTN1,BDNF,DKK4,FGF23,FGFR4,GSN,GRB2,IL17RD,IGF2,CXCL8,IQGAP1,KRT1,LRP6,MMP2,MMP3,MMP7,MMP9,PRDX1,PTPN11,RAP1A,RHOA,PLAU,VCL |
| GP1b-IX-V activation signalling | 3 | YWHAZ,FLNA,VWF |
| Phenylalanine and tyrosine catabolism | 3 | QDPR,FAH,PCBD1 |
| Regulation of Insulin Secretion by Glucagon-like Peptide-1 | 4 | GCG,IQGAP1,PRKAR2B,RAP1A |
| Synthesis and interconversion of nucleotide di- and triphosphates | 4 | GLRX,AK1,AK2,TXN |
| Cell surface interactions at the vascular wall | 10 | ANGPT1,ANGPT2,APOB,CAV1,FN1,GRB2,MMP1,PPIA,PTPN11,F2 |
| Alpha4 beta1 integrin signaling events | 6 | YWHAZ,CD81,FN1,TLN1,THBS1,THBS2 |
| TNF alpha/NF-kB | 19 | YWHAB,YWHAE,YWHAG,YWHAQ,YWHAZ,CAV1,FLNA,GNB2L1,HSP90AA1,HSP90AB1,HSPB1,PEBP1,PSMB5,PSMD13,PSMD6,PSMD7,PTPN11,TNFRSF11A,TRADD |
| Posttranslational regulation of adherens junction stability and dissassembly | 24 | YWHAB,YWHAE,YWHAG,YWHAQ,YWHAZ,BDNF,DKK4,FGF23,FGFR4,GSN,GRB2,IL17RD,IGF2,CXCL8,IQGAP1,KRT1,LRP6,MMP2,MMP3,MMP7,MMP9,PRDX1,PTPN11,PLAU |
| E-cadherin signaling events | 28 | YWHAB,YWHAE,YWHAG,YWHAQ,YWHAZ,ACTN1,BDNF,DKK4,FGF23,FGFR4,GSN,GRB2,IL17RD,IGF2,CXCL8,IQGAP1,KRT1,LRP6,MMP2,MMP3,MMP7,MMP9,PRDX1,PTPN11,RAP1A,RHOA,PLAU,VCL |
| FOXA2 and FOXA3 transcription factor networks | 7 | ALB,APOA1,AFP,SLC2A2,HADH,F2,TTR |
| Beta5 beta6 beta7 and beta8 integrin cell surface interactions | 4 | FN1,TGFBR1,PLAU,VTN |
| Rapoport-Luebering glycolytic shunt | 2 | PGAM1,BPGM |
| pentose phosphate pathway (oxidative branch) | 2 | PGD,PGLS |
| N-acetylglucosamine degradation II | 2 | GNPDA1,NAGK |
| Ethanol oxidation | 2 | ALDH1A1,ALDH2 |
| phenylalanine degradation I (aerobic) | 2 | QDPR,PCBD1 |
| Rap1 signalling | 3 | YWHAB,YWHAZ,RAP1A |
| GRB2:SOS provides linkage to MAPK signaling for Intergrins | 3 | GRB2,RAP1A,TLN1 |
| FOXA1 transcription factor network | 7 | SERPINA1,AP1B1,APOB,COL18A1,GCG,SOD1,VTN |
| a6b1 and a6b4 Integrin signaling | 6 | YWHAB,YWHAE,YWHAG,YWHAQ,YWHAZ,GRB2 |
| TNF receptor signaling pathway | 29 | YWHAB,YWHAE,YWHAG,YWHAQ,YWHAZ,ACTA1,ASAH1,BAX,CALM1,CTSD,CAV1,CFL2,CYCS,DIABLO,FKBP1A,GNB2L1,ARHGDIB,GSN,HSPB1,CXCL8,KPNB1,LCK,LMNA,LMNB2,NUMA1,SPTAN1,TXN,TRADD,VIM |
| CD28 dependent Vav1 pathway | 4 | GRB2,LCK,SPTBN1,SPTAN1 |
| Tie2 Signaling | 4 | ANGPT1,ANGPT2,GRB2,PTPN11 |
| The NLRP3 inflammasome | 3 | PYCARD,HSP90AB1,TXN |
| fatty acid beta-oxidation I | 4 | ECHS1,ECI1,HADH,ACAA2 |
| glutathione-mediated detoxification | 4 | GSTM3,GSTO1,GSTP1,GSTT1 |
| NCAM signaling for neurite out-growth | 8 | YWHAB,GRB2,NRTN,PTPN11,RAP1A,SPTBN1,SPTAN1,TLN1 |
| Beta2 integrin cell surface interactions | 5 | C3,FGA,FGB,FGG,PLAU |
| VEGFR1 specific signals | 5 | CALM1,CAV1,HSP90AA1,PTPN11,VEGFA |
| serotonin degradation | 2 | ADH1B,ALDH2 |
| Beta oxidation of octanoyl-CoA to hexanoyl-CoA | 2 | ECHS1,HADH |
| Beta oxidation of hexanoyl-CoA to butanoyl-CoA | 2 | ECHS1,HADH |
| Formyl peptide receptors bind formyl peptides and many other ligands | 2 | ANXA1,HEBP1 |
| Axonal growth stimulation | 2 | ARHGDIA,RHOA |
| pyruvate fermentation to lactate | 2 | LDHA,LDHB |
| Beta oxidation of lauroyl-CoA to decanoyl-CoA-CoA | 2 | ECHS1,HADH |
| Beta oxidation of decanoyl-CoA to octanoyl-CoA-CoA | 2 | ECHS1,HADH |
| FGF signaling pathway | 7 | FGF23,FGFR4,GRB2,IL17RD,MMP9,PTPN11,PLAU |
| Centrosome maturation | 9 | YWHAE,YWHAG,ACTR1A,DYNC1H1,HSP90AA1,PRKAR2B,PAFAH1B1,NUMA1,TUBB |
| Recruitment of mitotic centrosome proteins and complexes | 9 | YWHAE,YWHAG,ACTR1A,DYNC1H1,HSP90AA1,PRKAR2B,PAFAH1B1,NUMA1,TUBB |
| Calnexin/calreticulin cycle | 3 | PRKCSH,PDIA3,UGGT1 |
| Platelet Aggregation (Plug Formation) | 5 | GRB2,RAP1A,F2,TLN1,THPO |
| S1P3 pathway | 5 | CXCR4,GNAI1,GNAI2,RHOA,VEGFA |
| Nephrin interactions | 5 | ACTN1,ACTN4,IQGAP1,SPTBN1,SPTAN1 |
| EphrinB-EPHB pathway | 8 | CXCR4,FGA,FGB,FGG,GRB2,LCK,RAP1A,TF |
| Loss of Nlp from mitotic centrosomes | 8 | YWHAE,YWHAG,ACTR1A,DYNC1H1,HSP90AA1,PRKAR2B,PAFAH1B1,TUBB |
| Loss of proteins required for interphase microtubule organizationÃÂ from the centrosome | 8 | YWHAE,YWHAG,ACTR1A,DYNC1H1,HSP90AA1,PRKAR2B,PAFAH1B1,TUBB |
| Ephrin B reverse signaling | 5 | CXCR4,FGA,FGB,FGG,LCK |
| Mitotic Prophase | 8 | YWHAE,YWHAG,ACTR1A,DYNC1H1,HSP90AA1,PRKAR2B,PAFAH1B1,TUBB |
| Golgi Cisternae Pericentriolar Stack Reorganization | 8 | YWHAE,YWHAG,ACTR1A,DYNC1H1,HSP90AA1,PRKAR2B,PAFAH1B1,TUBB |
| Glycogen breakdown (glycogenolysis) | 3 | CALM1,PYGB,PYGL |
| SHC-mediated signalling | 3 | YWHAB,GRB2,PTPN11 |
| N-glycan trimming in the ER and Calnexin/Calreticulin cycle | 3 | PRKCSH,PDIA3,UGGT1 |
| G-protein activation | 3 | GNB1,GNAI1,GNAI2 |
| p75(NTR)-mediated signaling | 18 | YWHAB,YWHAE,YWHAG,YWHAQ,YWHAZ,BDNF,CYCS,DIABLO,ARHGDIA,GRB2,LCK,MMP3,MMP7,PLG,PTPN11,RAP1A,RHOA,RTN4 |
| methionine degradation I (to homocysteine) | 2 | MAT2B,AHCY |
| pentose phosphate pathway (non-oxidative branch) | 2 | TALDO1,TKT |
| Vitamin C (ascorbate) metabolism | 2 | CYB5A,CYB5R3 |
| mitochondrial fatty acid beta-oxidation of unsaturated fatty acids | 2 | DECR1,ECI1 |
| ethanol degradation II (cytosol) | 2 | ADH1B,ALDH2 |
| Methionine salvage pathway | 2 | GOT1,MTAP |
| Klotho-mediated ligand binding | 2 | FGF23,FGFR4 |
| "tryptophan degradation X (mammalian, via tryptamine)" | 2 | AKR1A1,ALDH2 |
| Signaling events mediated by PTP1B | 7 | CAV1,CSF1,GRB2,LCK,LEPR,RHOA,TXN |
| "Nucleotide-binding domain, leucine rich repeat containing receptor (NLR) signaling pathways" | 6 | PYCARD,HSP90AB1,RPS27A,TXN,-,UBE2N |
| ErbB4 signaling events | 5 | ERBB4,GRB2,HBEGF,NRG1,NRG2 |
| FGFR ligand binding and activation | 3 | FGF2,FGF23,FGFR4 |
| S1P4 pathway | 3 | GNAI1,GNAI2,RHOA |
| Ribosomal scanning and start codon recognition | 7 | EIF3B,EIF3H,EIF4B,RPS25,RPS27A,RPS3,RPSA |
| Translation initiation complex formation | 7 | EIF3B,EIF3H,EIF4B,RPS25,RPS27A,RPS3,RPSA |
| adenine and adenosine salvage I | 1 | APRT |
| Viral RNP Complexes in the Host Cell Nucleus | 1 | HSPA1A |
| NADPH regeneration | 1 | IDH1 |
| ascorbate recycling (cytosolic) | 1 | GLRX |
| S-methyl-5'-thioadenosine degradation II | 1 | MTAP |
| adenine and adenosine salvage VI | 1 | ADK |
| Reverse Transcription of HIV RNA | 1 | PPIA |
| glutamine biosynthesis I | 1 | GLUL |
| Plus-strand DNA synthesis | 1 | PPIA |
| Uncoating of the HIV Virion | 1 | PPIA |
| Minus-strand DNA synthesis | 1 | PPIA |
| "Activation of the mRNA upon binding of the cap-binding complex and eIFs, and subsequent binding to 43S" | 7 | EIF3B,EIF3H,EIF4B,RPS25,RPS27A,RPS3,RPSA |
| GRB2 events in EGFR signaling | 3 | YWHAB,GRB2,PTPN11 |
| Sema3A PAK dependent Axon repulsion | 3 | CFL1,HSP90AA1,HSP90AB1 |
| SOS-mediated signalling | 3 | YWHAB,GRB2,PTPN11 |
| Netrin mediated repulsion signals | 3 | PTPN11,RAP1A,TLN1 |
| FRS2-mediated cascade | 3 | YWHAB,GRB2,PTPN11 |
| PAR4-mediated thrombin signaling events | 3 | GNB1,RHOA,F2 |
| Lysosome Vesicle Biogenesis | 3 | AP1B1,CLTC,HSPA8 |
| Apoptotic factor-mediated response | 2 | CYCS,DIABLO |
| noradrenaline and adrenaline degradation | 2 | ADH1B,ALDH2 |
| Purine salvage | 2 | ADK,APRT |
| acetone degradation I (to methylglyoxal) | 2 | ADH1B,AKR1B1 |
| RNA Polymerase I Promoter Opening | 2 | -,- |
| TRAF6 mediated IRF7 activation in TLR7/8 or 9 signaling | 2 | -,UBE2N |
| Direct p53 effectors | 14 | AIFM2,PYCARD,BAX,CTSD,CAV1,COL18A1,AFP,GDF15,HSPA1A,IGFBP3,LIF,MMP2,SERPINE1,TNFRSF10B |
| p73 transcription factor network | 9 | SERPINA1,BAX,FASN,AFP,GNB2L1,GDF15,IL4R,SERPINE1,PEA15 |
| VEGFR3 signaling in lymphatic endothelium | 4 | COL1A1,FN1,GRB2,FIGF |
| Validated targets of C-MYC transcriptional activation | 9 | BAX,ENO1,GAPDH,HSP90AA1,LDHA,MMP9,NME2,PRDX3,PTMA |
| "Formation of the ternary complex, and subsequently, the 43S complex" | 6 | EIF3B,EIF3H,RPS25,RPS27A,RPS3,RPSA |
| Translation | 12 | EEF1D,EEF1G,EEF2,EIF3B,EIF3H,EIF4B,RPLP0,RPLP1,RPS25,RPS27A,RPS3,RPSA |
| Spry regulation of FGF signaling | 3 | GRB2,PTPN11,RPS27A |
| Signal amplification | 3 | GNB1,GNAI1,GNAI2 |
| SHC1 events in EGFR signaling | 3 | YWHAB,GRB2,PTPN11 |
| SHC-related events | 3 | YWHAB,GRB2,PTPN11 |
| Metabolism of nucleotides | 7 | ADK,APRT,GLRX,AK1,AK2,PAICS,TXN |
| Cell-Cell communication | 12 | ACTN1,ACTN4,FERMT2,FLNA,GRB2,IQGAP1,PARVA,PLEC,PTPN11,RSU1,SPTBN1,SPTAN1 |
| mitochondrial fatty acid beta-oxidation of saturated fatty acids | 2 | ECHS1,HADH |
| G beta:gamma signalling through PI3Kgamma | 2 | GNB1,RHOA |
| cysteine biosynthesis III (mammalia) | 2 | MAT2B,AHCY |
| Interleukin-6 signaling | 2 | IL6,PTPN11 |
| Platelet sensitization by LDL | 2 | APOB,PTPN11 |
| CXCR3-mediated signaling events | 5 | CXCL9,CXCL11,GNB1,GNAI1,GNAI2 |
| Role of Calcineurin-dependent NFAT signaling in lymphocytes | 10 | YWHAB,YWHAE,YWHAG,YWHAQ,YWHAZ,BAX,CALM1,FKBP1A,CXCL8,KPNB1 |
| Signaling by FGFR | 10 | YWHAB,CALM1,FGF2,FGF23,FGFR4,GRB2,IL17RD,PRKAR2B,PTPN11,RPS27A |
| Thrombin signalling through proteinase activated receptors (PARs) | 3 | GNB1,IGFBP3,F2 |
| DARPP-32 events | 3 | CALM1,PRKAR2B,PPP1CA |
| Negative regulation of FGFR signaling | 3 | GRB2,PTPN11,RPS27A |
| Inflammasomes | 3 | PYCARD,HSP90AB1,TXN |
| Apoptotic execution phase | 6 | GSN,KPNB1,LMNA,PLEC,SPTAN1,VIM |
| FoxO family signaling | 6 | YWHAB,YWHAE,YWHAG,YWHAQ,YWHAZ,SOD2 |
| EGFR downregulation | 4 | CLTC,EPS15,GRB2,RPS27A |
| "Fatty acid, triacylglycerol, and ketone body metabolism" | 9 | ACACB,DECR1,ECHS1,ECI1,FASN,GPD1,SLC2A2,HADH,ACAT1 |
| Eukaryotic Translation Elongation | 9 | EEF1D,EEF1G,EEF2,RPLP0,RPLP1,RPS25,RPS27A,RPS3,RPSA |
| Integration of energy metabolism | 9 | FASN,GNB1,GNB2,GCG,IQGAP1,PRKAR2B,RAP1A,TALDO1,TKT |
| Signaling events mediated by HDAC Class II | 5 | YWHAB,YWHAE,GNB1,HSP90AA1,TUBA1B |
| IL8-mediated signaling events | 5 | CXCR1,CXCR2,GNB1,GNAI2,CXCL8 |
| Biological oxidations | 10 | ALDH1A1,ALDH2,BPNT1,CNDP2,GGCT,GSTO1,GSTP1,OPLAH,AHCY,UGP2 |
| IL8- and CXCR1-mediated signaling events | 4 | CXCR1,GNB1,GNAI2,CXCL8 |
| Activation of GABAB receptors | 4 | GNB1,GNB2,GNAI1,GNAI2 |
| GABA B receptor activation | 4 | GNB1,GNB2,GNAI1,GNAI2 |
| ARMS-mediated activation | 3 | YWHAB,PTPN11,RAP1A |
| Signaling mediated by p38-alpha and p38-beta | 6 | YWHAB,YWHAE,YWHAG,YWHAQ,YWHAZ,HSPB1 |
| Nef mediated downregulation of MHC class I complex cell surface expression | 2 | AP1B1,B2M |
| Folding of actin by CCT/TriC | 2 | ACTB,CCT6A |
| gamma-glutamyl cycle | 2 | GGCT,OPLAH |
| tryptophan degradation III (eukaryotic) | 2 | ACAT2,ACAT1 |
| Caspase-8 is formed from procaspase-8 | 2 | TNFRSF10B,TRADD |
| Activation of Pro-Caspase 8 | 2 | TNFRSF10B,TRADD |
| IRAK1 recruits IKK complex upon TLR7/8 or 9 stimulation | 2 | -,UBE2N |
| IRAK1 recruits IKK complex | 2 | -,UBE2N |
| p75 NTR receptor-mediated signalling | 5 | YWHAE,ARHGDIA,RHOA,RPS27A,RTN4 |
| Apoptotic cleavage of cellular proteins | 5 | GSN,LMNA,PLEC,SPTAN1,VIM |
| Diabetes pathways | 19 | CCL2,C19orf10,HSP90B1,GCG,IGFBP3,IGFBP6,CXCL8,LMNA,MMP1,MMP2,PLG,RPLP0,RPLP1,RPS25,RPS27A,RPS3,RPSA,F2,TLN1 |
| Osteopontin-mediated events | 4 | GSN,MMP2,MMP9,PLAU |
| pyridoxal 5'-phosphate salvage pathway | 1 | PDXK |
| palmitate biosynthesis I (animals) | 1 | FASN |
| beta-alanine biosynthesis IV | 1 | ALDH2 |
| fatty acid biosynthesis initiation II | 1 | FASN |
| vRNP Assembly | 1 | HSP90AA1 |
| Entry of Influenza Virion into Host Cell via Endocytosis | 1 | CLTC |
| formaldehyde oxidation II (glutathione-dependent) | 1 | ADH5 |
| Binding and entry of HIV virion | 1 | PPIA |
| FGFR4 ligand binding and activation | 1 | FGFR4 |
| Vitamins B6 activation to pyridoxal phosphate | 1 | PDXK |
| "Activation, translocation and oligomerization of BAX" | 1 | BAX |
| glycerol-3-phosphate shuttle | 1 | GPD1 |
| Glycogen synthesis | 1 | UGP2 |
| Cam-PDE 1 activation | 1 | CALM1 |
| D-glucuronate degradation I | 1 | DCXR |
| Pyrophosphate hydrolysis | 1 | PPA1 |
| histamine degradation | 1 | HNMT |
| LPS transferred from LBP carrier to CD14 | 1 | LBP |
| methylglyoxal degradation I | 1 | GLO1 |
| Serotonin clearance from the synaptic cleft | 1 | ALDH2 |
| Metabolism of serotonin | 1 | ALDH2 |
| Astrocytic Glutamate-Glutamine Uptake And Metabolism | 1 | GLUL |
| Neurotransmitter uptake and Metabolism In Glial Cells | 1 | GLUL |
| Aurora B signaling | 5 | -,PEBP1,PSMA3,RHOA,VIM |
| Trk receptor signaling mediated by PI3K and PLC-gamma | 7 | YWHAB,YWHAE,YWHAG,YWHAQ,YWHAZ,GRB2,RAP1A |
| Citric acid cycle (TCA cycle) | 3 | CS,FH,MDH2 |
| Signaling by BMP | 3 | ACVR2A,BMPR1B,FSTL1 |
| Frs2-mediated activation | 3 | YWHAB,PTPN11,RAP1A |
| Opioid Signalling | 6 | CALM1,GNB1,GNAI1,GNAI2,PRKAR2B,PPP1CA |
| LPA receptor mediated events | 10 | GNB1,GNAI1,GNAI2,HBEGF,IL6,CXCL8,LCK,MMP2,MMP9,RHOA |
| Semaphorin interactions | 7 | CFL1,HSP90AA1,HSP90AB1,MYH9,MYL6,RHOA,TLN1 |
| Signalling to ERKs | 4 | YWHAB,GRB2,PTPN11,RAP1A |
| mevalonate pathway I | 2 | ACAT2,ACAT1 |
| Amino acid synthesis and interconversion (transamination) | 2 | GOT1,GLUL |
| p130Cas linkage to MAPK signaling for integrins | 2 | RAP1A,TLN1 |
| Nef-mediates down modulation of cell surface receptors by recruiting them to clathrin adapters | 3 | AP1B1,B2M,LCK |
| Prolonged ERK activation events | 3 | YWHAB,PTPN11,RAP1A |
| Integrin alphaIIb beta3 signaling | 3 | GRB2,RAP1A,TLN1 |
| Glypican 3 network | 18 | YWHAB,YWHAE,YWHAG,YWHAQ,YWHAZ,BMP4,CAV1,DKK4,FLNA,GSN,CXCL8,KRT1,KREMEN2,LRP6,MMP2,MMP9,PRDX1,RHOA |
| Validated transcriptional targets of TAp63 isoforms | 6 | YWHAQ,BAX,GDF15,IGFBP3,MFGE8,NQO1 |
| IL23-mediated signaling events | 7 | CCL2,CXCL9,CXCL1,IL17F,IL19,IL6,LCK |
| Canonical Wnt signaling pathway | 14 | YWHAB,YWHAE,YWHAG,YWHAQ,YWHAZ,CAV1,DKK4,GSN,CXCL8,KRT1,LRP6,MMP2,MMP9,PRDX1 |
| L1CAM interactions | 8 | CLTC,LAMB1,LAMC1,RAP1A,RHOA,SPTBN1,SPTAN1,TLN1 |
| Noncanonical Wnt signaling pathway | 16 | YWHAB,YWHAE,YWHAG,YWHAQ,YWHAZ,CAV1,DKK4,FLNA,GSN,CXCL8,KRT1,LRP6,MMP2,MMP9,PRDX1,RHOA |
| p63 transcription factor network | 10 | YWHAQ,BAX,FASN,GNB2L1,GDF15,IGFBP3,LRP6,MFGE8,NQO1,NRG1 |
| Signalling to RAS | 3 | YWHAB,GRB2,PTPN11 |
| isoleucine degradation I | 2 | ECHS1,ACAT1 |
| RAF/MAP kinase cascade | 2 | YWHAB,PTPN11 |
| G-protein beta:gamma signalling | 2 | GNB1,RHOA |
| Signal regulatory protein (SIRP) family interactions | 2 | GRB2,PTPN11 |
| PERK regulated gene expression | 2 | CCL2,CXCL8 |
| Glucagon signaling in metabolic regulation | 4 | GNB1,GNB2,GCG,PRKAR2B |
| Alpha-synuclein signaling | 4 | FKBP1A,LCK,PARK7,UCHL1 |
| G alpha (z) signalling events | 3 | GNB1,GNAI1,GNAI2 |
| Sema4D induced cell migration and growth-cone collapse | 3 | MYH9,MYL6,RHOA |
| Activation of BMF and translocation to mitochondria | 1 | DYNLL2 |
| Formation of apoptosome | 1 | CYCS |
| Stimulation of the cell death response by PAK-2p34 | 1 | SPTAN1 |
| Arachidonate production from DAG | 1 | MGLL |
| methylglyoxal degradation III | 1 | AKR1B1 |
| N-acetylglucosamine degradation I | 1 | GNPDA1 |
| Utilization of Ketone Bodies | 1 | ACAT1 |
| S-adenosyl-L-methionine biosynthesis | 1 | MAT2B |
| inosine-5'-phosphate biosynthesis II | 1 | PAICS |
| biotin-carboxyl carrier protein assembly | 1 | ACACB |
| tetrahydrobiopterin biosynthesis I | 1 | SPR |
| tetrahydrobiopterin biosynthesis II | 1 | SPR |
| The AIM2 inflammasome | 1 | PYCARD |
| "Formation of the active cofactor, UDP-glucuronate" | 1 | UGP2 |
| FGFR1c and Klotho ligand binding and activation | 1 | FGF23 |
| aspartate biosynthesis | 1 | GOT1 |
| glutamate degradation II | 1 | GOT1 |
| Validated transcriptional targets of AP1 family members Fra1 and Fra2 | 12 | B2M,CALM1,CCL2,FKBP1A,IL6,CXCL8,LIF,MMP1,MMP2,MMP9,TNFRSF18,PLAU |
| Wnt signaling network | 17 | YWHAB,YWHAE,YWHAG,YWHAQ,YWHAZ,CAV1,DKK4,FLNA,GSN,CXCL8,KRT1,KREMEN2,LRP6,MMP2,MMP9,PRDX1,RHOA |
| PD-1 signaling | 2 | LCK,PTPN11 |
| Facilitative Na+-independent glucose transporters | 2 | SLC2A2,SLC2A5 |
| Interaction between L1 and Ankyrins | 2 | SPTBN1,SPTAN1 |
| valine degradation I | 2 | ECHS1,HIBCH |
| G2/M Transition | 9 | YWHAE,YWHAG,ACTR1A,DYNC1H1,HSP90AA1,PRKAR2B,PAFAH1B1,NUMA1,TUBB |
| Factors involved in megakaryocyte development and platelet production | 10 | CAPZB,CAPZA1,CAPZA2,EHD2,-,HBB,HBD,HBG1,AK3,PRKAR2B |
| IL8- and CXCR2-mediated signaling events | 4 | CXCR2,GNB1,GNAI2,CXCL8 |
| HIF-2-alpha transcription factor network | 4 | EPO,SERPINE1,PGK1,VEGFA |
| EPO signaling pathway | 4 | EPO,GRB2,PTPN11,RAP1A |
| C-MYC pathway | 13 | BAX,CLU,ENO1,FTH1,GAPDH,HSP90AA1,LDHA,LGALS1,MMP9,NME2,PRDX3,PTMA,PPP2R4 |
| Membrane Trafficking | 8 | AP1B1,CLTC,FTH1,FTL,HSPA8,RPS27A,NAPA,TXNDC5 |
| "Antigen Presentation: Folding, assembly and peptide loading of class I MHC" | 3 | B2M,ERAP1,PDIA3 |
| Regulation of nuclear beta catenin signaling and target gene transcription | 12 | YWHAB,YWHAE,YWHAG,YWHAQ,YWHAZ,DKK4,GSN,CXCL8,KRT1,MMP2,MMP9,PRDX1 |
| Class A/1 (Rhodopsin-like receptors) | 22 | ANXA1,CCL2,CCL22,CCL27,CCL3,CCR4,CCR7,CCR9,C3,C5,CXCL9,CXCR1,CXCR2,CXCR5,CXCR6,CXCL11,HEBP1,CXCL8,HCRT,PTGDR2,PROK1,F2 |
| Metabolism of water-soluble vitamins and cofactors | 5 | MTHFD1,CYB5A,FASN,CYB5R3,PDXK |
| Metabolism of vitamins and cofactors | 5 | MTHFD1,CYB5A,FASN,CYB5R3,PDXK |
| Glucocorticoid receptor signaling | 8 | BAX,AFP,FGG,GNB1,HSP90AA1,IL6,CXCL8,MMP1 |
| Mitotic G2-G2/M phases | 9 | YWHAE,YWHAG,ACTR1A,DYNC1H1,HSP90AA1,PRKAR2B,PAFAH1B1,NUMA1,TUBB |
| Death Receptor Signalling | 2 | TNFRSF10B,TRADD |
| Extrinsic Pathway for Apoptosis | 2 | TNFRSF10B,TRADD |
| Adenylate cyclase inhibitory pathway | 2 | GNAI1,GNAI2 |
| Inhibition of adenylate cyclase pathway | 2 | GNAI1,GNAI2 |
| colanic acid building blocks biosynthesis | 2 | GPI,UGP2 |
| superpathway of geranylgeranyldiphosphate biosynthesis I (via mevalonate) | 2 | ACAT2,ACAT1 |
| Platelet Adhesion to exposed collagen | 2 | COL1A1,VWF |
| Purine metabolism | 3 | ADK,APRT,PAICS |
| RhoA signaling pathway | 17 | ACTA1,ACTR3,ARPC2,ARPC3,CFL1,ARHGDIA,ARHGDIB,IL6,CXCL8,IQGAP1,MMP2,MSN,RHOA,TGFB2,TLN1,PLAU,VCL |
| Regulation of RhoA activity | 17 | ACTA1,ACTR3,ARPC2,ARPC3,CFL1,ARHGDIA,ARHGDIB,IL6,CXCL8,IQGAP1,MMP2,MSN,RHOA,TGFB2,TLN1,PLAU,VCL |
| Regulation of RAC1 activity | 17 | ACTA1,ACTR3,ARPC2,ARPC3,CFL1,ARHGDIA,ARHGDIB,IL6,CXCL8,IQGAP1,MMP2,MSN,RHOA,TGFB2,TLN1,PLAU,VCL |
| RAC1 signaling pathway | 17 | ACTA1,ACTR3,ARPC2,ARPC3,CFL1,ARHGDIA,ARHGDIB,IL6,CXCL8,IQGAP1,MMP2,MSN,RHOA,TGFB2,TLN1,PLAU,VCL |
| CD28 co-stimulation | 4 | GRB2,LCK,SPTBN1,SPTAN1 |
| GABA receptor activation | 4 | GNB1,GNB2,GNAI1,GNAI2 |
| Axon guidance | 18 | YWHAB,CAP1,CLTC,CFL1,GRB2,HSP90AA1,HSP90AB1,LAMB1,LAMC1,MYH9,MYL6,NRTN,PTPN11,RAP1A,RHOA,SPTBN1,SPTAN1,TLN1 |
| Unfolded Protein Response | 6 | CCL2,C19orf10,HSP90B1,CXCL8,LMNA,TLN1 |
| 3' -UTR-mediated translational regulation | 9 | EIF3B,EIF3H,EIF4B,RPLP0,RPLP1,RPS25,RPS27A,RPS3,RPSA |
| L13a-mediated translational silencing of Ceruloplasmin expression | 9 | EIF3B,EIF3H,EIF4B,RPLP0,RPLP1,RPS25,RPS27A,RPS3,RPSA |
| Ceramide signaling pathway | 5 | ASAH1,BAX,CTSD,CYCS,TRADD |
| NOD1/2 Signaling Pathway | 3 | RPS27A,-,UBE2N |
| triacylglycerol degradation | 2 | LIPE,MGLL |
| Aurora C signaling | 1 | - |
| LDL-mediated lipid transport | 1 | APOB |
| NADE modulates death signalling | 1 | YWHAE |
| Breakdown of the nuclear lamina | 1 | LMNA |
| S6K1 signalling | 1 | EIF4B |
| Localization of the PINCH-ILK-PARVIN complex to focal adhesions | 1 | PARVA |
| betaKlotho-mediated ligand binding | 1 | FGFR4 |
| Prostacyclin signalling through prostacyclin receptor | 1 | GNB1 |
| NOSTRIN mediated eNOS trafficking | 1 | CAV1 |
| creatine-phosphate biosynthesis | 1 | CKB |
| "tetrahydrofolate salvage from 5,10-methenyltetrahydrofolate" | 1 | MTHFD1 |
| CaMK IV-mediated phosphorylation of CREB | 1 | CALM1 |
| Regulation of Signaling by NODAL | 1 | LEFTY2 |
| estrogen biosynthesis | 1 | AKR1C3 |
| Pregnenolone biosynthesis | 1 | AKR1B1 |
| glucose and glucose-1-phosphate degradation | 1 | PGM1 |
| Activation of CaMK IV | 1 | CALM1 |
| heme degradation | 1 | BLVRB |
| Class II GLUTs | 1 | SLC2A5 |
| GDP-glucose biosynthesis | 1 | PGM1 |
| Synthesis of Ketone Bodies | 1 | ACAT1 |
| Neurotrophic factor-mediated Trk receptor signaling | 9 | YWHAB,YWHAE,YWHAG,YWHAQ,YWHAZ,BDNF,GRB2,PTPN11,RAP1A |
| GTP hydrolysis and joining of the 60S ribosomal subunit | 9 | EIF3B,EIF3H,EIF4B,RPLP0,RPLP1,RPS25,RPS27A,RPS3,RPSA |
| Signalling by NGF | 12 | YWHAB,YWHAE,CALM1,CLTC,ARHGDIA,GRB2,PRKAR2B,PTPN11,RAP1A,RHOA,RPS27A,RTN4 |
| Regulation of IFNA signaling | 4 | GRB2,PTPN11,RAP1A,TLN1 |
| PLC beta mediated events | 4 | CALM1,GNAI1,GNAI2,PRKAR2B |
| ErbB2/ErbB3 signaling events | 4 | GRB2,NRG1,NRG2,PTPN11 |
| Signaling events mediated by HDAC Class III | 3 | BAX,-,TUBA1B |
| S1P2 pathway | 3 | GNAI1,GNAI2,RHOA |
| Nongenotropic Androgen signaling | 3 | GNB1,GNAI1,GNAI2 |
| BMP receptor signaling | 18 | YWHAB,YWHAE,YWHAG,YWHAQ,YWHAZ,BAX,BMP4,BMPR1B,CALM1,AHSG,FKBP1A,FST,HSPB1,CXCL8,KPNB1,LCK,PPP1CA,TXN |
| Formation of a pool of free 40S subunits | 8 | EIF3B,EIF3H,RPLP0,RPLP1,RPS25,RPS27A,RPS3,RPSA |
| Formation of ATP by chemiosmotic coupling | 2 | ATP5B,ATP5D |
| Cooperation of Prefoldin and TriC/CCT in actin and tubulin folding | 2 | ACTB,CCT6A |
| G protein gated Potassium channels | 2 | GNB1,GNB2 |
| Inhibition of voltage gated Ca2+ channels via Gbeta/gamma subunits | 2 | GNB1,GNB2 |
| Activation of G protein gated Potassium channels | 2 | GNB1,GNB2 |
| Metabolism of polyamines | 2 | GOT1,MTAP |
| G-protein mediated events | 4 | CALM1,GNAI1,GNAI2,PRKAR2B |
| Signaling by Interleukins | 8 | YWHAB,YWHAZ,GRB2,IL1R1,IL6,PTPN11,-,UBE2N |
| The role of Nef in HIV-1 replication and disease pathogenesis | 3 | AP1B1,B2M,LCK |
| Sema4D in semaphorin signaling | 3 | MYH9,MYL6,RHOA |
| Costimulation by the CD28 family | 5 | GRB2,LCK,PTPN11,SPTBN1,SPTAN1 |
| p53 pathway | 15 | AIFM2,PYCARD,BAX,CTSD,CAV1,COL18A1,AFP,GDF15,HSPA1A,IGFBP3,LIF,MMP2,SERPINE1,PPP2R4,TNFRSF10B |
| Signal Transduction | 86 | YWHAB,YWHAE,ANXA1,ACVR2A,BMPR1B,CALM1,CCL2,CCL22,CCL27,CCL3,CCR4,CCR7,CCR9,CLTC,COL1A1,C3,C5,CXCL9,CXCR1,CXCR2,CXCR5,CXCR6,CXCL11,EPS15,FGF2,FGF23,FGFR4,FGA,FGB,FGG,FN1,FSTL1,GNB1,GDI2,ARHGDIA,ARHGDIB,GCG,GNAI1,GNAI2,GRB2,HEBP1,IL17RD,EIF4B,CXCL8,PRKAR2B,LAMA2,LAMB1,LAMB2,LAMC1,MGLL,MMP9,HCRT,PTGDR2,PPP1CA,PROK1,PSMC5,PSMA1,PSMA2,PSMA3,PSMA4,PSMA5,PSMA6,PSMA7,PSMB1,PSMB2,PSMB3,PSMB4,PSMB5,PSMB6,PSMD13,PSMD14,PSMD2,PSMD6,PSMD7,PSME1,PSME2,PTPN11,RAP1A,RHOA,RPS27A,RTN4,TGFBR1,F2,TLN1,THBS1,VTN |
| IL2 signaling events mediated by STAT5 | 3 | GRB2,LCK,PTPN11 |
| Protein folding | 3 | ACTB,TBCA,CCT6A |
| Interleukin-2 signaling | 3 | YWHAB,GRB2,PTPN11 |
| Activation of caspases through apoptosome-mediated cleavage | 1 | CYCS |
| Cytochrome c-mediated apoptotic response | 1 | CYCS |
| CREB phosphorylation through the activation of CaMKK | 1 | CALM1 |
| SMAC-mediated apoptotic response | 1 | DIABLO |
| SMAC binds to IAPs | 1 | DIABLO |
| Activation of BIM and translocation to mitochondria | 1 | DYNLL2 |
| SMAC-mediated dissociation of IAP:caspase complexes | 1 | DIABLO |
| Extrinsic Pathway | 1 | F3 |
| G beta:gamma signalling through PLC beta | 1 | GNB1 |
| Ketone body metabolism | 1 | ACAT1 |
| tyrosine degradation I | 1 | FAH |
| "phenylalanine degradation IV (mammalian, via side chain)" | 1 | ALDH2 |
| "superpathway of N-acetylglucosamine, N-acetylmannosamine and N-acetylneuraminate degradation" | 1 | GNPDA1 |
| Cytosolic sulfonation of small molecules | 1 | BPNT1 |
| "Activation, myristolyation of BID and translocation to mitochondria" | 1 | VIM |
| Glyoxylate metabolism | 1 | GRHPR |
| RAF activation | 1 | YWHAB |
| putrescine degradation III | 1 | ALDH2 |
| folate polyglutamylation | 1 | MTHFD1 |
| APOBEC3G mediated resistance to HIV-1 infection | 1 | PPIA |
| tetrapyrrole biosynthesis II | 1 | ALAD |
| dopamine degradation | 1 | ALDH2 |
| CTLA4 inhibitory signaling | 1 | PTPN11 |
| Chaperonin-mediated protein folding | 2 | ACTB,CCT6A |
| Signaling by NODAL | 2 | ACVR2A,LEFTY2 |
| Signalling to p38 via RIT and RIN | 2 | YWHAB,PTPN11 |
| Glucocorticoid receptor regulatory network | 7 | BAX,AFP,FGG,HSP90AA1,IL6,CXCL8,MMP1 |
| Peptide chain elongation | 7 | EEF2,RPLP0,RPLP1,RPS25,RPS27A,RPS3,RPSA |
| CXCR4-mediated signaling events | 15 | CALM1,CFL1,CXCR4,FKBP1A,FLNA,GNB1,GNB2L1,GNAI1,GNAI2,GRB2,LCK,MMP9,PTPN11,RAP1A,RHOA |
| Regulation of cytoplasmic and nuclear SMAD2/3 signaling | 23 | YWHAB,YWHAE,YWHAG,YWHAQ,YWHAZ,BAX,CALM1,CAV1,DYNLRB1,FKBP1A,GRB2,HSPA8,HSPB1,CXCL8,KPNB1,LAMC1,LCK,SERPINE1,PPP1CA,RHOA,SPTBN1,TGFBR1,TXN |
| Regulation of nuclear SMAD2/3 signaling | 23 | YWHAB,YWHAE,YWHAG,YWHAQ,YWHAZ,BAX,CALM1,CAV1,DYNLRB1,FKBP1A,GRB2,HSPA8,HSPB1,CXCL8,KPNB1,LAMC1,LCK,SERPINE1,PPP1CA,RHOA,SPTBN1,TGFBR1,TXN |
| TGF-beta receptor signaling | 23 | YWHAB,YWHAE,YWHAG,YWHAQ,YWHAZ,BAX,CALM1,CAV1,DYNLRB1,FKBP1A,GRB2,HSPA8,HSPB1,CXCL8,KPNB1,LAMC1,LCK,SERPINE1,PPP1CA,RHOA,SPTBN1,TGFBR1,TXN |
| Cap-dependent Translation Initiation | 9 | EIF3B,EIF3H,EIF4B,RPLP0,RPLP1,RPS25,RPS27A,RPS3,RPSA |
| Eukaryotic Translation Initiation | 9 | EIF3B,EIF3H,EIF4B,RPLP0,RPLP1,RPS25,RPS27A,RPS3,RPSA |
| G alpha (i) signalling events | 3 | GNB1,GNAI1,GNAI2 |
| "Interleukin-3, 5 and GM-CSF signaling" | 3 | YWHAZ,GRB2,PTPN11 |
| ALK1 signaling events | 24 | YWHAB,YWHAE,YWHAG,YWHAQ,YWHAZ,ACVR2A,BAX,CALM1,CAV1,DYNLRB1,FKBP1A,GRB2,HSPA8,HSPB1,CXCL8,KPNB1,LAMC1,LCK,SERPINE1,PPP1CA,RHOA,SPTBN1,TGFBR1,TXN |
| superpathway of methionine degradation | 2 | MAT2B,AHCY |
| Branched-chain amino acid catabolism | 2 | HIBCH,ACAT1 |
| Activation of BH3-only proteins | 2 | YWHAB,DYNLL2 |
| Cell junction organization | 6 | ACTN1,FERMT2,FLNA,PARVA,PLEC,RSU1 |
| Striated Muscle Contraction | 3 | TPM3,TPM4,VIM |
| Signaling by EGFR | 8 | YWHAB,CALM1,CLTC,EPS15,GRB2,PRKAR2B,PTPN11,RPS27A |
| ALK1 pathway | 24 | YWHAB,YWHAE,YWHAG,YWHAQ,YWHAZ,ACVR2A,BAX,CALM1,CAV1,DYNLRB1,FKBP1A,GRB2,HSPA8,HSPB1,CXCL8,KPNB1,LAMC1,LCK,SERPINE1,PPP1CA,RHOA,SPTBN1,TGFBR1,TXN |
| Neurotransmitter Clearance In The Synaptic Cleft | 1 | ALDH2 |
| Regulation of signaling by CBL | 1 | GRB2 |
| FGFR1b ligand binding and activation | 1 | FGF2 |
| ChREBP activates metabolic gene expression | 1 | FASN |
| ER Quality Control Compartment (ERQC) | 1 | UGGT1 |
| glycogen biosynthesis II (from UDP-D-Glucose) | 1 | UGP2 |
| Presynaptic function of Kainate receptors | 1 | GNB1 |
| GDP-mannose biosynthesis | 1 | GPI |
| TRAIL signaling | 1 | TNFRSF10B |
| oxidative ethanol degradation III (microsomal) | 1 | ALDH2 |
| SHC-mediated cascade | 1 | GRB2 |
| ERK activation | 1 | PTPN11 |
| Glucuronidation | 1 | UGP2 |
| Methylation | 1 | AHCY |
| PKA activation | 2 | CALM1,PRKAR2B |
| G alpha (s) signalling events | 3 | GNB1,GNAI1,GNAI2 |
| Downstream TCR signaling | 3 | LCK,-,UBE2N |
| Pyruvate metabolism and Citric Acid (TCA) cycle | 3 | CS,FH,MDH2 |
| RNA Polymerase I Transcription | 3 | -,-,PTRF |
| Recycling pathway of L1 | 3 | CLTC,RAP1A,TLN1 |
| ATF-2 transcription factor network | 5 | IL6,CXCL8,MMP2,TGFB2,PLAU |
| PKA-mediated phosphorylation of CREB | 2 | CALM1,PRKAR2B |
| EPHA2 forward signaling | 2 | GRB2,RHOA |
| Metabolism of proteins | 19 | ACTB,EEF1D,EEF1G,EEF2,EIF3B,EIF3H,PRKCSH,EIF4B,PDIA3,RPLP0,RPLP1,RPS25,RPS27A,RPS3,RPSA,TBCA,CCT6A,F2,UGGT1 |
| Toll Like Receptor 4 (TLR4) Cascade | 6 | LBP,PTPN11,RPS27A,SIGIRR,-,UBE2N |
| RAF phosphorylates MEK | 1 | YWHAB |
| gamma-linolenate biosynthesis II (animals) | 1 | CYB5A |
| Translocation of ZAP-70 to Immunological synapse | 1 | LCK |
| Interleukin receptor SHC signaling | 1 | GRB2 |
| FGFR1 ligand binding and activation | 1 | FGF2 |
| Heme biosynthesis | 1 | ALAD |
| sphingosine and sphingosine-1-phosphate metabolism | 1 | ASAH1 |
| ethanol degradation IV (peroxisomal) | 1 | ALDH2 |
| MEK activation | 1 | YWHAB |
| TNF signaling | 1 | TRADD |
| Activation of BAD and translocation to mitochondria | 1 | YWHAB |
| histidine degradation III | 1 | MTHFD1 |
| PECAM1 interactions | 1 | PTPN11 |
| Vitamin D (calciferol) metabolism | 1 | GC |
| Purine ribonucleoside monophosphate biosynthesis | 1 | PAICS |
| Toll Receptor Cascades | 7 | EEA1,LBP,PTPN11,RPS27A,SIGIRR,-,UBE2N |
| Hedgehog signaling events mediated by Gli proteins | 4 | GNB1,GNAI1,GNAI2,LGALS3 |
| MyD88:Mal cascade initiated on plasma membrane | 5 | PTPN11,RPS27A,SIGIRR,-,UBE2N |
| Influenza Life Cycle | 10 | CLTC,HSP90AA1,HSPA1A,KPNB1,RPLP0,RPLP1,RPS25,RPS27A,RPS3,RPSA |
| Regulation of Insulin Secretion | 4 | GCG,IQGAP1,PRKAR2B,RAP1A |
| PDGFR-alpha signaling pathway | 2 | CAV1,GRB2 |
| "Cell death signalling via NRAGE, NRIF and NADE" | 2 | YWHAE,RPS27A |
| G alpha (12/13) signalling events | 2 | GNB1,RHOA |
| Toll Like Receptor TLR6:TLR2 Cascade | 5 | PTPN11,RPS27A,SIGIRR,-,UBE2N |
| Validated transcriptional targets of deltaNp63 isoforms | 5 | FASN,GNB2L1,IGFBP3,LRP6,NRG1 |
| Toll Like Receptor TLR1:TLR2 Cascade | 5 | PTPN11,RPS27A,SIGIRR,-,UBE2N |
| Regulation of p38-alpha and p38-beta | 12 | YWHAB,YWHAE,YWHAG,YWHAQ,YWHAZ,BAX,CALM1,FKBP1A,HSPB1,CXCL8,KPNB1,LCK |
| Activation of Kainate Receptors upon glutamate binding | 1 | GNB1 |
| Import of palmitoyl-CoA into the mitochondrial matrix | 1 | ACACB |
| Type I hemidesmosome assembly | 1 | PLEC |
| Integration of provirus | 1 | PPIA |
| Sema4D mediated inhibition of cell attachment and migration | 1 | RHOA |
| Nef and signal transduction | 1 | LCK |
| folate transformations | 1 | MTHFD1 |
| Apoptosis induced DNA fragmentation | 1 | KPNB1 |
| Activation of DNA fragmentation factor | 1 | KPNB1 |
| Regulation of the Fanconi anemia pathway | 1 | RPS27A |
| pyrimidine ribonucleotides interconversion | 1 | NME2 |
| Metabolism of folate and pterines | 1 | MTHFD1 |
| Transport of gamma-carboxylated protein precursors from the endoplasmic reticulum to the Golgi apparatus | 1 | F2 |
| Orexin and neuropeptides FF and QRFP bind to their respective receptors | 1 | HCRT |
| Formation of tubulin folding intermediates by CCT/TriC | 1 | CCT6A |
| androgen biosynthesis | 1 | AKR1C3 |
| Packaging Of Telomere Ends | 1 | - |
| Thromboxane signalling through TP receptor | 1 | GNB1 |
| NGF signalling via TRKA from the plasma membrane | 8 | YWHAB,CALM1,CLTC,GRB2,PRKAR2B,PTPN11,RAP1A,RHOA |
| Signaling events mediated by TCPTP | 7 | COL1A1,CSF1,GRB2,IL4R,KPNB1,PIGR,VEGFA |
| Toll Like Receptor 9 (TLR9) Cascade | 5 | EEA1,PTPN11,RPS27A,-,UBE2N |
| Toll Like Receptor 2 (TLR2) Cascade | 5 | PTPN11,RPS27A,SIGIRR,-,UBE2N |
| Validated nuclear estrogen receptor alpha network | 5 | AP1B1,CTSD,C3,COL18A1,SOD1 |
| Signaling events mediated by the Hedgehog family | 5 | GNB1,GNAI1,GNAI2,LGALS3,TGFB2 |
| IRS-mediated signalling | 4 | YWHAB,GRB2,EIF4B,PTPN11 |
| Influenza Infection | 10 | CLTC,HSP90AA1,HSPA1A,KPNB1,RPLP0,RPLP1,RPS25,RPS27A,RPS3,RPSA |
| Downstream signaling of activated FGFR | 5 | YWHAB,CALM1,GRB2,PRKAR2B,PTPN11 |
| Eukaryotic Translation Termination | 6 | RPLP0,RPLP1,RPS25,RPS27A,RPS3,RPSA |
| Viral mRNA Translation | 6 | RPLP0,RPLP1,RPS25,RPS27A,RPS3,RPSA |
| Developmental Biology | 30 | YWHAB,ADIPOQ,ACVR2A,CAP1,CLTC,CFL1,FABP4,GRB2,SLC2A2,HSP90AA1,HSP90AB1,LAMB1,LAMC1,LEFTY2,MYH9,MYL6,NRTN,PLIN1,PTPN11,RAP1A,RHOA,RPLP0,RPLP1,RPS25,RPS27A,RPS3,RPSA,SPTBN1,SPTAN1,TLN1 |
| IL2 signaling events mediated by PI3K | 5 | CALM1,GRB2,HSP90AA1,LCK,PTPN11 |
| Gamma-carboxylation of protein precursors | 1 | F2 |
| Association of TriC/CCT with target proteins during biosynthesis | 1 | CCT6A |
| Vitamin B5 (pantothenate) metabolism | 1 | FASN |
| Activation of Chaperones by ATF6-alpha | 1 | HSP90B1 |
| Rapid glucocorticoid signaling | 1 | GNB1 |
| Endosomal/Vacuolar pathway | 1 | B2M |
| ABCA transporters in lipid homeostasis | 1 | APOA1 |
| Nef Mediated CD4 Down-regulation | 1 | LCK |
| a4b7 Integrin signaling | 1 | RHOA |
| Role of Abl in Robo-Slit signaling | 1 | CAP1 |
| heme biosynthesis II | 1 | ALAD |
| glutathione redox reactions I | 1 | GPX3 |
| Phosphorylation of CD3 and TCR zeta chains | 1 | LCK |
| Prostanoid ligand receptors | 1 | PTGDR2 |
| Signaling events mediated by PRL | 2 | RHOA,TUBA1B |
| Inwardly rectifying K+ channels | 2 | GNB1,GNB2 |
| IRS-related events | 4 | YWHAB,GRB2,EIF4B,PTPN11 |
| Influenza Viral RNA Transcription and Replication | 7 | HSP90AA1,RPLP0,RPLP1,RPS25,RPS27A,RPS3,RPSA |
| Regulation of gene expression in beta cells | 7 | SLC2A2,RPLP0,RPLP1,RPS25,RPS27A,RPS3,RPSA |
| Interleukin-1 signaling | 3 | IL1R1,-,UBE2N |
| GPCR ligand binding | 23 | ANXA1,CCL2,CCL22,CCL27,CCL3,CCR4,CCR7,CCR9,C3,C5,CXCL9,CXCR1,CXCR2,CXCR5,CXCR6,CXCL11,GCG,HEBP1,CXCL8,HCRT,PTGDR2,PROK1,F2 |
| Calmodulin induced events | 2 | CALM1,PRKAR2B |
| CaM pathway | 2 | CALM1,PRKAR2B |
| RNA Polymerase I Chain Elongation | 2 | -,- |
| IL1-mediated signaling events | 16 | YWHAB,YWHAE,YWHAG,YWHAQ,YWHAZ,BAX,CALM1,FKBP1A,HSPB1,IL1R1,CXCL8,KPNB1,LCK,TXN,-,UBE2N |
| ATM pathway | 21 | YWHAB,YWHAZ,AIFM2,PYCARD,BAX,CTSD,CAV1,CDKN2C,COL18A1,CES1,AFP,GDF15,HSPA1A,IGFBP3,LIF,MMP2,SERPINE1,PPP2R4,TNFRSF10B,UBE2N,PLAU |
| Thromboxane A2 receptor signaling | 4 | GNB1,GNAI2,LCK,RHOA |
| Removal of aminoterminal propeptides from gamma-carboxylated proteins | 1 | F2 |
| S6K1-mediated signalling | 1 | EIF4B |
| mTORC1-mediated signalling | 1 | EIF4B |
| ADP signalling through P2Y purinoceptor 1 | 1 | GNB1 |
| pyrimidine ribonucleotides de novo biosynthesis | 1 | NME2 |
| Glycoprotein hormones | 1 | INHBB |
| Transport of organic anions | 1 | ALB |
| IRAK2 mediated activation of TAK1 complex | 1 | RPS27A |
| Metabolism of porphyrins | 1 | ALAD |
| IRAK2 mediated activation of TAK1 complex upon TLR7/8 or 9 stimulation | 1 | RPS27A |
| p38 MAPK signaling pathway | 13 | YWHAB,YWHAE,YWHAG,YWHAQ,YWHAZ,BAX,CALM1,FKBP1A,HSPB1,CXCL8,KPNB1,LCK,TXN |
| Signaling events regulated by Ret tyrosine kinase | 5 | GRB2,LCK,PTPN11,RAP1A,RHOA |
| ATR signaling pathway | 17 | YWHAB,YWHAZ,AIFM2,PYCARD,BAX,CTSD,CAV1,COL18A1,AFP,GDF15,HSPA1A,IGFBP3,LIF,MMP2,SERPINE1,PPP2R4,TNFRSF10B |
| Steroid hormones | 2 | AKR1B1,GC |
| superpathway of cholesterol biosynthesis | 2 | ACAT2,ACAT1 |
| EPHB forward signaling | 3 | GRB2,RAP1A,TF |
| Platelet homeostasis | 3 | APOB,GNB1,PTPN11 |
| Insulin receptor signalling cascade | 4 | YWHAB,GRB2,EIF4B,PTPN11 |
| Activated TLR4 signalling | 5 | PTPN11,RPS27A,SIGIRR,-,UBE2N |
| Nonsense Mediated Decay Independent of the Exon Junction Complex | 6 | RPLP0,RPLP1,RPS25,RPS27A,RPS3,RPSA |
| Signaling events mediated by HDAC Class I | 7 | YWHAB,YWHAE,BAX,GNB1,-,HSP90AA1,TUBA1B |
| FOXM1 transcription factor network | 3 | HSPA1A,LAMA4,MMP2 |
| Phospholipase C-mediated cascade | 2 | CALM1,PRKAR2B |
| Ca-dependent events | 2 | CALM1,PRKAR2B |
| DAG and IP3 signaling | 2 | CALM1,PRKAR2B |
| purine nucleotides de novo biosynthesis II | 1 | PAICS |
| Signal attenuation | 1 | GRB2 |
| ALK2 signaling events | 1 | FKBP1A |
| Retrograde neurotrophin signalling | 1 | CLTC |
| Recycling of bile acids and salts | 1 | ALB |
| Early Phase of HIV Life Cycle | 1 | PPIA |
| Endogenous TLR signaling | 4 | LCK,RHOA,S100A8,S100A9 |
| PI-3K cascade | 2 | GRB2,PTPN11 |
| GPVI-mediated activation cascade | 2 | LCK,RHOA |
| TRAF6 mediated induction of NFkB and MAP kinases upon TLR7/8 or 9 activation | 4 | PTPN11,RPS27A,-,UBE2N |
| MyD88 cascade initiated on plasma membrane | 4 | PTPN11,RPS27A,-,UBE2N |
| Transmission across Chemical Synapses | 8 | ALDH2,CALM1,GNB1,GNB2,GLUL,GNAI1,GNAI2,HSPA8 |
| EphrinA-EPHA pathway | 3 | GRB2,LCK,RHOA |
| Activation of Chaperones by IRE1alpha | 3 | C19orf10,LMNA,TLN1 |
| Apoptotic cleavage of cell adhesion proteins | 1 | SPTAN1 |
| Recruitment of NuMA to mitotic centrosomes | 1 | NUMA1 |
| "Gamma-carboxylation, transport, and amino-terminal cleavage of proteins" | 1 | F2 |
| Post-chaperonin tubulin folding pathway | 1 | TBCA |
| Peptide hormone biosynthesis | 1 | INHBB |
| Downstream signal transduction | 5 | YWHAB,CALM1,GRB2,PRKAR2B,PTPN11 |
| MyD88 dependent cascade initiated on endosome | 4 | PTPN11,RPS27A,-,UBE2N |
| Toll Like Receptor 5 (TLR5) Cascade | 4 | PTPN11,RPS27A,-,UBE2N |
| Toll Like Receptor 10 (TLR10) Cascade | 4 | PTPN11,RPS27A,-,UBE2N |
| Calcium signaling in the CD4+ TCR pathway | 2 | CALM1,FKBP1A |
| Triglyceride Biosynthesis | 2 | FASN,GPD1 |
| EGFR interacts with phospholipase C-gamma | 2 | CALM1,PRKAR2B |
| Regulation of beta-cell development | 7 | SLC2A2,RPLP0,RPLP1,RPS25,RPS27A,RPS3,RPSA |
| PLC-gamma1 signalling | 2 | CALM1,PRKAR2B |
| Reelin signaling pathway | 2 | PAFAH1B1,RAP1A |
| Netrin-1 signaling | 3 | PTPN11,RAP1A,TLN1 |
| pyrimidine deoxyribonucleotides de novo biosynthesis | 1 | NME2 |
| p75NTR recruits signalling complexes | 1 | RPS27A |
| NF-kB is activated and signals survival | 1 | RPS27A |
| Signaling by TGF beta | 1 | TGFBR1 |
| Regulation of IFNG signaling | 1 | PTPN11 |
| "Synthesis, Secretion, and Inactivation of Glucose-dependent Insulinotropic Polypeptide (GIP)" | 1 | GCG |
| guanosine nucleotides de novo biosynthesis | 1 | NME2 |
| TRAF6 mediated induction of TAK1 complex | 1 | RPS27A |
| Toll Like Receptor 7/8 (TLR7/8) Cascade | 4 | PTPN11,RPS27A,-,UBE2N |
| Signaling by PDGF | 5 | YWHAB,CALM1,GRB2,PRKAR2B,PTPN11 |
| TCR signaling | 3 | LCK,-,UBE2N |
| RNA Polymerase I Promoter Clearance | 2 | -,- |
| IL4-mediated signaling events | 4 | COL1A1,GRB2,IL4R,PIGR |
| Post-translational modification: gamma carboxylation and hypusine formation | 1 | F2 |
| salvage pathways of pyrimidine ribonucleotides | 1 | NME2 |
| Atypical NF-kappaB pathway | 1 | LCK |
| CREB phosphorylation through the activation of CaMKII | 1 | CALM1 |
| Prefoldin mediated transfer of substrate to CCT/TriC | 1 | CCT6A |
| NRIF signals cell death from the nucleus | 1 | RPS27A |
| SEMA3A-Plexin repulsion signaling by inhibiting Integrin adhesion | 1 | TLN1 |
| Validated targets of C-MYC transcriptional repression | 4 | CLU,FTH1,LGALS1,PPP2R4 |
| EPHA forward signaling | 2 | LCK,RHOA |
| Cyclin D associated events in G1 | 2 | CDKN2C,RPS27A |
| Circadian Clock | 2 | SERPINE1,RPS27A |
| G1 Phase | 2 | CDKN2C,RPS27A |
| GAB1 signalosome | 2 | GRB2,PTPN11 |
| Metabolism of steroid hormones and vitamins A and D | 2 | AKR1B1,GC |
| Regulation of Lipid Metabolism by Peroxisome proliferator-activated receptor alpha (PPARalpha) | 2 | FASN,SLC2A2 |
| Eicosanoid ligand-binding receptors | 1 | PTGDR2 |
| Association of licensing factors with the pre-replicative complex | 1 | RPS27A |
| Validated nuclear estrogen receptor beta network | 1 | C3 |
| Signaling by Aurora kinases | 6 | -,LCK,PEBP1,PSMA3,RHOA,VIM |
| Signaling by SCF-KIT | 4 | YWHAB,GRB2,MMP9,PTPN11 |
| Meiotic Synapsis | 2 | -,LMNA |
| p75NTR signals via NF-kB | 1 | RPS27A |
| Ras activation uopn Ca2+ infux through NMDA receptor | 1 | CALM1 |
| Glucagon-type ligand receptors | 1 | GCG |
| "Synthesis, Secretion, and Deacylation of Ghrelin" | 1 | GCG |
| Immunoregulatory interactions between a Lymphoid and a non-Lymphoid cell | 3 | B2M,CD81,C3 |
| Signaling events mediated by Stem cell factor receptor (c-Kit) | 3 | EPO,GRB2,PTPN11 |
| Signal transduction by L1 | 2 | CLTC,RHOA |
| HIV-1 Nef: Negative effector of Fas and TNF-alpha | 2 | CYCS,TRADD |
| CD28 dependent PI3K/Akt signaling | 1 | LCK |
| Destabilization of mRNA by Tristetraprolin (TTP) | 1 | YWHAB |
| Syndecan-3-mediated signaling events | 1 | CXCL8 |
| PKA activation in glucagon signalling | 1 | PRKAR2B |
| "GABA synthesis, release, reuptake and degradation" | 1 | HSPA8 |
| Degradation of beta catenin | 1 | LRP6 |
| The citric acid (TCA) cycle and respiratory electron transport | 7 | ATP5B,ATP5D,CS,COX6B1,CYCS,FH,MDH2 |
| Nonsense Mediated Decay Enhanced by the Exon Junction Complex | 6 | RPLP0,RPLP1,RPS25,RPS27A,RPS3,RPSA |
| Nonsense-Mediated Decay | 6 | RPLP0,RPLP1,RPS25,RPS27A,RPS3,RPSA |
| Neurotransmitter Receptor Binding And Downstream Transmission In The Postsynaptic Cell | 5 | CALM1,GNB1,GNB2,GNAI1,GNAI2 |
| "Synthesis, Secretion, and Inactivation of Glucagon-like Peptide-1 (GLP-1)" | 1 | GCG |
| Activated AMPK stimulates fatty-acid oxidation in muscle | 1 | ACACB |
| Fatty Acyl-CoA Biosynthesis | 1 | FASN |
| KitReceptor | 3 | CLTC,GRB2,PTPN11 |
| Iron uptake and transport | 2 | CP,TF |
| EGFR1 | 8 | YWHAB,CAV1,EPS15,GRB2,PEBP1,PLEC,PTPN11,SH3BGRL |
| Destabilization of mRNA by KSRP | 1 | YWHAZ |
| Generation of second messenger molecules | 1 | LCK |
| E2F transcription factor network | 4 | CDKN2C,CES1,SERPINE1,PLAU |
| Cellular roles of Anthrax toxin | 1 | CALM1 |
| Effects of PIP2 hydrolysis | 1 | MGLL |
| Peroxisomal lipid metabolism | 1 | IDH1 |
| Destabilization of mRNA by Butyrate Response Factor 1 (BRF1) | 1 | YWHAB |
| IL12-mediated signaling events | 6 | B2M,CCL3,CCL4L1,IL1R1,IL6,LCK |
| Cytokine Signaling in Immune system | 11 | YWHAB,YWHAZ,B2M,GRB2,IL1R1,IL6,PTPN11,RAP1A,TLN1,-,UBE2N |
| Hexose transport | 2 | SLC2A2,SLC2A5 |
| E-cadherin signaling in keratinocytes | 1 | RHOA |
| "Incretin Synthesis, Secretion, and Inactivation" | 1 | GCG |
| RNA Polymerase I Transcription Termination | 1 | PTRF |
| Interferon alpha/beta signaling | 4 | GRB2,PTPN11,RAP1A,TLN1 |
| Ras signaling in the CD4+ TCR pathway | 2 | CALM1,FKBP1A |
| JNK signaling in the CD4+ TCR pathway | 2 | CALM1,FKBP1A |
| Signaling by Insulin receptor | 4 | YWHAB,GRB2,EIF4B,PTPN11 |
| Deadenylation of mRNA | 1 | EIF4B |
| APC/C:Cdc20 mediated degradation of Cyclin B | 1 | RPS27A |
| Fanconi Anemia pathway | 1 | RPS27A |
| Deposition of New CENPA-containing Nucleosomes at the Centromere | 1 | - |
| Nucleosome assembly | 1 | - |
| Insulin Synthesis and Processing | 7 | GCG,RPLP0,RPLP1,RPS25,RPS27A,RPS3,RPSA |
| Androgen-mediated signaling | 7 | GNB1,GNB2L1,GSN,GNAI1,GNAI2,HSP90AA1,PRDX1 |
| mRNA Splicing - Minor Pathway | 2 | SNRPD1,SRSF7 |
| IL2-mediated signaling events | 6 | CALM1,GRB2,HSP90AA1,LCK,PTPN11,RHOA |
| TCR signaling in na&#xef;ve CD4+ T cells | 7 | CALM1,FKBP1A,FLNA,GRB2,LCK,PTPN11,RAP1A |
| Signaling by Robo receptor | 1 | CAP1 |
| BMAL1:CLOCK/NPAS2 Activates Gene Expression | 1 | SERPINE1 |
| NCAM1 interactions | 1 | NRTN |
| APC-Cdc20 mediated degradation of Nek2A | 1 | RPS27A |
| Interferon Signaling | 5 | B2M,GRB2,PTPN11,RAP1A,TLN1 |
| Cytosolic tRNA aminoacylation | 1 | PPA1 |
| Visual signal transduction: Rods | 1 | GNB1 |
| Regulation of Water Balance by Renal Aquaporins | 1 | PRKAR2B |
| Downstream signaling in na&#xef;ve CD8+ T cells | 3 | B2M,CALM1,TNFRSF18 |
| ABC-family proteins mediated transport | 1 | APOA1 |
| Metal ion SLC transporters | 1 | CP |
| Nephrin/Neph1 signaling in the kidney podocyte | 1 | GRB2 |
| Presenilin action in Notch and Wnt signaling | 2 | KREMEN2,LRP6 |
| C-MYB transcription factor network | 4 | CA1,HSPA8,IQGAP1,LYZ |
| M Phase | 8 | YWHAE,YWHAG,ACTR1A,DYNC1H1,HSP90AA1,PRKAR2B,PAFAH1B1,TUBB |
| Interferon gamma signaling | 2 | B2M,PTPN11 |
| IL27-mediated signaling events | 1 | IL6 |
| Neurotransmitter Release Cycle | 1 | HSPA8 |
| mTOR signalling | 1 | EIF4B |
| "RNA Polymerase I, RNA Polymerase III, and Mitochondrial Transcription" | 3 | -,-,PTRF |
| Calcineurin-regulated NFAT-dependent transcription in lymphocytes | 2 | CALM1,CXCL8 |
| Regulation of Telomerase | 3 | YWHAE,HNRNPC,HSP90AA1 |
| PKB-mediated events | 1 | EIF4B |
| Aquaporin-mediated transport | 1 | PRKAR2B |
| Endosomal Sorting Complex Required For Transport (ESCRT) | 1 | RPS27A |
| Bile acid and bile salt metabolism | 1 | ALB |
| Transferrin endocytosis and recycling | 1 | TF |
| CREB phosphorylation through the activation of Ras | 1 | CALM1 |
| Transcriptional Regulation of White Adipocyte Differentiation | 3 | ADIPOQ,FABP4,PLIN1 |
| Negative regulators of RIG-I/MDA5 signaling | 1 | RPS27A |
| mRNA Splicing - Major Pathway | 5 | HNRNPC,PCBP1,PTBP1,SNRPD1,SRSF7 |
| mRNA Splicing | 5 | HNRNPC,PCBP1,PTBP1,SNRPD1,SRSF7 |
| TRAF6 Mediated Induction of proinflammatory cytokines | 2 | PTPN11,RPS27A |
| TCR signaling in na&#xef;ve CD8+ T cells | 6 | B2M,CALM1,GRB2,LCK,RAP1A,TNFRSF18 |
| Meiotic Recombination | 1 | - |
| Transport of Ribonucleoproteins into the Host Nucleus | 1 | KPNB1 |
| "Respiratory electron transport, ATP synthesis by chemiosmotic coupling, and heat production by uncoupling proteins." | 4 | ATP5B,ATP5D,COX6B1,CYCS |
| Nuclear import of Rev protein | 1 | KPNB1 |
| PI3K/AKT activation | 1 | RHOA |
| "Transport of vitamins, nucleosides, and related molecules" | 1 | ALB |
| Export of Viral Ribonucleoproteins from Nucleus | 1 | HSPA1A |
| MAP kinase activation in TLR cascade | 1 | PTPN11 |
| NFkB and MAP kinases activation mediated by TLR4 signaling repertoire | 2 | PTPN11,RPS27A |
| Netrin-mediated signaling events | 1 | RHOA |
| TRIF mediated TLR3 signaling | 2 | PTPN11,RPS27A |
| Toll Like Receptor 3 (TLR3) Cascade | 2 | PTPN11,RPS27A |
| Post NMDA receptor activation events | 1 | CALM1 |
| Interactions of Rev with host cellular proteins | 1 | KPNB1 |
| Activation of NMDA receptor upon glutamate binding and postsynaptic events | 1 | CALM1 |
| Asparagine N-linked glycosylation | 3 | PRKCSH,PDIA3,UGGT1 |
| MyD88-independent cascade initiated on plasma membrane | 2 | PTPN11,RPS27A |
| mRNA 3'-end processing | 1 | SRSF7 |
| Trk receptor signaling mediated by the MAPK pathway | 1 | RAP1A |
| Post-Elongation Processing of Intron-Containing pre-mRNA | 1 | SRSF7 |
| Meiosis | 2 | -,LMNA |
| Canonical NF-kappaB pathway | 1 | LCK |
| Telomere Maintenance | 1 | - |
| Phase 1 - Functionalization of compounds | 2 | ALDH1A1,ALDH2 |
| Coregulation of Androgen receptor activity | 2 | GSN,PRDX1 |
| Fc-epsilon receptor I signaling in mast cells | 2 | GRB2,PTPN11 |
| PI3K Cascade | 1 | EIF4B |
| Glucose transport | 1 | SLC2A2 |
| Regulation of Androgen receptor activity | 4 | GNB2L1,GSN,HSP90AA1,PRDX1 |
| Neuronal System | 8 | ALDH2,CALM1,GNB1,GNB2,GLUL,GNAI1,GNAI2,HSPA8 |
| BCR signaling pathway | 2 | CALM1,GRB2 |
| tRNA Aminoacylation | 1 | PPA1 |
| Cleavage of Growing Transcript in the Termination Region | 1 | SRSF7 |
| RNA Polymerase II Transcription Termination | 1 | SRSF7 |
| Post-Elongation Processing of the Transcript | 1 | SRSF7 |
| Post-translational protein modification | 4 | PRKCSH,PDIA3,F2,UGGT1 |
| Gene Expression | 18 | EEF1D,EEF1G,EEF2,EIF3B,EIF3H,HNRNPC,EIF4B,PPA1,PCBP1,PTBP1,RPLP0,RPLP1,RPS25,RPS27A,RPS3,RPSA,SNRPD1,SRSF7 |
| Deadenylation-dependent mRNA decay | 1 | EIF4B |
| "Transport of glucose and other sugars, bile salts and organic acids, metal ions and amine compounds" | 3 | CP,SLC2A2,SLC2A5 |
| Processing of Capped Intron-Containing Pre-mRNA | 5 | HNRNPC,PCBP1,PTBP1,SNRPD1,SRSF7 |
| Class B/2 (Secretin family receptors) | 1 | GCG |
| Metabolism of non-coding RNA | 1 | SNRPD1 |
| snRNP Assembly | 1 | SNRPD1 |
| Potassium Channels | 2 | GNB1,GNB2 |
| Respiratory electron transport | 2 | COX6B1,CYCS |
| Transport of Mature mRNA derived from an Intron-Containing Transcript | 1 | SRSF7 |
| AndrogenReceptor | 2 | CAV1,FLNA |
| TGFBR | 4 | CAV1,FKBP1A,HSPA8,KPNB1 |
| PLK1 signaling events | 3 | LCK,RAB1A,RHOA |
| Wnt | 2 | LRP6,RHOA |
| Transport of Mature Transcript to Cytoplasm | 1 | SRSF7 |
| Chromosome Maintenance | 1 | - |
| Polo-like kinase signaling events in the cell cycle | 3 | LCK,RAB1A,RHOA |
| CD40/CD40L signaling | 1 | LCK |
| mRNA Processing | 5 | HNRNPC,PCBP1,PTBP1,SNRPD1,SRSF7 |
| Aurora A signaling | 1 | LCK |
| Notch-mediated HES/HEY network | 2 | ENO1,EPS15 |
| Notch signaling pathway | 2 | ENO1,EPS15 |
| Regulation of retinoblastoma protein | 1 | TGFB2 |
| RIG-I/MDA5 mediated induction of IFN-alpha/beta pathways | 1 | RPS27A |
| Transcription | 4 | -,-,PTRF,SRSF7 |
| Formation and Maturation of mRNA Transcript | 5 | HNRNPC,PCBP1,PTBP1,SNRPD1,SRSF7 |
| G alpha (q) signalling events | 2 | GNB1,MGLL |
| Mitotic Prometaphase | 1 | PAFAH1B1 |
| RNA Polymerase II Transcription | 1 | SRSF7 |
| HIV Life Cycle | 1 | PPIA |
| DNA Repair | 1 | RPS27A |
| Signaling by GPCR | 31 | ANXA1,CALM1,CCL2,CCL22,CCL27,CCL3,CCR4,CCR7,CCR9,C3,C5,CXCL9,CXCR1,CXCR2,CXCR5,CXCR6,CXCL11,GNB1,GCG,GNAI1,GNAI2,HEBP1,CXCL8,PRKAR2B,MGLL,HCRT,PTGDR2,PPP1CA,PROK1,RHOA,F2 |
| SLC-mediated transmembrane transport | 4 | ALB,CP,SLC2A2,SLC2A5 |
| Transmembrane transport of small molecules | 7 | ALB,APOA1,CP,SLC2A2,SLC2A5,PRKAR2B,TF |
| Olfactory Signaling Pathway | 1 | GNB1 |
| GPCR downstream signaling | 5 | GNB1,GNAI1,GNAI2,MGLL,RHOA |
