# Supplementary material for: Secretome analysis of breast cancer-associated adipose tissue to identify paracrine regulators of breast cancer growth
Source: Oncotarget. 2017 May 3;8(29):47239–49. doi: 10.18632/oncotarget.17592 (PMC5564561; doi:10.18632/oncotarget.17592)
Supplement: Supplementary file 4 [file oncotarget-08-47239-s004.docx]

**Supplementary Table 3: Table representing the common proteins between breast cancer-associated adipose tissue (BCAAT) and the proteomics data of Alvarez-Llamas G et al, Lehr S et al and Xie X et al.**

| **Accession Number** | **UniProt Nr** | **Identified Proteins Human Breast Cancer-Associated Adipose Tissue (BCAAT)** | **Alvarez-Llamas G et al.** | **Lehr S et al.** | **Xie X et al.** |
| --- | --- | --- | --- | --- | --- |
| 1433B_HUMAN | P31946 | 14-3-3 protein beta/alpha |  |  |  |
| 1433E_HUMAN | P62258 | 14-3-3 protein epsilon | + |  | + |
| 1433G_HUMAN | P61981 | 14-3-3 protein gamma | + | + | + |
| 1433T_HUMAN | P27348 | 14-3-3 protein theta |  |  | + |
| 1433Z_HUMAN | P63104 | 14-3-3 protein zeta/delta | + | + | + |
| 6PGD_HUMAN | P52209 | 6-phosphogluconate dehydrogenase, decarboxylating | + | + | + |
| 6PGL_HUMAN | O95336 | 6-phosphogluconolactonase | + | + | + |
| A1AG1_HUMAN | P02763 | Alpha-1-acid glycoprotein 1 |  |  |  |
| A1AG2_HUMAN | P19652 | Alpha-1-acid glycoprotein 2 |  |  |  |
| A1AT_HUMAN | P01009 | Alpha-1-antitrypsin |  | + |  |
| A1BG_HUMAN | P04217 | Alpha-1B-glycoprotein |  |  |  |
| A2GL_HUMAN | P02750 | Leucine-rich alpha-2-glycoprotein |  |  |  |
| A2MG_HUMAN | P01023 | Alpha-2-macroglobulin | + | + |  |
| A6PVI2_HUMAN | A6PVI2 | Angiostatin |  |  |  |
| AATC_HUMAN | P17174 | Aspartate aminotransferase, cytoplasmic | + | + | + |
| ABHEB_HUMAN | Q96IU4 | Alpha/beta hydrolase domain-containing protein 14B |  |  |  |
| ACACB_HUMAN | O00763 | Acetyl-CoA carboxylase 2 |  |  |  |
| ACBP_HUMAN | P07108 | Acyl-CoA-binding protein |  | + |  |
| ACOC_HUMAN | P21399 | Cytoplasmic aconitate hydratase |  |  | + |
| ACOT1_HUMAN | Q86TX2 | Acyl-coenzyme A thioesterase 1 |  |  | + |
| ACTB_HUMAN | P60709 | Actin, cytoplasmic 1 | + | + | + |
| ACTN1_HUMAN | P12814 | Alpha-actinin-1 | + | + | + |
| ACTN4_HUMAN | O43707 | Alpha-actinin-4 | + |  | + |
| ACTS_HUMAN | P68133 | Actin, alpha skeletal muscle |  |  |  |
| ACTZ_HUMAN | P61163 | Alpha-centractin |  |  | + |
| ACY1_HUMAN | Q03154 | Aminoacylase-1 | + |  | + |
| ACYP2_HUMAN | P14621 | Acylphosphatase-2 |  |  | + |
| ADH1A_HUMAN | P07327 | Alcohol dehydrogenase 1A |  |  |  |
| ADH1B_HUMAN | P00325 | Alcohol dehydrogenase 1B |  |  | + |
| ADHX_HUMAN | P11766 | Alcohol dehydrogenase class-3 | + |  | + |
| ADIPO_HUMAN | Q15848 | Adiponectin | + | + | + |
| ADIRF_HUMAN | Q15847 | Adipogenesis regulatory factor |  |  | + |
| ADK_HUMAN | P55263 | Adenosine kinase |  |  |  |
| AFAM_HUMAN | P43652 | Afamin |  |  |  |
| AHNK_HUMAN | Q09666 | Neuroblast differentiation-associated protein AHNAK |  |  | + |
| AIFM2_HUMAN | Q9BRQ8 | Apoptosis-inducing factor 2 |  |  |  |
| AK1A1_HUMAN | P14550 | Alcohol dehydrogenase [NADP(+)] |  | + | + |
| AK1C1_HUMAN | Q04828 | Aldo-keto reductase family 1 member C1 |  | + |  |
| AK1C3_HUMAN | P42330 | Aldo-keto reductase family 1 member C3 |  | + | + |
| AKA12_HUMAN | Q02952 | A-kinase anchor protein 12 |  |  |  |
| AL1A1_HUMAN | P00352 | Retinal dehydrogenase 1 | + |  | + |
| AL1L1_HUMAN | O75891 | Cytosolic 10-formyltetrahydrofolate dehydrogenase |  |  | + |
| ALBU_HUMAN | P02768 | Serum albumin | + | + |  |
| ALDH2_HUMAN | P05091 | Aldehyde dehydrogenase, mitochondrial |  |  | + |
| ALDOA_HUMAN | P04075 | Fructose-bisphosphate aldolase A | + | + | + |
| ALDOC_HUMAN | P09972 | Fructose-bisphosphate aldolase C | + |  |  |
| ALDR_HUMAN | P15121 | Aldose reductase |  | + | + |
| AMBP_HUMAN | P02760 | Protein AMBP |  |  |  |
| AMPL_HUMAN | P28838 | Cytosol aminopeptidase | + |  |  |
| AN32B_HUMAN | Q92688 | Acidic leucine-rich nuclear phosphoprotein 32 family member B |  |  |  |
| ANGL1_HUMAN | O95841 | Angiopoietin-related protein 1 |  |  |  |
| ANGP1_HUMAN | Q15389 | Angiopoietin-1 |  |  |  |
| ANGP2_HUMAN | O15123 | Angiopoietin-2 |  |  |  |
| ANXA1_HUMAN | P04083 | Annexin A1 |  | + | + |
| ANXA2_HUMAN | P07355 | Annexin A2 | + | + | + |
| ANXA4_HUMAN | P09525 | Annexin A4 |  |  | + |
| ANXA5_HUMAN | P08758 | Annexin A5 |  |  | + |
| AOC3_HUMAN | Q16853 | Membrane primary amine oxidase |  |  | + |
| AP1B1_HUMAN | Q10567 | AP-1 complex subunit beta-1 |  |  |  |
| APOA1_HUMAN | P02647 | Apolipoprotein A-I |  |  | + |
| APOA2_HUMAN | P02652 | Apolipoprotein A-II |  |  | + |
| APOA4_HUMAN | P06727 | Apolipoprotein A-IV |  |  |  |
| APOB_HUMAN | P04114 | Apolipoprotein B-100 |  |  |  |
| APOC3_HUMAN | P02656 | Apolipoprotein C-III |  |  |  |
| APOD_HUMAN | P05090 | Apolipoprotein D |  | + |  |
| APOE_HUMAN | P02649 | Apolipoprotein E |  | + | + |
| APOH_HUMAN | P02749 | Beta-2-glycoprotein 1 |  |  |  |
| APT_HUMAN | P07741 | Adenine phosphoribosyltransferase |  |  | + |
| ARF3_HUMAN | P61204 | ADP-ribosylation factor 3 |  |  |  |
| ARK72_HUMAN | O43488 | Aflatoxin B1 aldehyde reductase member 2 |  |  | + |
| ARP3_HUMAN | P61158 | Actin-related protein 3 |  |  | + |
| ARPC2_HUMAN | O15144 | Actin-related protein 2/3 complex subunit 2 |  |  | + |
| ARPC3_HUMAN | O15145 | Actin-related protein 2/3 complex subunit 3 |  |  | + |
| ARPC4_HUMAN | P59998 | Actin-related protein 2/3 complex subunit 4 |  | + | + |
| ASAH1_HUMAN | Q13510 | Acid ceramidase |  | + | + |
| ASC_HUMAN | Q9ULZ3 | Apoptosis-associated speck-like protein containing a CARD |  |  |  |
| AT2L2_HUMAN | Q8IUZ5 | 5-phosphohydroxy-L-lysine phospho-lyase |  |  |  |
| ATPB_HUMAN | P06576 | ATP synthase subunit beta, mitochondrial |  | + | + |
| ATPD_HUMAN | P30049 | ATP synthase subunit delta, mitochondrial |  |  | + |
| ATRN_HUMAN | O75882 | Attractin |  |  |  |
| AVR2A_HUMAN | P27037 | Activin receptor type-2A |  |  |  |
| B2MG_HUMAN | P61769 | Beta-2-microglobulin |  | + | + |
| BAX_HUMAN | Q07812 | Apoptosis regulator BAX |  | + |  |
| BDNF_HUMAN | P23560 | Brain-derived neurotrophic factor |  |  |  |
| BLVRB_HUMAN | P30043 | Flavin reductase (NADPH) |  | + | + |
| BMP4_HUMAN | P12644 | Bone morphogenetic protein 4 |  |  |  |
| BMP8B_HUMAN | P34820 | Bone morphogenetic protein 8B |  |  |  |
| BMR1B_HUMAN | O00238 | Bone morphogenetic protein receptor type-1B |  |  |  |
| BPNT1_HUMAN | O95861 | 3'(2'),5'-bisphosphate nucleotidase 1 |  | + |  |
| C163A_HUMAN | Q86VB7 | Scavenger receptor cysteine-rich type 1 protein M130 |  |  |  |
| C1QA_HUMAN | P02745 | Complement C1q subcomponent subunit A |  |  |  |
| C1QB_HUMAN | P02746 | Complement C1q subcomponent subunit B |  | + |  |
| C1QC_HUMAN | P02747 | Complement C1q subcomponent subunit C |  |  |  |
| C1R_HUMAN | P00736 | Complement C1r subcomponent |  | + |  |
| C1S_HUMAN | P09871 | Complement C1s subcomponent | + | + |  |
| C1TC_HUMAN | P11586 | C-1-tetrahydrofolate synthase, cytoplasmic |  |  | + |
| CAH1_HUMAN | P00915 | Carbonic anhydrase 1 | + |  | + |
| CAH2_HUMAN | P00918 | Carbonic anhydrase 2 |  | + | + |
| CALB2_HUMAN | P22676 | Calretinin | + | + | + |
| CALM_HUMAN | P62158 | Calmodulin |  | + | + |
| CAND1_HUMAN | Q86VP6 | Cullin-associated NEDD8-dissociated protein 1 |  |  |  |
| CAP1_HUMAN | Q01518 | Adenylyl cyclase-associated protein 1 |  | + |  |
| CAPG_HUMAN | P40121 | Macrophage-capping protein |  | + | + |
| CAPZB_HUMAN | P47756 | F-actin-capping protein subunit beta |  |  |  |
| CATC_HUMAN | P53634 | Dipeptidyl peptidase 1 |  |  |  |
| CATD_HUMAN | P07339 | Cathepsin D | + | + | + |
| CATZ_HUMAN | Q9UBR2 | Cathepsin Z | + | + |  |
| CAV1_HUMAN | Q03135 | Caveolin-1 |  |  |  |
| CAZA1_HUMAN | P52907 | F-actin-capping protein subunit alpha-1 |  |  | + |
| CAZA2_HUMAN | P47755 | F-actin-capping protein subunit alpha-2 |  |  | + |
| CBR1_HUMAN | P16152 | Carbonyl reductase [NADPH] 1 |  |  | + |
| CCL2_HUMAN | P13500 | C-C motif chemokine 2 |  |  |  |
| CCL22_HUMAN | O00626 | C-C motif chemokine 22 |  |  |  |
| CCL27_HUMAN | Q9Y4X3 | C-C motif chemokine 27 |  |  |  |
| CCL3_HUMAN | P10147 | C-C motif chemokine 3 |  |  |  |
| CCL4_HUMAN | P13236 | C-C motif chemokine 4 |  |  |  |
| CCR2_HUMAN | P41597 | C-C chemokine receptor type 2 |  |  |  |
| CCR4_HUMAN | P51679 | C-C chemokine receptor type 4 |  |  |  |
| CCR7_HUMAN | P32248 | C-C chemokine receptor type 7 |  |  |  |
| CCR8_HUMAN | P51685 | C-C chemokine receptor type 8 |  |  |  |
| CCR9_HUMAN | P51686 | C-C chemokine receptor type 9 |  |  |  |
| CD248_HUMAN | Q9HCU0 | Endosialin |  | + |  |
| CD5L_HUMAN | O43866 | CD5 antigen-like |  |  |  |
| CD81_HUMAN | P60033 | CD81 antigen |  |  | + |
| CDN2C_HUMAN | P42773 | Cyclin-dependent kinase 4 inhibitor C |  |  | + |
| CERU_HUMAN | P00450 | Ceruloplasmin |  |  |  |
| CFAB_HUMAN | P00751 | Complement factor B | + | + |  |
| CFAD_HUMAN | P00746 | Complement factor D | + | + |  |
| CFAH_HUMAN | P08603 | Complement factor H |  | + |  |
| CFAI_HUMAN | P05156 | Complement factor I |  |  |  |
| CH10_HUMAN | P61604 | 10 kDa heat shock protein, mitochondrial |  | + | + |
| CISY_HUMAN | O75390 | Citrate synthase, mitochondrial | + | + | + |
| CK054_HUMAN | Q9H0W9 | Ester hydrolase C11orf54 |  |  |  |
| CLH1_HUMAN | Q00610 | Clathrin heavy chain 1 |  |  |  |
| CLIC1_HUMAN | O00299 | Chloride intracellular channel protein 1 |  |  | + |
| CLIC4_HUMAN | Q9Y696 | Chloride intracellular channel protein 4 |  |  | + |
| CLUS_HUMAN | P10909 | Clusterin | + | + |  |
| CNDP2_HUMAN | Q96KP4 | Cytosolic non-specific dipeptidase |  |  |  |
| CNTF_HUMAN | P26441 | Ciliary neurotrophic factor |  |  |  |
| CO1A1_HUMAN | P02452 | Collagen alpha-1(I) chain | + | + |  |
| CO3_HUMAN | P01024 | Complement C3 | + | + |  |
| CO4A_HUMAN | P0C0L4 | Complement C4-A | + | + |  |
| CO5_HUMAN | P01031 | Complement C5 |  |  |  |
| CO6A1_HUMAN | P12109 | Collagen alpha-1(VI) chain | + | + | + |
| CO6A3_HUMAN | P12111 | Collagen alpha-3(VI) chain | + | + |  |
| CO7_HUMAN | P10643 | Complement component C7 | + | + |  |
| CO8G_HUMAN | P07360 | Complement component C8 gamma chain |  |  |  |
| CO9_HUMAN | P02748 | Complement component C9 |  |  |  |
| COEA1_HUMAN | Q05707 | Collagen alpha-1(XIV) chain | + |  |  |
| COF1_HUMAN | P23528 | Cofilin-1 |  | + | + |
| COF2_HUMAN | Q9Y281 | Cofilin-2 |  |  | + |
| COFA1_HUMAN | P39059 | Collagen alpha-1(XV) chain | + | + |  |
| COIA1_HUMAN | P39060 | Collagen alpha-1(XVIII) chain | + | + |  |
| COR1C_HUMAN | Q9ULV4 | Coronin-1C |  |  | + |
| COTL1_HUMAN | Q14019 | Coactosin-like protein |  |  | + |
| CPN2_HUMAN | P22792 | Carboxypeptidase N subunit 2 |  |  |  |
| CPPED_HUMAN | Q9BRF8 | Serine/threonine-protein phosphatase CPPED1 |  | + |  |
| CRDL2_HUMAN | Q6WN34 | Chordin-like protein 2 |  |  |  |
| CRYAB_HUMAN | P02511 | Alpha-crystallin B chain |  | + | + |
| CS010_HUMAN | Q969H8 | UPF0556 protein C19orf10 |  | + | + |
| CSF1_HUMAN | P09603 | Macrophage colony-stimulating factor 1 | + | + |  |
| CSN4_HUMAN | Q9BT78 | COP9 signalosome complex subunit 4 |  |  | + |
| CSN7A_HUMAN | Q9UBW8 | COP9 signalosome complex subunit 7a |  |  | + |
| CSN8_HUMAN | Q99627 | COP9 signalosome complex subunit 8 |  |  | + |
| CSPG4_HUMAN | Q6UVK1 | Chondroitin sulfate proteoglycan 4 |  | + | + |
| CUTA_HUMAN | O60888 | Protein CutA |  | + |  |
| CX6B1_HUMAN | P14854 | Cytochrome c oxidase subunit 6B1 |  |  | + |
| CXCL2_HUMAN | P19875 | C-X-C motif chemokine 2 |  |  |  |
| CXCL9_HUMAN | Q07325 | C-X-C motif chemokine 9 |  |  |  |
| CXCR1_HUMAN | P25024 | C-X-C chemokine receptor type 1 |  |  |  |
| CXCR2_HUMAN | P25025 | C-X-C chemokine receptor type 2 |  |  |  |
| CXCR4_HUMAN | P61073 | C-X-C chemokine receptor type 4 |  |  |  |
| CXCR5_HUMAN | P32302 | C-X-C chemokine receptor type 5 |  |  |  |
| CXCR6_HUMAN | O00574 | C-X-C chemokine receptor type 6 |  |  |  |
| CXL11_HUMAN | O14625 | C-X-C motif chemokine 11 |  |  |  |
| CXL14_HUMAN | O95715 | C-X-C motif chemokine 14 |  |  |  |
| CYB5_HUMAN | P00167 | Cytochrome b5 |  |  |  |
| CYC_HUMAN | P99999 | Cytochrome c | + | + | + |
| CYTB_HUMAN | P04080 | Cystatin-B | + | + | + |
| DBLOH_HUMAN | Q9NR28 | Diablo homolog, mitochondrial |  |  |  |
| DCXR_HUMAN | Q7Z4W1 | L-xylulose reductase |  |  | + |
| DDAH2_HUMAN | O95865 | N(G),N(G)-dimethylarginine dimethylaminohydrolase 2 |  | + | + |
| DECR_HUMAN | Q16698 | 2,4-dienoyl-CoA reductase, mitochondrial |  |  | + |
| DERM_HUMAN | Q07507 | Dermatopontin | + | + | + |
| DHPR_HUMAN | P09417 | Dihydropteridine reductase | + |  | + |
| DKK4_HUMAN | Q9UBT3 | Dickkopf-related protein 4 |  |  |  |
| DLRB1_HUMAN | Q9NP97 | Dynein light chain roadblock-type 1 |  |  |  |
| DOPD_HUMAN | P30046 | D-dopachrome decarboxylase |  | + | + |
| DUS3_HUMAN | P51452 | Dual specificity protein phosphatase 3 |  |  | + |
| DYHC1_HUMAN | Q14204 | Cytoplasmic dynein 1 heavy chain 1 |  |  | + |
| DYL2_HUMAN | Q96FJ2 | Dynein light chain 2, cytoplasmic |  |  |  |
| ECH1_HUMAN | Q13011 | Delta(3,5)-Delta(2,4)-dienoyl-CoA isomerase, mitochondrial |  | + | + |
| ECHD1_HUMAN | Q9NTX5 | Ethylmalonyl-CoA decarboxylase |  |  |  |
| ECHM_HUMAN | P30084 | Enoyl-CoA hydratase, mitochondrial |  |  | + |
| ECI1_HUMAN | P42126 | Enoyl-CoA delta isomerase 1, mitochondrial |  | + |  |
| EEA1_HUMAN | Q15075 | Early endosome antigen 1 |  |  | + |
| EF1A3_HUMAN | Q5VTE0 | Putative elongation factor 1-alpha-like 3 |  |  | + |
| EF1D_HUMAN | P29692 | Elongation factor 1-delta |  |  | + |
| EF1G_HUMAN | P26641 | Elongation factor 1-gamma |  |  | + |
| EF2_HUMAN | P13639 | Elongation factor 2 |  |  | + |
| EHD2_HUMAN | Q9NZN4 | EH domain-containing protein 2 | + |  | + |
| EIF3B_HUMAN | P55884 | Eukaryotic translation initiation factor 3 subunit B |  |  |  |
| EIF3H_HUMAN | O15372 | Eukaryotic translation initiation factor 3 subunit H |  |  |  |
| ELTD1_HUMAN | Q9HBW9 | EGF, latrophilin and seven transmembrane domain-containing protein 1 |  |  |  |
| ENOA_HUMAN | P06733 | Alpha-enolase |  | + |  |
| ENOB_HUMAN | P13929 | Beta-enolase |  |  |  |
| ENPL_HUMAN | P14625 | Endoplasmin | + |  | + |
| EPO_HUMAN | P01588 | Erythropoietin |  |  |  |
| EPS15_HUMAN | P42566 | Epidermal growth factor receptor substrate 15 |  |  |  |
| ERAP1_HUMAN | Q9NZ08 | Endoplasmic reticulum aminopeptidase 1 |  | + |  |
| ERBB4_HUMAN | Q15303 | Receptor tyrosine-protein kinase erbB-4 |  |  |  |
| ERP29_HUMAN | P30040 | Endoplasmic reticulum resident protein 29 |  |  | + |
| ES1_HUMAN | P30042 | ES1 protein homolog, mitochondrial |  |  |  |
| EST1_HUMAN | P23141 | Liver carboxylesterase 1 | + | + |  |
| ESYT1_HUMAN | Q9BSJ8 | Extended synaptotagmin-1 |  |  |  |
| F13A_HUMAN | P00488 | Coagulation factor XIII A chain |  |  | + |
| FA49B_HUMAN | Q9NUQ9 | Protein FAM49B | + |  | + |
| FAAA_HUMAN | P16930 | Fumarylacetoacetase | + | + | + |
| FABP4_HUMAN | P15090 | Fatty acid-binding protein, adipocyte | + | + | + |
| FABP5_HUMAN | Q01469 | Fatty acid-binding protein, epidermal | + | + | + |
| FAS_HUMAN | P49327 | Fatty acid synthase | + | + | + |
| FBLN1_HUMAN | P23142 | Fibulin-1 | + | + |  |
| FBLN2_HUMAN | P98095 | Fibulin-2 |  | + |  |
| FERM2_HUMAN | Q96AC1 | Fermitin family homolog 2 |  |  |  |
| FETA_HUMAN | P02771 | Alpha-fetoprotein |  | + |  |
| FETUA_HUMAN | P02765 | Alpha-2-HS-glycoprotein |  |  |  |
| FGF11_HUMAN | Q92914 | Fibroblast growth factor 11 |  |  |  |
| FGF16_HUMAN | O43320 | Fibroblast growth factor 16 |  |  |  |
| FGF2_HUMAN | P09038 | Fibroblast growth factor 2 |  |  |  |
| FGF20_HUMAN | Q9NP95 | Fibroblast growth factor 20 |  |  |  |
| FGF23_HUMAN | Q9GZV9 | Fibroblast growth factor 23 |  |  |  |
| FGF9_HUMAN | P31371 | Fibroblast growth factor 9 |  |  |  |
| FGFR4_HUMAN | P22455 | Fibroblast growth factor receptor 4 |  |  |  |
| FHL1_HUMAN | Q13642 | Four and a half LIM domains protein 1 |  |  |  |
| FIBA_HUMAN | P02671 | Fibrinogen alpha chain |  |  |  |
| FIBB_HUMAN | P02675 | Fibrinogen beta chain |  |  |  |
| FIBG_HUMAN | P02679 | Fibrinogen gamma chain |  |  |  |
| FINC_HUMAN | P02751 | Fibronectin | + | + |  |
| FIS1_HUMAN | Q9Y3D6 | Mitochondrial fission 1 protein |  |  | + |
| FKB1A_HUMAN | P62942 | Peptidyl-prolyl cis-trans isomerase FKBP1A |  | + | + |
| FLNA_HUMAN | P21333 | Filamin-A | + |  |  |
| FLNB_HUMAN | O75369 | Filamin-B |  |  |  |
| FRIH_HUMAN | P02794 | Ferritin heavy chain |  |  | + |
| FRIL_HUMAN | P02792 | Ferritin light chain |  |  | + |
| FST_HUMAN | P19883 | Follistatin |  |  |  |
| FSTL1_HUMAN | Q12841 | Follistatin-related protein 1 |  | + |  |
| FUMH_HUMAN | P07954 | Fumarate hydratase, mitochondrial | + | + |  |
| G3P_HUMAN | P04406 | Glyceraldehyde-3-phosphate dehydrogenase |  | + | + |
| G6PI_HUMAN | P06744 | Glucose-6-phosphate isomerase | + | + | + |
| GAPR1_HUMAN | Q9H4G4 | Golgi-associated plant pathogenesis-related protein 1 |  |  | + |
| GBB1_HUMAN | P62873 | Guanine nucleotide-binding protein G(I)/G(S)/G(T) subunit beta-1 |  |  | + |
| GBB2_HUMAN | P62879 | Guanine nucleotide-binding protein G(I)/G(S)/G(T) subunit beta-2 |  |  |  |
| GBLP_HUMAN | P63244 | Guanine nucleotide-binding protein subunit beta-2-like 1 |  |  | + |
| GDF15_HUMAN | Q99988 | Growth/differentiation factor 15 |  |  |  |
| GDIB_HUMAN | P50395 | Rab GDP dissociation inhibitor beta | + | + | + |
| GDIR1_HUMAN | P52565 | Rho GDP-dissociation inhibitor 1 | + | + | + |
| GDIR2_HUMAN | P52566 | Rho GDP-dissociation inhibitor 2 |  |  | + |
| GELS_HUMAN | P06396 | Gelsolin | + | + |  |
| GFRA2_HUMAN | O00451 | GDNF family receptor alpha-2 |  |  |  |
| GFRP_HUMAN | P30047 | GTP cyclohydrolase 1 feedback regulatory protein |  |  | + |
| GGCT_HUMAN | O75223 | Gamma-glutamylcyclotransferase |  | + |  |
| GLNA_HUMAN | P15104 | Glutamine synthetase |  |  | + |
| GLOD4_HUMAN | Q9HC38 | Glyoxalase domain-containing protein 4 |  |  |  |
| GLRX1_HUMAN | P35754 | Glutaredoxin-1 |  | + | + |
| GLU2B_HUMAN | P14314 | Glucosidase 2 subunit beta |  |  | + |
| GLUC_HUMAN | P01275 | Glucagon |  |  |  |
| GNAI1_HUMAN | P63096 | Guanine nucleotide-binding protein G(i) subunit alpha-1 |  |  | + |
| GNAI2_HUMAN | P04899 | Guanine nucleotide-binding protein G(i) subunit alpha-2 |  |  |  |
| GNPI1_HUMAN | P46926 | Glucosamine-6-phosphate isomerase 1 |  |  | + |
| GPC5_HUMAN | P78333 | Glypican-5 |  |  |  |
| GPD1L_HUMAN | Q8N335 | Glycerol-3-phosphate dehydrogenase 1-like protein |  |  | + |
| GPDA_HUMAN | P21695 | Glycerol-3-phosphate dehydrogenase [NAD(+)], cytoplasmic | + | + | + |
| GPNMB_HUMAN | Q14956 | Transmembrane glycoprotein NMB |  | + |  |
| GPX3_HUMAN | P22352 | Glutathione peroxidase 3 |  |  |  |
| GRB2_HUMAN | P62993 | Growth factor receptor-bound protein 2 |  |  |  |
| GRHPR_HUMAN | Q9UBQ7 | Glyoxylate reductase/hydroxypyruvate reductase |  |  | + |
| GROA_HUMAN | P09341 | Growth-regulated alpha protein |  |  |  |
| GSTM3_HUMAN | P21266 | Glutathione S-transferase Mu 3 |  |  | + |
| GSTO1_HUMAN | P78417 | Glutathione S-transferase omega-1 | + | + | + |
| GSTP1_HUMAN | P09211 | Glutathione S-transferase P | + | + | + |
| GSTT1_HUMAN | P30711 | Glutathione S-transferase theta-1 | + |  | + |
| GTR2_HUMAN | P11168 | Solute carrier family 2, facilitated glucose transporter member 2 |  |  |  |
| GTR5_HUMAN | P22732 | Solute carrier family 2, facilitated glucose transporter member 5 |  |  |  |
| H2A2C_HUMAN | Q16777 | Histone H2A type 2-C |  |  | + |
| H2B1D_HUMAN | P58876 | Histone H2B type 1-D |  |  |  |
| H33_HUMAN | P84243 | Histone H3.3 |  |  | + |
| H4_HUMAN | P62805 | Histone H4 | + |  | + |
| HBA_HUMAN | P69905 | Hemoglobin subunit alpha | + |  | + |
| HBB_HUMAN | P68871 | Hemoglobin subunit beta | + |  | + |
| HBD_HUMAN | P02042 | Hemoglobin subunit delta |  |  | + |
| HBEGF_HUMAN | Q99075 | Proheparin-binding EGF-like growth factor |  |  |  |
| HBG1_HUMAN | P69891 | Hemoglobin subunit gamma-1 |  |  |  |
| HCDH_HUMAN | Q16836 | Hydroxyacyl-coenzyme A dehydrogenase, mitochondrial |  |  |  |
| HDHD2_HUMAN | Q9H0R4 | Haloacid dehalogenase-like hydrolase domain-containing protein 2 |  |  |  |
| HEBP1_HUMAN | Q9NRV9 | Heme-binding protein 1 |  |  | + |
| HEBP2_HUMAN | Q9Y5Z4 | Heme-binding protein 2 |  | + |  |
| HEM2_HUMAN | P13716 | Delta-aminolevulinic acid dehydratase | + |  | + |
| HEMO_HUMAN | P02790 | Hemopexin |  |  |  |
| HIBCH_HUMAN | Q6NVY1 | 3-hydroxyisobutyryl-CoA hydrolase, mitochondrial |  |  |  |
| HINT2_HUMAN | Q9BX68 | Histidine triad nucleotide-binding protein 2, mitochondrial |  |  | + |
| HNMT_HUMAN | P50135 | Histamine N-methyltransferase |  |  | + |
| HNRPC_HUMAN | P07910 | Heterogeneous nuclear ribonucleoproteins C1/C2 |  |  |  |
| HPT_HUMAN | P00738 | Haptoglobin |  | + | + |
| HPTR_HUMAN | P00739 | Haptoglobin-related protein |  |  |  |
| HRG_HUMAN | P04196 | Histidine-rich glycoprotein |  |  |  |
| HS90A_HUMAN | P07900 | Heat shock protein HSP 90-alpha |  |  |  |
| HS90B_HUMAN | P08238 | Heat shock protein HSP 90-beta | + |  | + |
| HSP71_HUMAN | P08107 | Heat shock 70 kDa protein 1A/1B | + | + | + |
| HSP7C_HUMAN | P11142 | Heat shock cognate 71 kDa protein | + | + |  |
| HSPB1_HUMAN | P04792 | Heat shock protein beta-1 |  | + | + |
| HSPB6_HUMAN | O14558 | Heat shock protein beta-6 |  | + | + |
| HV305_HUMAN | P01766 | Ig heavy chain V-III region BRO |  |  |  |
| HV310_HUMAN | P01771 | Ig heavy chain V-III region HIL |  |  |  |
| HYEP_HUMAN | P07099 | Epoxide hydrolase 1 |  |  | + |
| HYI_HUMAN | Q5T013 | Putative hydroxypyruvate isomerase |  |  |  |
| I15RA_HUMAN | Q13261 | Interleukin-15 receptor subunit alpha |  |  |  |
| I17RD_HUMAN | Q8NFM7 | Interleukin-17 receptor D |  |  |  |
| I20RB_HUMAN | Q6UXL0 | Interleukin-20 receptor subunit beta |  |  |  |
| I36RA_HUMAN | Q9UBH0 | Interleukin-36 receptor antagonist protein |  |  |  |
| IBP3_HUMAN | P17936 | Insulin-like growth factor-binding protein 3 |  |  |  |
| IBP6_HUMAN | P24592 | Insulin-like growth factor-binding protein 6 |  |  |  |
| IBP7_HUMAN | Q16270 | Insulin-like growth factor-binding protein 7 | + | + |  |
| IC1_HUMAN | P05155 | Plasma protease C1 inhibitor | + | + |  |
| ICAM5_HUMAN | Q9UMF0 | Intercellular adhesion molecule 5 |  |  |  |
| IDHC_HUMAN | O75874 | Isocitrate dehydrogenase [NADP] cytoplasmic | + | + | + |
| IF4B_HUMAN | P23588 | Eukaryotic translation initiation factor 4B |  |  |  |
| IFNL1_HUMAN | Q8IU54 | Interferon lambda-1 |  |  |  |
| IGF2_HUMAN | P01344 | Insulin-like growth factor II |  |  |  |
| IGHA1_HUMAN | P01876 | Ig alpha-1 chain C region |  | + |  |
| IGHG1_HUMAN | P01857 | Ig gamma-1 chain C region | + | + | + |
| IGHG2_HUMAN | P01859 | Ig gamma-2 chain C region |  | + |  |
| IGHG3_HUMAN | P01860 | Ig gamma-3 chain C region |  |  |  |
| IGHG4_HUMAN | P01861 | Ig gamma-4 chain C region |  |  |  |
| IGHM_HUMAN | P01871 | Ig mu chain C region | + | + |  |
| IGJ_HUMAN | P01591 | Immunoglobulin J chain |  | + |  |
| IGKC_HUMAN | P01834 | Ig kappa chain C region | + | + | + |
| IGLL5_HUMAN | B9A064 | Immunoglobulin lambda-like polypeptide 5 |  |  |  |
| IL17B_HUMAN | Q9UHF5 | Interleukin-17B |  |  |  |
| IL17C_HUMAN | Q9P0M4 | Interleukin-17C |  |  |  |
| IL17D_HUMAN | Q8TAD2 | Interleukin-17D |  |  |  |
| IL17F_HUMAN | Q96PD4 | Interleukin-17F |  |  |  |
| IL19_HUMAN | Q9UHD0 | Interleukin-19 |  |  |  |
| IL1R1_HUMAN | P14778 | Interleukin-1 receptor type 1 |  |  |  |
| IL22_HUMAN | Q9GZX6 | Interleukin-22 |  |  |  |
| IL26_HUMAN | Q9NPH9 | Interleukin-26 |  |  |  |
| IL36A_HUMAN | Q9UHA7 | Interleukin-36 alpha |  |  |  |
| IL37_HUMAN | Q9NZH6 | Interleukin-37 |  |  |  |
| IL4RA_HUMAN | P24394 | Interleukin-4 receptor subunit alpha |  |  |  |
| IL6_HUMAN | P05231 | Interleukin-6 | + |  |  |
| IL8_HUMAN | P10145 | Interleukin-8 |  |  |  |
| IMB1_HUMAN | Q14974 | Importin subunit beta-1 |  |  | + |
| INHBB_HUMAN | P09529 | Inhibin beta B chain |  |  |  |
| IPYR_HUMAN | Q15181 | Inorganic pyrophosphatase |  |  | + |
| IQGA1_HUMAN | P46940 | Ras GTPase-activating-like protein IQGAP1 |  | + | + |
| ISOC1_HUMAN | Q96CN7 | Isochorismatase domain-containing protein 1 |  |  | + |
| ITIH1_HUMAN | P19827 | Inter-alpha-trypsin inhibitor heavy chain H1 |  |  |  |
| ITIH2_HUMAN | P19823 | Inter-alpha-trypsin inhibitor heavy chain H2 |  |  |  |
| ITIH4_HUMAN | Q14624 | Inter-alpha-trypsin inhibitor heavy chain H4 |  |  |  |
| K1C10_HUMAN | P13645 | Keratin, type I cytoskeletal 10 |  | + | + |
| K1C9_HUMAN | P35527 | Keratin, type I cytoskeletal 9 |  | + | + |
| K22E_HUMAN | P35908 | Keratin, type II cytoskeletal 2 epidermal |  |  | + |
| K2C1_HUMAN | P04264 | Keratin, type II cytoskeletal 1 |  | + | + |
| KAD1_HUMAN | P00568 | Adenylate kinase isoenzyme 1 |  |  | + |
| KAD2_HUMAN | P54819 | Adenylate kinase 2, mitochondrial |  | + |  |
| KAD3_HUMAN | Q9UIJ7 | GTP:AMP phosphotransferase AK3, mitochondrial |  | + | + |
| KAP3_HUMAN | P31323 | cAMP-dependent protein kinase type II-beta regulatory subunit |  |  | + |
| KCD12_HUMAN | Q96CX2 | BTB/POZ domain-containing protein KCTD12 |  | + |  |
| KCRB_HUMAN | P12277 | Creatine kinase B-type |  | + | + |
| KLKB1_HUMAN | P03952 | Plasma kallikrein |  |  |  |
| KREM2_HUMAN | Q8NCW0 | Kremen protein 2 |  |  |  |
| KV106_HUMAN | P01598 | Ig kappa chain V-I region EU |  |  |  |
| KV114_HUMAN | P01606 | Ig kappa chain V-I region OU |  |  |  |
| KV121_HUMAN | P01613 | Ig kappa chain V-I region Ni |  |  |  |
| KV202_HUMAN | P01615 | Ig kappa chain V-II region FR |  |  |  |
| KV204_HUMAN | P01617 | Ig kappa chain V-II region TEW |  |  |  |
| KV206_HUMAN | P06310 | Ig kappa chain V-II region RPMI 6410 |  |  | + |
| KV302_HUMAN | P01620 | Ig kappa chain V-III region SIE |  |  |  |
| KV307_HUMAN | P04206 | Ig kappa chain V-III region GOL |  |  |  |
| KV308_HUMAN | P04207 | Ig kappa chain V-III region CLL |  |  |  |
| KV402_HUMAN | P01625 | Ig kappa chain V-IV region Len |  |  |  |
| LAC2_HUMAN | P0CG05 | Ig lambda-2 chain C regions | + |  |  |
| LAC3_HUMAN | P0CG06 | Ig lambda-3 chain C regions |  |  |  |
| LAC7_HUMAN | A0M8Q6 | Ig lambda-7 chain C region |  |  |  |
| LAMA2_HUMAN | P24043 | Laminin subunit alpha-2 |  | + | + |
| LAMA4_HUMAN | Q16363 | Laminin subunit alpha-4 | + | + |  |
| LAMB1_HUMAN | P07942 | Laminin subunit beta-1 | + | + | + |
| LAMB2_HUMAN | P55268 | Laminin subunit beta-2 | + | + | + |
| LAMC1_HUMAN | P11047 | Laminin subunit gamma-1 | + | + | + |
| LBP_HUMAN | P18428 | Lipopolysaccharide-binding protein |  |  |  |
| LCK_HUMAN | P06239 | Tyrosine-protein kinase Lck |  |  |  |
| LCN1_HUMAN | P31025 | Lipocalin-1 |  |  |  |
| LDHA_HUMAN | P00338 | L-lactate dehydrogenase A chain | + | + |  |
| LDHB_HUMAN | P07195 | L-lactate dehydrogenase B chain | + | + | + |
| LEG1_HUMAN | P09382 | Galectin-1 |  | + | + |
| LEG3_HUMAN | P17931 | Galectin-3 | + | + | + |
| LEPR_HUMAN | P48357 | Leptin receptor |  |  |  |
| LEP_HUMAN | P41159 | Leptin (Ob) |  |  |  |
| LFTY2_HUMAN | O00292 | Left-right determination factor 2 |  |  |  |
| LG3BP_HUMAN | Q08380 | Galectin-3-binding protein | + | + | + |
| LGUL_HUMAN | Q04760 | Lactoylglutathione lyase | + |  | + |
| LHPP_HUMAN | Q9H008 | Phospholysine phosphohistidine inorganic pyrophosphate phosphatase |  |  | + |
| LIF_HUMAN | P15018 | Leukemia inhibitory factor | + |  |  |
| LIFR_HUMAN | P42702 | Leukemia inhibitory factor receptor |  |  |  |
| LIPS_HUMAN | Q05469 | Hormone-sensitive lipase |  |  |  |
| LIS1_HUMAN | P43034 | Platelet-activating factor acetylhydrolase IB subunit alpha | + |  |  |
| LMNA_HUMAN | P02545 | Prelamin-A/C |  | + |  |
| LMNB2_HUMAN | Q03252 | Lamin-B2 |  |  | + |
| LRP6_HUMAN | O75581 | Low-density lipoprotein receptor-related protein 6 |  |  |  |
| LUM_HUMAN | P51884 | Lumican | + | + |  |
| LV102_HUMAN | P01700 | Ig lambda chain V-I region HA |  |  |  |
| LV301_HUMAN | P01714 | Ig lambda chain V-III region SH |  |  |  |
| LV302_HUMAN | P80748 | Ig lambda chain V-III region LOI |  |  |  |
| LYSC_HUMAN | P61626 | Lysozyme C | + |  |  |
| MAP4_HUMAN | P27816 | Microtubule-associated protein 4 |  |  |  |
| MAT2B_HUMAN | Q9NZL9 | Methionine adenosyltransferase 2 subunit beta |  |  |  |
| MATR3_HUMAN | P43243 | Matrin-3 |  |  | + |
| MDHC_HUMAN | P40925 | Malate dehydrogenase, cytoplasmic | + | + | + |
| MDHM_HUMAN | P40926 | Malate dehydrogenase, mitochondrial | + | + | + |
| MFGM_HUMAN | Q08431 | Lactadherin |  |  |  |
| MGLL_HUMAN | Q99685 | Monoglyceride lipase |  |  | + |
| MIF_HUMAN | P14174 | Macrophage migration inhibitory factor |  |  |  |
| MIME_HUMAN | P20774 | Mimecan | + |  |  |
| MMP1_HUMAN | P03956 | Interstitial collagenase (Matrix metalloproteinase-1) | + |  |  |
| MMP10_HUMAN | P09238 | Stromelysin-2 (Matrix metalloproteinase-10) |  |  |  |
| MMP2_HUMAN | P08253 | 72 kDa type IV collagenase (Matrix metalloproteinase-2) | + | + |  |
| MMP3_HUMAN | P08254 | Stromelysin-1 (Matrix metalloproteinase-3) |  |  |  |
| MMP7_HUMAN | P09237 | Matrilysin (Matrix metalloproteinase-7) |  |  |  |
| MMP8_HUMAN | P22894 | Neutrophil collagenase (Matrix metalloproteinase-8) |  |  |  |
| MMP9_HUMAN | P14780 | Matrix metalloproteinase-9 | + | + |  |
| MOES_HUMAN | P26038 | Moesin | + |  | + |
| MRC1_HUMAN | P22897 | Macrophage mannose receptor 1 |  |  |  |
| MTAP_HUMAN | Q13126 | S-methyl-5'-thioadenosine phosphorylase |  |  | + |
| MUC18_HUMAN | P43121 | Cell surface glycoprotein MUC18 | + | + |  |
| MUCB_HUMAN | P04220 | Ig mu heavy chain disease protein |  |  |  |
| MYH9_HUMAN | P35579 | Myosin-9 |  |  |  |
| MYL6_HUMAN | P60660 | Myosin light polypeptide 6 |  | + |  |
| MYO1C_HUMAN | O00159 | Unconventional myosin-Ic |  |  |  |
| NACAM_HUMAN | E9PAV3 | Nascent polypeptide-associated complex subunit alpha, muscle-specific form |  |  |  |
| NAGK_HUMAN | Q9UJ70 | N-acetyl-D-glucosamine kinase |  |  | + |
| NB5R3_HUMAN | P00387 | NADH-cytochrome b5 reductase 3 |  |  |  |
| NBL1_HUMAN | P41271 | Neuroblastoma suppressor of tumorigenicity 1 |  |  |  |
| NDKB_HUMAN | P22392 | Nucleoside diphosphate kinase B | + | + |  |
| NID1_HUMAN | P14543 | Nidogen-1 | + | + |  |
| NID2_HUMAN | Q14112 | Nidogen-2 | + | + | + |
| NIT2_HUMAN | Q9NQR4 | Omega-amidase NIT2 |  |  | + |
| NNRE_HUMAN | Q8NCW5 | NAD(P)H-hydrate epimerase |  | + |  |
| NQO1_HUMAN | P15559 | NAD(P)H dehydrogenase [quinone] 1 |  |  | + |
| NRG1_HUMAN | Q02297 | Pro-neuregulin-1 |  |  |  |
| NRG2_HUMAN | O14511 | Pro-neuregulin-2 |  |  |  |
| NRN1_HUMAN | Q9NPD7 | Neuritin |  |  |  |
| NRTN_HUMAN | Q99748 | Neurturin |  |  |  |
| NUDT5_HUMAN | Q9UKK9 | ADP-sugar pyrophosphatase |  |  | + |
| NUMA1_HUMAN | Q14980 | Nuclear mitotic apparatus protein 1 |  |  |  |
| ONCM_HUMAN | P13725 | Oncostatin-M |  |  |  |
| OPLA_HUMAN | O14841 | 5-oxoprolinase |  |  | + |
| OREX_HUMAN | O43612 | Orexin |  |  |  |
| OTUB1_HUMAN | Q96FW1 | Ubiquitin thioesterase OTUB1 |  |  |  |
| PAI1_HUMAN | P05121 | Plasminogen activator inhibitor 1 | + | + |  |
| PARK7_HUMAN | Q99497 | Protein DJ-1 |  | + | + |
| PARVA_HUMAN | Q9NVD7 | Alpha-parvin |  |  |  |
| PCBP1_HUMAN | Q15365 | Poly(rC)-binding protein 1 |  | + | + |
| PD2R2_HUMAN | Q9Y5Y4 | Prostaglandin D2 receptor 2 |  |  |  |
| PDCD6_HUMAN | O75340 | Programmed cell death protein 6 |  |  | + |
| PDIA3_HUMAN | P30101 | Protein disulfide-isomerase A3 | + | + | + |
| PDIA6_HUMAN | Q15084 | Protein disulfide-isomerase A6 | + | + |  |
| PDXK_HUMAN | O00764 | Pyridoxal kinase |  | + |  |
| PEA15_HUMAN | Q15121 | Astrocytic phosphoprotein PEA-15 |  | + | + |
| PEBP1_HUMAN | P30086 | Phosphatidylethanolamine-binding protein 1 | + | + | + |
| PEDF_HUMAN | P36955 | Pigment epithelium-derived factor | + | + |  |
| PGAM1_HUMAN | P18669 | Phosphoglycerate mutase 1 | + | + | + |
| PGBM_HUMAN | P98160 | Basement membrane-specific heparan sulfate proteoglycan core protein | + | + | + |
| PGK1_HUMAN | P00558 | Phosphoglycerate kinase 1 | + |  | + |
| PGM1_HUMAN | P36871 | Phosphoglucomutase-1 | + |  |  |
| PGRC2_HUMAN | O15173 | Membrane-associated progesterone receptor component 2 |  |  | + |
| PHS_HUMAN | P61457 | Pterin-4-alpha-carbinolamine dehydratase |  |  | + |
| PIGR_HUMAN | P01833 | Polymeric immunoglobulin receptor |  |  |  |
| PIP_HUMAN | P12273 | Prolactin-inducible protein |  |  |  |
| PIPNB_HUMAN | P48739 | Phosphatidylinositol transfer protein beta isoform |  |  |  |
| PLEC_HUMAN | Q15149 | Plectin |  | + |  |
| PLIN1_HUMAN | O60240 | Perilipin-1 |  |  | + |
| PLIN3_HUMAN | O60664 | Perilipin-3 |  |  |  |
| PLIN4_HUMAN | Q96Q06 | Perilipin-4 |  | + |  |
| PLMN_HUMAN | P00747 | Plasminogen |  |  |  |
| PLTP_HUMAN | P55058 | Phospholipid transfer protein |  | + |  |
| PMGE_HUMAN | P07738 | Bisphosphoglycerate mutase |  |  |  |
| POTEF_HUMAN | A5A3E0 | POTE ankyrin domain family member F |  |  |  |
| POTEI_HUMAN | P0CG38 | POTE ankyrin domain family member I |  |  |  |
| PP1A_HUMAN | P62136 | Serine/threonine-protein phosphatase PP1-alpha catalytic subunit |  |  |  |
| PPIA_HUMAN | P62937 | Peptidyl-prolyl cis-trans isomerase A | + | + | + |
| PPIB_HUMAN | P23284 | Peptidyl-prolyl cis-trans isomerase B | + | + | + |
| PRDBP_HUMAN | Q969G5 | Protein kinase C delta-binding protein |  |  | + |
| PRDX1_HUMAN | Q06830 | Peroxiredoxin-1 | + | + | + |
| PRDX2_HUMAN | P32119 | Peroxiredoxin-2 | + | + | + |
| PRDX3_HUMAN | P30048 | Thioredoxin-dependent peroxide reductase, mitochondrial | + | + | + |
| PRDX5_HUMAN | P30044 | Peroxiredoxin-5, mitochondrial |  | + |  |
| PRDX6_HUMAN | P30041 | Peroxiredoxin-6 | + | + | + |
| PROF1_HUMAN | P07737 | Profilin-1 | + | + | + |
| PROK1_HUMAN | P58294 | Prokineticin-1 |  |  |  |
| PRS8_HUMAN | P62195 | 26S protease regulatory subunit 8 |  |  | + |
| PSA_HUMAN | P55786 | Puromycin-sensitive aminopeptidase |  |  | + |
| PSA1_HUMAN | P25786 | Proteasome subunit alpha type-1 | + |  |  |
| PSA2_HUMAN | P25787 | Proteasome subunit alpha type-2 | + |  | + |
| PSA3_HUMAN | P25788 | Proteasome subunit alpha type-3 | + |  |  |
| PSA4_HUMAN | P25789 | Proteasome subunit alpha type-4 | + |  | + |
| PSA5_HUMAN | P28066 | Proteasome subunit alpha type-5 |  |  | + |
| PSA6_HUMAN | P60900 | Proteasome subunit alpha type-6 | + |  | + |
| PSA7_HUMAN | O14818 | Proteasome subunit alpha type-7 | + |  |  |
| PSB1_HUMAN | P20618 | Proteasome subunit beta type-1 | + |  | + |
| PSB2_HUMAN | P49721 | Proteasome subunit beta type-2 |  |  | + |
| PSB3_HUMAN | P49720 | Proteasome subunit beta type-3 | + |  | + |
| PSB4_HUMAN | P28070 | Proteasome subunit beta type-4 | + |  | + |
| PSB5_HUMAN | P28074 | Proteasome subunit beta type-5 | + |  | + |
| PSB6_HUMAN | P28072 | Proteasome subunit beta type-6 |  |  | + |
| PSD13_HUMAN | Q9UNM6 | 26S proteasome non-ATPase regulatory subunit 13 |  |  | + |
| PSDE_HUMAN | O00487 | 26S proteasome non-ATPase regulatory subunit 14 |  |  | + |
| PSMD2_HUMAN | Q13200 | 26S proteasome non-ATPase regulatory subunit 2 |  |  | + |
| PSMD6_HUMAN | Q15008 | 26S proteasome non-ATPase regulatory subunit 6 |  |  | + |
| PSMD7_HUMAN | P51665 | 26S proteasome non-ATPase regulatory subunit 7 |  |  | + |
| PSME1_HUMAN | Q06323 | Proteasome activator complex subunit 1 |  |  | + |
| PSME2_HUMAN | Q9UL46 | Proteasome activator complex subunit 2 |  |  | + |
| PTBP1_HUMAN | P26599 | Polypyrimidine tract-binding protein 1 |  | + |  |
| PTER_HUMAN | Q96BW5 | Phosphotriesterase-related protein |  |  | + |
| PTGR1_HUMAN | Q14914 | Prostaglandin reductase 1 |  |  | + |
| PTMA_HUMAN | P06454 | Prothymosin alpha |  |  |  |
| PTN11_HUMAN | Q06124 | Tyrosine-protein phosphatase non-receptor type 11 |  |  |  |
| PTPA_HUMAN | Q15257 | Serine/threonine-protein phosphatase 2A activator |  |  |  |
| PTPRS_HUMAN | Q13332 | Receptor-type tyrosine-protein phosphatase S |  |  |  |
| PTRF_HUMAN | Q6NZI2 | Polymerase I and transcript release factor |  |  |  |
| PUR6_HUMAN | P22234 | Multifunctional protein ADE2 |  |  | + |
| PXDC2_HUMAN | Q6UX71 | Plexin domain-containing protein 2 |  |  |  |
| PYGB_HUMAN | P11216 | Glycogen phosphorylase, brain form |  |  | + |
| PYGL_HUMAN | P06737 | Glycogen phosphorylase, liver form | + |  | + |
| PZP_HUMAN | P20742 | Pregnancy zone protein |  |  |  |
| Q9NS53_HUMAN | Q9NS53 | Preadipocyte factor |  |  |  |
| QOR_HUMAN | Q08257 | Quinone oxidoreductase | + |  | + |
| RAB1A_HUMAN | P62820 | Ras-related protein Rab-1A |  |  |  |
| RAB2A_HUMAN | P61019 | Ras-related protein Rab-2A |  |  | + |
| RAB5C_HUMAN | P51148 | Ras-related protein Rab-5C |  |  | + |
| RAP1A_HUMAN | P62834 | Ras-related protein Rap-1A |  |  | + |
| REEP6_HUMAN | Q96HR9 | Receptor expression-enhancing protein 6 |  |  | + |
| RET4_HUMAN | P02753 | Retinol-binding protein 4 |  | + | + |
| RHOA_HUMAN | P61586 | Transforming protein RhoA |  |  | + |
| RINI_HUMAN | P13489 | Ribonuclease inhibitor |  |  | + |
| RLA0_HUMAN | P05388 | 60S acidic ribosomal protein P0 |  |  | + |
| RLA1_HUMAN | P05386 | 60S acidic ribosomal protein P1 |  |  | + |
| RNAS4_HUMAN | P34096 | Ribonuclease 4 |  | + |  |
| ROBO4_HUMAN | Q8WZ75 | Roundabout homolog 4 |  |  |  |
| RRAS2_HUMAN | P62070 | Ras-related protein R-Ras2 |  |  | + |
| RRBP1_HUMAN | Q9P2E9 | Ribosome-binding protein 1 |  |  |  |
| RS25_HUMAN | P62851 | 40S ribosomal protein S25 |  |  | + |
| RS27A_HUMAN | P62979 | Ubiquitin-40S ribosomal protein S27a |  |  | + |
| RS3_HUMAN | P23396 | 40S ribosomal protein S3 |  |  | + |
| RSSA_HUMAN | P08865 | 40S ribosomal protein SA |  |  | + |
| RSU1_HUMAN | Q15404 | Ras suppressor protein 1 |  |  | + |
| RTN1_HUMAN | Q16799 | Reticulon-1 |  |  |  |
| RTN4_HUMAN | Q9NQC3 | Reticulon-4 |  |  |  |
| S10A1_HUMAN | P23297 | Protein S100-A1 |  |  | + |
| S10A4_HUMAN | P26447 | Protein S100-A4 |  | + | + |
| S10A6_HUMAN | P06703 | Protein S100-A6 |  |  | + |
| S10A8_HUMAN | P05109 | Protein S100-A8 |  |  |  |
| S10A9_HUMAN | P06702 | Protein S100-A9 |  |  |  |
| S10AA_HUMAN | P60903 | Protein S100-A10 |  | + | + |
| S10AB_HUMAN | P31949 | Protein S100-A11 |  | + | + |
| S10AD_HUMAN | Q99584 | Protein S100-A13 |  |  | + |
| S10AG_HUMAN | Q96FQ6 | Protein S100-A16 |  |  | + |
| SAA4_HUMAN | P35542 | Serum amyloid A-4 protein |  |  |  |
| SAHH_HUMAN | P23526 | Adenosylhomocysteinase |  | + | + |
| SAMP_HUMAN | P02743 | Serum amyloid P-component |  |  |  |
| SAP_HUMAN | P07602 | Prosaposin |  | + |  |
| SBP1_HUMAN | Q13228 | Selenium-binding protein 1 | + | + | + |
| SC22B_HUMAN | O75396 | Vesicle-trafficking protein SEC22b |  |  | + |
| SCRN2_HUMAN | Q96FV2 | Secernin-2 |  | + | + |
| SDPR_HUMAN | O95810 | Serum deprivation-response protein |  |  | + |
| SEPT2_HUMAN | Q15019 | Septin-2 |  |  | + |
| SGTA_HUMAN | O43765 | Small glutamine-rich tetratricopeptide repeat-containing protein alpha |  |  |  |
| SH3L1_HUMAN | O75368 | SH3 domain-binding glutamic acid-rich-like protein | + | + | + |
| SH3L3_HUMAN | Q9H299 | SH3 domain-binding glutamic acid-rich-like protein 3 |  | + | + |
| SIGIR_HUMAN | Q6IA17 | Single Ig IL-1-related receptor |  |  |  |
| SIGL9_HUMAN | Q9Y336 | Sialic acid-binding Ig-like lectin 9 |  |  |  |
| SMD1_HUMAN | P62314 | Small nuclear ribonucleoprotein Sm D1 |  |  | + |
| SNAA_HUMAN | P54920 | Alpha-soluble NSF attachment protein |  |  | + |
| SODC_HUMAN | P00441 | Superoxide dismutase [Cu-Zn] | + | + | + |
| SODE_HUMAN | P08294 | Extracellular superoxide dismutase [Cu-Zn] | + | + | + |
| SODM_HUMAN | P04179 | Superoxide dismutase [Mn], mitochondrial | + | + | + |
| SORCN_HUMAN | P30626 | Sorcin |  |  | + |
| SPB6_HUMAN | P35237 | Serpin B6 |  | + | + |
| SPRC_HUMAN | P09486 | SPARC | + | + | + |
| SPRE_HUMAN | P35270 | Sepiapterin reductase |  |  | + |
| SPRL1_HUMAN | Q14515 | SPARC-like protein 1 |  |  |  |
| SPTB2_HUMAN | Q01082 | Spectrin beta chain, non-erythrocytic 1 |  |  |  |
| SPTN1_HUMAN | Q13813 | Spectrin alpha chain, non-erythrocytic 1 |  |  |  |
| SRBS1_HUMAN | Q9BX66 | Sorbin and SH3 domain-containing protein 1 |  |  |  |
| SRSF7_HUMAN | Q16629 | Serine/arginine-rich splicing factor 7 |  |  |  |
| STOM_HUMAN | P27105 | Erythrocyte band 7 integral membrane protein |  |  | + |
| STX7_HUMAN | O15400 | Syntaxin-7 |  |  |  |
| SYUG_HUMAN | O76070 | Gamma-synuclein |  |  | + |
| TAGL_HUMAN | Q01995 | Transgelin | + | + | + |
| TAGL2_HUMAN | P37802 | Transgelin-2 |  | + | + |
| TALDO_HUMAN | P37837 | Transaldolase | + | + | + |
| TBA1B_HUMAN | P68363 | Tubulin alpha-1B chain |  |  |  |
| TBB5_HUMAN | P07437 | Tubulin beta chain |  |  | + |
| TBC9B_HUMAN | Q66K14 | TBC1 domain family member 9B |  |  |  |
| TBCA_HUMAN | O75347 | Tubulin-specific chaperone A |  |  | + |
| TCPZ_HUMAN | P40227 | T-complex protein 1 subunit zeta |  |  | + |
| TEFF1_HUMAN | Q8IYR6 | Tomoregulin-1 |  |  |  |
| TENS1_HUMAN | Q9HBL0 | Tensin-1 |  |  |  |
| TENX_HUMAN | P22105 | Tenascin-X | + |  |  |
| TERA_HUMAN | P55072 | Transitional endoplasmic reticulum ATPase | + |  | + |
| TETN_HUMAN | P05452 | Tetranectin |  | + |  |
| TF_HUMAN | P13726 | Tissue factor |  |  |  |
| TGFB2_HUMAN | P61812 | Transforming growth factor beta-2 |  |  |  |
| TGFR1_HUMAN | P36897 | TGF-beta receptor type-1 |  |  |  |
| THIC_HUMAN | Q9BWD1 | Acetyl-CoA acetyltransferase, cytosolic |  |  | + |
| THIL_HUMAN | P24752 | Acetyl-CoA acetyltransferase, mitochondrial |  |  | + |
| THIM_HUMAN | P42765 | 3-ketoacyl-CoA thiolase, mitochondrial |  |  | + |
| THIO_HUMAN | P10599 | Thioredoxin |  | + | + |
| THRB_HUMAN | P00734 | Prothrombin |  |  |  |
| TIMP1_HUMAN | P01033 | Metalloproteinase inhibitor 1 | + | + |  |
| TKT_HUMAN | P29401 | Transketolase | + | + | + |
| TLN1_HUMAN | Q9Y490 | Talin-1 |  |  | + |
| TLN2_HUMAN | Q9Y4G6 | Talin-2 |  |  | + |
| TNFC_HUMAN | Q06643 | Lymphotoxin-beta |  |  |  |
| TNFL8_HUMAN | P32971 | Tumor necrosis factor ligand superfamily member 8 |  |  |  |
| TNR11_HUMAN | Q9Y6Q6 | Tumor necrosis factor receptor superfamily member 11A |  |  |  |
| TNR18_HUMAN | Q9Y5U5 | Tumor necrosis factor receptor superfamily member 18 |  |  |  |
| TPIS_HUMAN | P60174 | Triosephosphate isomerase | + | + |  |
| TPM3_HUMAN | P06753 | Tropomyosin alpha-3 chain |  |  |  |
| TPM4_HUMAN | P67936 | Tropomyosin alpha-4 chain |  |  |  |
| TPO_HUMAN | P40225 | Thrombopoietin |  |  |  |
| TR10B_HUMAN | O14763 | Tumor necrosis factor receptor superfamily member 10B |  |  |  |
| TR11B_HUMAN | O00300 | Tumor necrosis factor receptor superfamily member 11B |  |  |  |
| TR19L_HUMAN | Q969Z4 | Tumor necrosis factor receptor superfamily member 19L |  |  |  |
| TRADD_HUMAN | Q15628 | Tumor necrosis factor receptor type 1-associated DEATH domain protein |  |  |  |
| TRFE_HUMAN | P02787 | Serotransferrin | + | + | + |
| TRFL_HUMAN | P02788 | Lactotransferrin | + | + |  |
| TRYB1_HUMAN | Q15661 | Tryptase alpha/beta-1 |  | + |  |
| TSG6_HUMAN | P98066 | Tumor necrosis factor-inducible gene 6 protein | + |  |  |
| TSNAX_HUMAN | Q99598 | Translin-associated protein X |  |  | + |
| TSP1_HUMAN | P07996 | Thrombospondin-1 | + | + |  |
| TSP2_HUMAN | P35442 | Thrombospondin-2 | + | + |  |
| TSP4_HUMAN | P35443 | Thrombospondin-4 |  |  |  |
| TTHY_HUMAN | P02766 | Transthyretin |  |  | + |
| TXND5_HUMAN | Q8NBS9 | Thioredoxin domain-containing protein 5 | + | + | + |
| TXNL1_HUMAN | O43396 | Thioredoxin-like protein 1 |  |  | + |
| UB2V1_HUMAN | Q13404 | Ubiquitin-conjugating enzyme E2 variant 1 |  |  |  |
| UBA1_HUMAN | P22314 | Ubiquitin-like modifier-activating enzyme 1 |  | + | + |
| UBE2N_HUMAN | P61088 | Ubiquitin-conjugating enzyme E2 N |  |  | + |
| UBP14_HUMAN | P54578 | Ubiquitin carboxyl-terminal hydrolase 14 |  |  | + |
| UCHL1_HUMAN | P09936 | Ubiquitin carboxyl-terminal hydrolase isozyme L1 |  | + | + |
| UFM1_HUMAN | P61960 | Ubiquitin-fold modifier 1 |  |  | + |
| UGGG1_HUMAN | Q9NYU2 | UDP-glucose:glycoprotein glucosyltransferase 1 |  |  |  |
| UGPA_HUMAN | Q16851 | UTP--glucose-1-phosphate uridylyltransferase | + | + |  |
| UK114_HUMAN | P52758 | Ribonuclease UK114 |  |  | + |
| UROK_HUMAN | P00749 | Urokinase-type plasminogen activator |  |  |  |
| VASN_HUMAN | Q6EMK4 | Vasorin |  | + |  |
| VAT1_HUMAN | Q99536 | Synaptic vesicle membrane protein VAT-1 homolog |  |  | + |
| VEGFA_HUMAN | P15692 | Vascular endothelial growth factor A |  |  |  |
| VEGFD_HUMAN | O43915 | Vascular endothelial growth factor D |  | + |  |
| VIME_HUMAN | P08670 | Vimentin | + | + | + |
| VINC_HUMAN | P18206 | Vinculin | + | + |  |
| VP26A_HUMAN | O75436 | Vacuolar protein sorting-associated protein 26A | + | + | + |
| VPS29_HUMAN | Q9UBQ0 | Vacuolar protein sorting-associated protein 29 |  |  |  |
| VPS35_HUMAN | Q96QK1 | Vacuolar protein sorting-associated protein 35 |  |  | + |
| VTDB_HUMAN | P02774 | Vitamin D-binding protein |  |  |  |
| VTNC_HUMAN | P04004 | Vitronectin |  |  |  |
| VWF_HUMAN | P04275 | von Willebrand factor | + |  |  |
| WFKN2_HUMAN | Q8TEU8 | WAP, Kazal, immunoglobulin, Kunitz and NTR domain-containing protein 2 |  |  |  |
| ZA2G_HUMAN | P25311 | Zinc-alpha-2-glycoprotein |  | + |  |
